# Fungal Biotransformation of Chloroflavanones and Antimicrobial Activity of Parent Compounds and Derived Products

**DOI:** 10.3390/ijms262010138

**Published:** 2025-10-18

**Authors:** Agnieszka Krawczyk-Łebek, Tomasz Janeczko, Barbara Żarowska, Edyta Kostrzewa-Susłow

**Affiliations:** 1 Department of Food Chemistry and Biocatalysis, Faculty of Biotechnology and Food Science, Wrocław University of Environmental and Life Sciences, 50-375 Wrocław, Poland; tomasz.janeczko@upwr.edu.pl (T.J.); edyta.kostrzewa-suslow@upwr.edu.pl (E.K.-S.); 2 Department of Biotechnology and Food Microbiology, Faculty of Biotechnology and Food Science, Wrocław University of Environmental and Life Sciences, 51-630 Wrocław, Poland; barbara.zarowska@upwr.edu.pl

**Keywords:** biotransformations, flavanones with a chlorine atom, glycosylation, *Isaria fumosorosea*, *Beauveria bassiana*, antimicrobial activity

## Abstract

This study explores the synthesis of chlorine-substituted flavanones and their biotechnologically derived glycosides in order to evaluate how structural modifications influence both antimicrobial activity and pharmacokinetic properties, with attention to issues such as solubility and membrane transport. Four chloroflavanones (2′-, 3′-, 4′-, and 6-chloroflavanone) were synthesized and biotransformed using entomopathogenic fungi to obtain glycosylated derivatives. Antimicrobial activity was assessed against five microbial strains, while pharmacokinetic properties were predicted computationally. Results showed that 4′-chloroflavanone demonstrated the strongest antimicrobial activity, particularly against Gram-positive bacteria *Staphylococcus aureus* ATCC 29213 and *Enterococcus faecalis* ATCC 19433. Most compounds unexpectedly promoted *Escherichia coli* ATCC 25922 growth, except 4′-chloroflavanone and 3′-chloroflavanone 6-*O*-β-D-(4″-*O*-methyl)-glucopyranoside. Nearly all compounds exhibited antifungal activity against *Candida albicans* ATCC 10231. Glycosylation generally reduced antimicrobial potency but improved water solubility and in silico predictions indicate markedly reduced blood–brain barrier permeation and potential P-glycoprotein recognition. Selective chlorine substitution combined with biotechnological glycosylation may offer a route to antimicrobial flavonoids with improved aqueous solubility and favorable predicted pharmacokinetics.

## 1. Introduction

**Figure 1:**
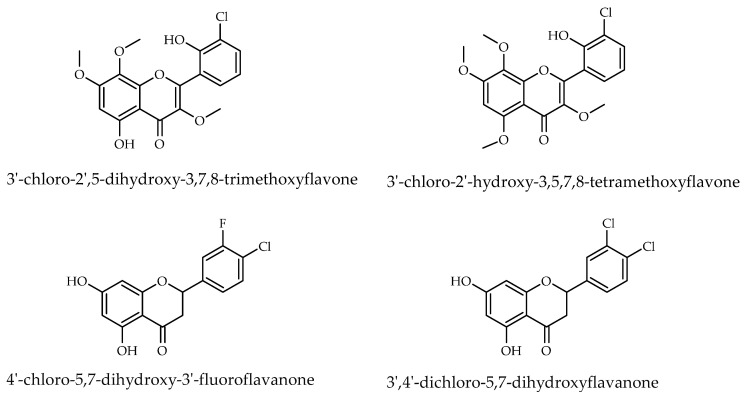
Structural formulas for 3′-chloro-2′,5-dihydroxy-3,7,8-trimethoxyflavone, 3′-chloro-2′-hydroxy-3,5,7,8-tetramethoxyflavone, 4′-chloro-5,7-dihydroxy-3′-fluoroflavanone and 3′,4′-dichloro-5,7-dihydroxyflavanone.

Flavonoids are a diverse class of plant secondary metabolites widely recognized for their broad spectrum of biological activities, including antimicrobial, anti-inflammatory, antioxidant, and anticancer properties. Structural modifications, such as halogenation and glycosylation, can significantly alter their physicochemical characteristics, membrane permeability, metabolic stability, and biological activity profiles [1,2,3,4,5,6,7,8,9]. In particular, chlorine substitution has been shown to modulate lipophilicity and electron distribution, thereby influencing target binding and antimicrobial potency [10,11]. Although relatively uncommon in nature compared to their non-halogenated analogues, chlorinated flavonoids have attracted considerable interest for their diverse biological activities and therapeutic potential [2,5,12]. Notable examples of natural flavonoids with a chlorine include 3′-chloro-2′,5-dihydroxy-3,7,8-trimethoxyflavone [13,14,15] and structurally related aspergivone A (3′-chloro-2′-hydroxy-3,5,7,8-tetramethoxyflavone) [16], both isolated from *Aspergillus candidus* fungus (Figure 1). Understanding how both chlorine substitution patterns and glycosylation affect antimicrobial activity could provide valuable insights for the design of more selective and potent bioactive molecules.

Consequently, efforts were directed toward synthesizing flavonoids and evaluating their biological activities. The resulting chloro-substituted flavonoids demonstrated anti-inflammatory properties, exhibiting enhanced efficacy in regulating neutrophil oxidative burst responses relative to their non-halogenated analogs [2,3,17,18]. One example of chlorinated flavanone research and biological activity evaluation is demonstrated by the synthesis of a series of 5,7-dihydroxyflavanone derivatives. Their antimicrobial efficacy was assessed against Gram-negative bacteria, Gram-positive bacteria, and yeast. Among these compounds, most halogenated derivatives, including 4′-chloro-5,7-dihydroxy-3′-fluoroflavanone and 3′,4′-dichloro-5,7-dihydroxyflavanone, displayed significant antimicrobial activity against Gram-positive ad Gram-negative bacteria and the yeast *Saccharomyces cerevisiae* [6].

Likewise, Kamboj and collaborators prepared a panel of chlorinated flavonoid compounds that displayed good to moderate antimicrobial activity against tested microbial strains [19]. The Fowler research team employed the natural flavonoid structure as a template to create non-natural flavanone compounds, subsequently testing their antimicrobial properties against various pathogens including *Escherichia coli*, *Bacillus subtilis*, *Cryptococcus neoformans*, and *Aspergillus fumigatus*. Through systematic screening, 4-chloroflavanone was identified as the lead antimicrobial compound, exhibiting potent activity with MIC values of 70 µg/mL against *E. coli* (in combination with Phe-Arg-β-naphthylamide) and 30 µg/mL against both *S. cerevisiae* and *C. neoformans* when used independently [7]. Given these findings, chloro-substituted flavonoids constitute an attractive research focus owing to their antimicrobial and anti-inflammatory characteristics, especially within structure–activity relationship investigations.

The biological activity and bioavailability of flavonoids can be altered also through the introduction of a glucose moiety. This modification improves their water solubility and, consequently, their bioavailability [20,21]. Glucosides are absorbed primarily in the small intestine, leading to higher plasma concentrations compared to other glycosides that are mainly absorbed in the colon [22,23]. Therefore, glycosylation represents an effective strategy to improve both the bioavailability and pharmacological efficacy of flavonoids. A promising approach involves microbial enzyme–mediated glycosylation, particularly via whole-cell biotransformation using filamentous fungi as biocatalysts [24,25,26]. Genome mining and heterologous expression have enabled the identification of functional glycosyltransferase–methyltransferase (GT–MT) modules in *Beauveria bassiana* fungi. These enzymes exhibit both substrate promiscuity and regiospecificity, enabling efficient methylglucosylation of flavonoids [27].

The presented study aimed to synthesize a series of four chloroflavanones (**1**–**4**) bearing chlorine substituents at distinct positions on the flavonoid framework and explore their enzymatic conversion by entomopathogenic fungi to yield corresponding glycosylated products. This study extends our previous work [25] on chlorinated flavones to the related but distinct class of chlorinated flavanones, whose non-planar scaffold and C-2 stereocenter lead to different hydroxylation and *O*-glycosylation patterns and strain-/substrate-dependent regioselectivity. Microbial transformation was conducted using two fungal strains, *Isaria fumosorosea* KCH J2 and *Beauveria bassiana* KCH J1.5, chosen for their broad enzymatic capabilities in previous research. These strains possess diverse glycosyltransferases, methyltransferases, and oxidative enzymes, enabling the biotransformation of a wide range of exogenous substrates, among others flavonoids. Entomopathogenic fungi such as *I. fumosorosea* (syn. *Cordyceps fumosorosea*) *B. bassiana* (syn. *Cordyceps bassiana*) are known to produce various secondary metabolites, including beauvericin, bassianolide, and fumosorinone and mostly serve as biopesticides. Despite their metabolic activity, they are regarded as environmentally friendly and have not been associated with the harmful effects typical of chemical pesticides [28,29,30,31]. Previous studies have demonstrated their efficiency in flavonoid modification, leading to the hypothesis that these fungi could effectively glycosylate chloroflavones, potentially yielding novel derivatives with unique biological properties [25,32,33].

## 2. Results and Discussion

**Figure 2:**
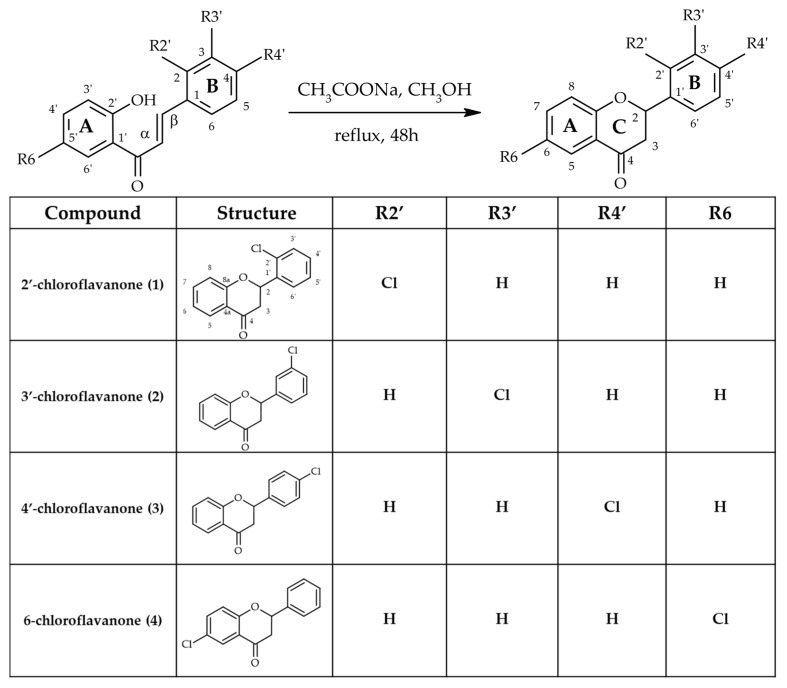
Chemical synthesis and structures of the obtained chloroflavanones (**1**–**4**). NMR (Nuclear Magnetic Resonance) spectroscopy was employed to confirm the structures of all prepared products (**1**–**4**), with data presented in Table 1, Table 2, Table 3 and Table 4. The obtained compounds were used as biotransformation’s substrates.

The initial phase of research focused on producing four chlorine-substituted flavanones: 2′-chloroflavanone, 3′-chloroflavanone, 4′-chloroflavanone, and 6-chloroflavanone. These chlorinated compounds were prepared through cyclization reactions of the corresponding 2′-hydroxychalcones containing chlorine substituents, using sodium acetate which acts as a base to deprotonate the 2′-hydroxyl group and facilitate the intramolecular cyclization reaction (Figure 2).

Two entomopathogenic filamentous fungal isolates, *Isaria fumosorosea* KCH J2 and *Beauveria bassiana* KCH J1.5, were utilized as biocatalytic agents for the biotransformation of chlorinated flavanone derivatives: 2′-chloroflavanone (**1**), 3′-chloroflavanone (**2**), 4′-chloroflavanone (**3**), and 6-chloroflavanone (**4**). The microbial transformation process yielded four previously unreported glycosylated flavonoid derivatives originating from substrates **1**, **2**, **3**, and **4**.

### 2.1. Biotransformation of 2′-Chloroflavanone (***1***) in Culture of B. bassiana KCH J1.5

**Figure 3:**
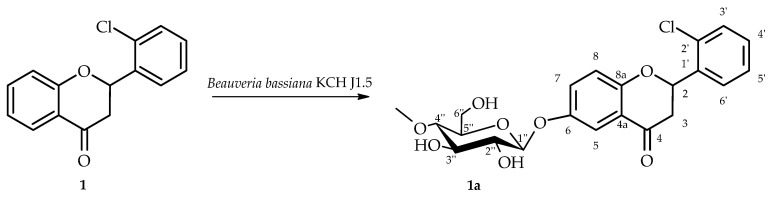
Biotransformation of 2′-chloroflavanone (**1**) in *B. bassiana* KCH J1.5 culture.

2′-Chloroflavanone (**1**) was biotransformed in culture of *B. bassiana* KCH J1.5 into 2′-chloroflavanone 6-*O*-*β*-D-(4″-*O*-methyl)-glucopyranoside (**1a**) yielding 6.6% (5.8 mg) (Figure 3). This strain–substrate pattern was first observed at the screening scale (Section 3.4) and subsequently confirmed in the semipreparative experiments.

**Figure 4:**
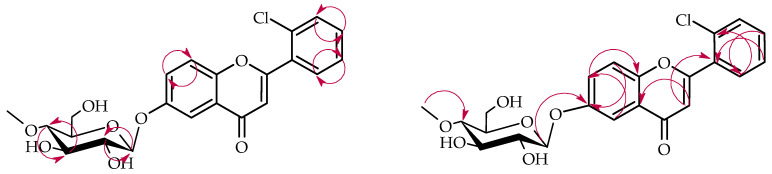
Key COSY (on the **left**) and HMBC (on the **right**) correlations for the structure elucidation of product **1a**.

NMR spectroscopy was employed to elucidate the structure of compound **1a**, with spectral data presented in Table 1 and Table 2. Critical COSY (COrrelation SpectroscopY) and HMBC (Heteronuclear Multiple Bond Correlation) correlations are depicted in Figure 4.

NMR spectroscopic analysis confirmed the presence of a glucose unit in biotransformation product **1a**. Five distinctive carbon signals appeared in the ^13^C-NMR spectrum within the δ = 62.0–80.0 ppm range (Supplementary Information: Figure S24), while the ^1^H-NMR spectrum showed corresponding proton signals within the δ = 3.24–3.83 ppm range (Supplementary Information: Figure S21). The glucose anomeric configuration was determined to be *β* based on the anomeric proton signal appearing as a doublet at δ = 4.92 ppm with a coupling constant of *J* = 7.8 Hz, which is diagnostic for *β*-glucose (Supplementary Information: Figure S21). Evidence for *O*-methylation at the C-4″ glucose position was provided by a methyl singlet at δ = 3.57 ppm in the ^1^H-NMR spectrum and its corresponding carbon signal at δ = 60.5 ppm in the ^13^C-NMR spectrum (Supplementary Information: Figures S21 and S24). The HMBC experiment provided further evidence for the glucose unit’s structure. A correlation was observed between the -O-CH_3_ moiety and the C-4″ signal (δ = 80.0 ppm). This correlation confirms the position of the methoxy group substitution on the glucose unit (Supplementary Information: Figure S32). The attachment position of the 4″-*O*-methylglucosyl unit was elucidated through two principal spectroscopic approaches. Comparative analysis of ^1^H NMR spectra between substrate **1** and product **1a** showed characteristic ring A proton chemical shift changes (H-5: δ = 7.88 → 7.50 ppm; H-7: δ = 7.61 → 7.36 ppm; H-8: δ = 7.13 → 7.06 ppm) along with complete disappearance of the H-6 signal, indicating glycosylation at the C-6 position (Supplementary Information: Figures S3 and S20). HMBC correlation data provided corroborating evidence through observed cross-peaks between H-1″ (δ = 4.92 ppm), H-5 (δ = 7.50 ppm), H-7 (δ = 7.36 ppm), and H-8 (δ = 7.06 ppm) with the downfield-shifted C-6 carbon signal at δ = 153.4 ppm (Supplementary Information: Figure S34).

### 2.2. Biotransformation of 2′-Chloroflavanone (***1***) in Culture of I. fumosorosea KCH J2

2′-Chloroflavanone (**1**) was also biotransformed in culture of *I. fumosorosea* KCH J2, but the resulting products were formed in trace amounts that were too small to purify and characterize structurally using NMR analysis.

### 2.3. Biotransformation of 3′-Chloroflavanone (***2***) in Culture of B. bassiana KCH J1.5

**Figure 5:**
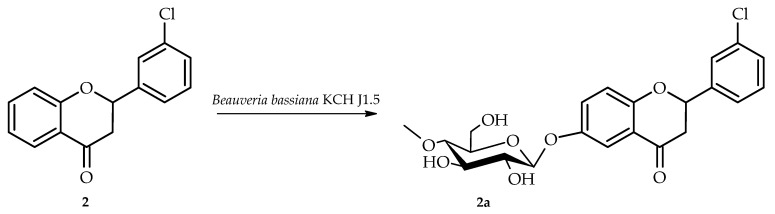
Biotransformation of 3′-chloroflavanone (**2**) in *B. bassiana* KCH J1.5 culture.

Biotransformation of 3′-chloroflavanone (**2**) in culture of *B. bassiana* KCH J1.5 resulted in the formation of one product, i.e., 3′-chloroflavanone 6-*O*-*β*-D-(4″-*O*-methyl)-glucopyranoside (**2a**) yielding 5.1% (4.5 mg) (Figure 5).

**Figure 6:**
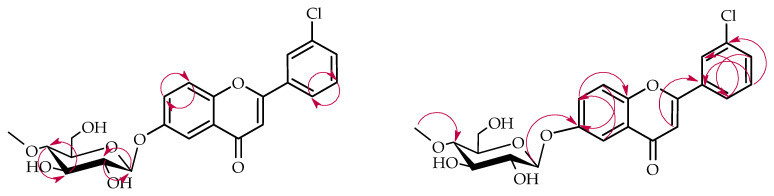
Key COSY (on the **left**) and HMBC (on the **right**) correlations for the structure elucidation of product **2a**.

The structure of product **2a** was also established based on NMR spectroscopy (Table 1 and Table 2, Figure 6 below with key COSY and HMBC correlations).

This biotransformation product also underwent 4″-*O*-methylglucosylation, as evidenced by characteristic NMR signals that were analogous to those previously described for product **1a** (Supplementary Information: Figures S56 and S59). Analysis of the ^1^H NMR chemical shifts for rings A and B indicated that glycosylation occurred on ring A, since the ring B proton signals remained unchanged from the starting compound. The attachment position of the 4″-O-methylglucosyl in product **2a** is C-6, the same as in product **1a**.Comparative analysis of ^1^H NMR spectra between substrate **2** and product **2a** showed characteristic ring A proton chemical shift changes (H-5: δ = 7.85 → 7.47 ppm; H-7: δ = 7.60 → 7.35 ppm; H-8: δ = 7.11 → 7.07 ppm) along with complete disappearance of the H-6 signal, indicating glycosylation at the C-6 position (Supplementary Information: Figures S38 and S55). HMBC correlation data provided corroborating evidence through observed cross-peaks between H-1″ (δ = 4.90 ppm), H-5 (δ = 7.47 ppm), H-7 (δ = 7.35 ppm), and H-8 (δ = 7.07 ppm) with the downfield-shifted C-6 carbon signal at δ = 153.3 ppm (Supplementary Information: Figures S67 and S69).

### 2.4. Biotransformation of 3′-Chloroflavanone (***2***) in Culture of I. fumosorosea KCH J2

Biotransformation of the same substrate 3′-chloroflavanone (**2**) in culture of another fungal strain *I. fumosorosea* KCH J2 resulted in the formation of a mixture of the products, which were formed in minute quantities, preventing adequate purification and subsequent NMR structural analysis.

### 2.5. Biotransformation of 4′-Chloroflavanone (***3***) in Culture of B. bassiana KCH J1.5

**Figure 7:**
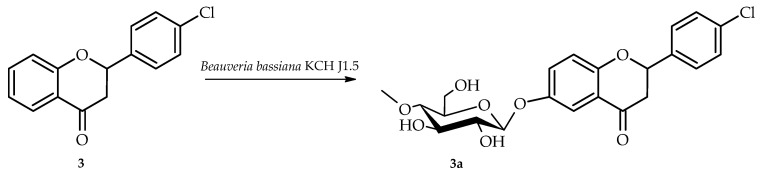
Biotransformation of 4′-chloroflavanone (**3**) in *B. bassiana* KCH J1.5 culture.

4′-Chloroflavanone (**3**) was biotransformed in culture of *B. bassiana* KCH J1.5 into 4′-chloroflavanone 6-*O*-*β*-D-(4″-*O*-methyl)-glucopyranoside (**3a**) yielding 5.8% (5.1 mg) (Figure 7).

**Figure 8:**
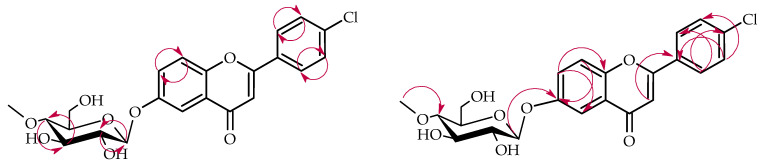
Key COSY (on the **left**) and HMBC (on the **right**) correlations for the structure elucidation of product **3a**.

The structure of product **3a** was established using NMR spectroscopy (Table 1 and Table 2, Figure 8 with key COSY and HMBC correlations). Like products **1a** and **2a**, compound **3a** also underwent 4″-O-methylglucosylation at the C-6 position, as confirmed by analogous NMR spectral features (Supplementary Information: Figures S91 and S94). This was evidenced by characteristic ring A proton downfield shifts (H-5: δ = 7.85 → 7.46 ppm; H-7: δ = 7.59 → 7.34 ppm; H-8: δ = 7.11 → 7.04 ppm) and loss of the H-6 signal (Supplementary Information: Figures S73 and S90), and HMBC correlations between the anomeric proton H-1″ (δ = 4.89 ppm) and ring A protons with the shifted C-6 carbon at δ = 153.3 ppm (Supplementary Information: Figure S104).

### 2.6. Biotransformation of 4′-Chloroflavanone (***3***) in Culture of I. fumosorosea KCH J2

Biotransformation of 4′-chloroflavanone (**3**) by *I. fumosorosea* KCH J2 strain also yielded multiple products, but these were obtained in quantities insufficient for isolation and structural characterization by NMR spectroscopy.

### 2.7. Biotransformation of 6-Chloroflavanone (***4***) in Cultures of I. fumosorosea KCH J2

**Figure 9:**
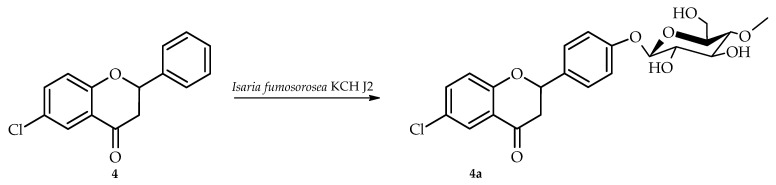
Biotransformation of 6-chloroflavanone (**4**) in *I. fumosorosea* KCH J2.

6-Chloroflavanone (**4**) was biotransformed in culture of *I. fumosorosea* KCH J2 into 6-chloroflavanone 4′-*O*-*β*-D-(4″-*O*-methyl)-glucopyranoside (**4a**) yielding 7.8% (6.8 mg) (Figure 9).

**Figure 10:**
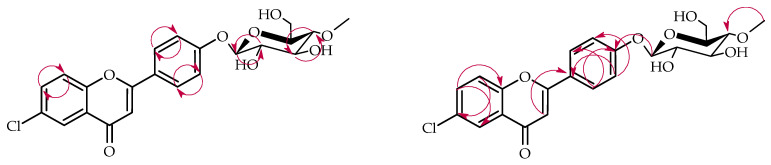
Key COSY (on the **left**) and HMBC (on the **right**) correlations for the structure elucidation of product **4a**.

NMR spectroscopic characterization of compound **4a** was performed, with spectral data summarized in Table 1 and Table 2 and essential COSY and HMBC connectivities depicted in Figure 10. The biotransformation yielded product **4a** through glycosylation with a 4″-O-methylglucosyl unit, as demonstrated by the emergence of diagnostic signals for this substituent in both ^1^H and ^13^C NMR spectra (Supplementary Information: Figures S126 and S129). While ring A proton signals remained unchanged in the ^1^H NMR spectrum (compared to biotransformation substrate **4**), ring B exhibited altered signals with characteristic multiples at δ = 7.51 ppm (H-2′, H-6′) and δ = 7.12 ppm (H-3′, H-5′), displaying an AA′BB′ coupling pattern diagnostic of 4′-substitution in the 6-chloroflavanone B-ring (Supplementary Information: Figure S126). HMBC analysis substantiated this assignment through key correlations between H-2′/H-6′ (δ = 7.51 ppm) and H-3′/H-5′ (δ = 7.12 ppm) with the shifted C-4′ signal (δ = 159.0 ppm). Furthermore, the anomeric proton H-1″ (δ = 4.98 ppm) showed correlation with C-4′ (δ = 159.0 ppm), confirming the glycosidic linkage position (Supplementary Information: Figure S139).

### 2.8. Biotransformation of 6-Chloroflavanone (***4***) in Cultures of B. bassiana KCH J1.5

6-Chloroflavanone (**4**) was not efficiently and regioselectively biotransformed in cultures of B. bassiana KCH J1.5 strain. The obtained mixture of products was not sufficient for proper purification and structural characterization by NMR spectroscopy.

### 2.9. NMR Characterization and Stereochemical Considerations of Flavanone Glycosides

The ^1^H NMR spectrum of the synthesized flavanones glycosides (**2a**, and **3a**) revealed a characteristic doublet of doublets pattern for the anomeric proton with coupling constants of *J* = 7.7 Hz and *J* = 6.4 Hz in the compound **2a** and *J* = 7.7 Hz and *J* = 6.6 Hz in the compound **3a**, which does not correspond to conventional coupling with adjacent sugar proton but rather represents overlapping signals from two distinct diastereomeric products. The *J* = 7.8 Hz coupling constant is diagnostic for the *β*-anomeric configuration, reflecting the trans-diaxial arrangement between H-1 and H-2 in the sugar moiety (typical *β*-anomer *J*_1_,_2_∼7.8–8.5 Hz) while α-anomers would exhibit significantly smaller coupling constants (*J*_1_,_2_∼3.7 Hz) [34] due to the different dihedral angle relationship. Since the starting flavanones were obtained through chemical synthesis as a racemic mixture of (2R)- and (2S)-enantiomers, the subsequent glycosylation reaction yielded both (2R)-flavanone-*β*-glycoside and (2S)-flavanone *β*-glycoside diastereomers exclusively in the *β*-anomeric form. The observed coupling pattern arises from the superposition of two nearly identical but non-equivalent anomeric proton signals, where the different C-2 configuration in each flavanone enantiomer influences the overall molecular conformation and electronic environment around the glycosidic moiety, resulting in subtly different coupling constants for the same H-1-H-2 interaction in each diastereomer. This interpretation is further supported by ^13^C NMR analysis, which shows characteristic signal doubling for the stereochemically sensitive C-7 and C-3 carbons of the flavanone framework, confirming the presence of both diastereomeric glycosides in the reaction mixture. Accordingly, the glycosylation of racemic chloroflavanones yielded inseparable mixtures of two diastereomeric β-D-glucosides, corresponding to the (2R)- and (2S)-configured aglycones. The absolute configuration at C-2 of the aglycone was not determined in this study. The phenomenon demonstrates how remote chiral centers in the aglycone can influence NMR parameters of the glycosidic unit, providing valuable structural information for characterizing flavonoid glycoside diastereomers.

Biotransformation substrates, i.e., 2′-chloroflavanone (**1**), 3′-chloroflavanone (**2**), and 4′-chloroflavanone (**3**) were successfully biotransformed in cultures of fungi strain *B. bassiana* KCH J1.5 into glycosylated flavanones. Similar biotransformation attempts using *I. fumosorosea* KCH J2 resulted in the formation of complex mixtures containing small amounts of various products, which did not allow for their isolation with sufficient purity to determine their structures by NMR spectroscopy. However, the last biotransformation substrate, 6-chloroflavanone (**4**), was successfully transformed exclusively by strain *I. fumosorosea J2*, yielding a glycosylated product. The first three biotransformation products demonstrated identical regioselectivity of glycosylation occurring at the C-6 position, regardless of the chlorine substituent location (C-2′, C-3′, or C-4′ positions). In contrast, 6-chloroflavanone (**4**) possessed a chlorine substituent at the C-6 position, which may have sterically hindered the regioselective transformation at this site. Nevertheless, second used strain *I. fumosorosea* KCH J2 successfully catalyzed the biotransformation by attaching a 4″-*O*-methylglucopyranoside unit at the C-4′ position, demonstrating alternative regioselectivity when the preferred C-6 site was occupied.

### 2.10. Biotransformation Efficiency and Regioselectivity in Relation to Substrate Structure

For both fungal strains, the biotransformation of flavanones proved challenging, as evidenced by the low isolated product yields ranging from 5 to 8%. The progress of the biotransformations was monitored by TLC, and in most cases the reactions were continued until nearly complete substrate consumption. Nevertheless, the isolated yields remained low because only the major products were subjected to labor-intensive purification to achieve the highest possible purity for unambiguous structural assignment. This deliberate choice prioritized structural characterization over maximizing the overall isolated yield. And reflect the performance of wild-type fungal whole-cell systems with these particular compounds, where competing metabolic pathways and basal enzyme expression limit product accumulation. While yield enhancement through metabolic engineering could potentially improve conversion efficiency, such resource-intensive strategies require justification through demonstration of exceptional biological activity. The current yields provided sufficient material for preliminary biological evaluation and structure–activity relationship establishment, serving as a foundation for future optimization efforts, should superior lead compounds be identified. In comparison, biotransformation of analogous flavones by both strains, in our previous studies, yielded products in the range of 11 to 62%. Notably, 4′-chloroflavone was not biotransformed, while the remaining substrates were equally transformed by both strains: 2′-chloroflavone underwent glucosylation at the C-3′ position, 3′-chloroflavone at the C-4′ position—in both cases ortho to the chlorine substituent—and 6-chloroflavone at the C-4′ position, analogous to 6-chloroflavanone biotransformation [25]. The structural differences between these compound classes may account for the observed biotransformation efficiency disparities. Flavones possess a planar structure due to the C-2/C-3 double bond, whereas flavanones, as mentioned above, form two diastereoisomers, which may influence both the regioselectivity and efficiency of biotransformation processes.

This strain- and substrate-dependent regioselectivity pattern is further exemplified by biotransformation of methyl-substituted flavanones from our previous studies, demonstrating that both the nature (methyl vs. chloro) and position of substituents influence glycosylation site preference. For 2′-methylflavanone, *B*. *bassiana* KCH J1.5 generated products in which glycosylation followed prior hydroxylation at the C-6 position of the flavanone scaffold, whereas *I*. *fumosorosea* KCH J2 favored alternative modifications leading to glycosides at the C-3′ positions, thus illustrating clear strain-dependent regioselectivity [35]. In turn, 4′-methylflavanone revealed pronounced differences in both selectivity and yield: *B*. *bassiana* KCH J1.5 predominantly formed the 4′-methylene-*O*-β-D-(4″-*O*-methyl)-glucopyranoside in high isolated yield (52.0%), whereas *I*. *fumosorosea* KCH J2 produced the same product in lower yield (13.7%) along with minor hydroxylated derivatives [36]. For 6-methylflavanone, strain-dependent outcomes were also observed: *B*. *bassiana* KCH J1.5 generated several glycosides derived from hydroxylation at C-3′ or C-6, whereas *I*. *fumosorosea* KCH J2 preferentially produced 4′- and 4-*O*-glucosides [37]. These findings demonstrate the complex interplay between substrate structure, steric hindrance, and strain-specific enzyme repertoires in determining biotransformation regioselectivity and product diversity.

Fungal cultures of *Cunninghamella blakesleana* AS 3.970 glycosylated norkurarinone at C-7 (6′′-hydroxyl-2′-methoxyl-norkurarinone 7-*O*-*β*-D-glucoside) and C-4′ (6′′-hydroxylnorkurarinone 4′-*O*-*β*-D-glucoside) [38], while *Cunninghamella echinulata* AS 3.3400 glycosylated kurarinone at C-7 to yield kurarinone 7-*O*-*β*-D-glucoside [39]. Another fungal system, *Isaria fumosorosea* ACCC 37814 glycosylated naringenin at C-4′ and C-7 [24]. Glycosylation of flavanones in plant and bacterial systems exhibited similar regioselectivity patterns. *Eucalyptus perriniana* cultured cells efficiently transformed naringenin into: naringenin 7-*O*-*β*-D-glucopyranoside, naringenin 5,7-*O*-*β*-D-diglucopyranoside, naringenin 4′,7-*O*-*β*-D-diglucopyranoside, naringenin 7-*O*-[6-*O*-(*β*-D-glucopyranosyl)]-*β*-D-glucopyranoside, naringenin 7-*O*-[6-*O*-(α-L-rhamnopyranosyl)]-*β*-D-glucopyranoside, and 7-*O*-*β*-D-gentiobiosyl-4′-*O*-*β*-D-glucopyranosylnaringenin [40]. Marvalin and coworkers reported that bacterial strain *Streptomyces* sp. M52104 showed preferential glycosylation of naringenin at the C-7 position (25% yield for naringenin 7-*O*-*β*-D-glucuronide) compared to the C-4′ position (5% yield) [41]. Similarly, Shimoda and coworkers demonstrated that *Xanthomonas campestris* exhibited another regioselectivity pattern with hesperetin, producing glycosylated derivatives at the C-7 (15.0%), C-3′ (12.0%), and C-5 (10.0%) positions, with the highest yield observed for the C-7 glucopyranoside [42].

All of these findings indicate that regioselectivity in flavanone biotransformation can vary depending on the substrate structure and type of biocatalysts, with each organism enzymatic system displaying distinct positional preferences for glycosylation. Overall, our present studies reveal that entomopathogenic fungi strain *B. bassiana* KCH J1.5 can biotransform flavanones with a chlorine substituent at C-2′, C-3′, and C-6, while *I. fumosorosea* KCH J2 can glycosylated effectively only 6-chloroflavanone, demonstrating that the position of substitution significantly influences glycosylation capacity.

### 2.11. Pharmacokinetics and Drug-likeness Prediction of Compounds ***1***, ***1a***, ***2**,* ***2a***, ***3***, ***3a***, ***4***, ***4a***

The SwissADME platform (http://www.swissadme.ch, accessed on 28 July 2025), created and managed by the Molecular Modeling Group at the Swiss Institute of Bioinformatics (SIB) [43], was employed to evaluate the pharmacokinetic properties, water solubility, and drug-like characteristics of compounds **1**, **1a**, **2**, **2a**, **3**, **3a**, **4**, **4a**, along with flavanone (**5**) lacking chlorine substitution for comparative analysis. These computational assessments were performed on 28 July, with the respective prediction outputs documented in the Supplementary Materials: Figure S17 (**1**), Figure S35 (**1a**), Figure S52 (**2**), Figure S70 (**2a**), Figure S87 (**3**), Figure S105 (**3a**), Figure S122 (**4**), Figure S140 (**4a**), Figure S141 (**5**). The Brain Or IntestinaL EstimateD permeation methodology (BOILED-Egg) [44], which is a computational framework for evaluating molecular lipophilicity and polarity in small molecules, demonstrated favorable gastrointestinal absorption potential across all examined compounds. Aqueous solubility forecasts, derived through the Estimated SOLubility (ESOL) algorithm, showed marked distinctions between glycoside forms and their corresponding aglycone structures.

Glycosylated derivatives **1a**, **2a**, **3a**, and **4a** showed approximately an 11-fold increase in water solubility compared to their respective aglycone forms **1**, **2**, **3**, and **4** (0.1600 vs. 0.0147 mg/mL). These data illustrate how glycosylation can dramatically improve water solubility, potentially leading to altered absorption, distribution, metabolism, and excretion properties. Membrane transport properties were significantly altered in the biotransformation products (**1a**, **2a**, **3a**, and **4a**) compared to the corresponding aglycones (**1**, **2**, **3**, and **4**). The glycosylated forms lost their capacity for passive blood-brain barrier permeation but acquired recognition by P-glycoprotein efflux pumps. Computational simulations revealed distinct cytochrome P450 enzyme inhibition profiles for the tested compounds. Flavonoid aglycones **1**, **2**, **3**, and **4** showed inhibitory activity against CYP1A2 and CYP2C19, while compounds **2** and **3** additionally inhibited CYP2C9. Notably, compound **3** also demonstrated inhibitory potential against CYP3A4. In contrast, their glycosylated derivatives (**1a**, **2a**, **3a**, and **4a**) lost the ability to inhibit CYP1A2, CYP2C9, and CYP2C19, while compound **3a** retained inhibitory activity against CYP3A4. None of the tested compounds showed inhibitory activity against CYP2D6 (Table 5). Drug-likeness assessments using multiple established criteria (Lipinski, Ghose, Veber, Egan, and Muegge) through the SwissADME platform revealed that all compounds met the required standards without exception. The uniform Abbott bioavailability score of 0.55 across all tested molecules suggests a moderate probability (55%) of achieving adequate oral bioavailability (>10% in rats) or sufficient Caco-2 permeability. PAINS analysis showed no alerts for any compound, demonstrating low potential for interference in biochemical screening assays. Table 5 provides a detailed overview of these predictive outcomes.

Glycosylated compounds (**1a**, **2a**, **3a**, and **4a**) exhibited substantially increased water solubility compared to parent aglycones, enhanced predicted compatibility with oral delivery. However, reduced passive blood-brain barrier penetration and elevated susceptibility to efflux mechanisms, notably P-glycoprotein-mediated transport, represent important considerations for therapeutic targeting, especially in potential relevance to compounds targeting the central nervous system. Further investigation through systematic in vitro and in vivo bioavailability studies would therefore warrant further evaluation of pharmaceutical potential. These findings corroborate literature evidence that glycosylation modifications typically augment flavonoid solubility characteristics while altering membrane permeability profiles, thereby modulating overall pharmacokinetic behavior and systemic bioavailability [26]. Extensive research has established the profound impact of glycosylation on flavonoid pharmacological properties, particularly regarding solubility, permeability, and systemic availability. The attachment of sugar residues fundamentally alters molecular behavior by incorporating hydrophilic components that counteract the inherent lipophilicity of flavonoid backbones. Cellular uptake mechanisms of flavonoids undergo significant modification through glycoside formation. Unlike aglycones that rely predominantly on passive diffusion, glycosylated variants exploit specialized intestinal transport systems, including sodium-glucose co-transporter 1 (SGLT1) and glucose transporter 2 (GLUT2), as exemplified by quercetin-3-*O*-glucoside and cyanidin-3-*O*-glucoside uptake pathways [26,45]. Enhanced bioavailability emerges through multiple synergistic mechanisms. Glycosylated derivatives demonstrate superior chemical stability under physiological stress conditions, with e.g., rutin exhibiting greater resistance to metal-catalyzed degradation than quercetin [46]. Sugar substituents promote carrier-mediated transport across intestinal barriers, overcoming the diffusion-limited uptake characteristic of non-glycosylated compounds [47]. This differential transport is demonstrated by quercetin derivatives, where glucosides achieve more efficient absorption than rhamnosides due to selective recognition by β-glucosidase in the small intestine [46]. Additionally, glycosylation provides partial protection against Phase II conjugation reactions, such as glucuronidation, thereby maintaining higher concentrations of pharmacologically active species in circulation [48]. From this perspective, glycosylation serves as a crucial molecular modification strategy for enhancing flavonoid bioavailability and therapeutic efficacy by improving their pharmacokinetic properties [26,48,49].

### 2.12. Antimicrobial Activity of Compounds ***1***, ***1a***, ***2***, ***2a***, ***3***, ***3a***, ***4***, ***4a***

The antimicrobial properties of compounds **1**, **1a**, **2**, **2a**, **3**, **3a**, **4**, **4a**, and **5** were examined by measuring optical density using the turbidimetric method with the Synergy H1 microplate reader (BioTek Instruments, Winooski, VT, USA) to determine the effects of chlorine incorporation, substitution pattern, and 4′-O-methylglucopyranose attachment on flavanone antimicrobial activity. Testing was conducted against three Gram-positive bacteria strains: *Enterococcus faecalis* ATCC 19433, *Staphylococcus aureus* ATCC 29213, *Lactobacillus acidophilus* ATCC 4356 (lactic acid bacteria), one Gram-negative bacterium *Escherichia coli* ATCC 25922, and the fungal strain *Candida albicans* ATCC 10231. The findings are applicable exclusively to the specific ATCC-designated strains tested and cannot be generalized to the broader species. Growth measurements for control cultures (strain only) and experimental samples (with flavonoids or antibiotic standards), reported as optical density increases (ΔOD), are detailed in Table 6. These data should be interpreted as screening-level growth-inhibition trends rather than definitive potency metrics (MIC).

**Figure 11:**
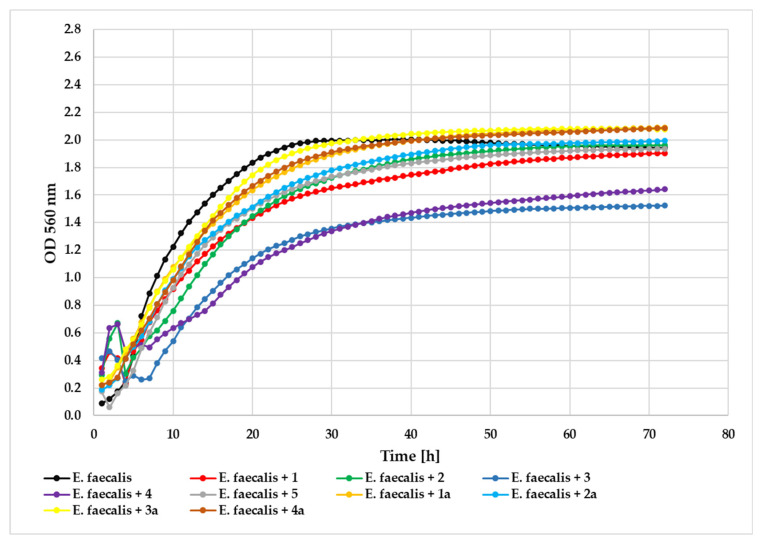
The effect of the action of compounds **1**, **1a**, **2**, **2a**, **3**, **3a**, **4**, **4a**, and **5** in concentration 0.05% on the growth of *E. faecalis* ATCC 19433.

Biological assays were conducted on the obtained flavonoids to assess how structural modifications in the flavanone scaffold—specifically the addition of a chlorine substituent and a 4′-*O*-methylglucosyl group—influenced antimicrobial efficacy. Among the tested compounds, flavonoid aglycone **3** demonstrated the most pronounced antibacterial activity against the *E. faecalis* strain (Gram-positive) at 0.05% concentration with a ΔOD value of 1.11 compared to the control (bacterial strain without flavonoid treatment with ΔOD = 1.86). The remaining aglycones exhibited weaker inhibitory effects: compound **4** (ΔOD = 1.33), compound **1** (ΔOD = 1.56), compound **2** (ΔOD = 1.67), and flavanone without substituents **5** (ΔOD = 1.76). Notably, the corresponding glycoside derivatives (**1a**, **2a**, **3a**, **4a**) showed no measurable inhibition of bacterial growth. The comparative growth profiles of *E. faecalis* in the presence of each test compound at 0.05% concentration are illustrated in Figure 11.

**Figure 12:**
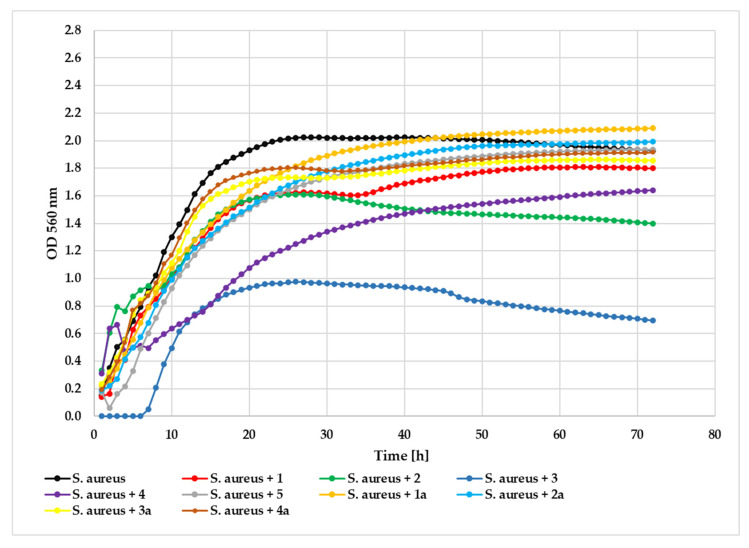
The effect of the action of compounds **1**, **1a**, **2**, **2a**, **3**, **3a**, **4**, **4a**, and **5** in concentration 0.05% on the growth of *S. aureus* ATCC 29213.

Antimicrobial evaluation against the *S. aureus* strain revealed that compound **3** exhibited the strongest inhibitory activity (ΔOD = 0.70) at 0.05% concentration, compared to the untreated control (ΔOD = 1.77). Compound **4** demonstrated moderate antibacterial effects (ΔOD = 1.01), followed by compound **2** (ΔOD = 1.10) and compound **5** (ΔOD = 1.17). Compound **1** showed minimal inhibition (ΔOD = 1.66). The glycoside derivatives consistently displayed reduced activity: **1a** (ΔOD = 1.97), **2a** (ΔOD = 1.88), **4a** (ΔOD = 1.72), and **3a** (ΔOD = 1.62), with values approaching those of the untreated control. The growth of the tested *S. aureus* strain in the presence of the tested compounds at a concentration 0.05% is shown in Figure 12.

**Figure 13:**
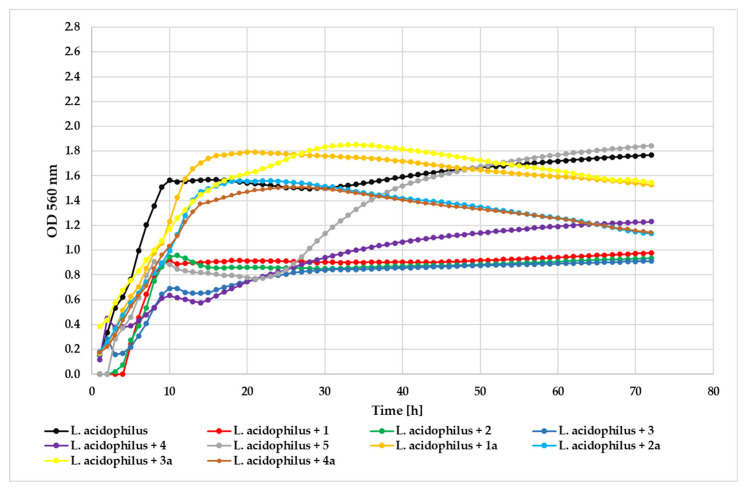
The effect of the action of compounds **1**, **1a**, **2**, **2a**, **3**, **3a**, **4**, **4a**, and **5** in concentration 0.05% on the growth of *L. acidophilus* ATCC 4356.

Testing against *L. acidophilus* demonstrated that compound **3** maintained superior antimicrobial efficacy (ΔOD = 0.76) relative to the control (ΔOD = 1.61). Compounds **2** (ΔOD = 0.94) and **1** (ΔOD = 0.98) exhibited comparable moderate inhibitory effects, while compounds **5** (ΔOD = 1.06) and **4** (ΔOD = 1.11) showed weaker activity. Among the glycosides, **2a** (ΔOD = 0.95) and **4a** (ΔOD = 0.97) displayed modest inhibition, whereas **3a** (ΔOD = 1.16) and **1a** (ΔOD = 1.36) were less effective. The growth of the *L. acidophilus* strain in the presence of the tested compounds at a concentration 0.05% is shown in Figure 13. The observed inhibitory activity against this beneficial strain suggests limited selectivity between pathogenic and commensal bacteria. Future investigations should explore whether the glycosylated derivatives might exhibit prebiotic properties that could promote the growth of beneficial probiotic strains, potentially offsetting their mild inhibitory effects.

**Figure 14:**
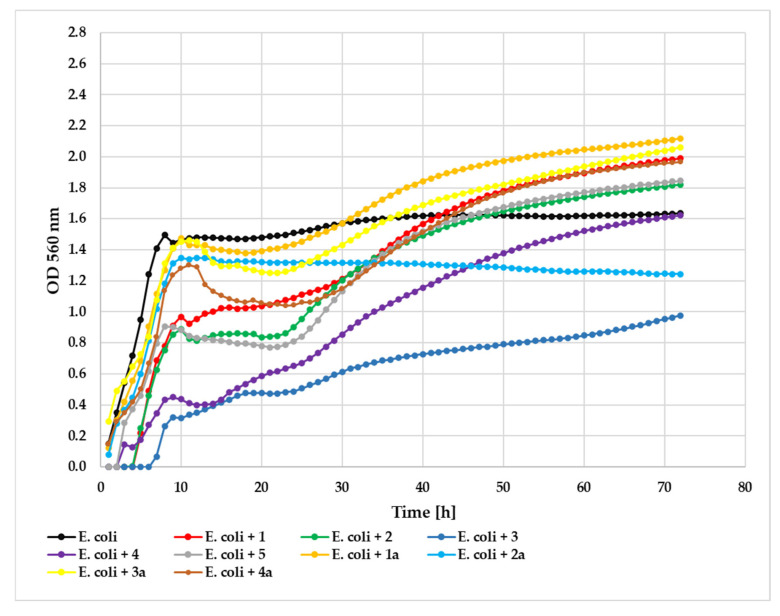
The effect of the action of compounds **1**, **1a**, **2**, **2a**, **3**, **3a**, **4, 4a**, and **5** in concentration 0.05% on the growth of *E. coli* ATCC 25922.

Evaluation against the Gram-negative *E. coli* strain showed compound **3** as the only effective inhibitor (ΔOD = 0.98) compared to the control (ΔOD = 1.48). Remarkably, most tested compounds actually promoted bacterial growth, with ΔOD values exceeding the control: compounds **1** (ΔOD = 1.99), **1a** (ΔOD = 2.00), **2** (ΔOD = 1.82), **3a** (ΔOD = 1.77), **4** (ΔOD = 1.62), **4a** (ΔOD = 1.82), and **5** (ΔOD = 1.84). Only glycoside **2a** demonstrated modest inhibitory activity (ΔOD = 1.17). This growth-promoting effect suggests that certain flavonoid structures may serve as growth stimulants for this Gram-negative strain, highlighting the importance of structural modifications in determining antimicrobial versus growth-promoting properties. The growth of the *E. coli* strain against the tested compounds at a concentration 0.05% is shown in Figure 14.

**Figure 15:**
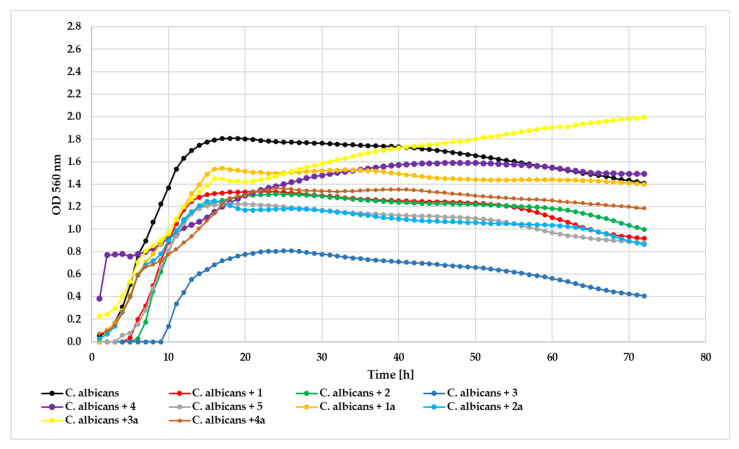
The effect of the action of compounds **1**, **1a**, **2**, **2a**, **3**, **3a**, **4**, **4a**, and **5** in concentration 0.05% on the growth of *C. albicans* ATCC 10231.

Antifungal assessment against *C. albicans* revealed compound **3** as the most potent inhibitor (ΔOD = 0.41) relative to the control (ΔOD = 1.35). Glycoside **2a** (ΔOD = 0.84) and compound **5** (ΔOD = 0.88) demonstrated notable antifungal activity, followed by compound **1** (ΔOD = 0.92) and compound **2** (ΔOD = 1.00). Compounds **4** (ΔOD = 1.11) and **4a** (ΔOD = 1.12) showed moderate inhibitory effects, while **1a** (ΔOD = 1.40) and **3a** (ΔOD = 1.77) exhibited progressively weaker antifungal activity against this fungal strain.The effect of the action of the tested compounds on *Candida albicans* at concentration 0.05% is shown in Figure 15.

The antimicrobial evaluation of chlorinated flavanone aglycones (**1**, **2**, **3**, **4**) and their glycoside derivatives (**1a**, **2a**, **3a**, **4a**), in comparison with the non-chlorinated flavanone **5**, revealed that chlorination markedly enhanced antimicrobial activity, with the extent of this effect depending on the position of chlorine substitution. In general, aglycones exhibited stronger inhibitory effects than their glycosylated counterparts, as reflected by consistently lower ΔOD values. Among all tested compounds, aglycone **3**, bearing chlorine in a specific substitution pattern at C-4′, demonstrated the broadest and most potent spectrum of activity, showing the lowest ΔOD readings across nearly all bacterial and yeast strains, particularly against *Staphylococcus aureus*, *Lactobacillus acidophilus*, and *Candida albicans*. In contrast, compound **5** displayed notably weaker inhibition, underscoring the contribution of chlorine to antimicrobial potency. Glycosylation reduced activity in nearly all cases, with ΔOD values approaching control levels for several derivatives. Gram-positive bacteria (*S. aureus*, *Enterococcus faecalis*, *L. acidophilus*) were generally more susceptible than Gram-negative *Escherichia coli* and the yeast *C. albicans*, although compound **3** retained significant activity across all taxa. Compared with the previously studied flavone series [25], the present flavanones showed overall weaker and occasionally paradoxical growth effects under the screening conditions, consistent with structure-dependent differences. Considering that the lactic acid bacterium *L. acidophilus* ATCC 4356 is regarded as a strain beneficial to humans, it is noteworthy that in the presence of some tested compounds, including glycosides **1a** and **3a** as well as the non-chlorinated flavanone **5**, relatively higher ΔOD values were observed for this microorganism than most other tested substances, indicating weaker growth inhibition. These results suggest that chlorinated flavanones, particularly with substitution patterns as in compound **3**, may display relatively stronger antimicrobial effects than their non-chlorinated analogs, and that both the presence and position of chlorine can influence biological activity. The studies showed the influence of chlorine atom substitution in the absence of other substituents, thereby allowing for the isolated assessment of chlorine’s impact on antibacterial activity. Additional research is needed to clarify the mechanistic pathways of these compounds, assess the specific contributions of chlorine and glucosyl substituents, and validate findings through in vivo studies. Current literature provides conflicting evidence regarding glycosylation’s effect on flavonoid bioactivity, with notable discrepancies between in vitro and in vivo models. Our results confirm this pattern across bacterial and yeast systems, highlighting chloroflavanones antimicrobial potential. Interestingly, some compounds appeared to stimulate the growth of *E*. *coli*, which may reflect the biphasic nature of flavonoid–microbe interactions, where certain derivatives at sub-inhibitory concentrations can transiently support microbial metabolism rather than suppress it.

When we compared our studies with the results of Fowler and coworkers, a similar trend was observed: chlorine substitution was associated with higher antimicrobial activity compared with non-halogenated analogs. The researchers identified 4-chloroflavanone as the most active synthetic derivative in their panel, with MIC values against *Bacillus subtilis* and certain fungi approaching those of conventional antibiotics, particularly when combined with efflux pump inhibitors in *Escherichia coli*. In both cases, the data indicate that chlorine substitution can enhance intrinsic antimicrobial properties, though the overall effect depends strongly on substitution position and the microbial species tested [7]. Researchers Kamboj and coworkers conducted antimicrobial screening studies which revealed that flavanones with chloro- and hydroxyl- substituents (6-chloro-3′-hydroxyflavanone and 6-chloro-4′-hydroxyflavanone) exhibited the highest antibacterial activity against Gram-positive bacteria, with inhibition zones of 21.6–23.6 mm against *S. aureus* and 20.6–22.6 mm against *B. subtilis*, and the lowest MIC values of 32 μg/mL. Notably, chloro-substituted flavanones showed variable activity depending on additional substituents, while 6-chloro- derivatives with hydroxyl groups demonstrated excellent activity, those with methyl or methoxy groups (6-chloro-4′-methylflavanone, 6-chloro-4′-methoxyflavanone, 6-chloro-3′,4′,5′-trimethoxyflavanone) showed moderate activity, suggesting that the combination of chlorine with hydroxyl groups enhances antimicrobial potency. All tested compounds were inactive against Gram-negative bacteria [19]. Researchers Zhang and coworkers conducted synthesis and biological evaluation studies of 5,7-dihydroxyflavanone derivatives, revealing that halogenated compounds demonstrated superior antimicrobial activity compared to their natural analogs, with the 5,7-dihydroxy-3′,4′-dichloroflavanone showing the most potent activity against Gram-positive bacteria strains and also demonstrating activity against all tested Gram-negative bacteria strains. The study found that while fluorinated derivatives showed variable activity depending on the number of fluorine substituents, compounds with hydroxyl or methoxy groups were generally inactive, and importantly, all halogenated derivatives exhibited low cytotoxicity against HepG2 cells at therapeutic concentrations, with the dichlorinated compound even promoting cell proliferation [6].

The systematic review examined the antibacterial activity of flavanones (naringin, naringenin, hesperidin, and taxifolin) against oral pathogens by analyzing 11 studies and found that flavanones demonstrate significant antibacterial effects, particularly against Gram-positive bacteria like *Enterococcus faecalis*, with minimum inhibitory concentrations ranging from 100–256 μg/mL for Gram-positive bacteria and 1000–62,500 μg/mL for Gram-negative bacteria. The study revealed that Gram-negative bacteria associated with periodontal disease require much higher concentrations of flavanones to achieve antibacterial effects compared to Gram-positive bacteria [50]. However, another study investigated the antimicrobial mechanisms of three flavonoids (quercetin, naringenin and catechin) against *Staphylococcus aureus* and their protective effects against bacterial hemolysis. The researchers found that quercetin was the most effective compound, with the lowest minimum inhibitory concentration (100 μM) compared to naringenin (200 μM) and catechin (150 μM). The key antimicrobial mechanism involved membrane disruption, where quercetin significantly increased bacterial cell diameter and enhanced membrane fluidity at both inner hydrophobic and surface regions of bacterial membranes. However the study revealed that quercetin’s superior activity was attributed to its lipophilicity, planar molecular structure with a double bond between carbons C2 and C3, and optimal membrane penetration properties. Unlike naringenin and catechin, quercetin could effectively incorporate into lipid bilayers and alter membrane dynamics, leading to bacterial cell dysfunction while simultaneously protecting host cells from bacterial toxins through membrane stabilization [51]. Our previous investigations of analogous flavones demonstrated superior antimicrobial efficacy against the same microbial strains. Notably, the flavonoid aglycones 4′-chloroflavone and 6-chloroflavone exhibited the highest antimicrobial activity. These findings suggest that chlorine substitution at specific positions significantly enhances antimicrobial potency, regardless of whether the compound belongs to the flavone or flavanone subclass, indicating a consistent structure–activity relationship across these flavonoid categories [25].

## 3. Materials and Methods

### 3.1. Substrates

The biotransformation substrates 2′-chloroflavanone (**1**), 3′-chloroflavanone (**2**), 4′-chloroflavanone (**3**), and 6-chloroflavanone (**4**) were synthesized from the corresponding chlorinated 2′-hydroxychalcones obtained in our previous studies [32,33]. The synthetic pathways are illustrated in Figure 1 within Section 2 (Results and Discussion). The synthetic approach involved sodium acetate-mediated cyclization of chlorinated 2′-hydroxychalcones under reflux for 48 h [52], achieving the following synthetic yields: 69.4% for compound **1**, 76.3% for compound **2**, 71.3% for compound **3**, and 60.6% for compound **4** [32,33,53]. All chloroflavanone substrates were obtained as racemic mixtures of (2R)- and (2S)-enantiomers. Comprehensive characterization data for the synthesized chloroflavanones (**1**–**4**) are compiled in Table 1, Table 2, Table 3 and Table 4 (NMR spectral data), Supplementary Materials (NMR spectra and HPLC chromatograms) and below, where data presented include physical properties, melting point temperatures, the optical rotation [α]^20^_D_, and concentration c (M) and chromatographic behavior (retention times t_R_ in minutes).

2′-Chloroflavanone (**1**): light-yellow crystals, mp = 75–77 °C, t_R_ = 17.67 (Supplementary Materials: Figure S1), ^1^H-NMR, see Table 1, ^13^C-NMR, see Table 2 and also Supplementary Materials: Figures S2–S16.

3′-Chloroflavanone (**2**): light-yellow crystals, mp = 77–79 °C, t_R_ = 17.51 (Supplementary Materials: Figure S36), ^1^H-NMR, see Table 1, ^13^C-NMR, see Table 2 and also Supplementary Materials: Figures S37–S51.

4′-Chloroflavanone (**3**): light-yellow crystals, mp = 92–94 °C, t_R_ = 17.50 (Supplementary Materials: Figure S71), ^1^H-NMR, see Table 3, ^13^C-NMR, see Table 4 and also Supplementary Materials: Figures S72–S86.

6-Chloroflavanone (**4**): light-yellow crystals, mp = 88–90 °C, t_R_ = 17.71 (Supplementary Materials: Figure S106), ^1^H-NMR, see Table 3, ^13^C-NMR, see Table 4 and also Supplementary Materials: Figures S107–S139.

### 3.2. Microorganisms Used for Biotransformation

The biotransformation studies of chloroflavanones **1**–**4** were conducted using two entomopathogenic fungal species belonging to the *Cordycipitaceae* family: *Isaria fumosorosea* strain KCH J2 and *Beauveria bassiana* strain KCH J1.5. Both fungal isolates originated from the microorganism repository housed at the Faculty of Biotechnology and Food Microbiology, Department of Food Chemistry and Biocatalysis at Wrocław University of Environmental and Life Sciences, Poland. The protocols for fungal isolation, growth conditions, and molecular taxonomic identification of these entomopathogenic species have been comprehensively described in our previous research contributions [54,55].

### 3.3. Analysis

Biotransformation progression was tracked through chromatographic techniques, employing thin-layer chromatography (TLC) and high-performance liquid chromatography (HPLC) for substrate conversion assessment.

TLC analyses were executed on Silica gel 60/Kieselguhr F254 plates (0.2 mm layer thickness; Merck, Darmstadt, Germany). The chromatographic development utilized a chloroform:methanol solvent system (9:1 *v*/*v*), with chloroform and methanol sourced from Chempur (Piekary Śląskie, Poland). Compound detection was accomplished through UV illumination at 254 nm and 365 nm wavelengths without supplementary visualization reagents [33].

HPLC analyses were performed using a Dionex Ultimate 3000 chromatographic system (Thermo Fisher Scientific, Waltham, MA, USA) coupled with a DAD-3000 diode array detection unit. Chromatographic resolution was accomplished on an ODS 2 analytical column (4.6 × 250 mm, Waters, Milford, MA, USA) paired with a matching guard column. The binary mobile phase comprised: solvent A (0.1% formic acid in ultrapure water *v*/*v* (Honeywall, Charlotte, NC, USA; water from Supelco, Darmstadt, Germany) and solvent B (0.1% formic acid in acetonitrile *v*/*v* (Supelco, Darmstadt, Germany). The gradient elution profile proceeded as follows: 0 min—32.5% B in A, 4 min—40% B in A, 8 min—40% B in A, 10 min—45% B in A, 15 min—95% B in A, 18 min—95% B in A, 19 min—32.5% B in A, 23 min—32.5% B in A. Sample preparation involved dissolution in acetonitrile at 1 mg/mL concentration with 10 µL injection volumes. The mobile phase flow rate was set to 1 mL/min, with detection wavelengths of 254 nm [33].

Preparative isolation of biotransformation products was accomplished through preparative TLC using silica gel plates (500 µm and 1000 µm thickness; Analtech, Gehrden, Germany) developed with chloroform:methanol (9:1 *v*/*v*) mobile phase. Target compounds were recovered through triple extraction of scraped silica zones using 20 mL ethyl acetate each time sourced form Stanlab (Lublin, Poland), with subsequent solvent evaporation under vacuum using a rotary evaporator (Heidolph, Schwabach, Germany) [33].

Structural elucidation was performed using a Bruker DRX Avance 600 MHz NMR spectrometer through comprehensive spectroscopic analysis (^1^H, ^13^C, COSY, HMQC (Heteronuclear Multiple Quantum Coherence), and HMBC experiments) with deuterated acetone as the dissolution medium. Optical rotation measurements were obtained using an ABL&E-JASCO P-2000-Na digital polarimeter (Kraków, Poland).

### 3.4. Screening Procedure

Initial screening studies were performed to assess the biotransformation capacity and determine the optimal reaction duration for substrates **1**–**4** prior to conducting semipreparative-scale investigations. The experimental procedures utilized a modified Sabouraud medium (containing 10 g aminobac and 30 g glucose sourced from BTL, Warsaw, Poland, per 1 L of distilled water). The biotransformation protocol involved a two-phase cultivation approach. In the initial phase, fungal isolates were transferred from potato dextrose agar slants to liquid modified Sabouraud medium (100 mL) contained in 300 mL Erlenmeyer flasks. These cultures underwent incubation for 72 h at 25 °C under agitation at 140 rpm (DHN shaker, Warsaw, Poland). In the subsequent phase, 1 mL aliquots from these starter cultures were inoculated into fresh medium (100 mL) in new 300 mL Erlenmeyer flasks and maintained under equivalent conditions. Biotransformation reactions were commenced by introducing 10 mg of substrates **1**–**4** (solubilized in 0.5 mL dimethyl sulfoxide (Chempur, Piekary Śląskie, Poland) to cultures of *I. fumosorosea* KCH J2 or *B. bassiana* KCH J1.5 at the terminal phase of logarithmic growth, resulting in a final substrate concentration of 0.39 mM. Sample collection occurred at 3, 6, and 8 days following substrate introduction. Product isolation was achieved through ethyl acetate extraction (30 mL), followed by drying over anhydrous magnesium sulfate (Chempur, Piekary Śląskie, Poland), and concentration via rotary evaporation at 55 °C. Experimental procedures were concluded after 8 days upon achieving complete substrate depletion or when reaction progression ceased, as determined through TLC and HPLC monitoring. Control experiments encompassed substrate stability assessments under biotransformation conditions and microbial cultivation in the absence of substrate addition [33]. The screening was intended solely to guide strain–substrate selection and incubation time for the semipreparative runs; detailed, reportable outcomes are presented in the *Results and Discussion*.

### 3.5. The Semipreparative Biotransformation

Scaled-up biotransformation reactions were conducted in 2 L flasks containing 500 mL of modified Sabouraud medium. This expanded scale facilitated the recovery of adequate product quantities for comprehensive structural analysis via NMR spectroscopy. The procedure initiated with the inoculation of 5 mL pre-cultivation cultures of *I. fumosorosea* KCH J2 or *B. bassiana* KCH J1.5 into respective flasks, subsequently incubated for 72 h under parameters identical to those employed in the screening procedure. Individual substrates (**1**–**4**) were solubilized in 2.0 mL dimethyl sulfoxide and introduced separately to the fungal cultures to achieve a final concentration of 0.39 mM, maintaining consistency with screening experimental conditions. While the original protocol anticipated 8-day incubation periods based on preliminary screening data, reactions were monitored throughout this duration and discontinued upon verification of complete substrate depletion. Product recovery was accomplished through sequential extraction of reaction mixtures utilizing three 300 mL portions of ethyl acetate. The combined organic extracts were subjected to brief dehydration over anhydrous magnesium sulfate (5 min), followed by filtration and solvent removal under reduced pressure. Compound purification was achieved using preparative thin-layer chromatography plates according to established methodology. Product zones were visualized under ultraviolet illumination, excised, and subjected to triple extraction with 20 mL ethyl acetate aliquots. The isolated compounds were subjected to spectroscopic characterization for structural determination. Reaction yields were determined based on the recovered masses of purified products. Table 7 below presents a summary of biotransformation yields and principal reaction parameters [33].

### 3.6. Fungal Biotransformation Products

Comprehensive characterization data for fungal biotransformation products (**1a**, **2a**, **3a**, **4a**) are compiled in Table 1, Table 2, Table 3 and Table 4 (NMR spectral data), Supplementary Materials (NMR spectra and HPLC chromatograms) and below, where data presented include physical properties, melting point temperatures, the optical rotation [α]^20^_D_, and concentration c (M) and chromatographic behavior (retention times t_R_ in minutes). The reported specific rotations [α]^20^_D_ refer to the bulk diastereomeric mixtures; values should not be interpreted as assignments of absolute configuration at C-2.

2′-Chloroflavanone 6-*O*-*β*-D-(4″-*O*-methyl)-glucopyranoside (**1a**): white crystals, mp = 99–101 °C, t_R_ = 8.28 (Supplementary Materials: Figure S18), [α]^20^_D_ = −34.79 (c = 0.58, acetone), ^1^H-NMR, see Table 1, ^13^C-NMR, see Table 2 and also Supplementary Materials: Figures S19–S34.

3′-Chloroflavanone 6-*O*-*β*-D-(4″-*O*-methyl)-glucopyranoside (**2a**): white crystals, mp = 112–114 °C, t_R_ = 9.12 (Supplementary Materials: Figure S53), [α]^20^_D_ = −45.34 (c = 0.45, acetone), ^1^H-NMR, see Table 1, ^13^C-NMR, see Table 2 and also Supplementary Materials: Figures S54–S69.

4′-Chloroflavanone 6-*O*-*β*-D-(4″-*O*-methyl)-glucopyranoside (**3a**): white crystals, mp = 115–117 °C, t_R_ = 9.34 (Supplementary Materials: Figure S88), [α]^20^_D_ = −42.75 (c = 0.51, acetone), ^1^H-NMR, see Table 3, ^13^C-NMR, see Table 4 and also Supplementary Materials: Figures S89–S104.

6-Chloroflavanone 4′-*O*-*β*-D-(4″-*O*-methyl)-glucopyranoside (**4a**): white crystals, mp = 106–108 °C, t_R_ = 8.88 (Supplementary Materials: Figure S123), [α]^20^_D_ = −26.77 (c = 0.68, acetone), ^1^H-NMR, see Table 3, ^13^C-NMR, see Table 4 and also Supplementary Materials: Figures S124–S139.

### 3.7. Pharmacokinetics, Drug Nature, Biological Activity Predictions

SwissADME (accessed 28 July 2025) was used to compute in silico descriptors and prediction panels for flavonoids **1**, **1a**, **2**, **2a**, **3**, **3a**, **4**, **4a**, and **5.** Molecular structures were generated using ACD Chemsketch 2021.2.0 software and converted to .mol file format for SwissADME input processing. Computational prediction outcomes are presented in the Supplementary Materials: Figure S17 (**1**), Figure S35 (**1a**), Figure S52 (**2**), Figure S70 (**2a**), Figure S87 (**3**), Figure S105 (3**a**), Figure S122 (**4**), Figure S140 (**4a**), Figure S141 (**5**).

### 3.8. Antimicrobial Activity Assays

Antimicrobial efficacy assessment of compounds **1, 1a, 2, 2a, 3, 3a, 4, 4a**, (synthesized as described above) and compound **5** (obtained from Sigma-Aldrich, Saint Louis, MO, USA) was performed utilizing a BioTek microplate reader (Winooski, VT, USA) [32,56]. Antimicrobial effects were assessed using a turbidimetric growth-inhibition readout (ΔOD), i.e., the difference between endpoint and baseline optical density, with growth and solvent controls included. The ΔOD method was deliberately chosen to eliminate potential artifacts arising from differential diffusion rates that would inevitably occur in agar-based MIC assays ((broth microdilution per CLSI/EUCAST). Aglycones, being more lipophilic, would demonstrate superior agar penetration compared to their glycosidic forms, potentially leading to systematically underestimated MIC values for glycosides that would reflect diffusion limitations rather than true antimicrobial potency. This single-concentration assay was selected as a screening tool to compare structure–activity trends across multiple compounds and organisms under uniform conditions, in line with widely used preliminary antimicrobial screening practices. Moreover, standardized MIC testing was not pursued here due to limited available amounts of purified products; instead, ΔOD enabled time-resolved, resource-efficient ranking of compounds. The microbial test panel included bacterial isolates: *Enterococcus faecalis* ATCC 19433, *Staphylococcus aureus* ATCC 29213, *Lactobacillus acidophilus* ATCC 4356, *Escherichia coli* ATCC 25922, and the yeast isolate *Candida albicans* ATCC 10231. Microorganisms were pre-cultivated for 48 h in Mueller-Hinton broth obtained from Merck (Darmstadt, Germany) before experimental use. Bioassays were executed in 96-well microtiter plates with a final volume of 300 μL per well, consisting of 240 μL growth medium, 50 μL standardized microbial inoculum, and 10 μL flavonoid test solution (0.05% in dimethyl sulfoxide). The test concentration (0.05%) was chosen empirically in preliminary runs to avoid complete growth arrest while preserving dynamic range for trend discrimination; solvent controls at the same level confirmed no solvent-driven effects. Growth kinetics were monitored at 37 °C over 72 h with optical density measurements at 560 nm recorded at 60-min intervals. All assays were performed in triplicate under continuous microplate agitation. Reference antimicrobial agents oxytetracycline and cycloheximide, both sourced from Sigma-Aldrich (Saint Louis, MO, USA), served as positive controls. Data analysis was conducted using Microsoft Excel 365 software. Growth kinetic profiles were constructed from mean absorbance values, and antimicrobial efficacy was quantified as the increase of the optical density (**Δ**OD) compared to control cultures containing dimethyl sulfoxide alone.

## 4. Conclusions

Introducing a chlorine atom and glucosyl moiety leads to significant changes in flavonoid biological activity and bioavailability. Our studies revealed the capacity of enzymatic systems from entomopathogenic fungi strains *Isaria fumosorosea* KCH J2 and *Beauveria bassiana* KCH J1.5 to produce 4-*O*-methylglucosides from synthetic chloroflavanones containing chlorine atoms at different positions on the flavonoid backbone (2′, 3′, 4′, and 6). Both fungal strains in this case exhibited distinct differences in their ability to biotransform these xenobiotics. Strain *B. bassiana* KCH J1.5 successfully biotransformed substrates with chlorine atom at positions 2′, 3′, and 4′, whereas strain *I. fumosorosea* KCH J2 regioselectively biotransformation only 6-chloroflavanone. All other biotransformation reactions resulted in complex mixtures of products.

2′-Chloroflavanone underwent biotransformation to yield 2′-chloroflavanone 6-*O*-*β*-D-(4″-*O*-methyl)-glucopyranoside (**1a**), while 3′-chloroflavanone was converted to 3′-chloroflavanone 6-*O*-*β*-D-(4″-*O*-methyl)-glucopyranoside (**2a**). 4′-Chloroflavanone was biotransformed to the analogous product 4′-chloroflavone 6-*O*-*β*-D-(4″-*O*-methyl)-glucopyranoside (**3a**). The final biotransformation substrate, 6-chloroflavanone, was successfully transformed into 6-chloroflavanone 4′-*O*-*β*-D-(4″-*O*-methyl)-glucopyranoside (**4a**). Unfortunately, all biotransformations proceeded with low efficiency, with isolated product yields ranging from 5 to 8%. Based on comprehensive literature analysis, these biotransformation products represent previously unreported compounds, which significantly expand the current knowledge base regarding chlorinated flavonoids and their enzymatic glycosylation pathways. The results show that chloroflavanones are not as easily glycosylated by the used entomopathogenic strains as their flavone analogs.

In parallel with experimental work, computational structure–activity relationship studies were conducted to predict the biological activities of the synthesized compounds and guide future screening efforts. However, comprehensive in vitro and in vivo studies remain essential to fully characterize their biological activities, pharmacokinetic profiles, and molecular mechanisms of action. Our antimicrobial screening demonstrated that chlorine substitution in the flavanone backbone enhances antimicrobial efficacy against the tested microorganisms, with activity patterns dependent on the positional placement of the chlorine atom. Further mechanistic studies are required to elucidate the precise modes of antimicrobial action. Our results corroborate previous observations on the antimicrobial potential of halogenated flavonoids. By examining compounds devoid of additional substituents, we were able to assess the isolated effects of chlorine atoms, demonstrating enhanced antimicrobial activity in both bacterial and yeast models.

Future mechanistic elucidation of the antimicrobial activity represents a critical research priority, requiring detailed investigation of molecular pathways and comprehensive characterization including cytotoxicity profiling, pharmacokinetic evaluation, and in vivo efficacy assessment. Expanding the biotransformation approach through screening additional entomopathogenic fungal strains may yield structurally diverse derivatives with enhanced biological properties. Integration of structure-based approaches with bioactivity data could facilitate further rational development of optimized derivatives with improved potency and selectivity for therapeutic applications.

## Figures and Tables

**Table 1:** ^1^H-NMR chemical shifts δ (ppm) and coupling constants *J* (Hz) of 2′-chloroflavanone (**1**), 3′-chloroflavanone (**2**) and their biotransformation products **1a** and **2a** in Acetone-d6, 600 MHz (Supplementary Information: Figures S2, S3, S19–S21, S37, S38 and S54–S56).

Proton	Compound			
	1	1a	2	2a
H-2	5.94 (dd) *J =* 13.4 *J =* 2.8	5.89 (dd) *J =* 13.5 *J =* 2.7	5.69 (dd) *J =* 13.1 *J =* 2.9	5.64 (m)
H-3_ax_	3.10 (dd) *J =* 16.8 *J =* 13.4	3.07 (dd) *J =* 16.8 *J =* 13.5	3.17 (dd) *J =* 16.8 *J =* 13.1	3.13 (dd) *J =* 16.8 *J =* 13.1
H-3_eq_	2.92 (dd) *J =* 16.8 *J =* 2.8	2.91 (dd) *J =* 16.8 *J =* 2.8	2.91 (dd) *J =* 16.8 *J =* 3.0	2.91 (dt) *J =* 16.8 *J =* 2.9
H-5	7.88 (dd) *J =* 7.8 *J =* 1.7	7.50 (m)	7.85 (dd) *J =* 8.1 *J =* 1.8	7.47 (t) *J =* 2.9
H-6	7.13 (m)	-	7.11 (m)	-
H-7	7.61 (ddd) *J =* 8.4 *J =* 7.3 *J =* 1.8	7.36 (dd) *J =* 9.0 *J =* 3.1	7.60 (ddd) *J* = 8.5 *J* = 7.4 *J* = 1.8	7.35 (dd) *J* = 9.0 *J* = 3.1
H-8	7.13 (m)	7.06 (dd) *J* = 9.0 *J* = 1.7	7.11 (m)	7.07 (dd) *J* = 9.0 *J* = 1.2
H-2′	-	-	7.67 (t) *J* = 1.8	7.65 (m)
H-3′	7.51 (m)	7.50 (m)	-	-
H-4′	7.44 (m)	7.44 (td) *J* = 7.7 *J* = 1.7	7.44 (m)	7.43 (m)
H-5′	7.51 (m)	7.50 (m)	7.49 (t) *J =* 7.7	7.49 (d) *J =* 7.9
H-6′	7.85 (dd) *J =* 7.6 *J =* 1.7	7.83 (dt) *J =* 7.7 *J =* 1.9	7.56 (m)	7.55 (dd) *J =* 7.7 *J =* 1.5
H-1″	-	4.92 (d) *J =* 7.8	-	4.90 (dd) *J =* 7.7 *J =* 6.4
H-2″	-	3.46 (m)	-	3.45 (m)
H-3″	-	3.64 (ddd) *J =* 9.0 *J =* 7.8 *J =* 3.3	-	3.63 (ddd) *J =* 9.0 *J =* 7.7 *J =* 3.2
H-4″	-	3.24 (t) *J =* 9.3	-	3.24 (m)
H-5″	-	3.46 (m)	-	3.45 (m)
H-6″	-	3.83 (m) 3.71 (m)	-	3.83 (m) 3.71 (m)
4″-OCH_3_	-	3.57 (s)	-	3.56 (s)
2″-OH	-	4.69 (m)	-	4.68 (m)
3″-OH	-	4.40 (d) *J =* 4.1	-	4.39 (d) *J =* 4.0
6″-OH	-	3.71 (m)	-	3.71 (m)

**Table 2:** ^13^C-NMR chemical shifts δ (ppm) and coupling constants *J* (Hz) of 2′-chloroflavanone (**1**), 3′-chloroflavanone (**2**) and their biotransformation products **1a** and **2a** in Acetone-d6, 151 MHz (Supplementary Information: Figures S4, S5, S22–S24, S39, S40 and S57–S59).

Carbon	Compound			
	1	1a	2	2a
C-2	77.2	77.0	79.5	79.6
C-3	43.7	43.6	44.9	44.8
C-4	191.3	191.2	191.4	191.3
C-4a	121.9	122.0	121.9	122.1
C-5	127.5	113.8	127.4	113.7
C-6	122.6	153.4	122.5	153.3
C-7	136.9	126.9	136.9	126.8
C-8	118.9	119.9	118.9	119.9
C-8a	162.3	157.7	162.2	157.5
C-1′	137.7	137.7	142.8	142.8
C-2′	132.5	132.5	127.3	127.3
C-3′	130.5	130.5	134.9	134.9
C-4′	130.9	130.9	129.3	129.3
C-5′	128.6	128.6	131.3	131.3
C-6′	128.7	128.7	125.8	125.8
C-1″	-	102.6	-	102.6
C-2″	-	75.0	-	75.0
C-3″	-	77.9	-	77.9
C-4″	-	80.0	-	80.0
C-5″	-	77.3	-	77.0
C-6″	-	62.0	-	62.0
4″-OCH_3_	-	60.5	-	60.5

**Table 3:** ^1^H-NMR chemical shifts δ (ppm) and coupling constants *J* (Hz) of 4′-chloroflavanone (**3**), 6-chloroflavanone (**4**) and their biotransformation products **3a** and **4a** in Acetone-d6, 600 MHz (Supplementary Information: Figures S72, S73, S89–S91, S108–S110 and S124–S126).

Proton.	Compound			
	3	3a	4	4a
H-2	5.67 (dd) *J =* 13.1 *J =* 2.8	5.62 (dd) *J =* 13.1 *J =* 2.9	5.69 (dd) *J =* 13.1 *J =* 2.9	5.63 (dd) *J =* 13.0 *J =* 2.8
H-3_ax_	3.15 (dd) *J =* 16.8 *J =* 13.1	3.11 (dd) *J =* 16.8 *J =* 13.1	3.21 (dd) *J =* 16.9 *J =* 13.1	3.22 (m)
H-3_eq_	2.89 (m)	2.89 (m)	2.90 (dd) *J =* 16.9 *J =* 2.9	2.88 (m) *J =* 2.9
H-5	7.85 (m)	7.46 (t) *J =* 3.1	7.77 (d) *J =* 2.7	7.75 (d) *J =* 2.6
H-6	7.11 (m)	-	-	-
H-7	7.59 (m)	7.34 (dd) *J =* 9.0 *J =* 3.1	7.59 (m)	7.57 (dd) *J =* 8.8 *J =* 2.7
H-8	7.11 (m)	7.04 (dd) *J =* 9.0 *J =* 1.2	7.15 (d) *J =* 8.8	7.12 (m)
H-2′	7.64 (m)	7.62 (m)	7.59 (m)	7.51 (d) *J =* 8.5
H-3′	7.49 (m)	7.49 (m)	7.47 (m)	7.12 (m)
H-4′	-	-	7.41 (m)	-
H-5′	7.49 (m)	7.49 (m)	7.47 (m)	7.12 (m)
H-6′	7.64 (m)	7.62 (m)	7.59 (m)	7.51 (d) *J =* 8.5
H-1″	-	4.89 (dd) *J =* 7.7 *J =* 6.6	-	4.98 (d) *J =* 7.8
H-2″	-	3.46 (m)	-	3.48 (m)
H-3″	-	3.62 (m)	-	3.66 (m)
H-4″	-	3.23 (t) *J =* 9.3	-	3.22 (m)
H-5″	-	3.46 (m)	-	3.48 (m)
H-6″	-	3.82 (m) 3.70 (m)	-	3.82 (m) 3.66 (m)
4″-OCH_3_	-	3.56 (s)	-	3.56 (s)
2″-OH	-	4.71 (s)	-	4.67 (d) *J =* 4.1
3″-OH	-	4.42 (s)	-	4.43 (d) *J =* 4.0
6″-OH	-	3.75 (m)	-	3.66 (m)

**Table 4:** ^13^C-NMR chemical shifts δ (ppm) and coupling constants *J* (Hz) of 4′-chloroflavanone (**3**), 6-chloroflavanone (**4**) and their biotransformation products **3a** and **4a** in Acetone-d6, 151 MHz (Supplementary Information: Figures S74, S75, S92–S94, S109, S110 and S127–S129).

Carbon		Compound		
	3	3a	4	4a
C-2	79.6	79.7	80.7	80.3
C-3	44.9	44.8	44.5	44.3
C-4	191.5	191.5	190.9	191.1
C-4a	121.9	122.0	122.9	122.9
C-5	127.4	113.8	126.4	126.4
C-6	122.4	153.3	127.2	127.0
C-7	136.9	126.8	136.5	136.4
C-8	118.9	119.9	121.2	121.2
C-8a	162.2	157.6	161.1	161.1
C-1′	139.3	139.4	139.9	133.3
C-2′	129.1	129.1	127.4	128.8
C-3′	129.6	129.6	129.5	117.4
C-4′	134.6	134.6	129.5	159.0
C-5′	129.6	129.6	129.5	117.4
C-6′	129.1	129.1	127.4	128.8
C-1″	-	102.6	-	101.5
C-2″	-	75.0	-	74.9
C-3″	-	77.9	-	78.0
C-4″	-	80.0	-	80.1
C-5″	-	77.0	-	77.0
C-6″	-	62.0	-	62.1
4″-OCH_3_	-	60.5	-	60.6

**Table 5:** Pharmacokinetics, drug-likeness, and biological activity prediction data from the SwissADME online tool of compounds **1**, **1a**, **2**, **2a**, **3**, **3a**, **4**, **4a**, and **5**.

Activity/Parameter	1	1a	2	2a	3	3a	4	4a	5
**Lipophilicity consensus Log Po/w**	3.46	1.64	3.47	1.58	3.47	1.62	3.46	1.60	2.93
**Water solubility [mg/mL]**	0.0147	0.1600	0.0147	0.1600	0.0147	0.1600	0.0147	0.1600	0.0485
**Gastrointestinal absorption**	High	High	High	High	High	High	High	High	High
**BBB permeant**	Yes	No	Yes	No	Yes	No	Yes	No	Yes
**P-gp substrate**	No	Yes	No	Yes	No	Yes	No	Yes	No
**CYP1A2 inhibitor**	Yes	No	Yes	No	Yes	No	Yes	No	Yes
**CYP2C9 inhibitor**	No	No	Yes	No	Yes	No	No	No	No
**CYP2C19 inhibitor**	Yes	No	Yes	No	Yes	No	Yes	No	No
**CYP2D6 inhibitor**	No	No	No	No	No	No	No	No	No
**CYP3A4 inhibitor**	No	Yes	No	Yes	No	Yes	No	Yes	No
**Log Kp (skin permeation) [cm/s]**	−5.20	−8.08	−5.20	−8.08	−5.20	−8.08	−5.20	−8.08	−5.44
**Drug-likeness (Lipinski, Ghose, Veber, Egan, and Muegge)**	Yes	Yes	Yes	Yes	Yes	Yes	Yes	Yes	Yes
**Abbott bioavailability score (ABS)**	0.55	0.55	0.55	0.55	0.55	0.55	0.55	0.55	0.55
**PAINS**	0 alert	0 alert	0 alert	0 alert	0 alert	0 alert	0 alert	0 alert	0 alert

**Table 6:** Antimicrobial activity of compounds **1**, **1a**, **2**, **2a**, **3**, **3a**, **4**, **4a**, and **5** against microbial strains: *Enterococcus faecalis* ATCC 19433, *Staphylococcus aureus* ATCC 29213, *Lactobacillus acidophilus* ATCC 4356, *Escherichia coli* ATCC 25922, and *Candida albicans* ATCC 10231.

Compound	*E. faecalis* (Gram+)	*S. aureus* (Gram+)	*L. acidophilus* (Gram+)	*E. coli* (Gram−)	*C. albicans* (yeast)
	ΔOD (0.05%)				
**Control**	1.86	1.77	1.61	1.48	1.35
**Oxytetracycline**	0	0	0	0	-
**Cycloheximide**	-	-	-	-	0
**1**	1.56	1.66	0.98	1.99	0.92
**1a**	1.87	1.97	1.36	2.00	1.40
**2**	1.67	1.10	0.94	1.82	1.00
**2a**	1.81	1.88	0.95	1.17	0.84
**3**	1.11	0.70	0.76	0.98	0.41
**3a**	1.82	1.62	1.16	1.77	1.77
**4**	1.33	1.01	1.11	1.62	1.11
**4a**	1.86	1.72	0.97	1.82	1.12
**5**	1.76	1.17	1.06	1.84	0.88

**Table 7:** Summary of the biotransformation yields and key reaction conditions.

Substrate	Fungal Strain	Biotransformation Yield (%)
2′-Chloroflavanone (**1**)	*B. bassiana* KCH J1.5	6.6
	*I. fumosorosea* KCH J2	complex mixture
3′-Chloroflavanone (**2**)	*B. bassiana* KCH J1.5	5.1
	*I. fumosorosea* KCH J2	complex mixture
4′-Chloroflavanone (**3**)	*B. bassiana* KCH J1.5	5.8
	*I. fumosorosea* KCH J2	complex mixture
6-Chloroflavanone (**4**)	*B. bassiana* KCH J1.5	complex mixture
	*I. fumosorosea* KCH J2	7.8

Reaction conditions: flask volume: 2 L, medium: modified Sabouraud medium, medium volume: 500 mL, inoculum: 5 mL preculture, substrate concentration: 0.39 mM, substrate solvent: DMSO (2.0 mL), duration of biotransformation: 8 days.

## Data Availability

The original data presented in the study are included in the article and Supplementary Materials. Raw data are openly available in the Wrocław University of Environmental and Life Sciences Repository (https://bazawiedzy.upwr.edu.pl/info/researchdata/UPWR974e21204d5345ec8c9b7201fea3f05c/, accessed on 13 August 2025).

## Supplementary Materials

The following supporting information can be downloaded at https://www.mdpi.com/article/10.3390/ijms262010138/s1.

## References

[1] Dias T.A., Duarte C.L., Lima C.F., Proença M.F., Pereira-Wilson C. (2013). Superior Anticancer Activity of Halogenated Chalcones and Flavonols over the Natural Flavonol Quercetin. Eur. J. Med. Chem..

[2] Proença C., Ribeiro D., Soares T., Tomé S.M., Silva A.M.S., Lima J.L.F.C., Fernandes E., Freitas M. (2017). Chlorinated Flavonoids Modulate the Inflammatory Process in Human Blood. Inflammation.

[3] Freitas M., Ribeiro D., Tomé S.M., Silva A.M.S., Fernandes E. (2014). Synthesis of Chlorinated Flavonoids with Anti-Inflammatory and pro-Apoptotic Activities in Human Neutrophils. Eur. J. Med. Chem..

[4] Binsack R., Boersma B.J., Patel R.P., Kirk M., White C.R., Darley-Usmar V., Barnes S., Zhou F., Parks D.A. (2001). Enhanced Antioxidant Activity after Chlorination of Quercetin by Hypochlorous Acid. Alcohol. Clin. Exp. Res..

[5] Marzec E., Świtalska M., Winiewska-Szajewska M., Wójcik J., Wietrzyk J., Maciejewska A.M., Poznański J., Mieczkowski A. (2020). The Halogenation of Natural Flavonoids, Baicalein and Chrysin, Enhances Their Affinity to Human Protein Kinase CK2. IUBMB Life.

[6] Zhang X., Khalidi O., Kim S.Y., Wang R., Schultz V., Cress B.F., Gross R.A., Koffas M.A.G., Linhardt R.J. (2016). Synthesis and Biological Evaluation of 5,7-Dihydroxyflavanone Derivatives as Antimicrobial Agents. Bioorg. Med. Chem. Lett..

[7] Fowler Z.L., Shah K., Panepinto J.C., Jacobs A., Koffas M.A.G. (2011). Development of Non-Natural Flavanones as Antimicrobial Agents. PLoS ONE.

[8] Xiao J., Muzashvili T.S., Georgiev M.I. (2014). Advances in the biotechnological glycosylation of valuable flavonoids. Biotechnol. Adv..

[9] Vazhappilly C.G., Amararathna M., Cyril A.C., Linger R., Matar R., Merheb M., Ramadan W.S., Radhakrishnan R., Rupasinghe H.P.V. (2021). Current Methodologies to Refine Bioavailability, Delivery, and Therapeutic Efficacy of Plant Flavonoids in Cancer Treatment. J. Nutr. Biochem..

[10] Cyboran-Mikołajczyk S., Matczak K., Olchowik-Grabarek E., Sękowski S., Nowicka P., Krawczyk-Łebek A., Kostrzewa-Susłow E. (2024). The Influence of the Chlorine Atom on the Biological Activity of 2′-Hydroxychalcone in Relation to the Lipid Phase of Biological Membranes—Anticancer and Antimicrobial Activity. Chem. Biol. Interact..

[11] Dudek A., Szulc N., Pawlak A., Strugała-Danak P., Krawczyk-Łebek A., Perz M., Kostrzewa-Susłow E., Pruchnik H. (2024). Structural Investigation of Interactions between Halogenated Flavonoids and the Lipid Membrane along with Their Role as Cytotoxic Agents. Sci. Rep..

[12] Hurtová M., Káňová K., Dobiasová S., Holasová K., Čáková D., Hoang L., Biedermann D., Kuzma M., Cvačka J., Křen V. (2022). Selectively Halogenated Flavonolignans—Preparation and Antibacterial Activity. Int. J. Mol. Sci..

[13] Richards M., Bird A.E., Munden J.E. (1969). Chlorflavonin, a New Antifungal Antibiotic. J. Antibiot..

[14] Munden J.E., Butterworth D., Hanscomb G., Verrall M.S. (1970). Production of Chlorflavonin, an Antifungal Metabolite of Aspergillus Candidus. Appl. Microbiol..

[15] Marchelli R., Vining L.C. (1973). The Biosynthetic Origin of Chlorflavonin, a Flavonoid Antibiotic from Aspergillus Candidus. Can. J. Biochem..

[16] Ma J., Zhang X.L., Wang Y., Zheng J.Y., Wang C.Y., Shao C.L. (2017). Aspergivones A and B, Two New Flavones Isolated from a Gorgonian-Derived Aspergillus Candidus Fungus. Nat. Prod. Res..

[17] Freitas M., Ribeiro D., Sara T.M., Artur S.M.S., Fernandes E. (2014). Anti-Inflammatory and pro-Apoptotic Activities of Chlorinated Flavonoids in Human Neutrophils. Free Radic. Biol. Med..

[18] Krych-Madej J., Stawowska K., Gebicka L. (2016). Oxidation of Flavonoids by Hypochlorous Acid: Reaction Kinetics and Antioxidant Activity Studies. Free Radic. Res..

[19] Kamboj R.C., Sharma G., Kumar D., Arora R., Sharma C., Aneja K.R. (2011). An Environmentally Sound Approach for the Synthesis of Some Flavanones and Their Antimicrobial Activity. Int. J. ChemTech Res..

[20] Xiao J. (2017). Dietary Flavonoid Aglycones and Their Glycosides: Which Show Better Biological Significance?. Crit. Rev. Food. Sci. Nutr..

[21] Thilakarathna S.H., Rupasinghe V.H.P. (2013). Flavonoid Bioavailability and Attempts for Bioavailability Enhancement. Nutrients.

[22] Dias M.C., Pinto D.C.G.A., Silva A.M.S. (2021). Plant Flavonoids: Chemical Characteristics and Biological Activity. Molecules.

[23] Hollman P. (2004). Absorption, Bioavailability, and Metabolism of Flavonoids. Pharm. Biol..

[24] Dou F., Wang Z., Li G., Dun B. (2019). Microbial Transformation of Flavonoids by Isaria Fumosorosea ACCC 37814. Molecules.

[25] Krawczyk-Łebek A., Żarowska B., Janeczko T., Kostrzewa-Susłow E. (2025). Antimicrobial Activity of New Glycoside Derivatives of Chloroflavones Obtained by Fungal Biotransformation. Sci. Rep..

[26] Tronina T., Łużny M., Dymarska M., Urbaniak M., Kozłowska E., Piegza M., Stępień Ł., Janeczko T. (2023). Glycosylation of Quercetin by Selected Entomopathogenic Filamentous Fungi and Prediction of Its Products’ Bioactivity. Int. J. Mol. Sci..

[27] Xie L., Zhang L., Bai J., Yue Q., Zhang M., Li J., Wang C., Xu Y. (2019). Methylglucosylation of Phenolic Compounds by Fungal Glycosyltransferase-Methyltransferase Functional Modules. J. Agric. Food Chem..

[28] Weng Q., Zhang X., Chen W., Hu Q. (2019). Secondary Metabolites and the Risks of *Isaria fumosorosea* and *Isaria farinosa*. Molecules.

[29] Amobonye A., Bhagwat P., Pandey A., Singh S., Pillai S. (2020). Biotechnological Potential of Beauveria Bassiana as a Source of Novel Biocatalysts and Metabolites. Crit. Rev. Biotechnol..

[30] Ávila-Hernández J.G., Carrillo-Inungaray M.L., De-La-Cruz-Quiroz R., Wong-Paz J.E., Muñiz-Márquez D.B., Parra R., Aguilar C.N., Aguilar-Zárate P. (2020). Beauveria Bassiana Secondary Metabolites: A Review inside Their Production Systems, Biosynthesis, and Bioactivities. Mex. J. Biotechnol..

[31] Pedrini N. (2022). The Entomopathogenic Fungus Beauveria Bassiana Shows Its Toxic Side within Insects: Expression of Genes Encoding Secondary Metabolites during Pathogenesis. J. Fungi.

[32] Krawczyk-Łebek A., Żarowska B., Janeczko T., Kostrzewa-Susłow E. (2024). Antimicrobial Activity of Chalcones with a Chlorine Atom and Their Glycosides. Int. J. Mol. Sci..

[33] Krawczyk-Łebek A., Żarowska B., Dymarska M., Janeczko T., Kostrzewa-Susłow E. (2024). Synthesis, Fungal Biotransformation, and Evaluation of the Antimicrobial Potential of Chalcones with a Chlorine Atom. Sci. Rep..

[34] Fontana C., Widmalm G. (2023). Primary Structure of Glycans by NMR Spectroscopy. Chem. Rev..

[35] Krawczyk-Łebek A., Dymarska M., Janeczko T., Kostrzewa-Susłow E. (2021). Fungal Biotransformation of 2′-Methylflavanone and 2′-Methylflavone as a Method to Obtain Glycosylated Derivatives. Int. J. Mol. Sci..

[36] Krawczyk-Łebek A., Dymarska M., Janeczko T., Kostrzewa-Susłow E. (2022). 4′-Methylflavanone Glycosides Obtained Using Biotransformation in the Entomopathogenic Filamentous Fungi Cultures as Potential Anticarcinogenic, Antimicrobial, and Hepatoprotective Agents. Int. J. Mol. Sci..

[37] Krawczyk-Łebek A., Dymarska M., Janeczko T., Kostrzewa-Susłow E. (2020). Entomopathogenic Filamentous Fungi as Biocatalysts in Glycosylation of Methylflavonoids. Catalysts.

[38] Shi Y.Q., Xin X.L., Zhang H.C., Zhang B.J., Wang C.Y., Hou J., Yuan Q.P., Deng S., Tian Y., Ma X.C. (2012). Microbial Transformation of Norkurarinone by Cunninghamella Blakesleana AS 3.970. J. Asian Nat. Prod. Res..

[39] Shi Y.Q., Xin X.L., Yuan Q.P., Wang C.Y., Zhang B.J., Hou J., Tian Y., Deng S., Huang S.S., Ma X.C. (2012). Microbial Biotransformation of Kurarinone by Cunninghamella Echinulata AS 3.3400. J. Asian Nat. Prod. Res..

[40] Shimoda K., Kubota N., Taniuchi K., Sato D., Nakajima N., Hamada H., Hamada H. (2010). Biotransformation of Naringin and Naringenin by Cultured Eucalyptus Perriniana Cells. Phytochemistry.

[41] Marvalin C., Azerad R. (2011). Microbial Glucuronidation of Polyphenols. J. Mol. Catal. B Enzym..

[42] Shimoda K., Hamada H. (2010). Production of Hesperetin Glycosides by Xanthomonas Campestris and Cyclodextrin Glucanotransferase and Their Anti-Allergic Activities. Nutrients.

[43] Daina A., Michielin O., Zoete V. (2017). SwissADME: A Free Web Tool to Evaluate Pharmacokinetics, Drug-Likeness and Medicinal Chemistry Friendliness of Small Molecules. Sci. Rep..

[44] Daina A., Zoete V. (2016). A BOILED-Egg To Predict Gastrointestinal Absorption and Brain Penetration of Small Molecules. ChemMedChem.

[45] Zhang H., Hassan Y.I., Liu R., Mats L., Yang C., Liu C., Tsao R. (2020). Molecular Mechanisms Underlying the Absorption of Aglycone and Glycosidic Flavonoids in a Caco-2 BBe1 Cell Model. ACS Omega.

[46] Dahiya A., Majee C., Mazumder R., Priya N., Salahauddin, Atriya A. (2023). Insight into the Glycosylation Methods of the Flavonoids as an Approach to Enhance Its Bioavailability and Pharmacological Activities. Indian J. Pharm. Educ. Res..

[47] Loo D.D., Wright E.M., Hamilton K.L., Devor D.C. (2020). Sugar Transport across Epithelia. Studies of Epithelial Transporters and Ion Channels: Ion Channels and Transporters of Epithelia in Health and Disease.

[48] Kowsalya K., Vidya N., Halka J., Preetha J.S.Y., Saradhadevi M., Sahayarayan J.J., Gurusaravanan P., Arun M. (2025). Plant Glycosides and Glycosidases: Classification, Sources, and Therapeutic Insights in Current Medicine. Glycoconj. J..

[49] Hu L., Luo Y., Yang J., Cheng C. (2025). Botanical Flavonoids: Efficacy, Absorption, Metabolism and Advanced Pharmaceutical Technology for Improving Bioavailability. Molecules.

[50] Sánchez-Villamil J.P., Ochoa F.A., Portillo O.C., Montero J.M., Martinez-Galán J.P., Patricia J., Villamil S. (2023). Systematic Review of Antibacterial Activity of Naringin and Other Flavanones against Oral Pathogens. https://papers.ssrn.com/sol3/papers.cfm?abstract_id=4366409.

[51] Veiko A.G., Olchowik-Grabarek E., Sekowski S., Roszkowska A., Lapshina E.A., Dobrzynska I., Zamaraeva M., Zavodnik I.B. (2023). Antimicrobial Activity of Quercetin, Naringenin and Catechin: Flavonoids Inhibit Staphylococcus Aureus-Induced Hemolysis and Modify Membranes of Bacteria and Erythrocytes. Molecules.

[52] Murti Y., Mishra P. (2014). Synthesis and Evaluation of Flavanones as Anticancer Agents. Indian J. Pharm. Sci..

[53] Silva A.M.S., Tavares H.R., Barros A.I.N.R.A., Cavaleiro J.A.S. (1997). NMR and Structural and Conformational Features of 2’-Hydroxychalcones and Flavones. Spectrosc. Lett..

[54] Kozłowska E., Urbaniak M., Hoc N., Grzeszczuk J., Dymarska M., Stępień Ł., Pląskowska E., Kostrzewa-Susłow E., Janeczko T. (2018). Cascade Biotransformation of Dehydroepiandrosterone (DHEA) by Beauveria Species. Sci. Rep..

[55] Dymarska M., Grzeszczuk J., Urbaniak M., Janeczko T., Pląskowska E., Stępień Ł., Kostrzewa-Susłow E. (2017). Glycosylation of 6-Methylflavone by the Strain *Isaria fumosorosea* KCH J2. PLoS ONE.

[56] Kozłowska J., Potaniec B., Zarowska B., Anioł M. (2017). Synthesis and Biological Activity of Novel O-Alkyl Derivatives of Naringenin and Their Oximes. Molecules.

