# Supplementary material for: Fungal Biotransformation of Chloroflavanones and Antimicrobial Activity of Parent Compounds and Derived Products
Source: Int J Mol Sci. 2025 Oct 18;26(20):10138. doi: 10.3390/ijms262010138 (PMC12563750; doi:10.3390/ijms262010138)
Supplement: Supplementary file 1 [file ijms-26-10138-s001.zip › ijms-3854180-supplementary.pdf]

Fungal Biotransformation of Chloroflavanones and Antimicrobial Activity of Parent Compounds and Derived Products

Agnieszka Krawczyk-Łebek[1]*, Tomasz Janeczko[1], Barbara Żarowska[2], Edyta Kostrzewa-Susłow[1]

[1]Department of Food Chemistry and Biocatalysis, [2]Departement of Biotechnology and Microbiology, Faculty of Biotechnology and Food Science, Wrocław University of Environmental and Life Sciences, Wrocław, Poland

Table of contents

Figure S1. HPLC analysis of 2′-chloroflavanone (**1**).
Figure S2. ^{1}H NMR spectrum (*δ*, acetone-d6, 600 MHz) of 2′-chloroflavanone (**1**).
Figure S3. ^{1}H NMR spectrum expansion (*δ*, acetone-d6, 600 MHz) of 2′-chloroflavanone (**1**).
Figure S4. ^{13}C NMR spectrum (*δ*, acetone-d6, 151 MHz) of 2′-chloroflavanone (**1**).
Figure S5. ^{13}C NMR spectrum expansion (*δ*, acetone-d6, 151 MHz) of 2′-chloroflavanone (**1**).
Figure S6. COSY contour map – ^{1}H x ^{1}H of 2′-chloroflavanone (**1**).
Figure S7. COSY contour map – ^{1}H x ^{1}H expansion of 2′-chloroflavanone (**1**).
Figure S8. COSY contour map – ^{1}H x ^{1}H expansion of 2′-chloroflavanone (**1**).
Figure S9. HMQC contour map – ^{1}H x ^{13}C of 2′-chloroflavanone (**1**).
Figure S10. HMQC contour map – ^{1}H x ^{13}C of 2′-chloroflavanone (**1**).
Figure S11. HMQC contour map – ^{1}H x ^{13}C expansion of 2′-chloroflavanone (**1**).
Figure S12. HMBC contour map – ^{1}H x ^{13}C of 2′-chloroflavanone (**1**).
Figure S13. HMBC contour map – ^{1}H x ^{13}C expansion of 2′-chloroflavanone (**1**).
Figure S14. HMBC contour map – ^{1}H x ^{13}C expansion of 2′-chloroflavanone (**1**).
Figure S15. HMBC contour map – ^{1}H x ^{13}C expansion of 2′-chloroflavanone (**1**).
Figure S16. HMBC contour map – ^{1}H x ^{13}C expansion of 2′-chloroflavanone (**1**).
Figure S17. 2′-Chloroflavanone (**1**) physicochemical and ADME parameters prediction using the SwissADME modelling.

Figure S18. HPLC analysis of 2′-chloroflavanone 6-*O*-*β*-D-(4′′-*O*-methyl)-glucopyranoside (**1a**).
Figure S19. ^{1}H NMR spectrum (*δ*, acetone-d6, 600 MHz) of 2′-chloroflavanone 6-*O*-*β*-D-(4′′-*O*-methyl)-glucopyranoside (**1a**).
Figure S20. ^{1}H NMR spectrum expansion (*δ*, acetone-d6, 600 MHz) of 2′-chloroflavanone 6-*O*-*β*-D-(4′′-*O*-methyl)-glucopyranoside (**1a**).
Figure S21. ^{1}H NMR spectrum expansion (*δ*, acetone-d6, 600 MHz) of 2′-chloroflavanone 6-*O*-*β*-D-(4′′-*O*-methyl)-glucopyranoside (**1a**).
Figure S22. ^{13}C NMR spectrum (*δ*, acetone-d6, 151 MHz) of 2′-chloroflavanone 6-*O*-*β*-D-(4′′-*O*-methyl)-glucopyranoside (**1a**).
Figure S23. ^{13}C NMR spectrum expansion (*δ*, acetone-d6, 151 MHz) of 2′-chloroflavanone 6-*O*-*β*-D-(4′′-*O*-methyl)-glucopyranoside (**1a**).

Figure S24. ^{13}C NMR spectrum expansion (*δ*, acetone-d6, 151 MHz) of 2′-chloroflavanone 6-*O*-*β*-D-(4′′-*O*-methyl)-glucopyranoside (**1a**).
Figure S25. COSY contour map – ^{1}H x ^{1}H of 2′-chloroflavanone 6-*O*-*β*-D-(4′′-*O*-methyl)-glucopyranoside (**1a**).
Figure S26. COSY contour map – ^{1}H x ^{1}H expansion of 2′-chloroflavanone 6-*O*-*β*-D-(4′′-*O*-methyl)-glucopyranoside (**1a**).
Figure S27. COSY contour map – ^{1}H x ^{1}H expansion of 2′-chloroflavanone 6-*O*-*β*-D-(4′′-*O*-methyl)-glucopyranoside (**1a**).
Figure S28. HMQC contour map – ^{1}H x ^{13}C of 2′-chloroflavanone 6-*O*-*β*-D-(4′′-*O*-methyl)-glucopyranoside (**1a**).
Figure S29. HMQC contour map – ^{1}H x ^{13}C expansion of 2′-chloroflavanone 6-*O*-*β*-D-(4′′-*O*-methyl)-glucopyranoside (**1a**).
Figure S30. HMQC contour map – ^{1}H x ^{13}C expansion of 2′-chloroflavanone 6-*O*-*β*-D-(4′′-*O*-methyl)-glucopyranoside (**1a**).
Figure S31. HMBC contour map – ^{1}H x ^{13}C of 2′-chloroflavanone 6-*O*-*β*-D-(4′′-*O*-methyl)-glucopyranoside (**1a**).
Figure S32. HMBC contour map – ^{1}H x ^{13}C expansion of 2′-chloroflavanone 6-*O*-*β*-D-(4′′-*O*-methyl)-glucopyranoside (**1a**).
Figure S33. HMBC contour map – ^{1}H x ^{13}C expansion of 2′-chloroflavanone 6-*O*-*β*-D-(4′′-*O*-methyl)-glucopyranoside (**1a**).
Figure S34. HMBC contour map – ^{1}H x ^{13}C expansion of 2′-chloroflavanone 6-*O*-*β*-D-(4′′-*O*-methyl)-glucopyranoside (**1a**).
Figure S35. 2′-Chloroflavanone 6-*O*-*β*-D-(4′′-*O*-methyl)-glucopyranoside (**1a**) physicochemical and ADME parameters prediction using the SwissADME modelling.

Figure S36. HPLC analysis of 3′-chloroflavanone (**2**).
Figure S37. ^{1}H NMR spectrum (*δ*, acetone-d6, 600 MHz) of 3′-chloroflavanone (**2**).
Figure S38. ^{1}H NMR spectrum expansion (*δ*, acetone-d6, 600 MHz) of 3′-chloroflavanone (**2**).
Figure S39. ^{13}C NMR spectrum (*δ*, acetone-d6, 151 MHz) of 3′-chloroflavanone (**2**).
Figure S40. ^{13}C NMR spectrum expansion (*δ*, acetone-d6, 151 MHz) of 3′-chloroflavanone (**2**).
Figure S41. COSY contour map – ^{1}H x ^{1}H of 3′-chloroflavanone (**2**).
Figure S42. COSY contour map – ^{1}H x ^{1}H expansion of 3′-chloroflavanone (**2**).
Figure S43. COSY contour map – ^{1}H x ^{1}H expansion of 3′-chloroflavanone (**2**).
Figure S44. HMQC contour map – ^{1}H x ^{13}C of 3′-chloroflavanone (**2**).
Figure S45. HMQC contour map – ^{1}H x ^{13}C expansion of 3′-chloroflavanone (**2**).
Figure S46. HMQC contour map – ^{1}H x ^{13}C expansion of 3′-chloroflavanone (**2**).
Figure S47. HMBC contour map – ^{1}H x ^{13}C of 3′-chloroflavanone (**2**).
Figure S48. HMBC contour map – ^{1}H x ^{13}C expansion of 3′-chloroflavanone (**2**).
Figure S49. HMBC contour map – ^{1}H x ^{13}C expansion of 3′-chloroflavanone (**2**).
Figure S50. HMBC contour map – ^{1}H x ^{13}C expansion of 3′-chloroflavanone (**2**).
Figure S51. HMBC contour map – ^{1}H x ^{13}C expansion of 3′-chloroflavanone (**2**).
Figure S52. 3′-Chloroflavanone (**2**) physicochemical and ADME parameters prediction using the SwissADME modelling.

Figure S53. HPLC analysis of 3′-chloroflavanone 6-*O*-*β*-D-(4′′-*O*-methyl)-glucopyranoside (**2a**).
Figure S54. ^{1}H NMR spectrum (*δ*, acetone-d6, 600 MHz) of 3′-chloroflavanone 6-*O*-*β*-D-(4′′-*O*-methyl)-glucopyranoside (**2a**).
Figure S55. ^{1}H NMR spectrum expansion (*δ*, acetone-d6, 600 MHz) of 3′-chloroflavanone 6-*O*-*β*-D-(4′′-*O*-methyl)-glucopyranoside (**2a**).
Figure S56. ^{1}H NMR spectrum expansion (*δ*, acetone-d6, 600 MHz) of 3′-chloroflavanone 6-*O*-*β*-D-(4′′-*O*-methyl)-glucopyranoside (**2a**).

Figure S57. ^{13}C NMR spectrum (*δ*, acetone-d6, 151 MHz) of 3′-chloroflavanone 6-*O*-*β*-D-(4′′-*O*-methyl)-glucopyranoside (**2a**).
Figure S58. ^{13}C NMR spectrum expansion (*δ*, acetone-d6, 151 MHz) of 3′-chloroflavanone 6-*O*-*β*-D-(4′′-*O*-methyl)-glucopyranoside (**2a**).
Figure S59. ^{13}C NMR spectrum expansion (*δ*, acetone-d6, 151 MHz) of 3′-chloroflavanone 6-*O*-*β*-D-(4′′-*O*-methyl)-glucopyranoside (**2a**).
Figure S60. COSY contour map – ^{1}H x ^{1}H of 3′-chloroflavanone 6-*O*-*β*-D-(4′′-*O*-methyl)-glucopyranoside (**2a**).
Figure S61. COSY contour map – ^{1}H x ^{1}H expansion of 3′-chloroflavanone 6-*O*-*β*-D-(4′′-*O*-methyl)-glucopyranoside (**2a**).
Figure S62. COSY contour map – ^{1}H x ^{1}H expansion of 3′-chloroflavanone 4′-*O*-*β*-D-(4′′-*O*-methyl)-glucopyranoside (**2a**).
Figure S63. HMQC contour map – ^{1}H x ^{13}C of 3′-chloroflavanone 6-*O*-*β*-D-(4′′-*O*-methyl)-glucopyranoside (**2a**).
Figure S64. HMQC contour map – ^{1}H x ^{13}C expansion of 3′-chloroflavanone 6-*O*-*β*-D-(4′′-*O*-methyl)-glucopyranoside (**2a**).
Figure S65. HMQC contour map – ^{1}H x ^{13}C expansion of 3′-chloroflavanone 6-*O*-*β*-D-(4′′-*O*-methyl)-glucopyranoside (**2a**).
Figure S66. HMBC contour map – ^{1}H x ^{13}C of 3′-chloroflavanone 6-*O*-*β*-D-(4′′-*O*-methyl)-glucopyranoside (**2a**).
Figure S67. HMBC contour map – ^{1}H x ^{13}C expansion of 3′-chloroflavanone 6-*O*-*β*-D-(4′′-*O*-methyl)-glucopyranoside (**2a**).
Figure S68. HMBC contour map – ^{1}H x ^{13}C expansion of 3′-chloroflavanone 6-*O*-*β*-D-(4′′-*O*-methyl)-glucopyranoside (**2a**).
Figure S69. HMBC contour map – ^{1}H x ^{13}C expansion of 3′-chloroflavanone 4′-*O*-*β*-D-(4′′-*O*-methyl)-glucopyranoside (**2a**).
Figure S70. 3′-Chloroflavanone 6-*O*-*β*-D-(4′′-*O*-methyl)-glucopyranoside (**2a**) physicochemical and ADME parameters prediction using the SwissADME modelling.

Figure S71. HPLC analysis of 4′-chloroflavanone (**3**).
Figure S72. ^{1}H NMR spectrum (*δ*, acetone-d6, 600 MHz) of 4′-chloroflavanone (**3**).
Figure S73. ^{1}H NMR spectrum expansion (*δ*, acetone-d6, 600 MHz) of 4′-chloroflavanone (**3**).
Figure S74. ^{13}C NMR spectrum (*δ*, acetone-d6, 151 MHz) of 4′-chloroflavanone (**3**).
Figure S75. ^{13}C NMR spectrum expansion (*δ*, acetone-d6, 151 MHz) of 4′-chloroflavanone (**3**).
Figure S76. COSY contour map – ^{1}H x ^{1}H of 4′-chloroflavanone (**3**).
Figure S77. COSY contour map – ^{1}H x ^{1}H expansion of 4′-chloroflavanone (**3**).
Figure S78. COSY contour map – ^{1}H x ^{1}H expansion of 4′-chloroflavanone (**3**).
Figure S79. HMQC contour map – ^{1}H x ^{13}C of 4′-chloroflavanone (**3**).
Figure S80. HMQC contour map – ^{1}H x ^{13}C expansion of 4′-chloroflavanone (**3**).
Figure S81. HMQC contour map – ^{1}H x ^{13}C expansion of 4′-chloroflavanone (**3**).
Figure S82. HMBC contour map – ^{1}H x ^{13}C of 4′-chloroflavanone (**3**).
Figure S83. HMBC contour map – ^{1}H x ^{13}C expansion of 4′-chloroflavanone (**3**).
Figure S84. HMBC contour map – ^{1}H x ^{13}C expansion of 4′-chloroflavanone (**3**).
Figure S85. HMBC contour map – ^{1}H x ^{13}C expansion of 4′-chloroflavanone (**3**).
Figure S86. HMBC contour map – ^{1}H x ^{13}C expansion of 4′-chloroflavanone (**3**).
Figure S87. 4′-Chloroflavanone (**3**) physicochemical and ADME parameters prediction using the SwissADME modelling.

Figure S88. HPLC analysis of 4′-chloroflavanone 6-*O*-*β*-D-(4′′-*O*-methyl)-glucopyranoside (**3a**).
Figure S89. ^{1}H NMR spectrum (*δ*, acetone-d6, 600 MHz) of 4′-chloroflavanone 6-*O*-*β*-D-(4′′-*O*-methyl)-glucopyranoside (**3a**).

Figure S90. ^{1}H NMR spectrum expansion (*δ,* acetone-d6, 600 MHz) of 4′-chloroflavanone 6-*O*-*β*-D-(4′′-*O*-methyl)-glucopyranoside (**3a**).
Figure S91. ^{1}H NMR spectrum expansion (*δ,* acetone-d6, 600 MHz) of 4′-chloroflavanone 6-*O*-*β*-D-(4′′-*O*-methyl)-glucopyranoside (**3a**).
Figure S92. ^{13}C NMR spectrum (*δ,* acetone-d6, 151 MHz) of 4′-chloroflavanone 6-*O*-*β*-D-(4′′-*O*-methyl)-glucopyranoside (**3a**).
Figure S93. ^{13}C NMR spectrum expansion (*δ,* acetone-d6, 151 MHz) of 4′-chloroflavanone 6-*O*-*β*-D-(4′′-*O*-methyl)-glucopyranoside (**3a**).
Figure S94. ^{13}C NMR spectrum expansion (*δ,* acetone-d6, 151 MHz) of 4′-chloroflavanone 6-*O*-*β*-D-(4′′-*O*-methyl)-glucopyranoside (**3a**).
Figure S95. COSY contour map – ^{1}H x ^{1}H of 4′-chloroflavanone 6-*O*-*β*-D-(4′′-*O*-methyl)-glucopyranoside (**3a**).
Figure S96. COSY contour map – ^{1}H x ^{1}H expansion of 4′-chloroflavanone 6-*O*-*β*-D-(4′′-*O*-methyl)-glucopyranoside (**3a**).
Figure S97. COSY contour map – ^{1}H x ^{1}H expansion of 4′-chloroflavanone 6-*O*-*β*-D-(4′′-*O*-methyl)-glucopyranoside (**3a**).
Figure S98. HMQC contour map – ^{1}H x ^{13}C of 4′-chloroflavanone 6-*O*-*β*-D-(4′′-*O*-methyl)-glucopyranoside (**3a**).
Figure S99. HMQC contour map – ^{1}H x ^{13}C expansion of 4′-chloroflavanone 6-*O*-*β*-D-(4′′-*O*-methyl)-glucopyranoside (**3a**).
Figure S100. HMQC contour map – ^{1}H x ^{13}C expansion of 4′-chloroflavanone 6-*O*-*β*-D-(4′′-*O*-methyl)-glucopyranoside (**3a**).
Figure S101. HMBC contour map – ^{1}H x ^{13}C of 4′-chloroflavanone 6-*O*-*β*-D-(4′′-*O*-methyl)-glucopyranoside (**3a**).
Figure S102. HMBC contour map – ^{1}H x ^{13}C expansion of 4′-chloroflavanone 6-*O*-*β*-D-(4′′-*O*-methyl)-glucopyranoside (**3a**).
Figure S103. HMBC contour map – ^{1}H x ^{13}C expansion of 4′-chloroflavanone 6-*O*-*β*-D-(4′′-*O*-methyl)-glucopyranoside (**3a**).
Figure S104. HMBC contour map – ^{1}H x ^{13}C expansion of 4′-chloroflavanone 6-*O*-*β*-D-(4′′-*O*-methyl)-glucopyranoside (**3a**).
Figure S105. 4′-Chloroflavanone 6-*O*-*β*-D-(4′′-*O*-methyl)-glucopyranoside (**3a**) physicochemical and ADME parameters prediction using the SwissADME modelling.

Figure S106. HPLC analysis of 6-chloroflavanone (**4**).
Figure S107. ^{1}H NMR spectrum (*δ,* acetone-d6, 600 MHz) of 6-chloroflavanone (**4**).
Figure S108. ^{1}H NMR spectrum expansion (*δ,* acetone-d6, 600 MHz) of 6-chloroflavanone (**4**).
Figure S109. ^{13}C NMR spectrum (*δ,* acetone-d6, 151 MHz) of 6-chloroflavanone (**4**).
Figure S110. ^{13}C NMR spectrum expansion (*δ,* acetone-d6, 151 MHz) of 6-chloroflavanone (**4**).
Figure S111. COSY contour map – ^{1}H x ^{1}H of 6-chloroflavanone (**4**).
Figure S112. COSY contour map – ^{1}H x ^{1}H expansion of 6-chloroflavanone (**4**).
Figure S113. COSY contour map – ^{1}H x ^{1}H expansion of 6-chloroflavanone (**4**).
Figure S114. HMQC contour map – ^{1}H x ^{13}C of 6-chloroflavanone (**4**).
Figure S115. HMQC contour map – ^{1}H x ^{13}C expansion of 6-chloroflavanone (**4**).
Figure S116. HMQC contour map – ^{1}H x ^{13}C expansion of 6-chloroflavanone (**4**).
Figure S117. HMBC contour map – ^{1}H x ^{13}C of 6-chloroflavanone (**4**).
Figure S118. HMBC contour map – ^{1}H x ^{13}C expansion of 6-chloroflavanone (**4**).
Figure S119. HMBC contour map – ^{1}H x ^{13}C expansion of 6-chloroflavanone (**4**).
Figure S120. HMBC contour map – ^{1}H x ^{13}C expansion of 6-chloroflavanone (**4**).
Figure S121. HMBC contour map – ^{1}H x ^{13}C expansion of 6-chloroflavanone (**4**).
Figure S122. 6-Chloroflavanone (**4**) physicochemical and ADME parameters prediction using the SwissADME modelling.

Figure S123. HPLC analysis of 6-chloroflavanone 4′-*O*-*β*-D-(4′′-*O*-methyl)-glucopyranoside (**4a**).
Figure S124. ^{1}H NMR spectrum (*δ*, acetone-d6, 600 MHz) of 6-chloroflavanone 4′-*O*-*β*-D-(4′′-*O*-methyl)-glucopyranoside (**4a**).
Figure S125. ^{1}H NMR spectrum expansion (*δ*, acetone-d6, 600 MHz) of 6-chloroflavanone 4′-*O*-*β*-D-(4′′-*O*-methyl)-glucopyranoside (**4a**).
Figure S126. ^{1}H NMR spectrum expansion (*δ*, acetone-d6, 600 MHz) of 6-chloroflavanone 4′-*O*-*β*-D-(4′′-*O*-methyl)-glucopyranoside (**4a**).
Figure S127. ^{13}C NMR spectrum (*δ*, acetone-d6, 151 MHz) of 6-chloroflavanone 4′-*O*-*β*-D-(4′′-*O*-methyl)-glucopyranoside (**4a**).
Figure S128. ^{13}C NMR spectrum expansion (*δ*, acetone-d6, 151 MHz) of 6-chloroflavanone 4′-*O*-*β*-D-(4′′-*O*-methyl)-glucopyranoside (**4a**).
Figure S129. ^{13}C NMR spectrum expansion (*δ*, acetone-d6, 151 MHz) of 6-chloroflavanone 4′-*O*-*β*-D-(4′′-*O*-methyl)-glucopyranoside (**4a**).
Figure S130. COSY contour map – ^{1}H x ^{1}H of 6-chloroflavanone 4′-*O*-*β*-D-(4′′-*O*-methyl)-glucopyranoside (**4a**).
Figure S131. COSY contour map – ^{1}H x ^{1}H expansion of 6-chloroflavanone 4′-*O*-*β*-D-(4′′-*O*-methyl)-glucopyranoside (**4a**).
Figure S132. COSY contour map – ^{1}H x ^{1}H expansion of 6-chloroflavanone 4′-*O*-*β*-D-(4′′-*O*-methyl)-glucopyranoside (**4a**).
Figure S133. HMQC contour map – ^{1}H x ^{13}C of 6-chloroflavanone 4′-*O*-*β*-D-(4′′-*O*-methyl)-glucopyranoside (**4a**).
Figure S134. HMQC contour map – ^{1}H x ^{13}C expansion of 6-chloroflavanone 4′-*O*-*β*-D-(4′′-*O*-methyl)-glucopyranoside (**4a**).
Figure S135. HMQC contour map – ^{1}H x ^{13}C expansion of 6-chloroflavanone 4′-*O*-*β*-D-(4′′-*O*-methyl)-glucopyranoside (**4a**).
Figure S136. HMBC contour map – ^{1}H x ^{13}C of 6-chloroflavanone 4′-*O*-*β*-D-(4′′-*O*-methyl)-glucopyranoside (**4a**).
Figure S137. HMBC contour map – ^{1}H x ^{13}C expansion of 6-chloroflavanone 4′-*O*-*β*-D-(4′′-*O*-methyl)-glucopyranoside (**4a**).
Figure S138. HMBC contour map – ^{1}H x ^{13}C expansion of 6-chloroflavanone 4′-*O*-*β*-D-(4′′-*O*-methyl)-glucopyranoside (**4a**).
Figure S139. HMBC contour map – ^{1}H x ^{13}C expansion of 6-chloroflavanone 4′-*O*-*β*-D-(4′′-*O*-methyl)-glucopyranoside (**4a**).
Figure S140. 6-Chloroflavanone 4′-*O*-*β*-D-(4′′-*O*-methyl)-glucopyranoside (**4a**) physicochemical and ADME parameters prediction using the SwissADME modelling.
Figure S141. Flavanone (**5**) physicochemical and ADME parameters prediction using the SwissADME modelling.

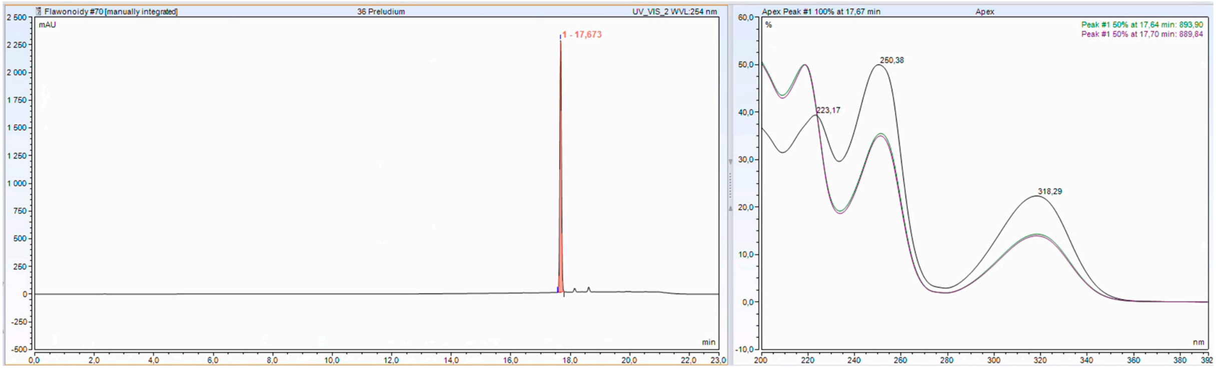

Figure S1. HPLC analysis of 2′-chloroflavanone (**1**).

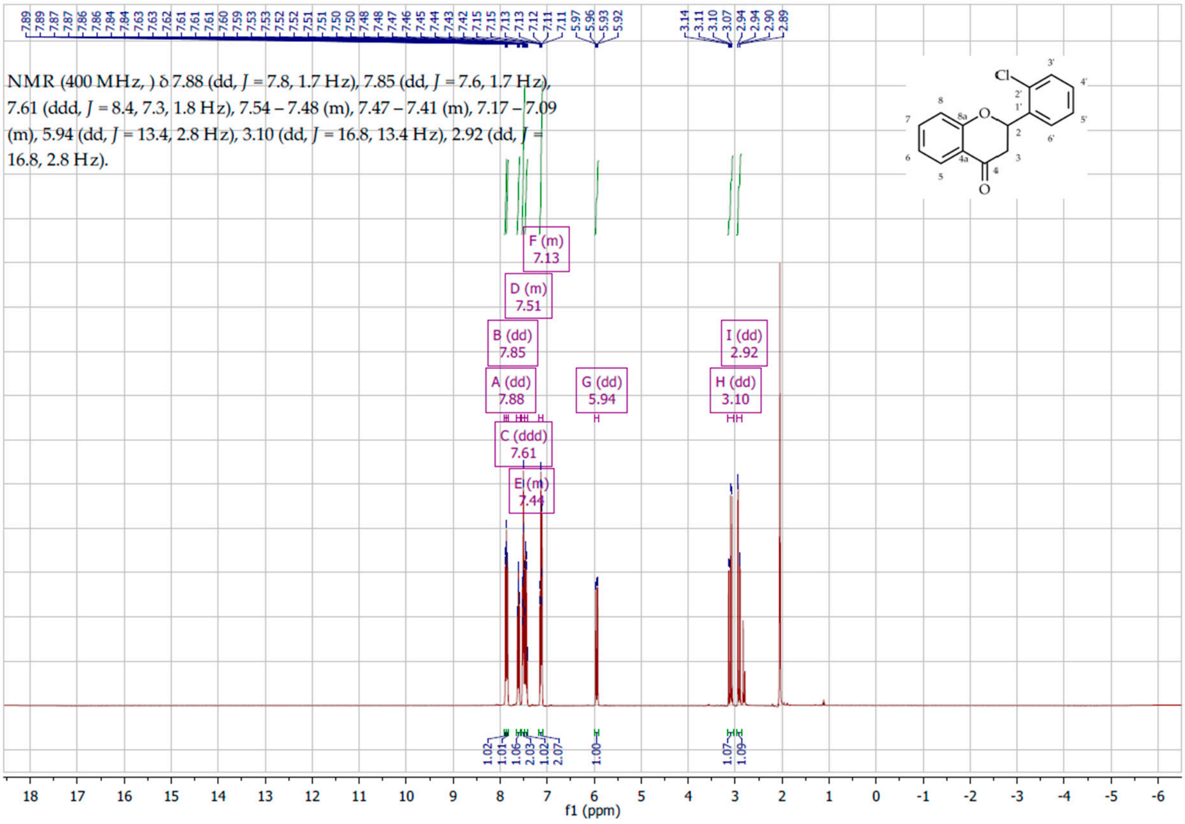

Figure S2. ^{1}H NMR spectrum (*δ*, acetone-d6, 600 MHz) of 2′-chloroflavanone (**1**).

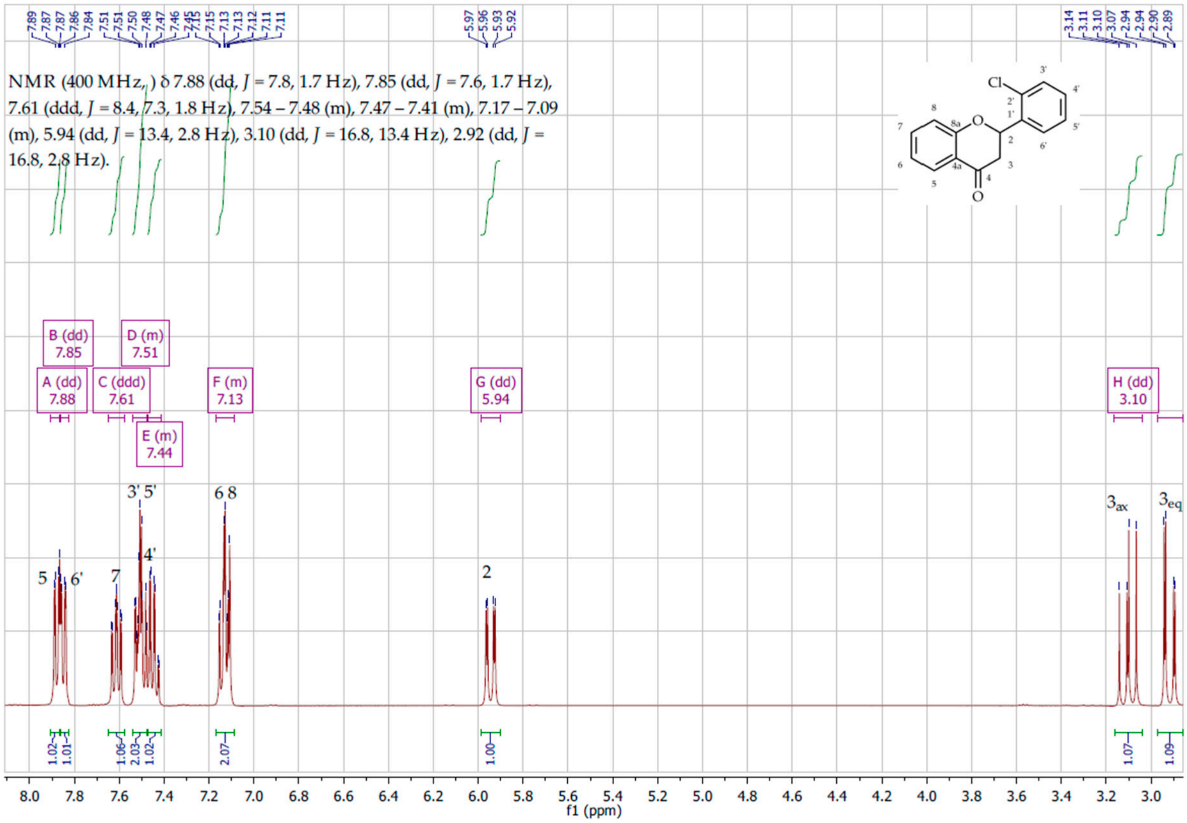

Figure S3. ^{1}H NMR spectrum expansion (*δ*, acetone-d6, 600 MHz) of 2′-chloroflavanone (**1**).

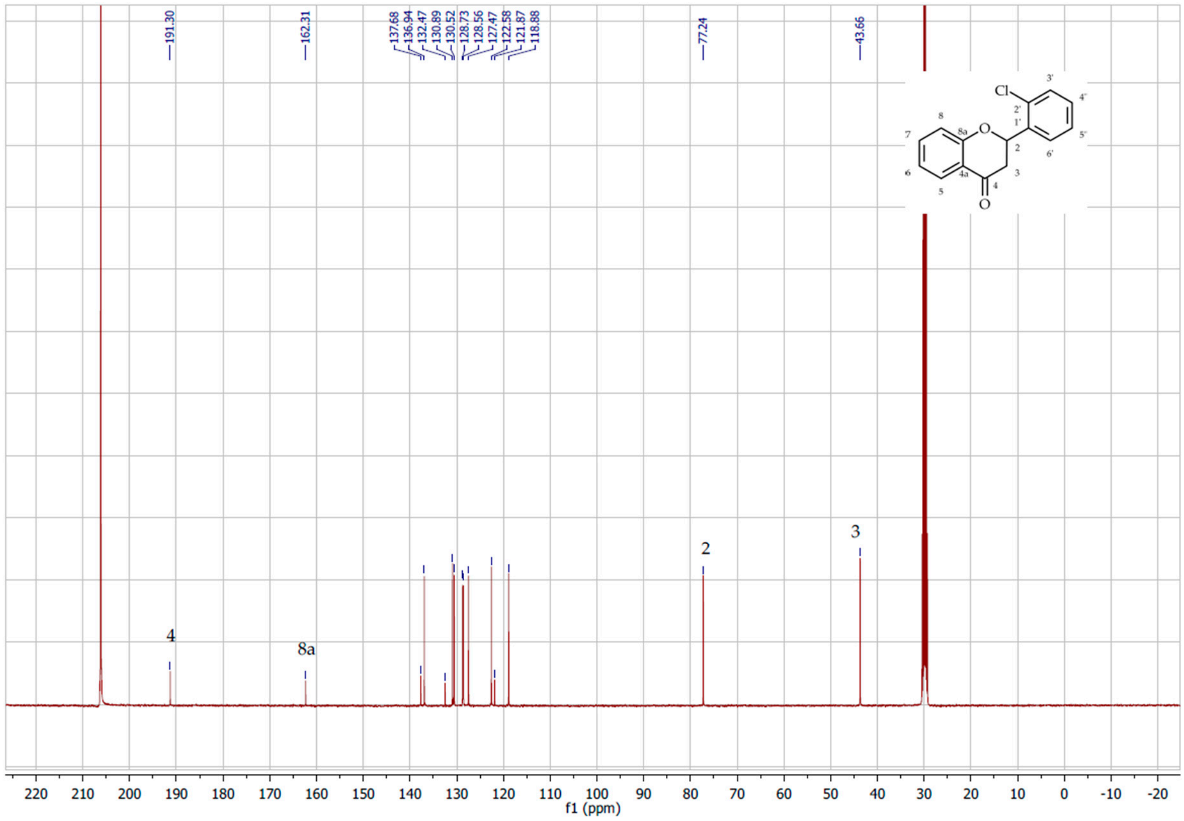

Figure S4. ^{13}C NMR spectrum (*δ*, acetone-d6, 151 MHz) of 2′-chloroflavanone (**1**).

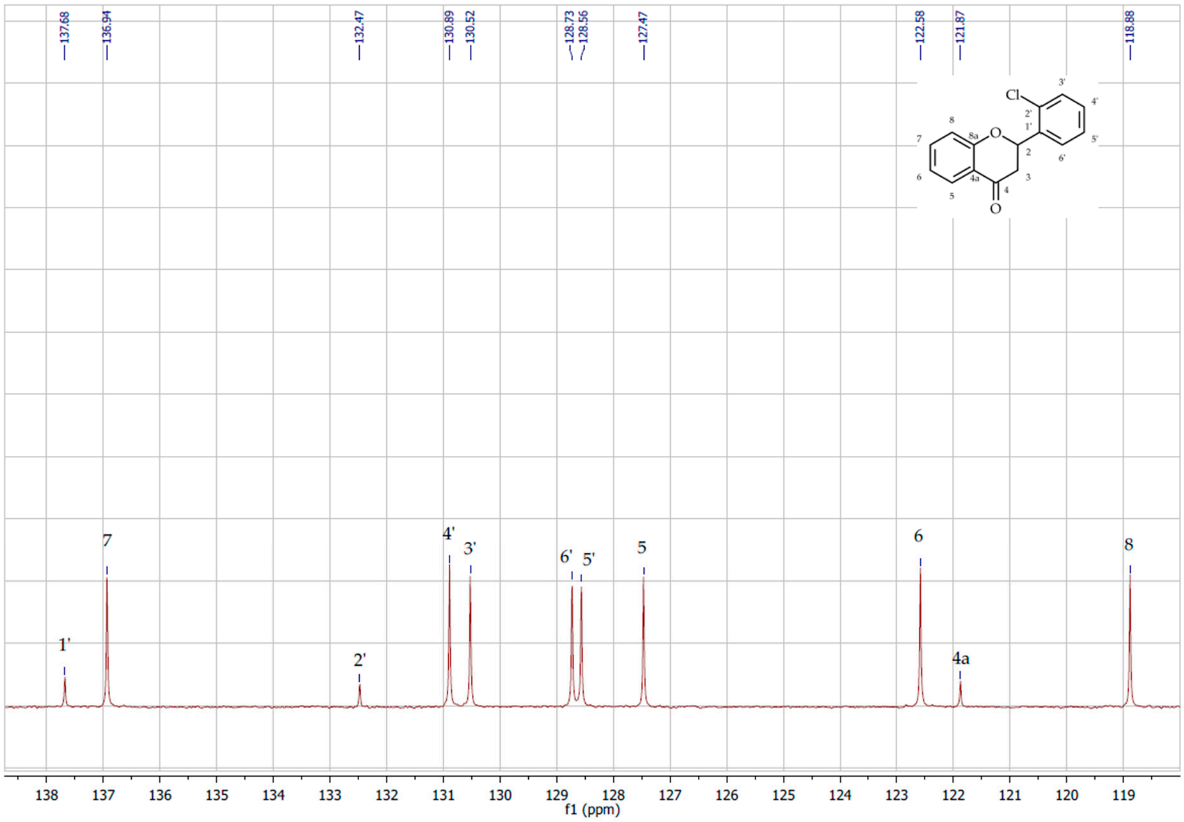

Figure S5. ^{13}C NMR spectrum expansion (*δ*, acetone-d6, 151 MHz) of 2′-chloroflavanone (**1**).

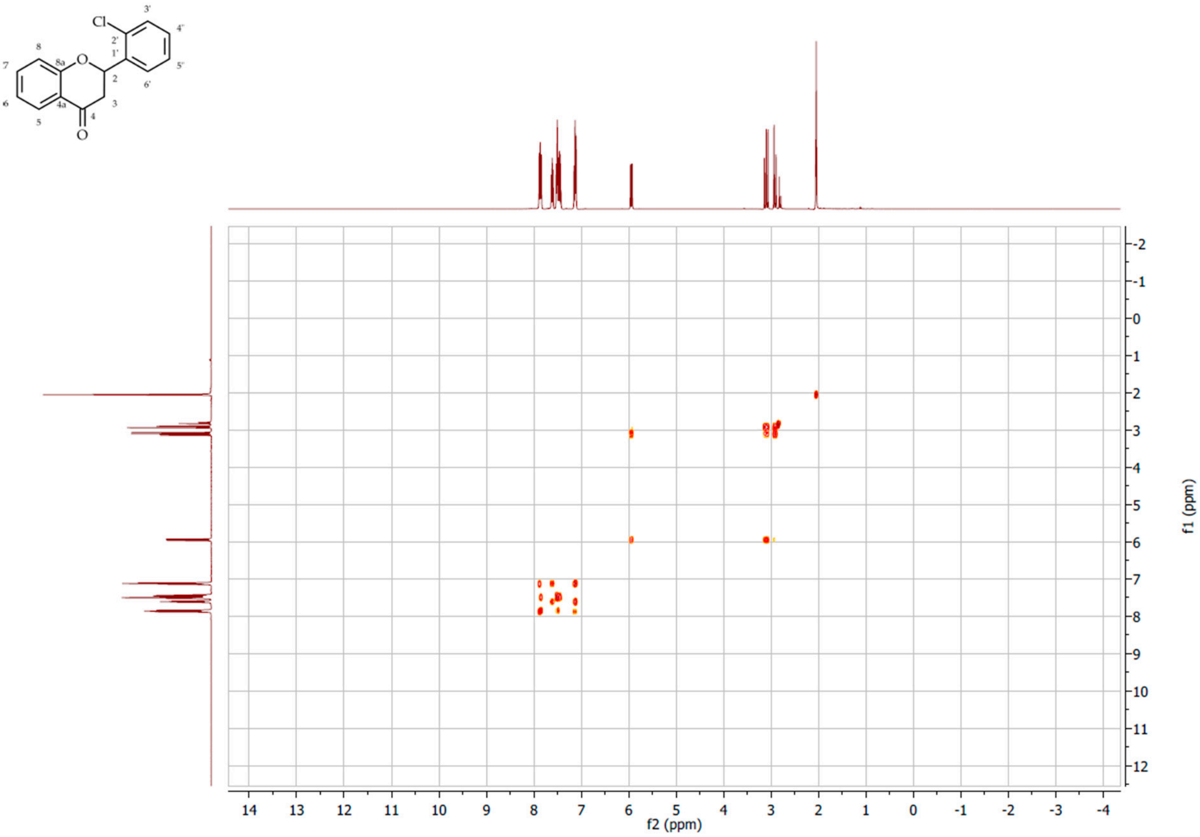

Figure S6. COSY contour map – ^{1}H x ^{1}H of 2′-chloroflavanone (**1**).

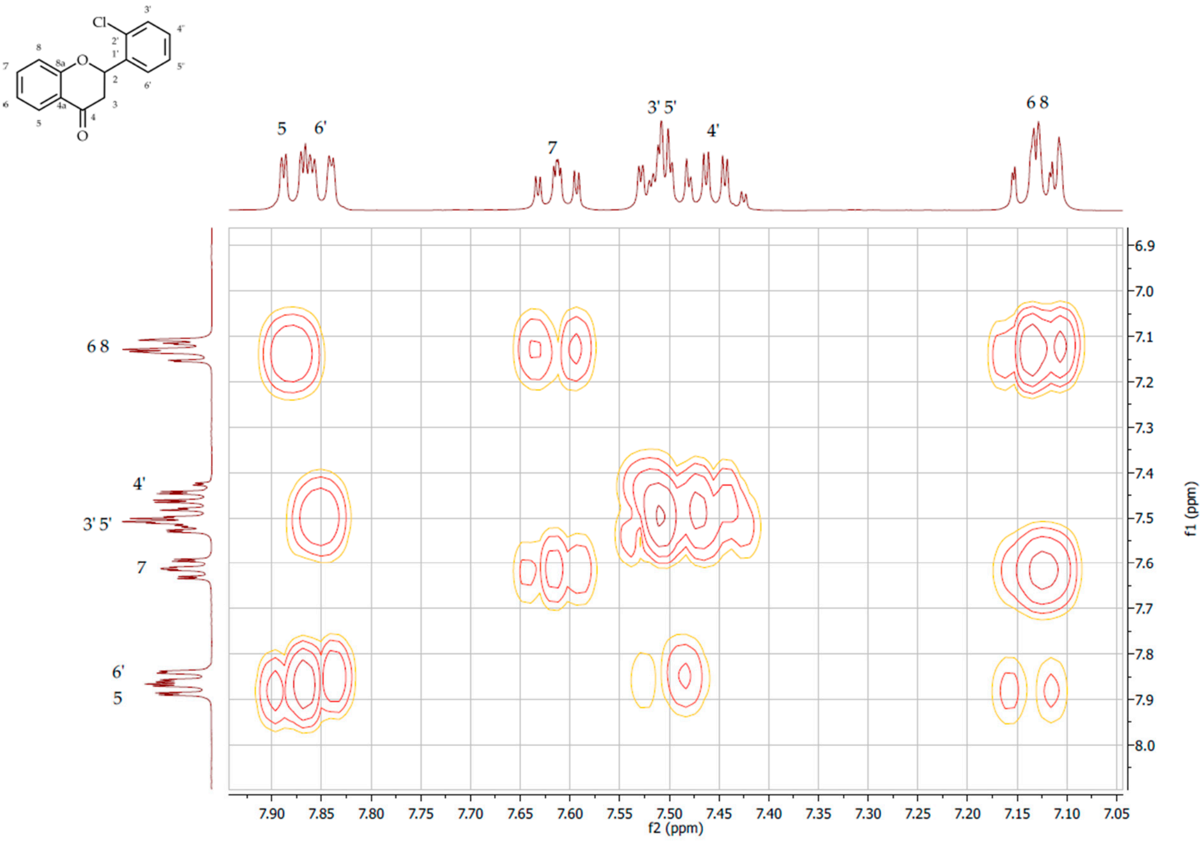

Figure S7. COSY contour map – ^{1}H x ^{1}H expansion of 2′-chloroflavanone (**1**).

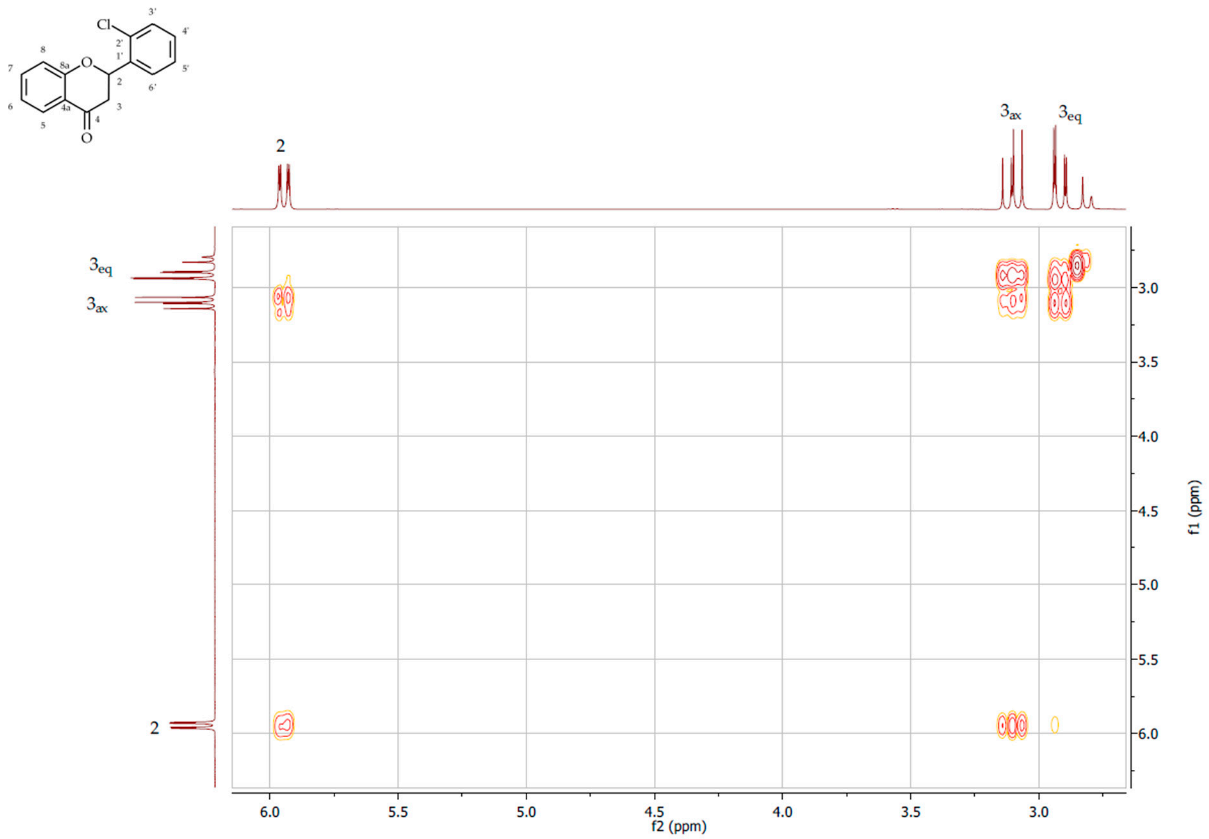

Figure S8. COSY contour map – ^{1}H x ^{1}H expansion of 2′-chloroflavanone (**1**).

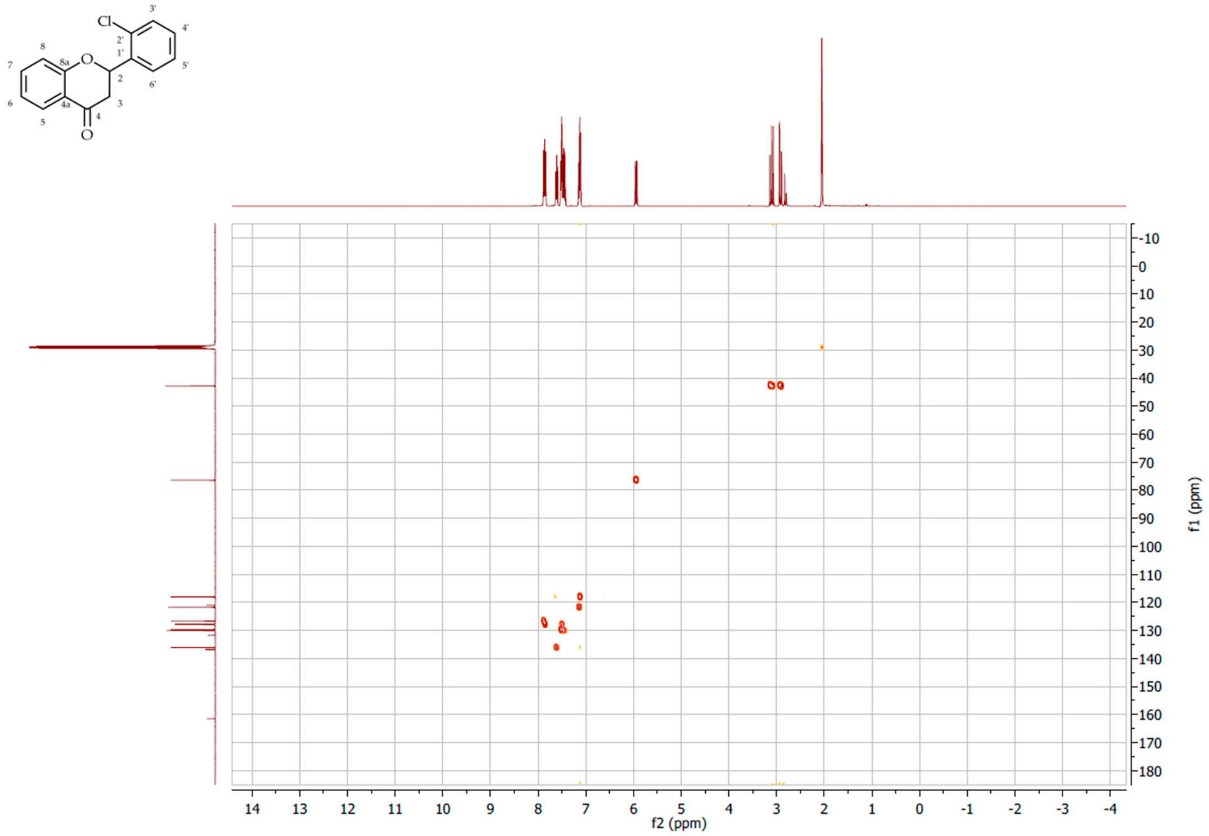

Figure S9. HMQC contour map – ^{1}H x ^{13}C of 2′-chloroflavanone (**1**).

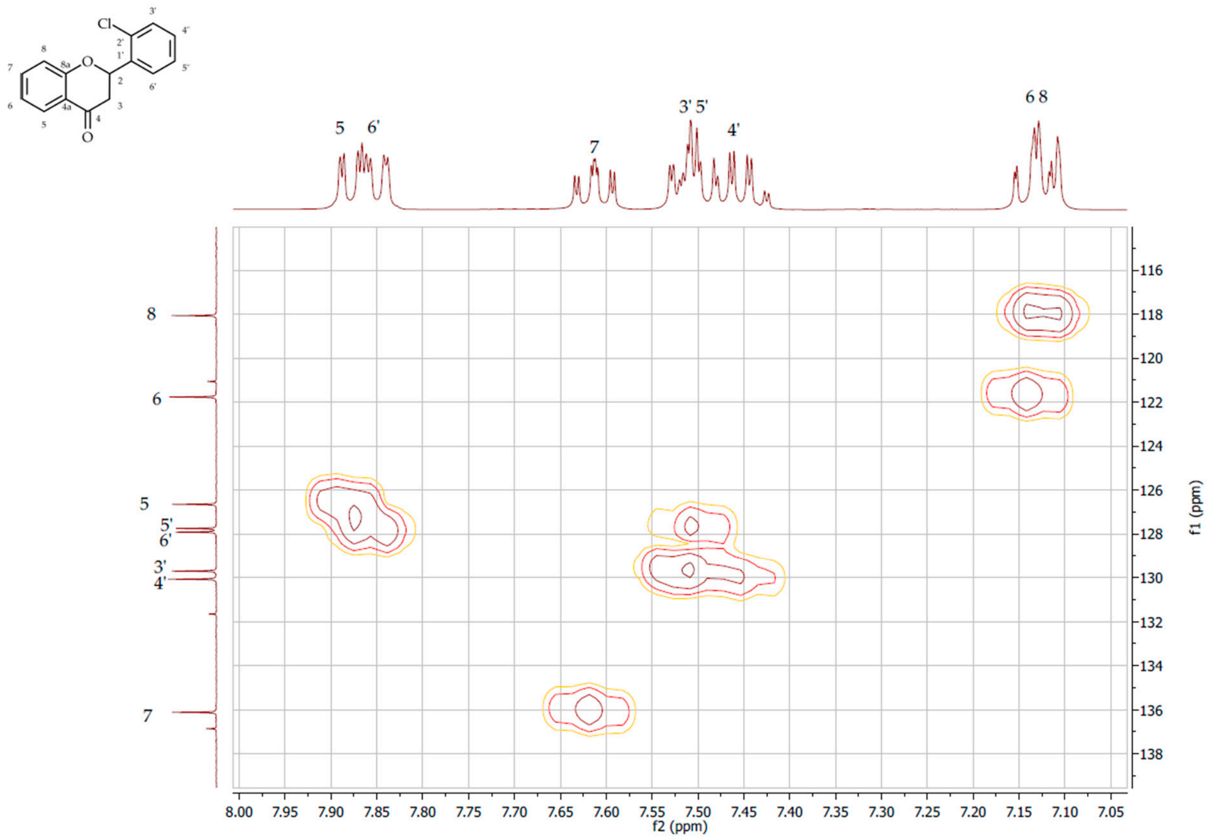

Figure S10. HMQC contour map – ^{1}H x ^{13}C expansion of 2′-chloroflavanone (**1**).

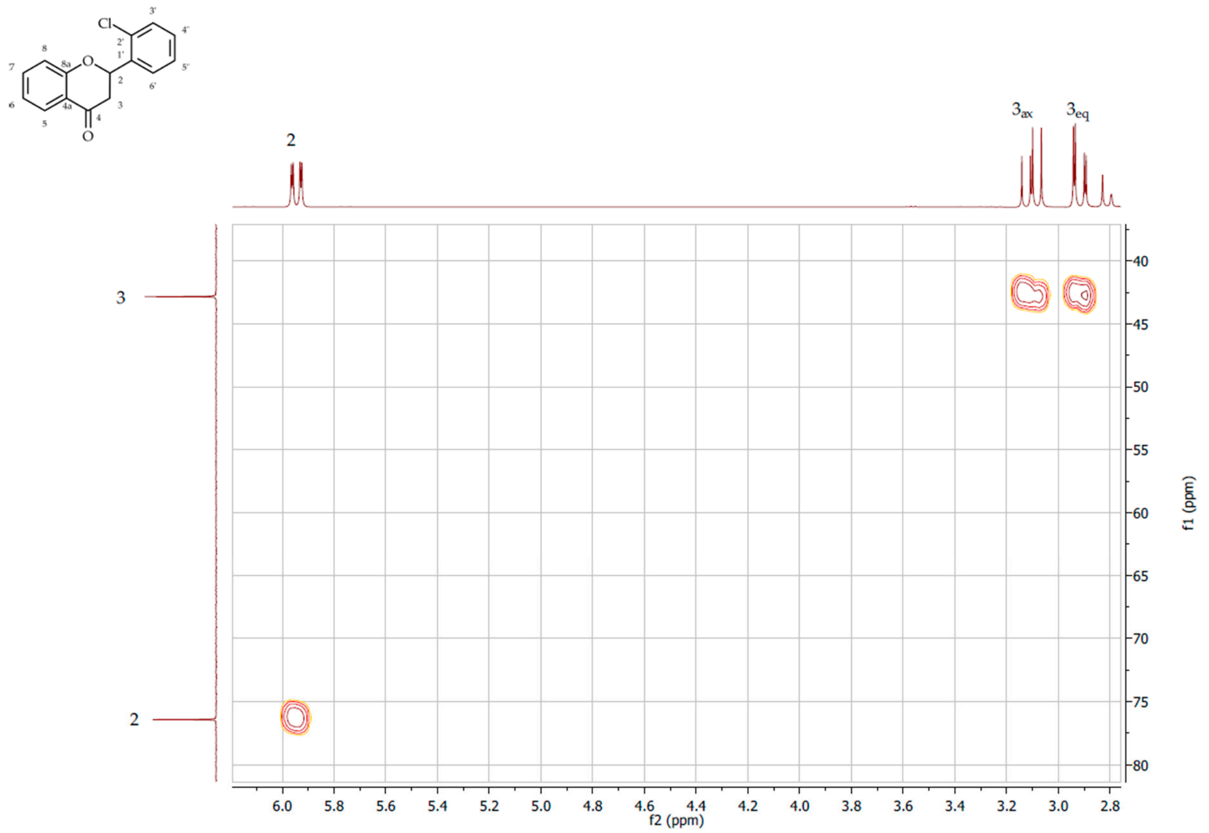

Figure S11. HMQC contour map – ^{1}H x ^{13}C expansion of 2′-chloroflavanone (**1**).

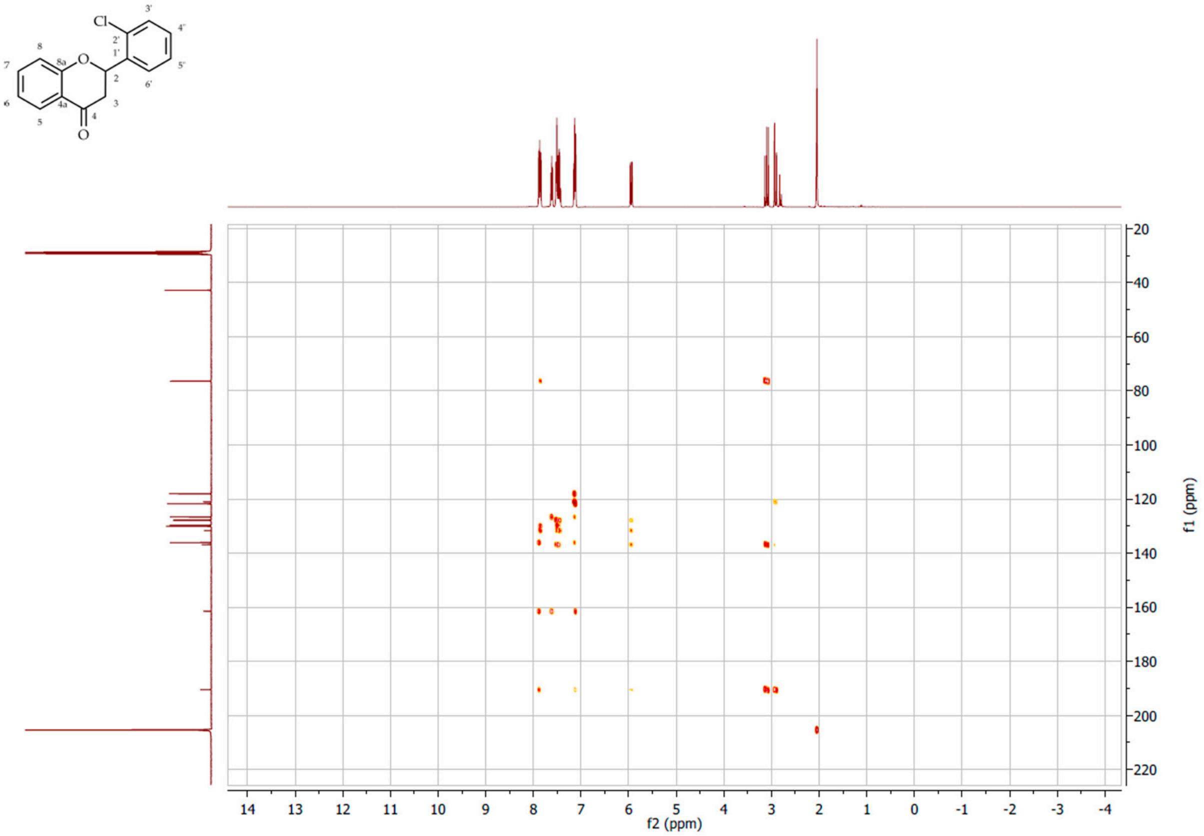

Figure S12. HMBC contour map – ^{1}H x ^{13}C of 2′-chloroflavanone (**1**).

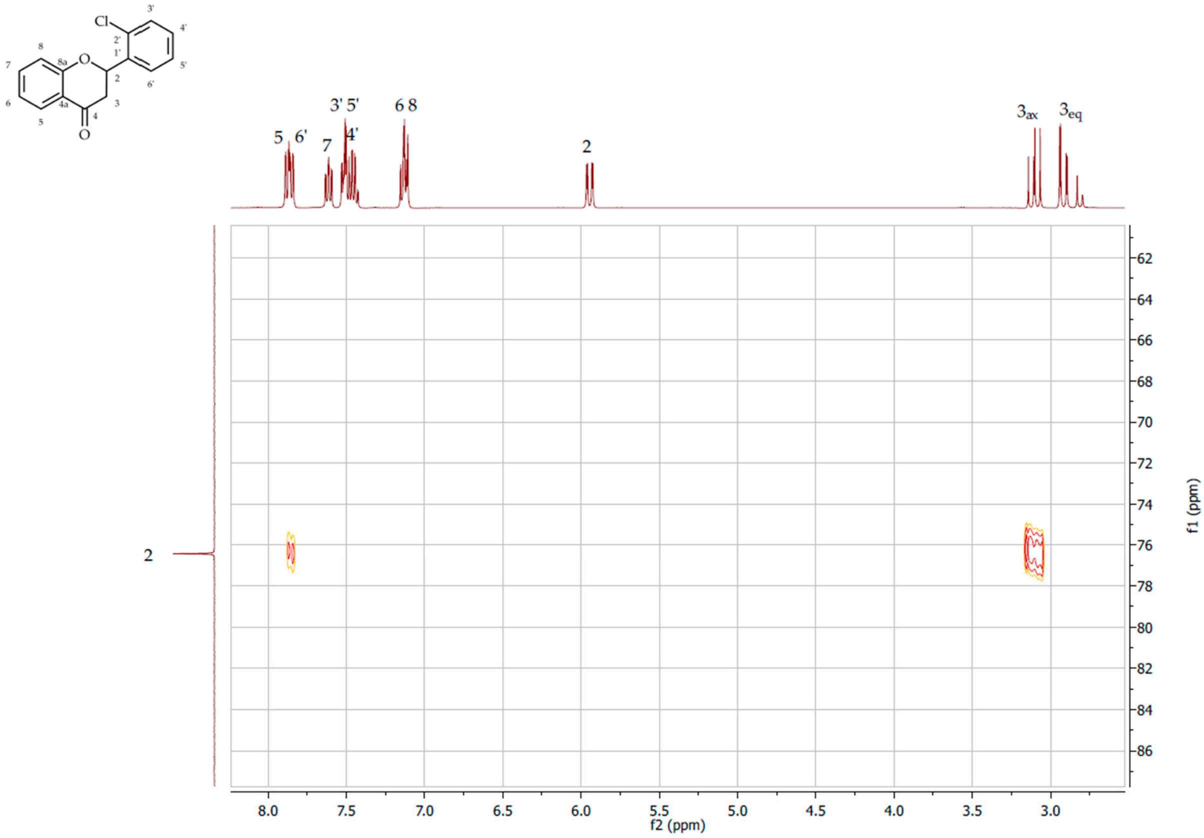

Figure S13. HMBC contour map – ^{1}H x ^{13}C expansion of 2′-chloroflavanone (**1**).

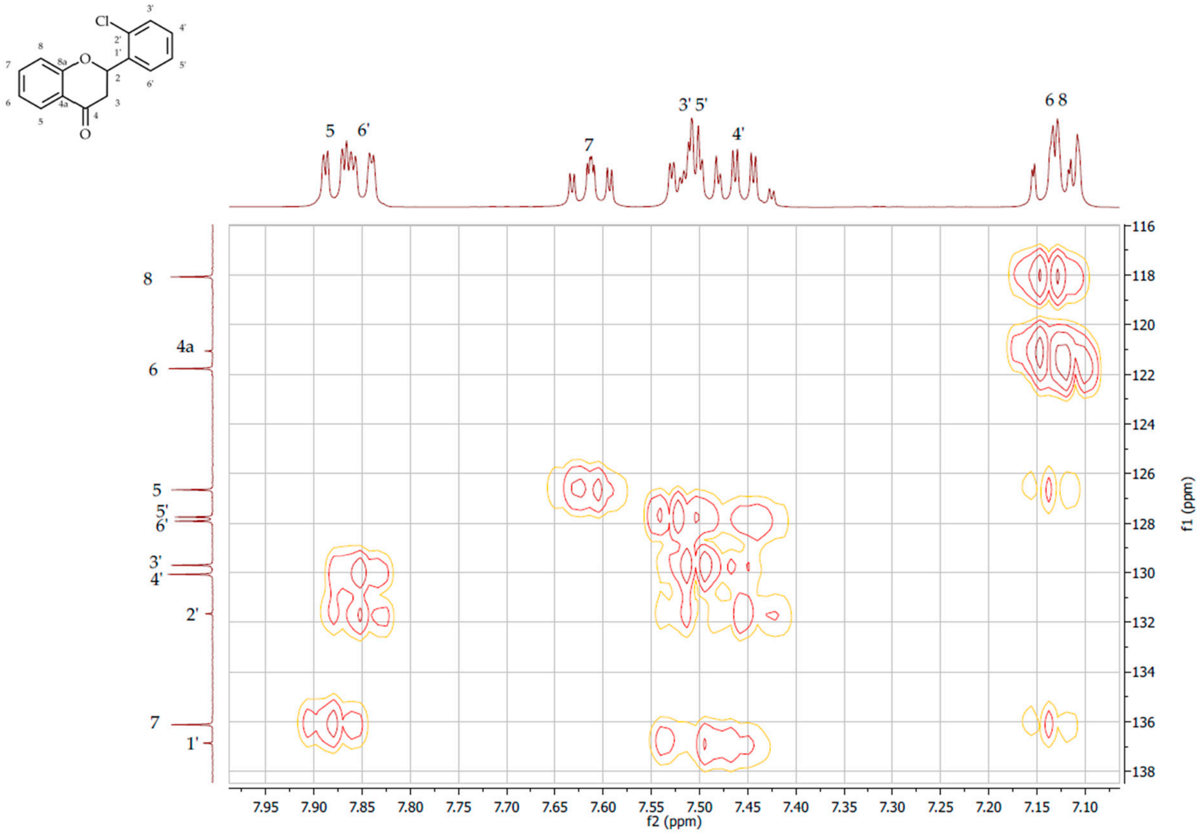

Figure S14. HMBC contour map – ^{1}H x ^{13}C expansion of 2′-chloroflavanone (**1**).

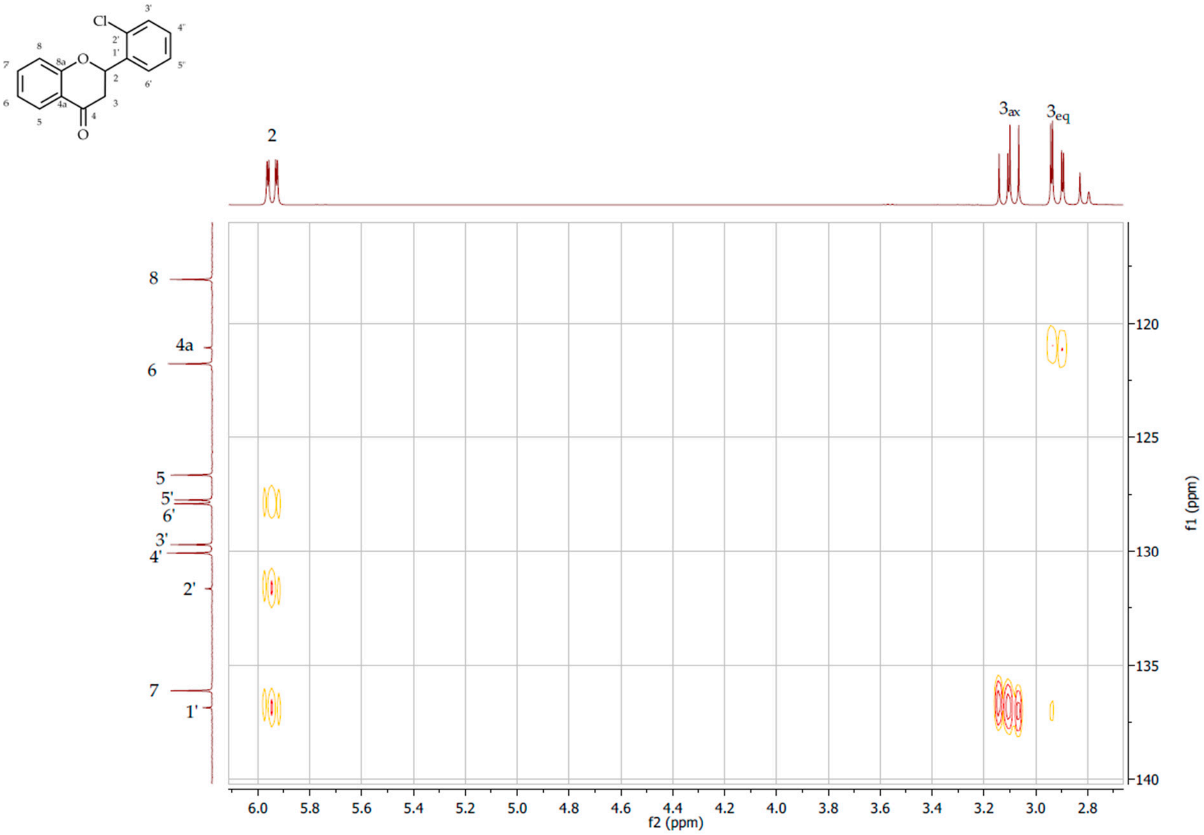

Figure S15. HMBC contour map – ^{1}H x ^{13}C expansion of 2′-chloroflavanone (**1**).

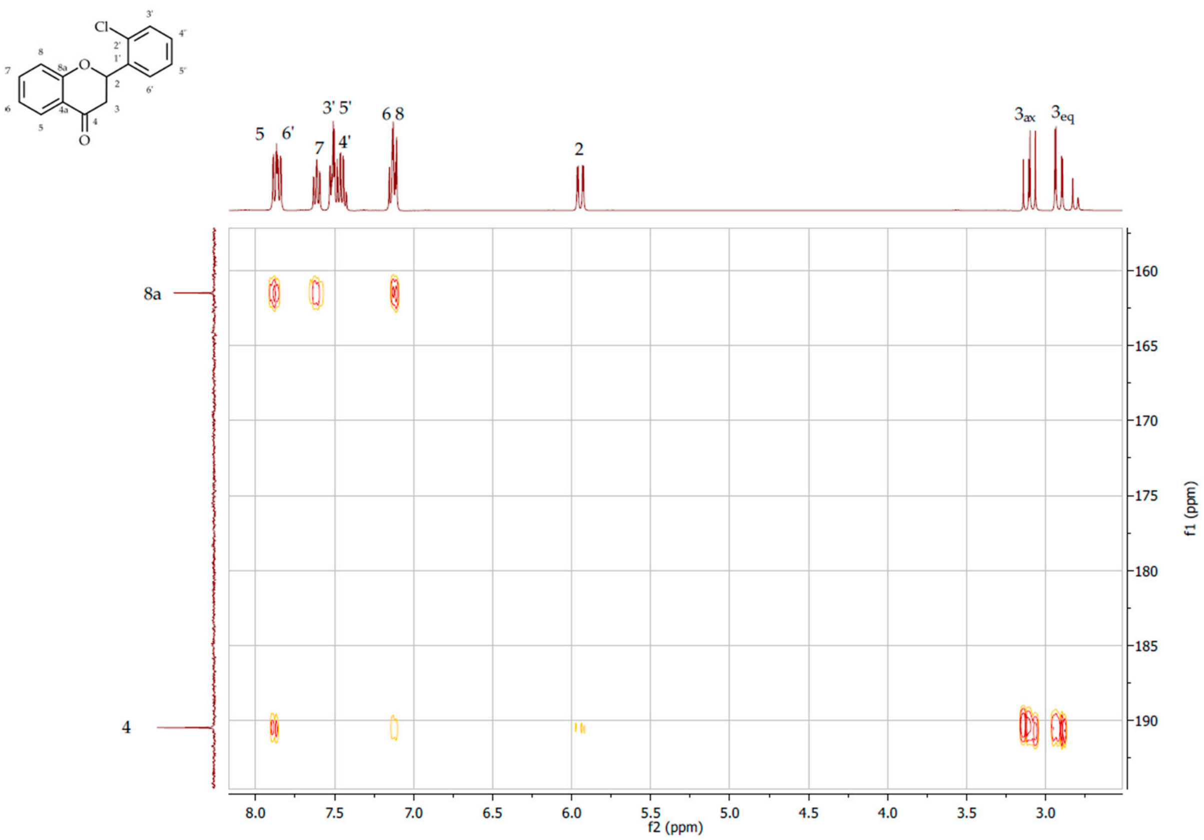

Figure S16. HMBC contour map – ^{1}H x ^{13}C expansion of 2′-chloroflavanone (**1**).

Molecule 1

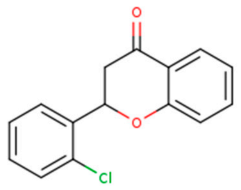

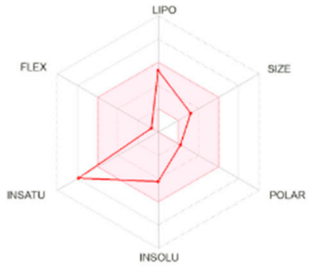

SMILES Clc1ccccc1C1CC(=O)c2c(O1)cccc2

Physicochemical Properties	
Formula	C15H11ClO2
Molecular weight	258.70 g/mol
Num. heavy atoms	18
Num. arom. heavy atoms	12
Fraction Csp3	0.13
Num. rotatable bonds	1
Num. H-bond acceptors	2
Num. H-bond donors	0
Molar Refractivity	70.51
TPSA	26.30 Å²

Lipophilicity	
Log $P_{o/w}$ (iLOGP)	2.63
Log $P_{o/w}$ (XLOGP3)	3.77
Log $P_{o/w}$ (WLOGP)	3.72
Log $P_{o/w}$ (MLOGP)	2.98
Log $P_{o/w}$ (SILICOS-IT)	4.18
Consensus Log $P_{o/w}$	3.46

Water Solubility	
Log *S* (ESOL)	-4.25
Solubility	1.47e-02 mg/ml ; 5.67e-05 mol/l
Class	Moderately soluble
Log *S* (Ali)	-4.02
Solubility	2.50e-02 mg/ml ; 9.65e-05 mol/l
Class	Moderately soluble
Log *S* (SILICOS-IT)	-5.76
Solubility	4.48e-04 mg/ml ; 1.73e-06 mol/l
Class	Moderately soluble

Pharmacokinetics	
GI absorption	High
BBB permeant	Yes
P-gp substrate	No
CYP1A2 inhibitor	Yes
CYP2C19 inhibitor	Yes
CYP2C9 inhibitor	No
CYP2D6 inhibitor	No
CYP3A4 inhibitor	No
Log K_p (skin permeation)	-5.20 cm/s

Druglikeness	
Lipinski	Yes; 0 violation
Ghose	Yes
Veber	Yes
Egan	Yes
Muegge	Yes
Bioavailability Score	0.55

Medicinal Chemistry	
PAINS	0 alert
Brenk	0 alert
Leadlikeness	No; 1 violation: XLOGP3>3.5
Synthetic accessibility	2.90

Figure S17. 2′-Chloroflavanone (**1**) physicochemical and ADME parameters prediction using the SwissADME modelling.

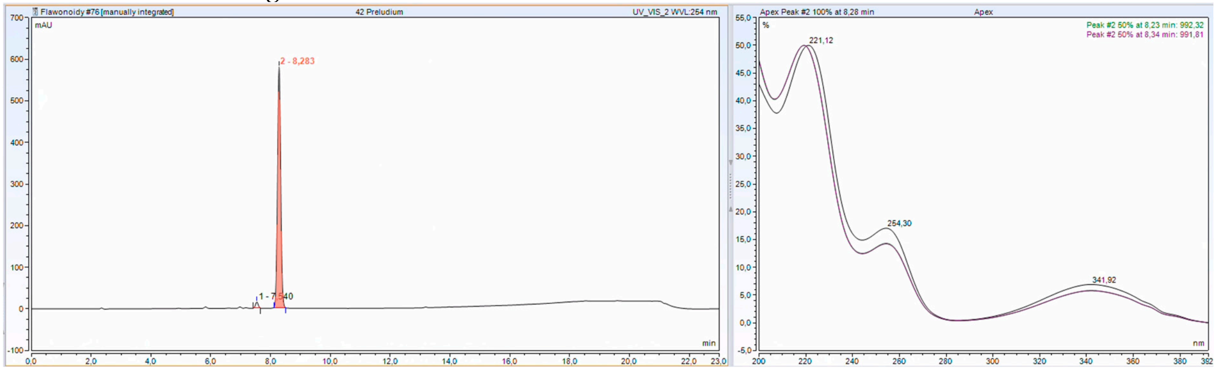

Figure S18. HPLC analysis of 2′-chloroflavanone 6-*O*-*β*-D-(4′′-*O*-methyl)-glucopyranoside (**1a**).

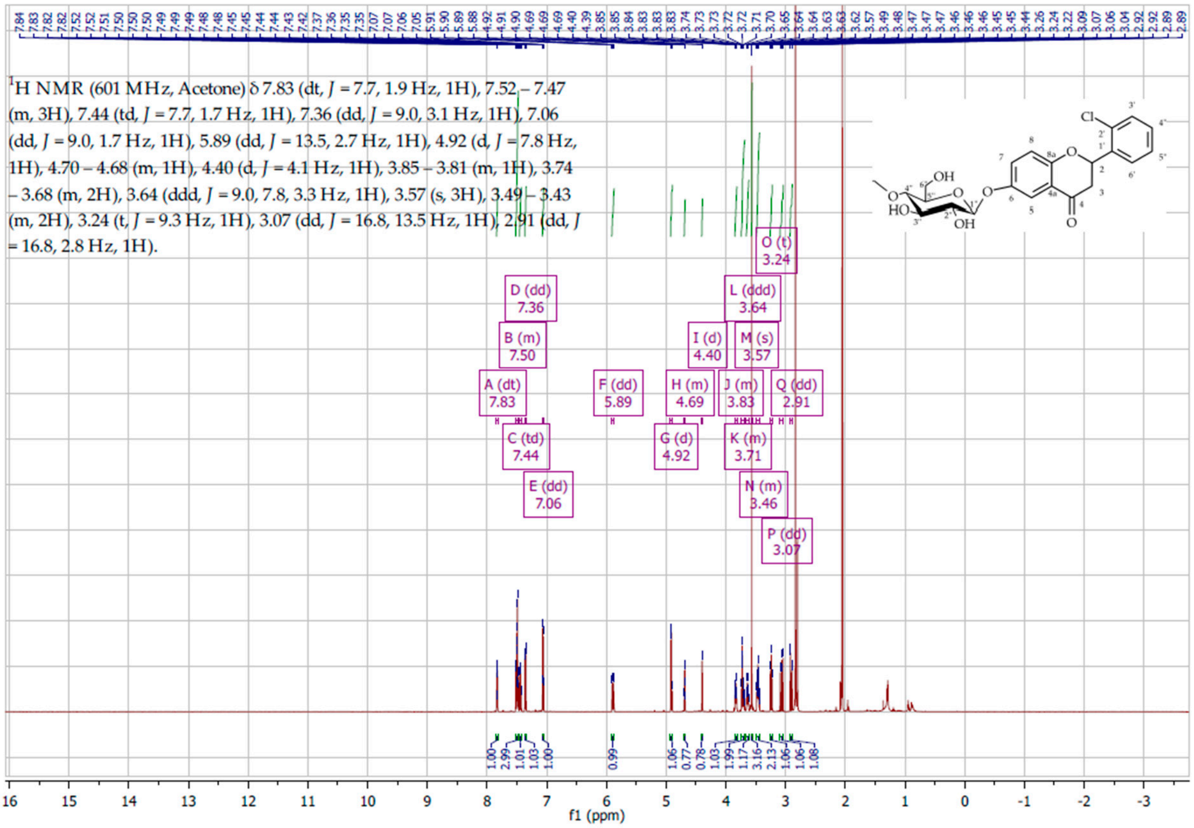

Figure S19. ^{1}H NMR spectrum (*δ*, acetone-d6, 600 MHz) of 2′-chloroflavanone 6-*O*-*β*-D-(4′′-*O*-methyl)-glucopyranoside (**1a**).

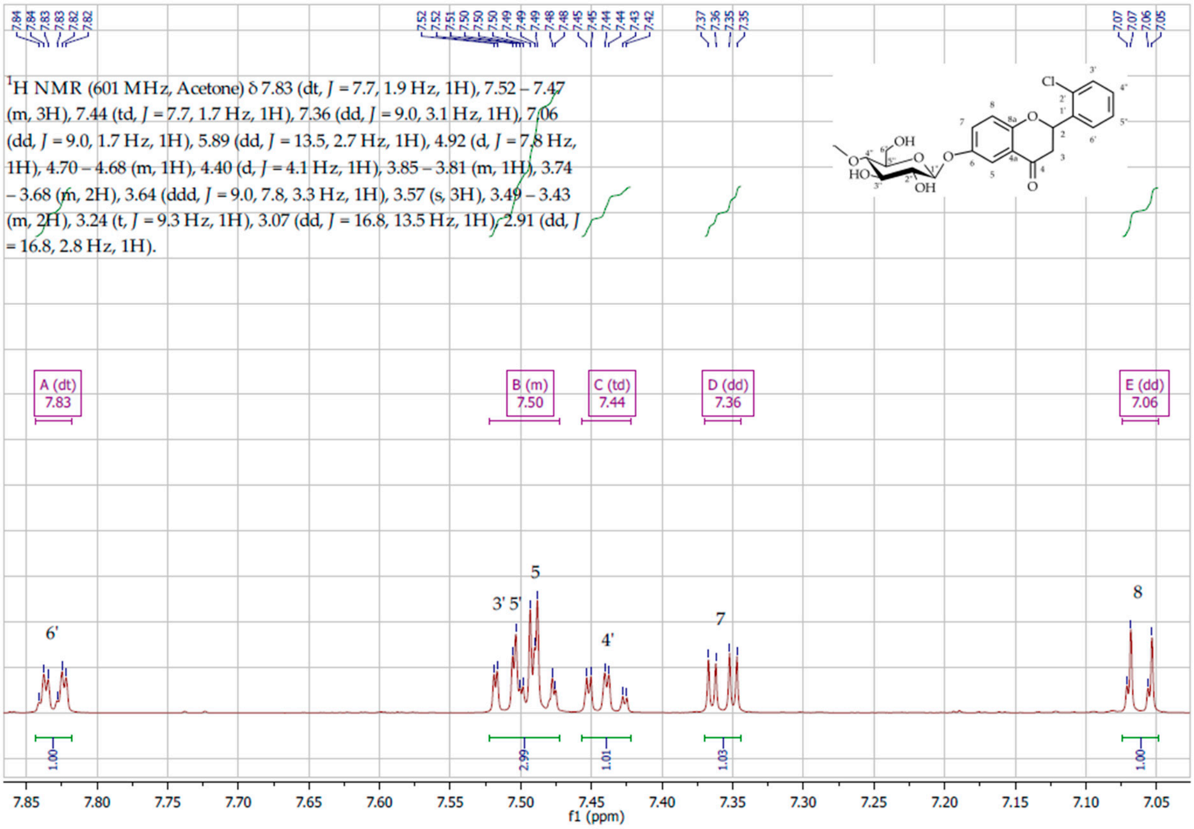

Figure S20. ^{1}H NMR spectrum expansion (*δ*, acetone-d6, 600 MHz) of 2′-chloroflavanone 6-*O*-*β*-D-(4′′-*O*-methyl)-glucopyranoside (**1a**).

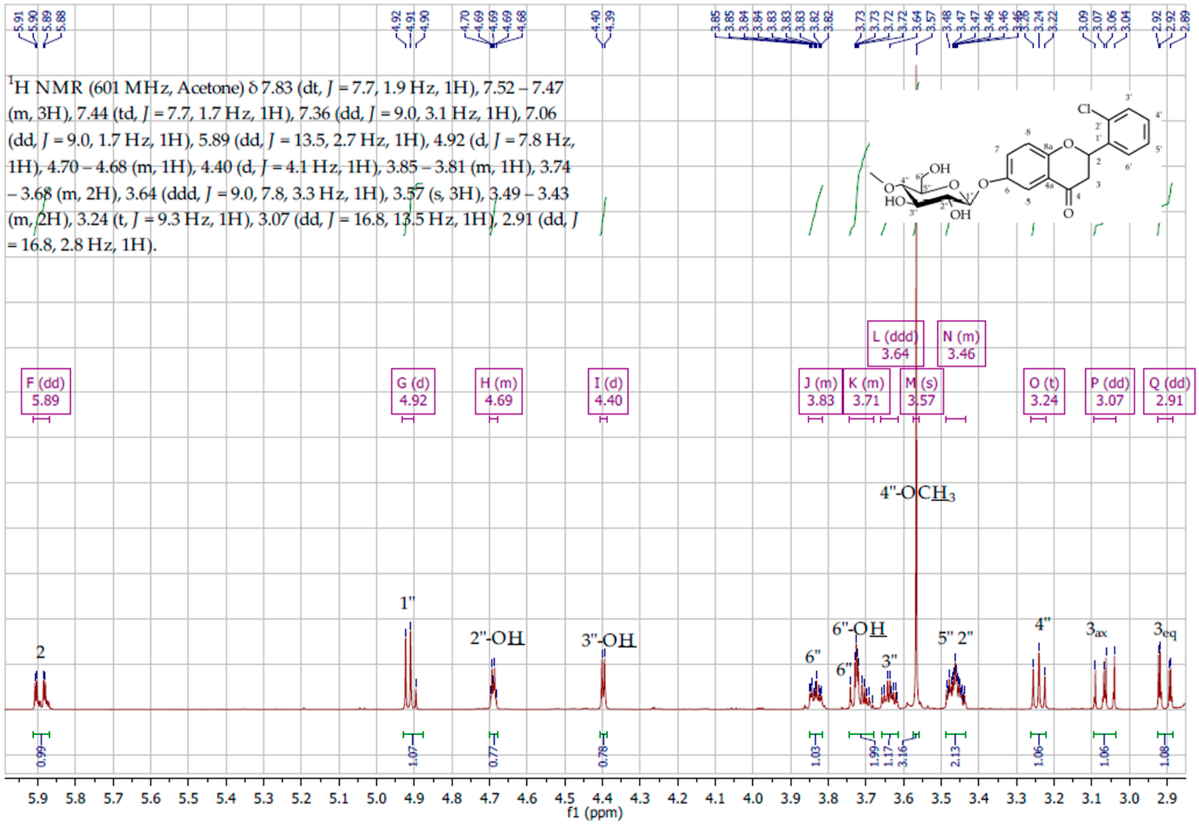

Figure S21. ^{1}H NMR spectrum expansion (*δ*, acetone-d6, 600 MHz) of 2′-chloroflavanone 6-*O*-*β*-D-(4′′-*O*-methyl)-glucopyranoside (**1a**).

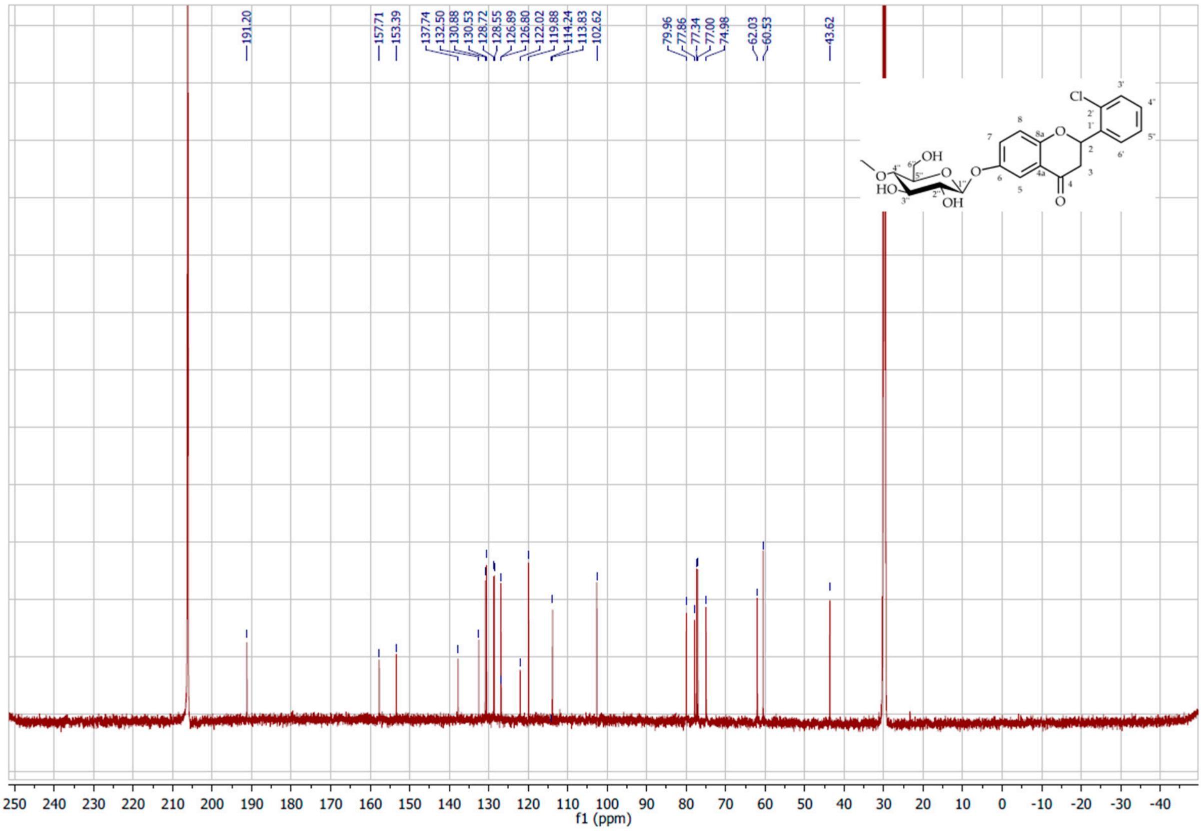

Figure S22. ^{13}C NMR spectrum (*δ*, acetone-d6, 151 MHz) of 2′-chloroflavanone 6-*O*-*β*-D-(4′′-*O*-methyl)-glucopyranoside (**1a**).

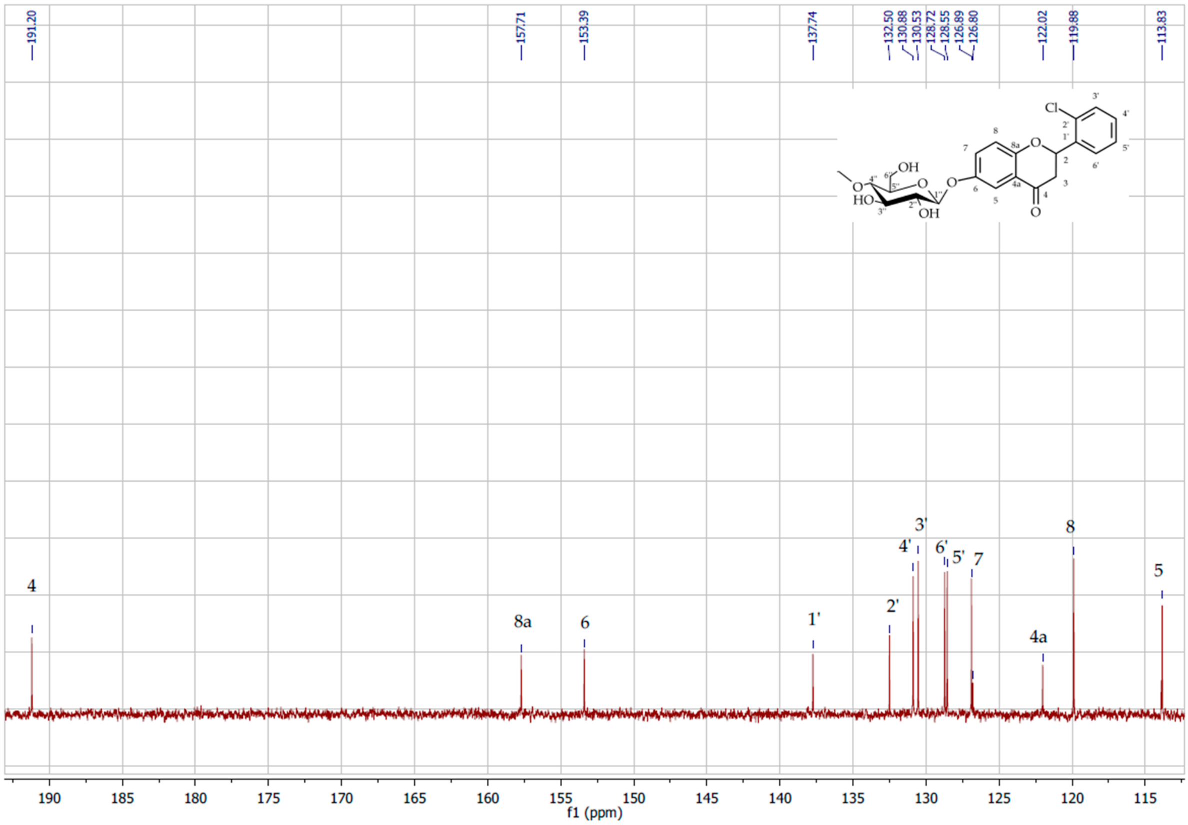

Figure S23. ^{13}C NMR spectrum expansion (*δ*, acetone-d6, 151 MHz) of 2′-chloroflavanone 6-*O*-*β*-D-(4′′-*O*-methyl)-glucopyranoside (**1a**).

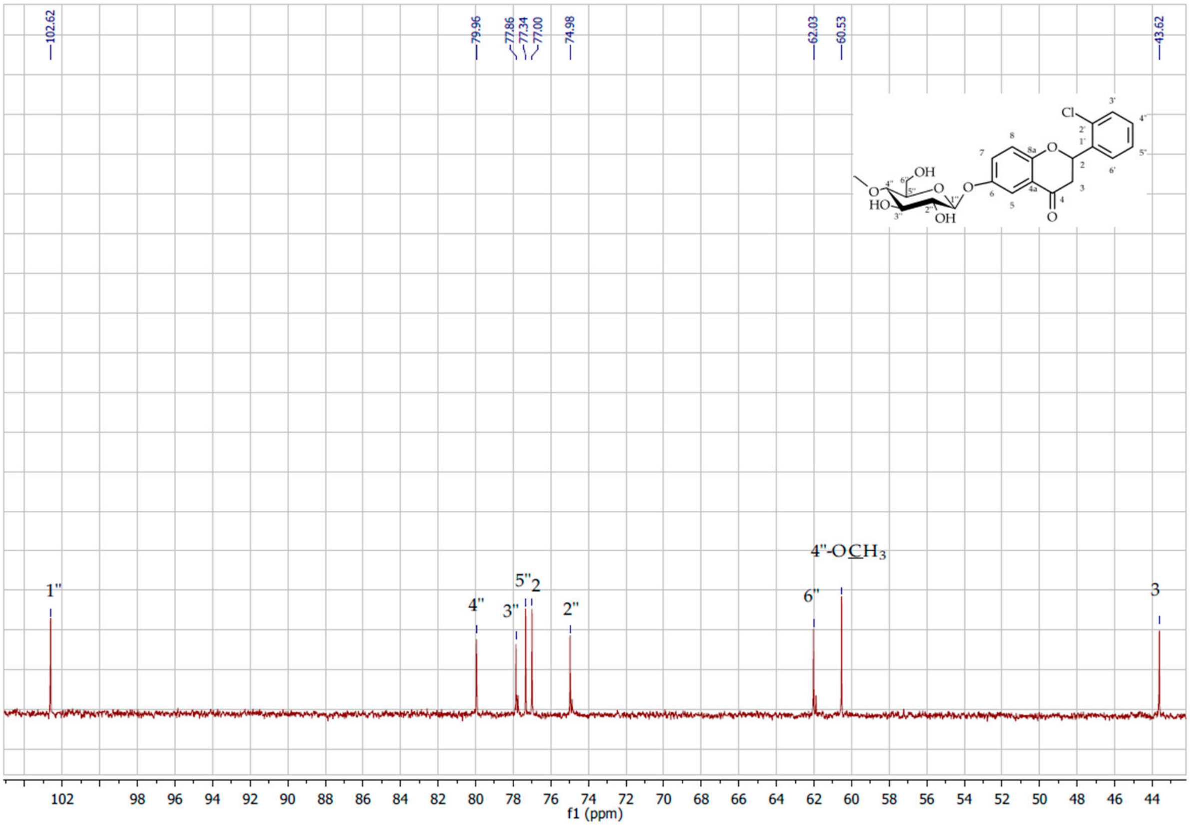

Figure S24. ^{13}C NMR spectrum expansion (*δ*, acetone-d6, 151 MHz) of 2′-chloroflavanone 6-*O*-*β*-D-(4′′-*O*-methyl)-glucopyranoside (**1a**).

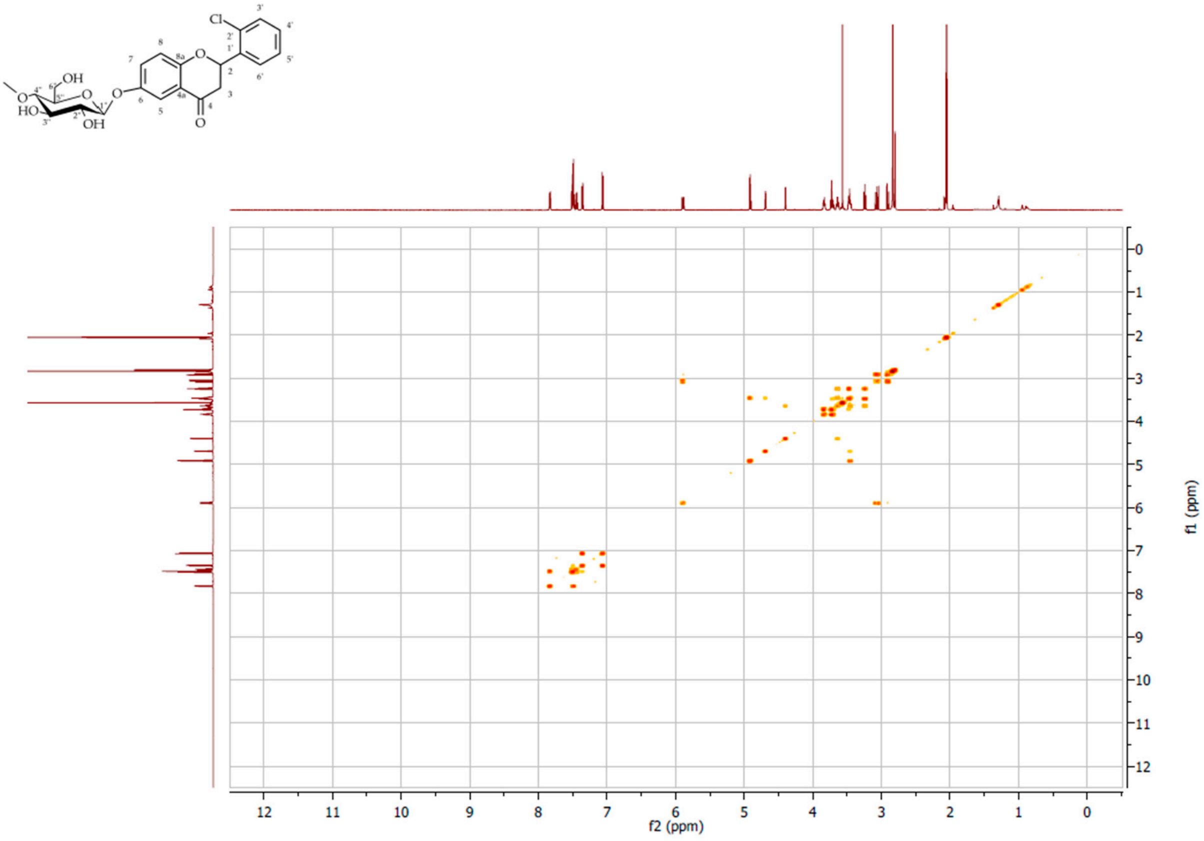

Figure S25. COSY contour map – ^1H x ^1H of 2′-chloroflavanone 6-*O*-*β*-D-(4′′-*O*-methyl)-glucopyranoside (**1a**).

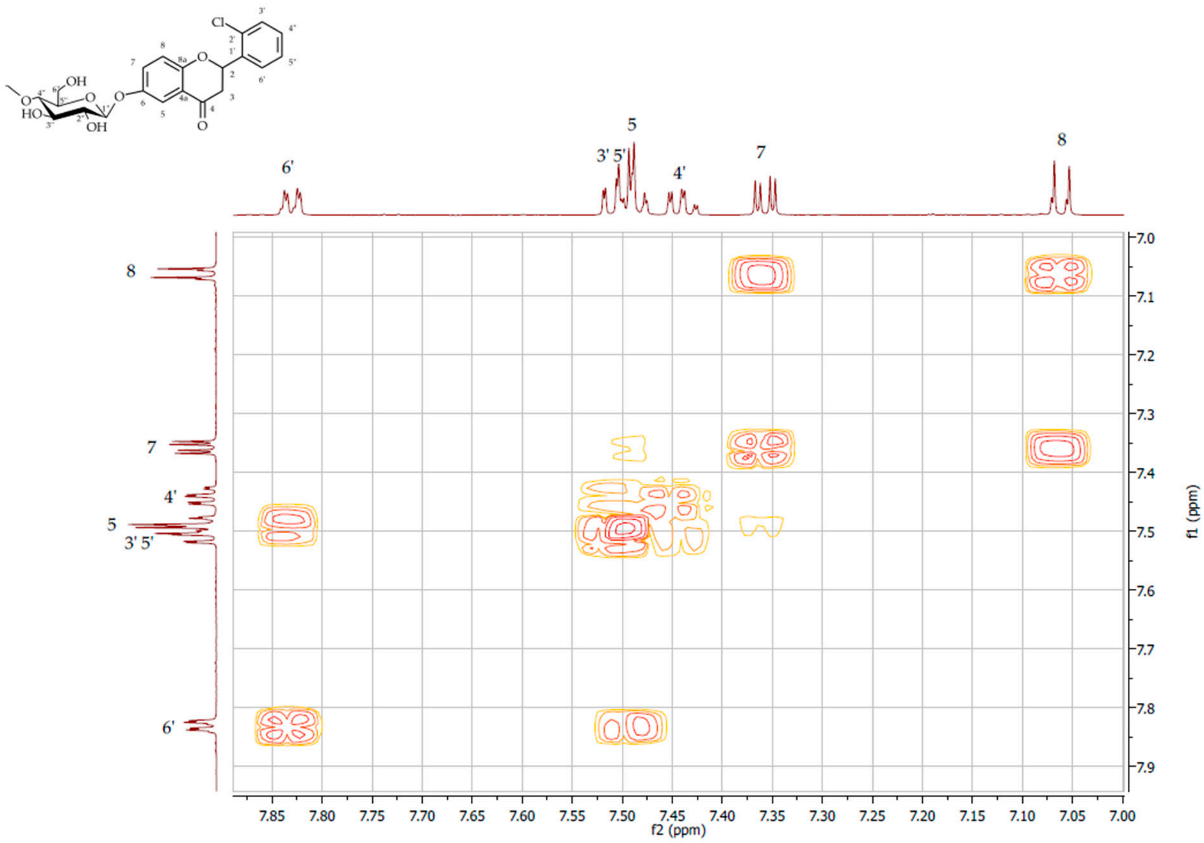

Figure S26. COSY contour map – $^1H \times ^1H$ expansion of 2′-chloroflavanone 6-*O*-*β*-D-(4′′-*O*-methyl)-glucopyranoside (**1a**).

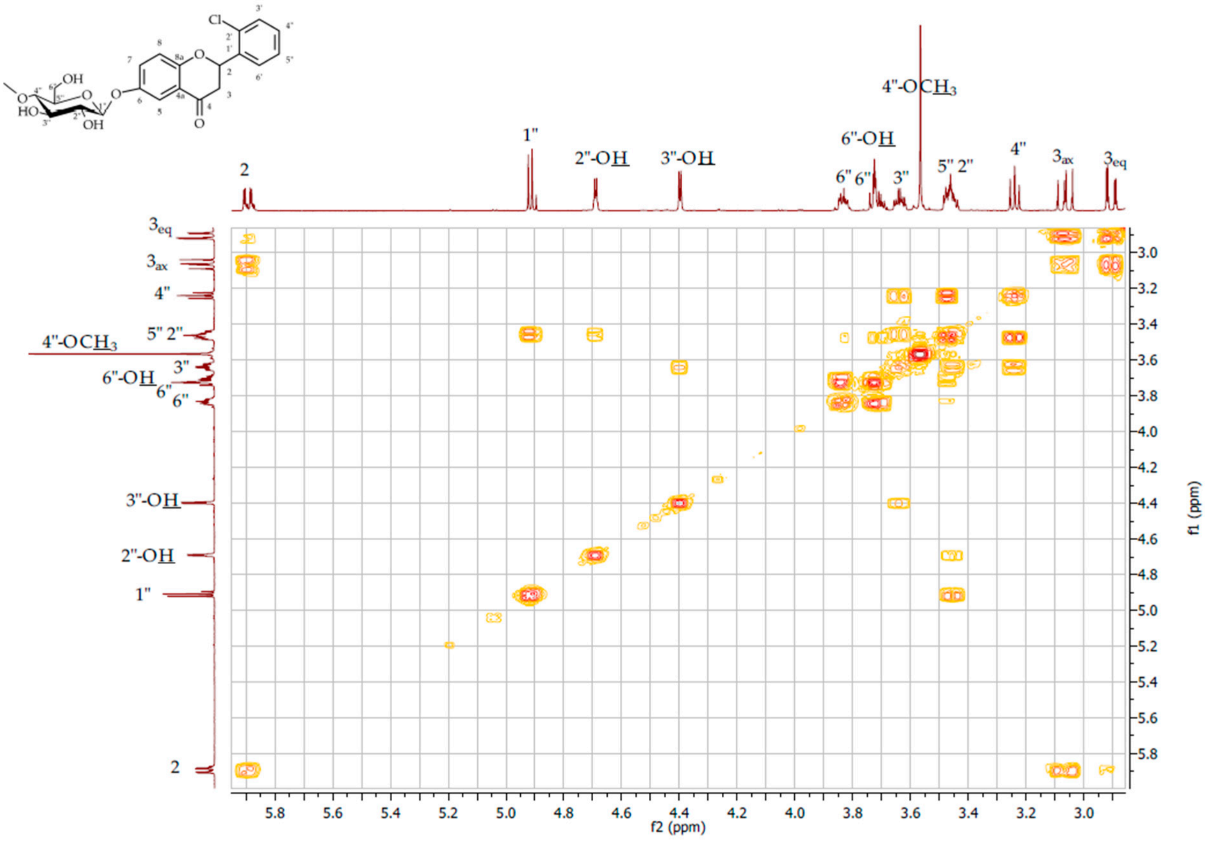

Figure S27. COSY contour map – $^1H \times ^1H$ expansion of 2′-chloroflavanone 6-*O*-*β*-D-(4′′-*O*-methyl)-glucopyranoside (**1a**).

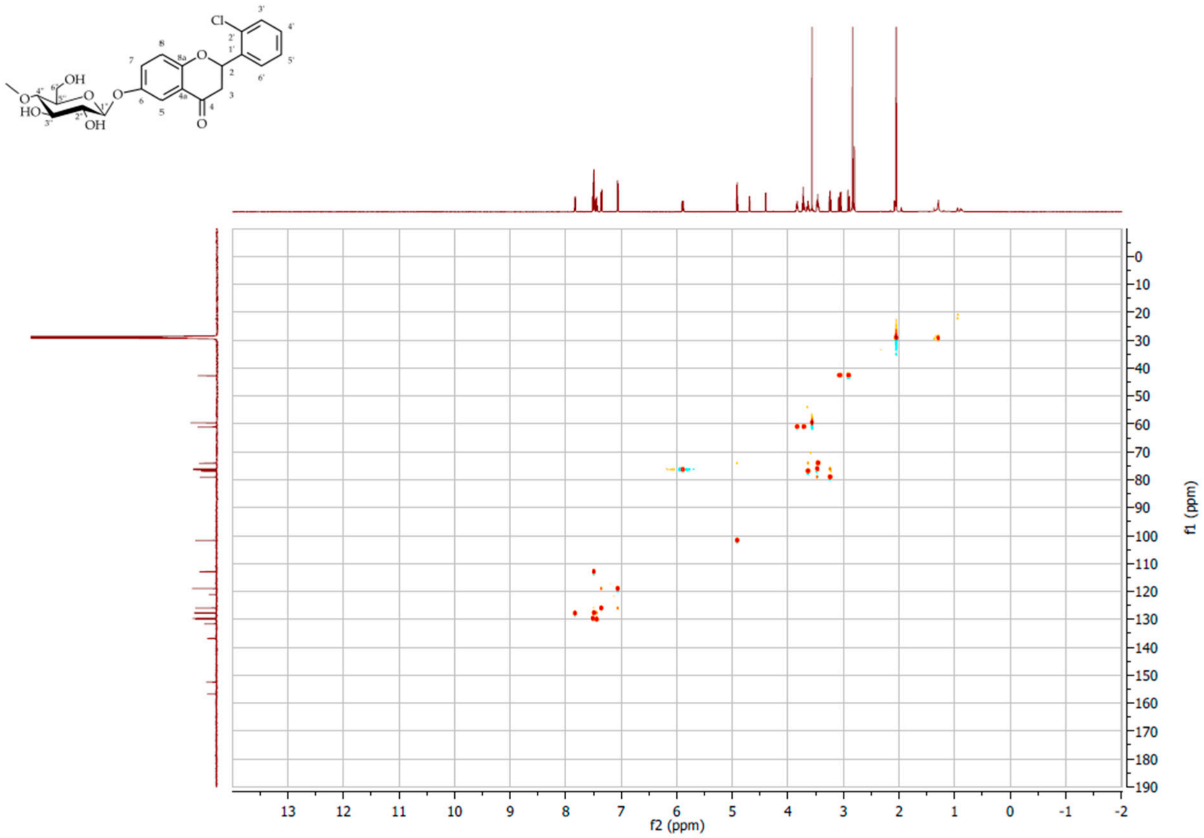

Figure S28. HMQC contour map – ¹H x ¹³C of 2′-chloroflavanone 6-*O*-*β*-D-(4′′-*O*-methyl)-glucopyranoside (**1a**).

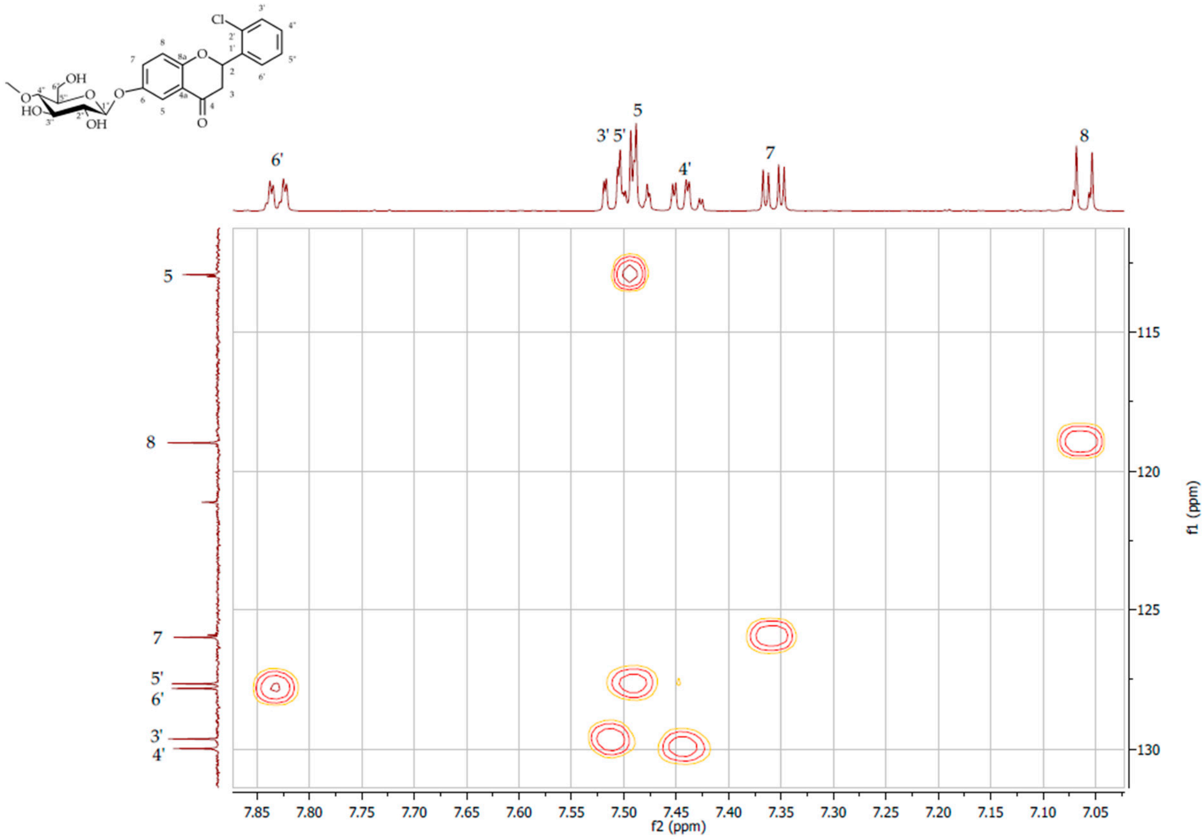

Figure S29. HMQC contour map – ¹H x ¹³C expansion of 2′-chloroflavanone 6-*O*-*β*-D-(4′′-*O*-methyl)-glucopyranoside (**1a**).

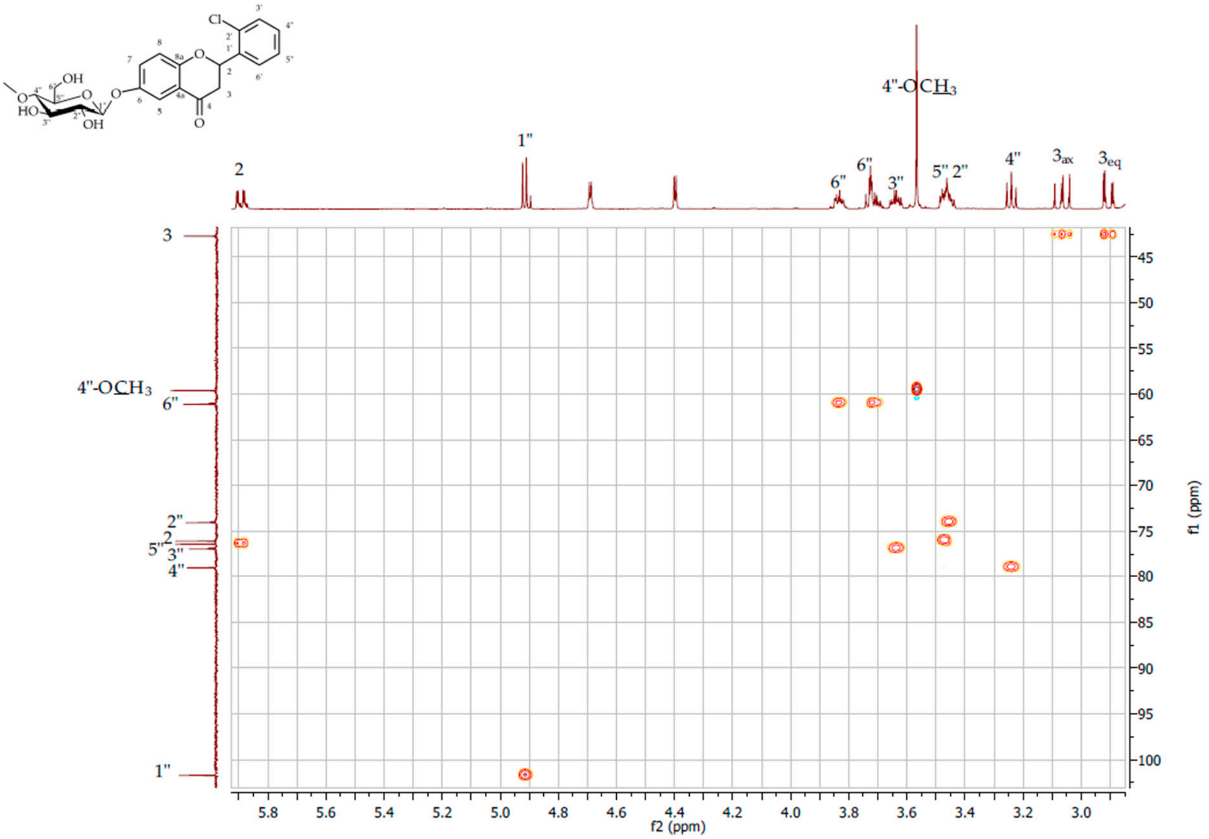

Figure S30. HMQC contour map – [1]H x ^{13}C expansion of 2′-chloroflavanone 6-*O*-*β*-D-(4′′-*O*-methyl)-glucopyranoside (**1a**).

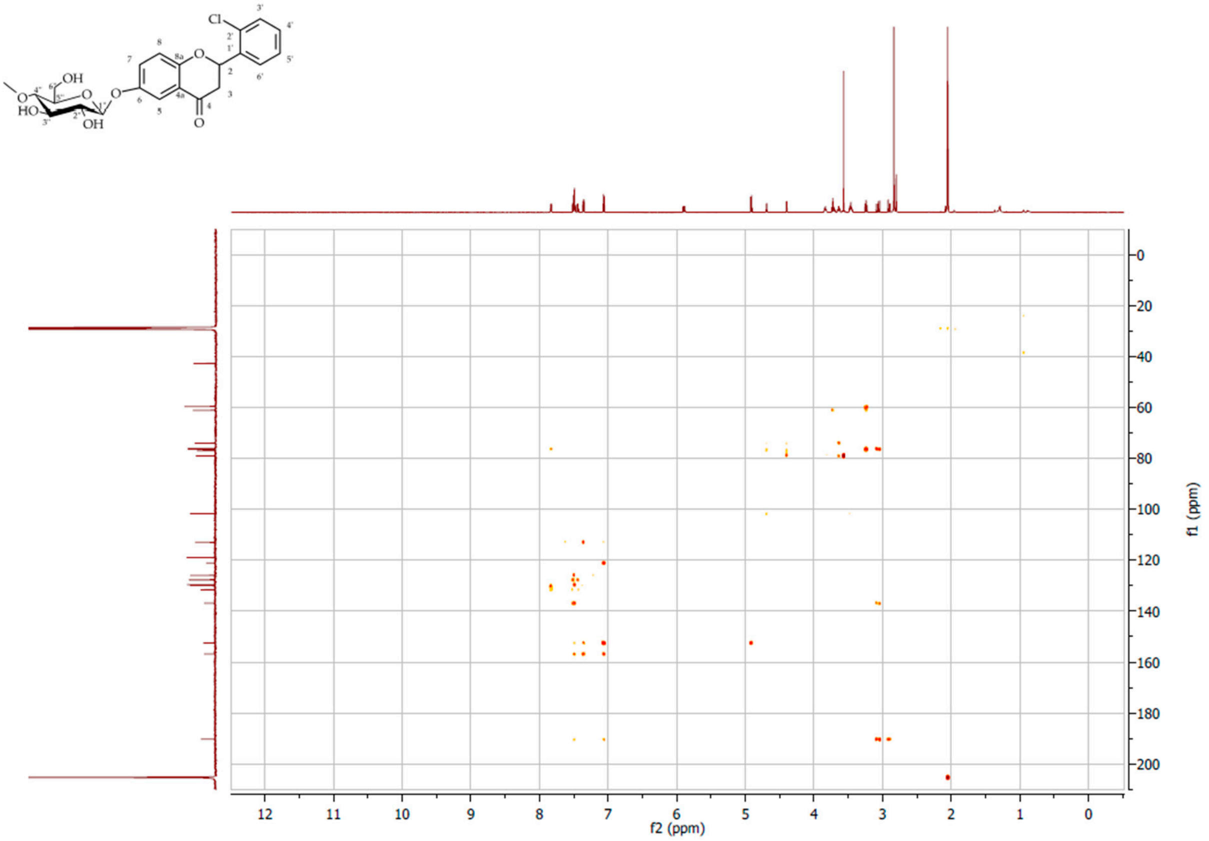

Figure S31. HMBC contour map – ^{1}H x ^{13}C of 2′-chloroflavanone 6-*O*-*β*-D-(4′′-*O*-methyl)-glucopyranoside (**1a**).

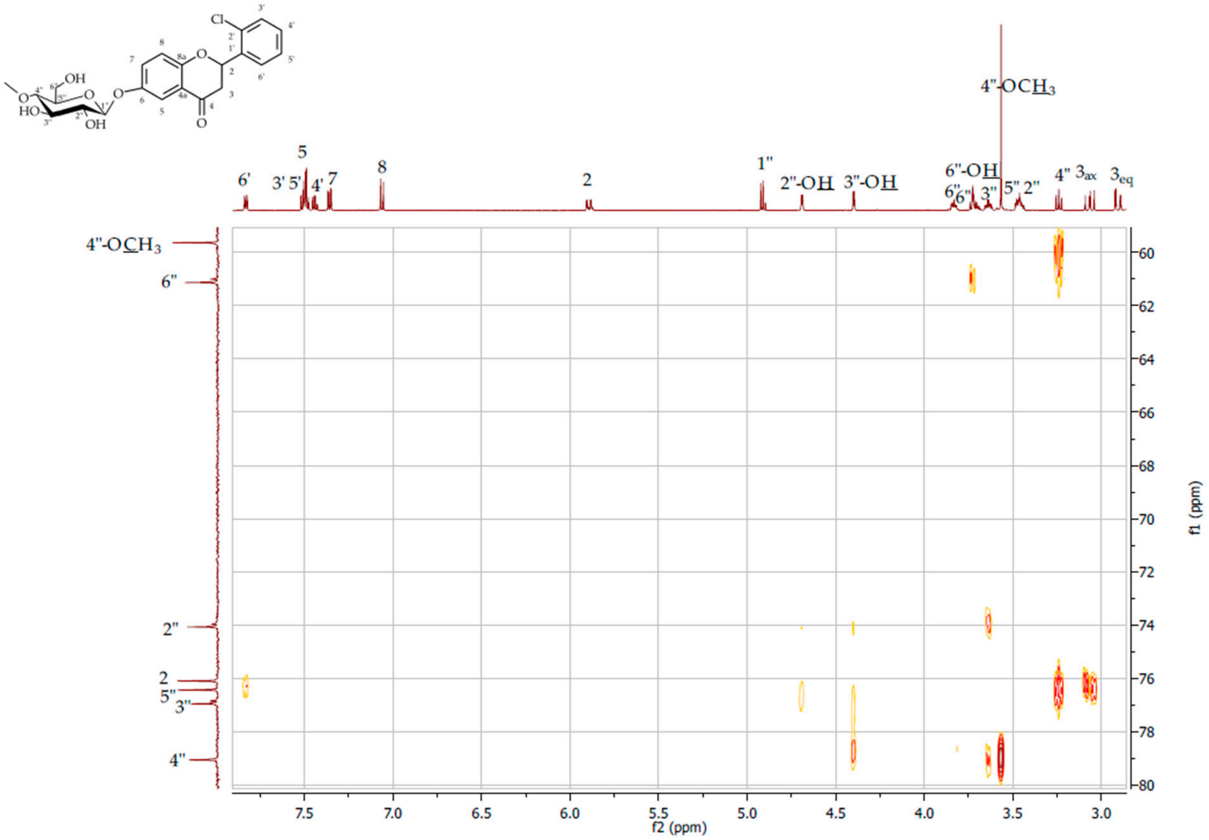

Figure S32. HMBC contour map – ^{1}H x ^{13}C expansion of 2′-chloroflavanone 6-*O*-*β*-D-(4′′-*O*-methyl)-glucopyranoside (**1a**).

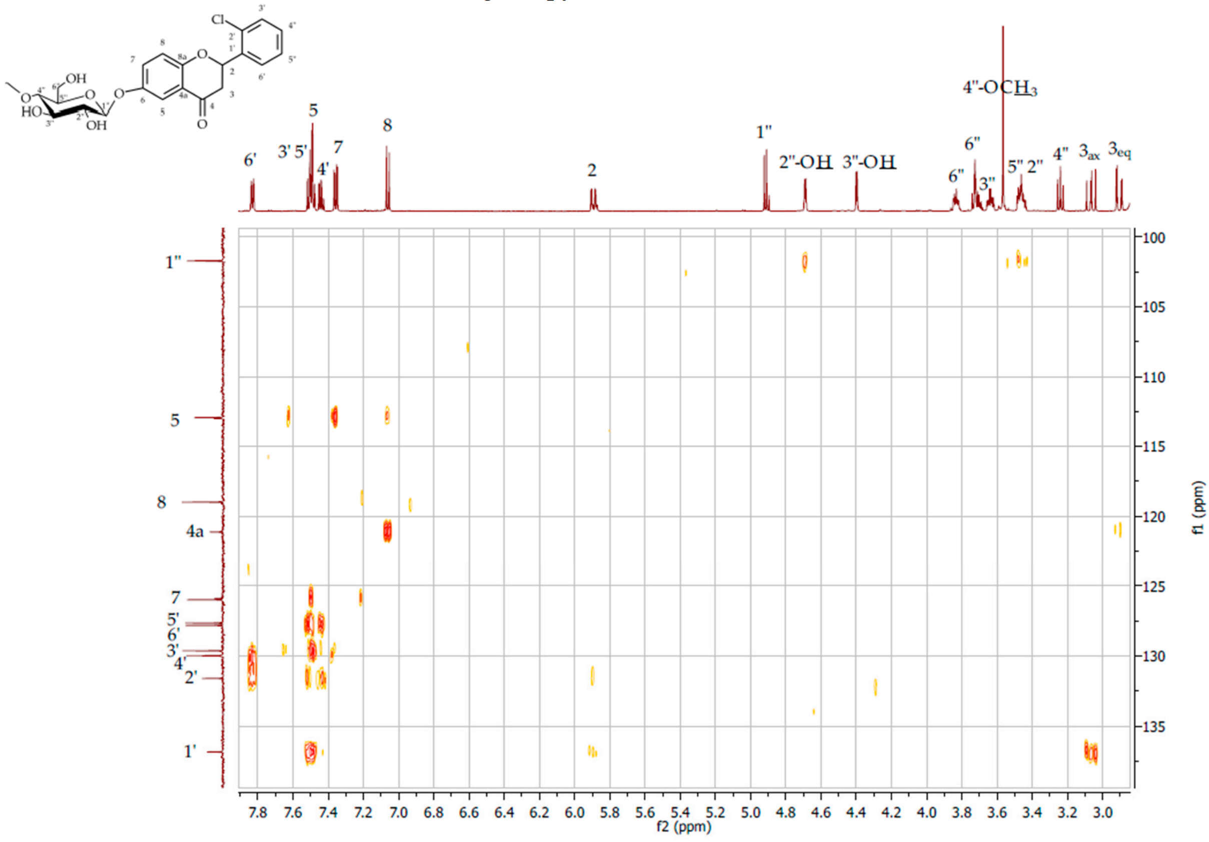

Figure S33. HMBC contour map – ^{1}H x ^{13}C expansion of 2′-chloroflavanone 6-*O*-*β*-D-(4′′-*O*-methyl)-glucopyranoside (**1a**).

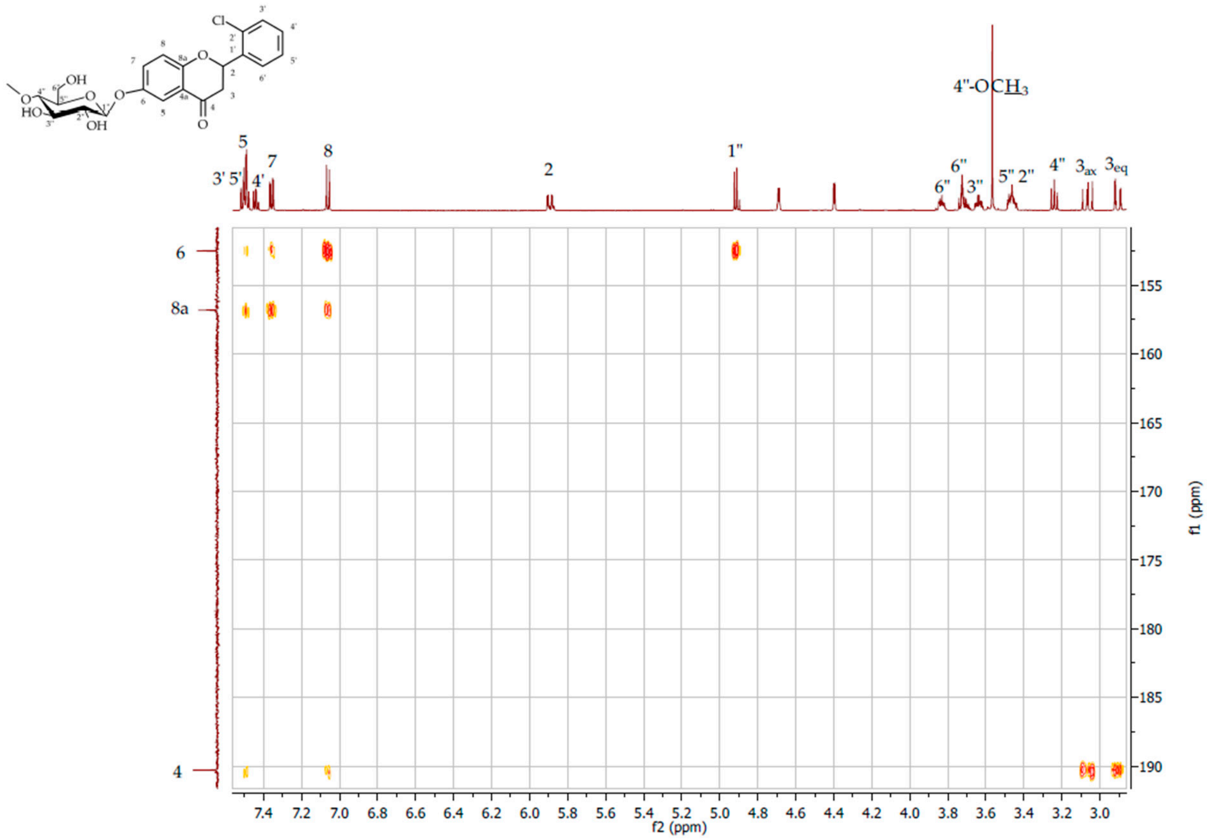

Figure S34. HMBC contour map – ^{1}H x ^{13}C expansion of 2′-chloroflavanone 6-*O*-*β*-D-(4′′-*O*-methyl)-glucopyranoside (**1a**).

Molecule 1

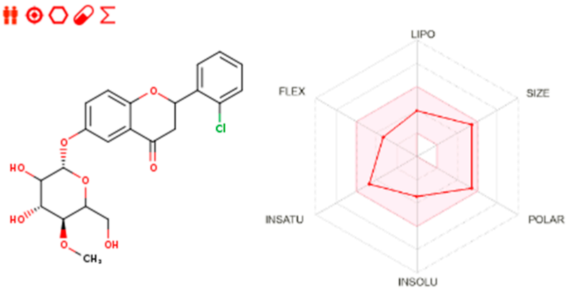

SMILES OCC1O[C@@H](Oc2ccc3c(c2)C(=O)CC(O3)c2ccccc2Cl)C([C@H]([C@@H]1OC)O)O

Physicochemical Properties	
Formula	C22H23ClO8
Molecular weight	450.87 g/mol
Num. heavy atoms	31
Num. arom. heavy atoms	12
Fraction Csp3	0.41
Num. rotatable bonds	5
Num. H-bond acceptors	8
Num. H-bond donors	3
Molar Refractivity	109.39
TPSA	114.68 Å²

Lipophilicity	
Log $P_{o/w}$ (iLOGP)	2.96
Log $P_{o/w}$ (XLOGP3)	1.36
Log $P_{o/w}$ (WLOGP)	1.55
Log $P_{o/w}$ (MLOGP)	0.29
Log $P_{o/w}$ (SILICOS-IT)	2.02
Consensus Log $P_{o/w}$	1.64

Water Solubility	
Log *S* (ESOL)	-3.45
Solubility	1.60e-01 mg/ml ; 3.56e-04 mol/l
Class	Soluble
Log *S* (Ali)	-3.37
Solubility	1.92e-01 mg/ml ; 4.26e-04 mol/l
Class	Soluble
Log *S* (SILICOS-IT)	-4.16
Solubility	3.13e-02 mg/ml ; 6.95e-05 mol/l
Class	Moderately soluble

Pharmacokinetics	
GI absorption	High
BBB permeant	No
P-gp substrate	Yes
CYP1A2 inhibitor	No
CYP2C19 inhibitor	No
CYP2C9 inhibitor	No
CYP2D6 inhibitor	No
CYP3A4 inhibitor	Yes
Log K_p (skin permeation)	-8.08 cm/s

Druglikeness	
Lipinski	Yes; 0 violation
Ghose	Yes
Veber	Yes
Egan	Yes
Muegge	Yes
Bioavailability Score	0.55

Medicinal Chemistry	
PAINS	0 alert
Brenk	0 alert
Leadlikeness	No; 1 violation: MW>350
Synthetic accessibility	5.05

Figure S35. 2′-Chloroflavanone 6-*O*-*β*-D-(4′′-*O*-methyl)-glucopyranoside (**1a**) physicochemical and ADME parameters prediction using the SwissADME modelling.

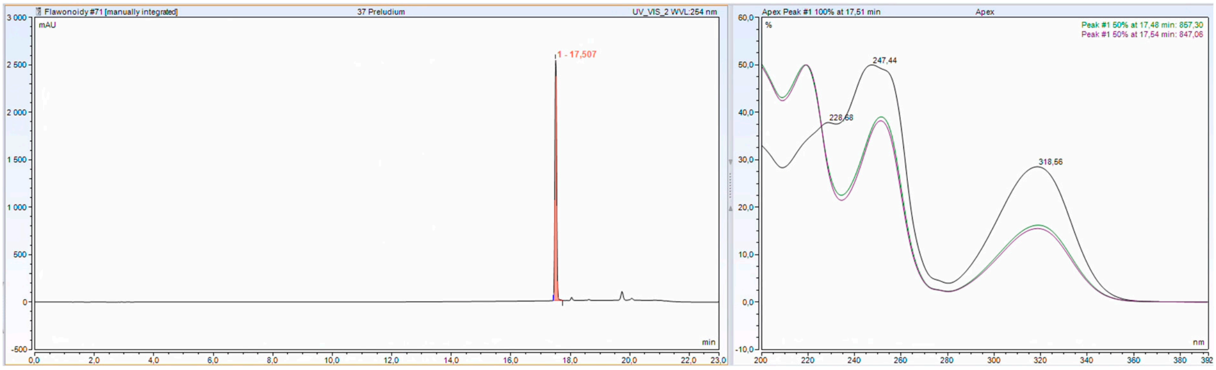

Figure S36. HPLC analysis of 3′-chloroflavanone (**2**).

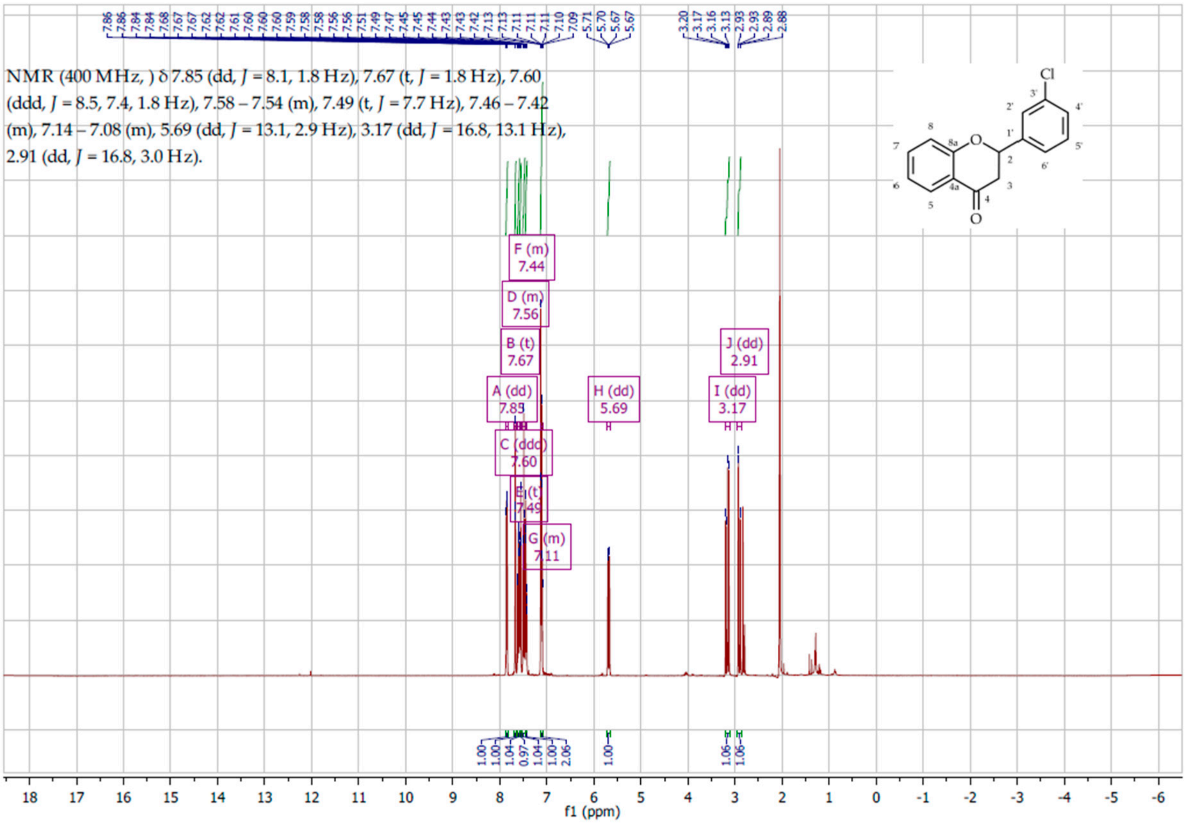

Figure S37. ^{1}H NMR spectrum (*δ*, acetone-d6, 600 MHz) of 3′-chloroflavanone (**2**).

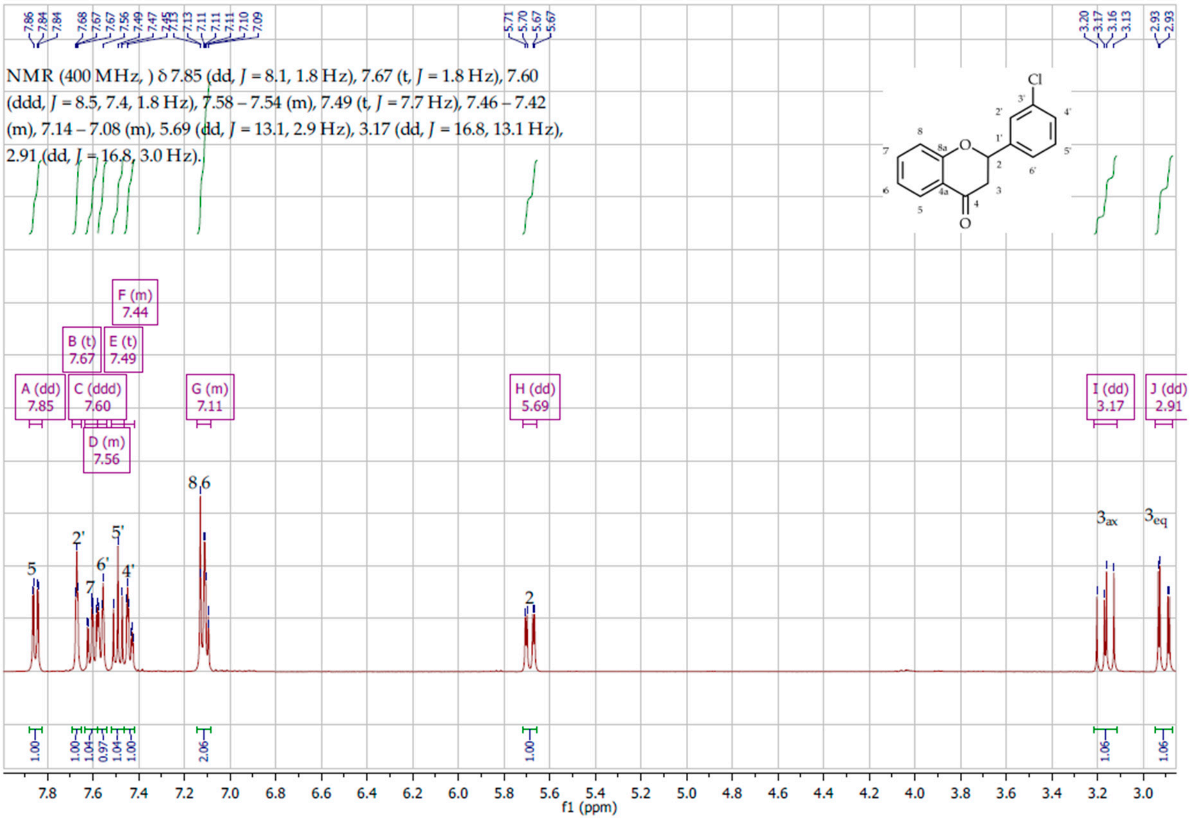

Figure S38. ^{1}H NMR spectrum expansion (*δ*, acetone-d6, 600 MHz) of 3′-chloroflavanone (**2**).

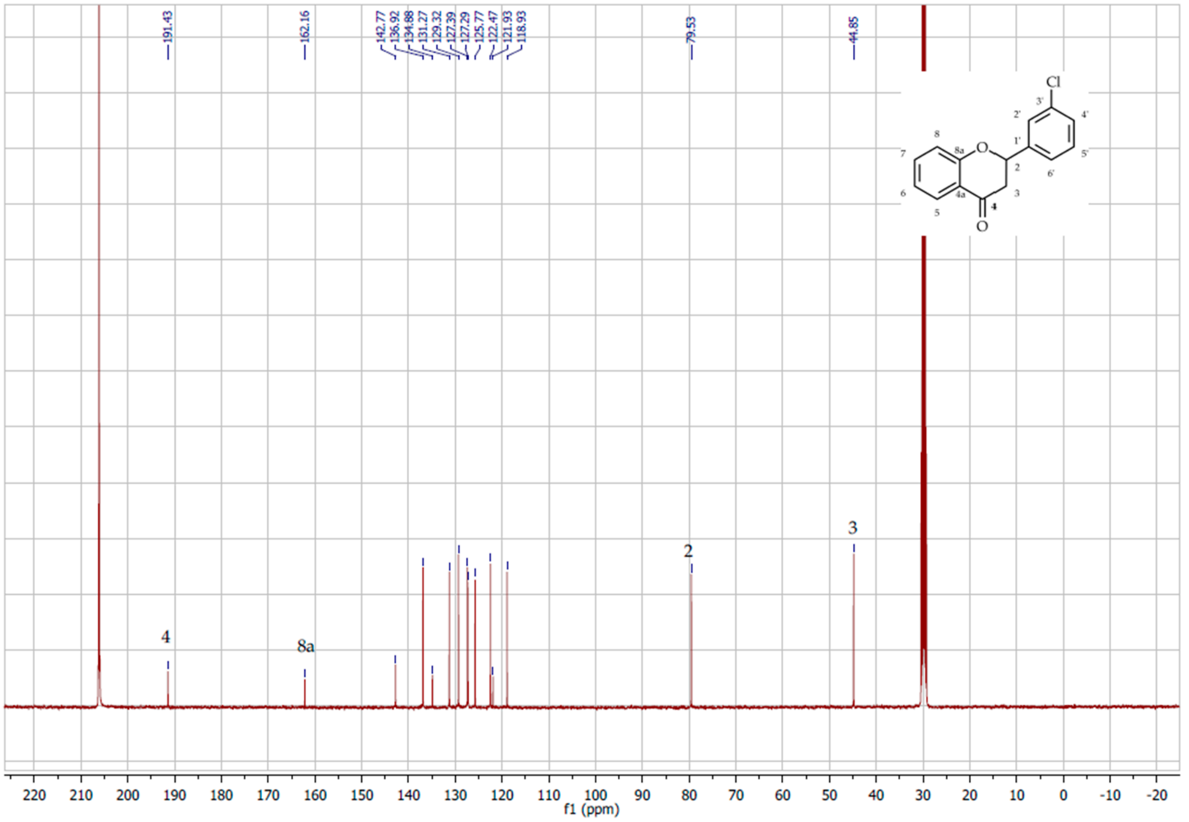

Figure S39. ^{13}C NMR spectrum (*δ*, acetone-d6, 151 MHz) of 3′-chloroflavanone (**2**).

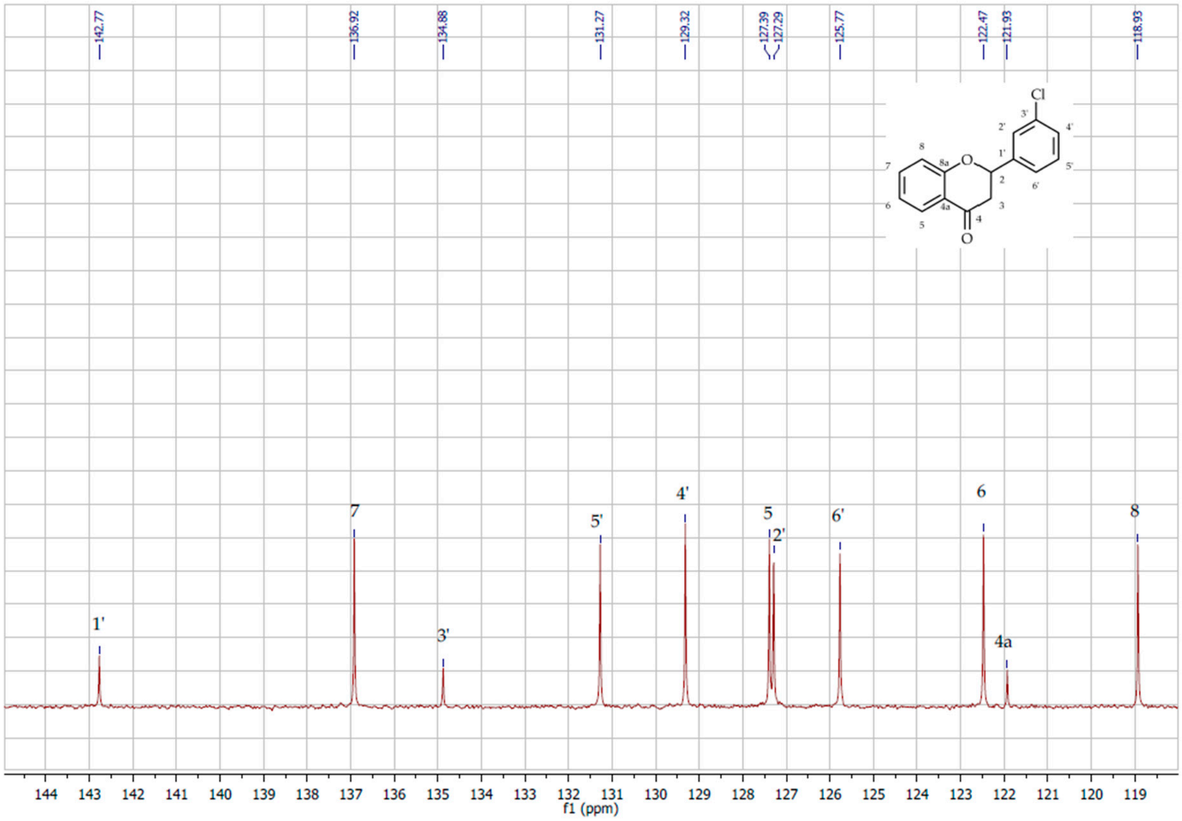

Figure S40. ^{13}C NMR spectrum expansion (*δ*, acetone-d6, 151 MHz) of 3′-chloroflavanone (**2**).

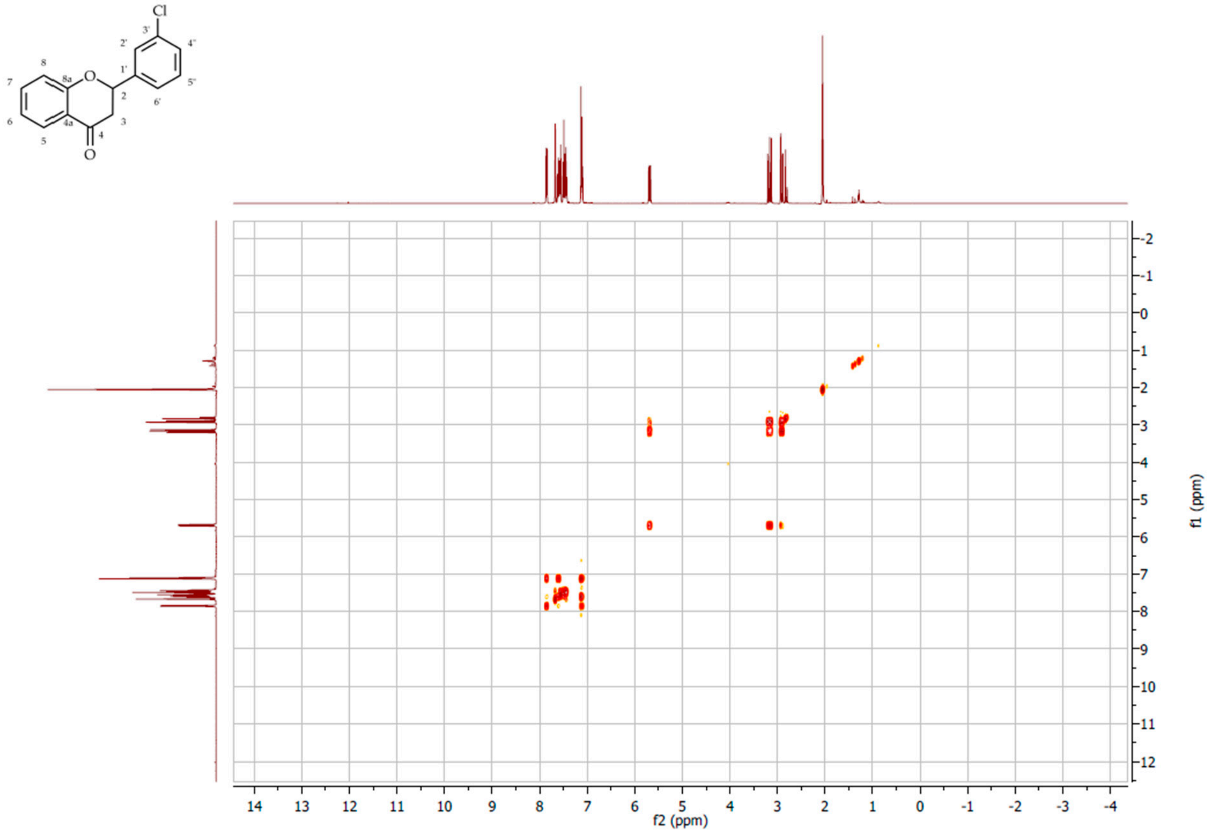

Figure S41. COSY contour map – ^1H x ^1H of 3′-chloroflavanone (**2**).

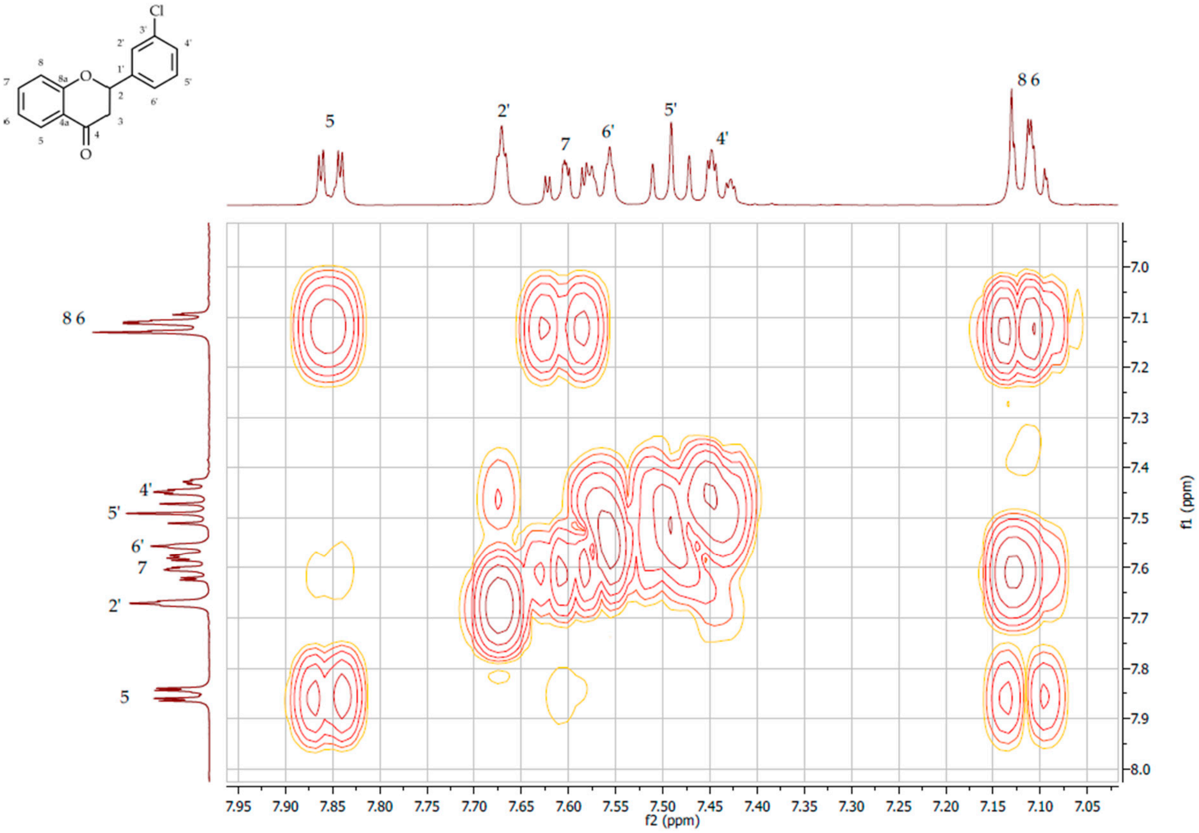

Figure S42. COSY contour map – ^1H x ^1H expansion of 3′-chloroflavanone (**2**).

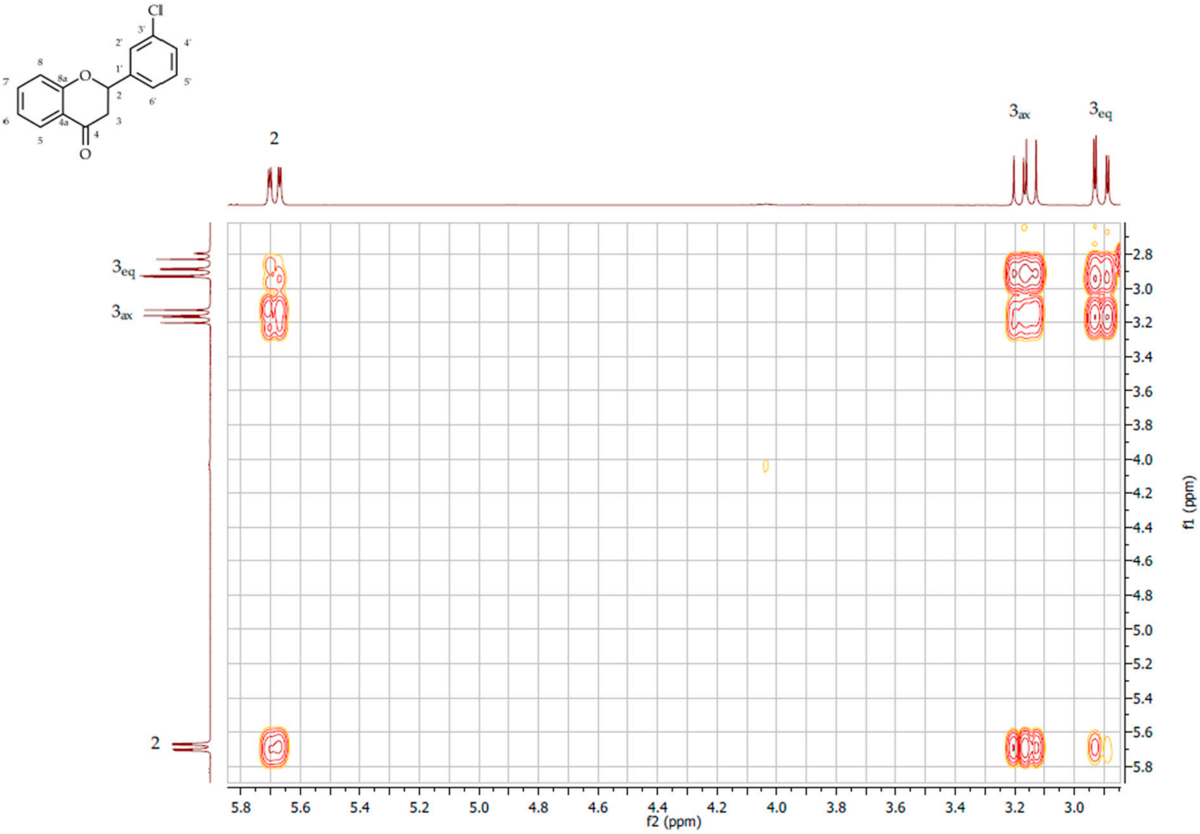

Figure S43. COSY contour map – ^1H x ^1H expansion of 3′-chloroflavanone (**2**).

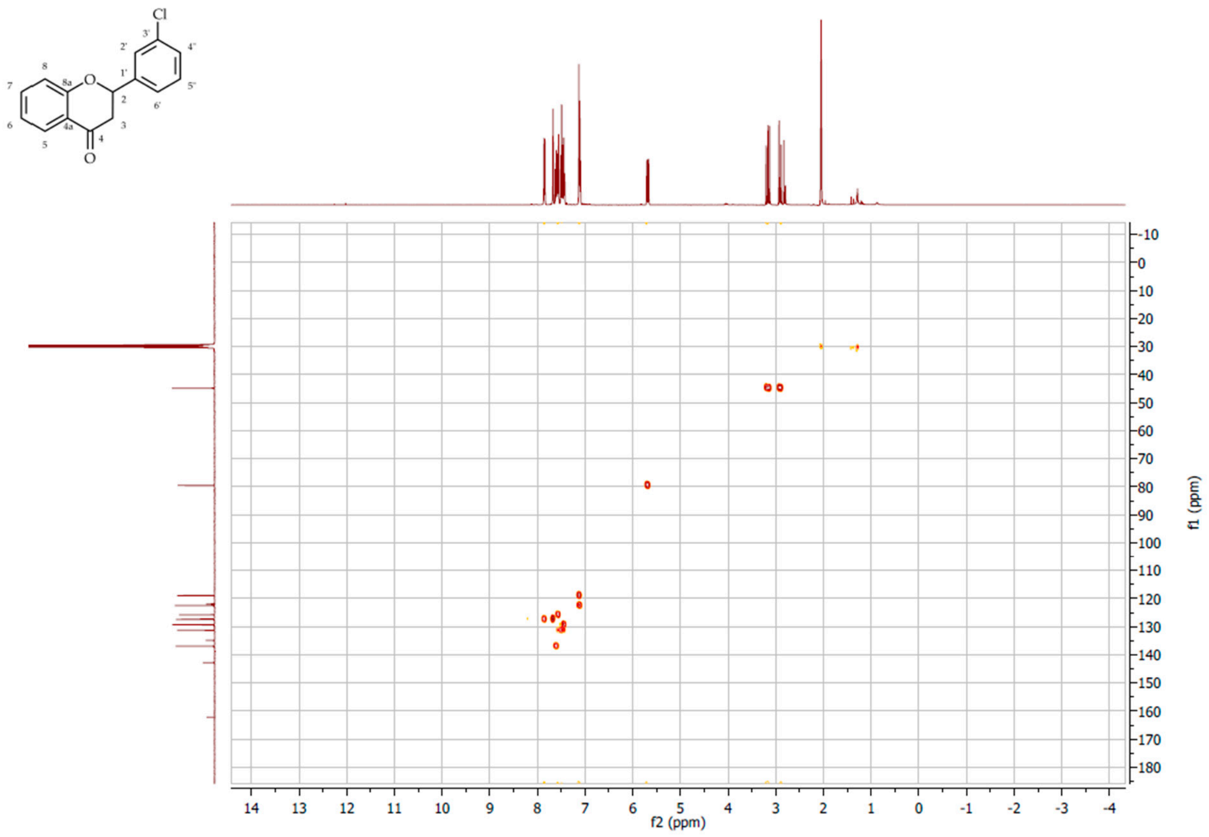

Figure S44. HMQC contour map – ^{1}H x ^{13}C of 3′-chloroflavanone (**2**).

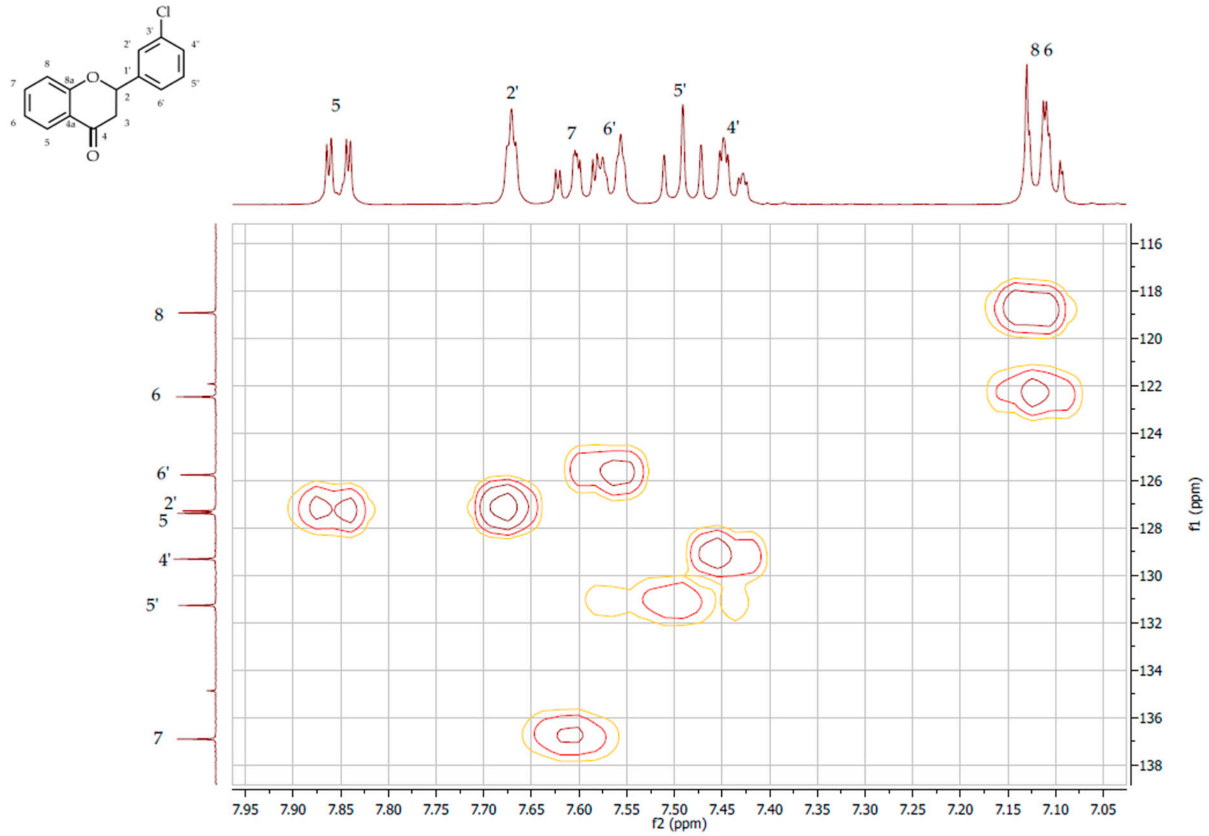

Figure S45. HMQC contour map – ^{1}H x ^{13}C expansion of 3′-chloroflavanone (**2**).

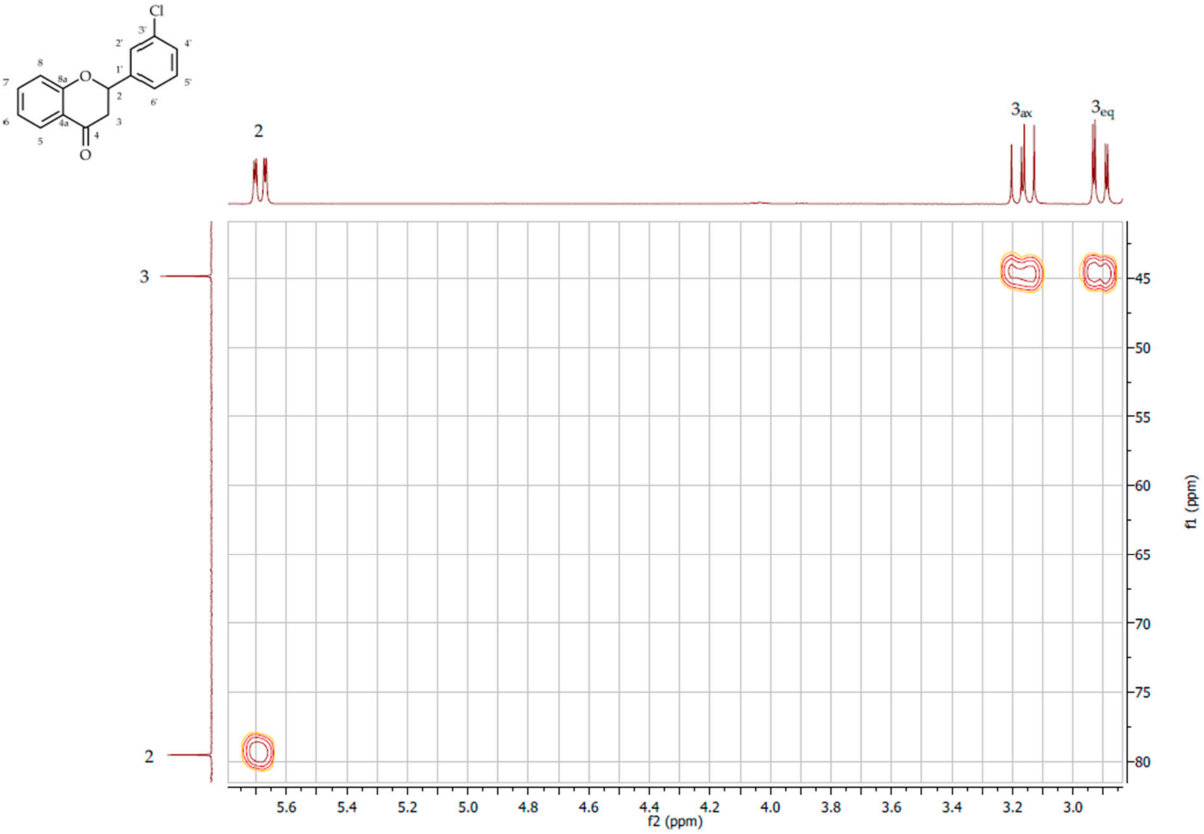

Figure S46. HMQC contour map – ^{1}H x ^{13}C expansion of 3′-chloroflavanone (**2**).

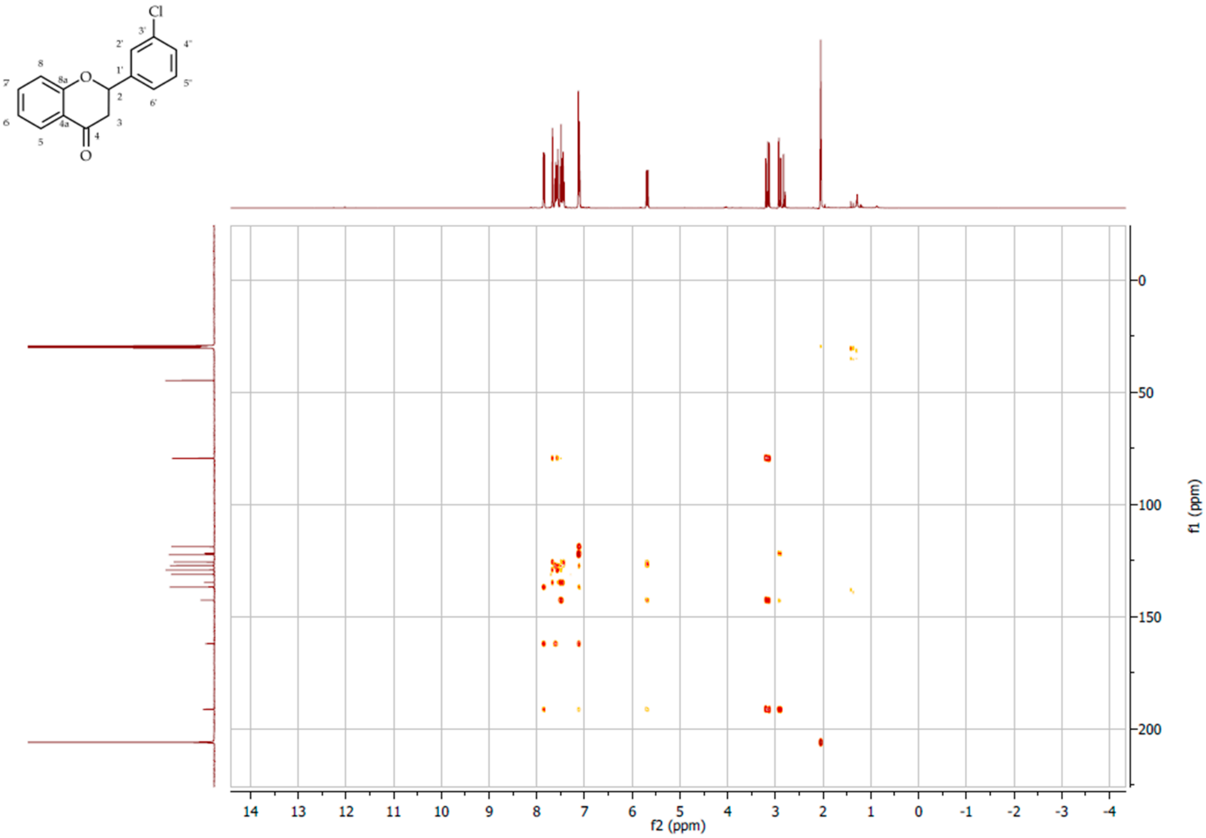

Figure S47. HMBC contour map – ^{1}H x ^{13}C of 3′-chloroflavanone (**2**).

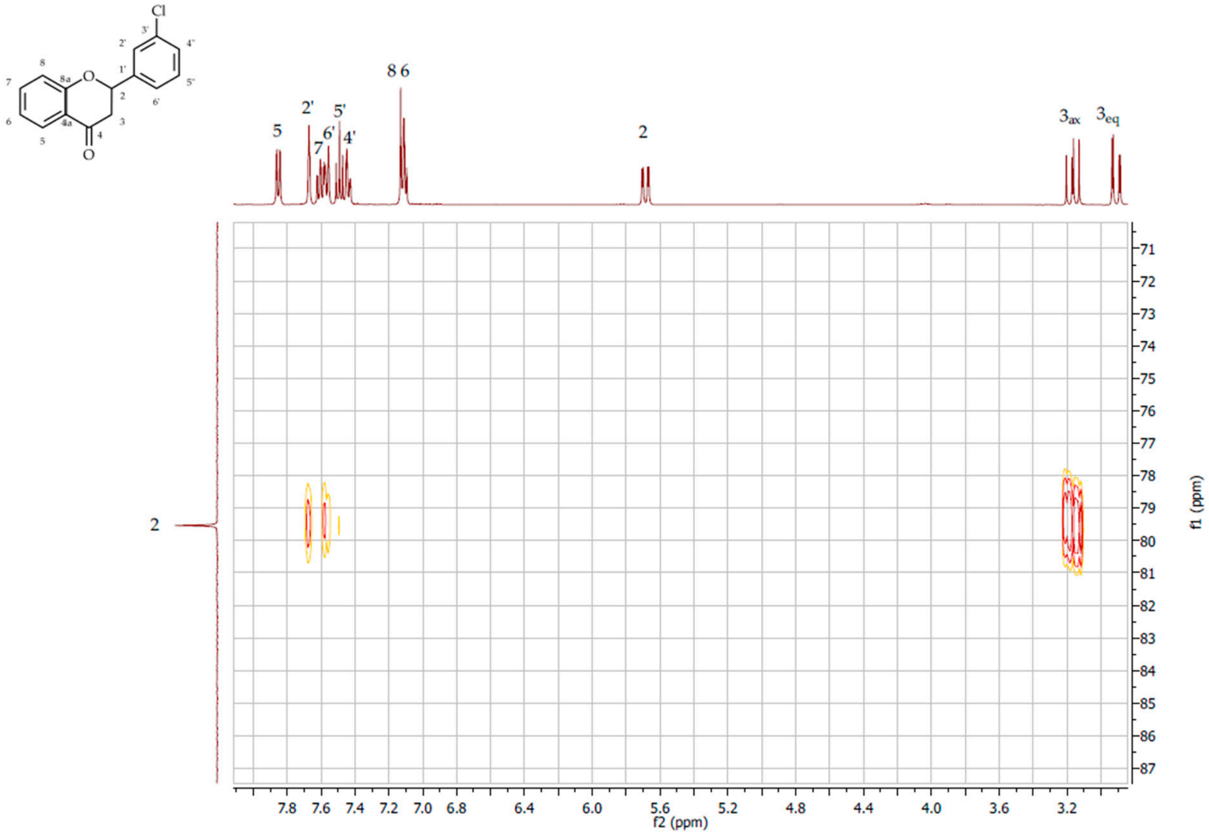

Figure S48. HMBC contour map – ^{1}H x ^{13}C expansion of 3′-chloroflavanone (**2**).

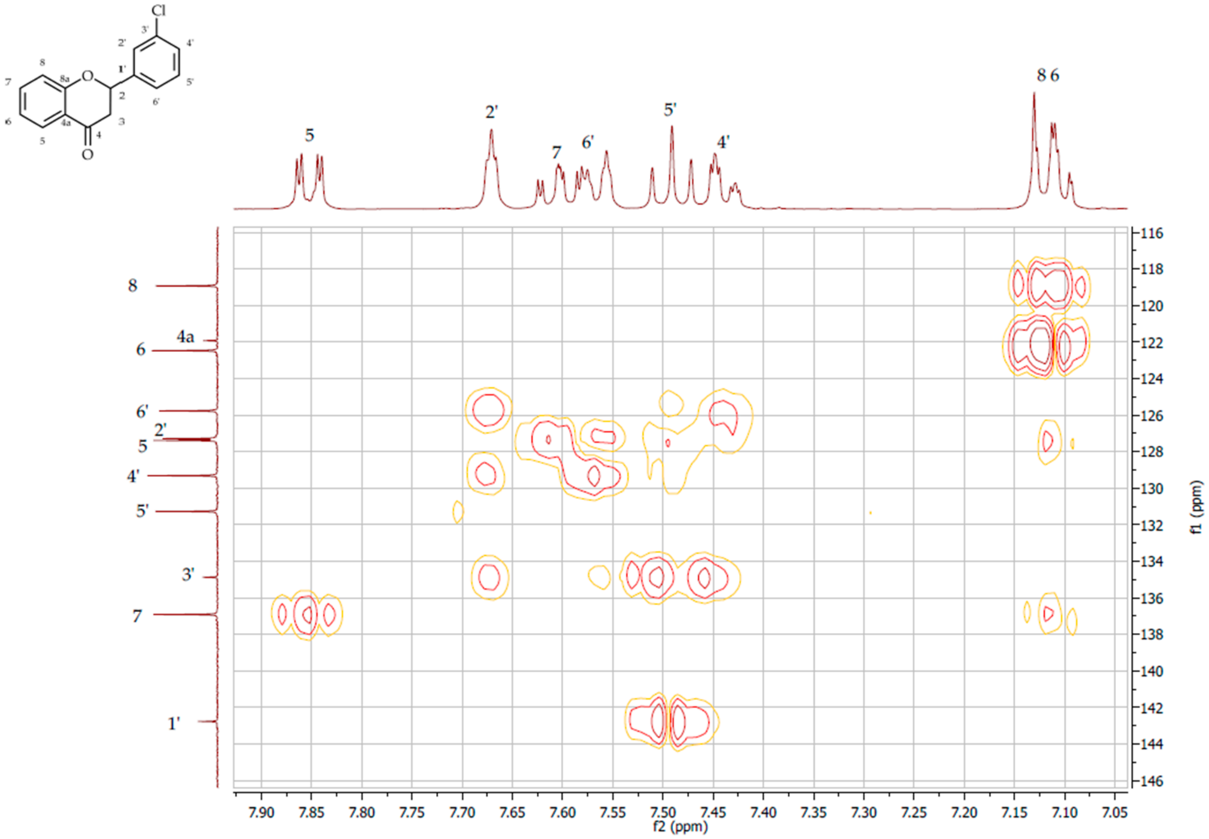

Figure S49. HMBC contour map – ^{1}H x ^{13}C expansion of 3′-chloroflavanone (**2**).

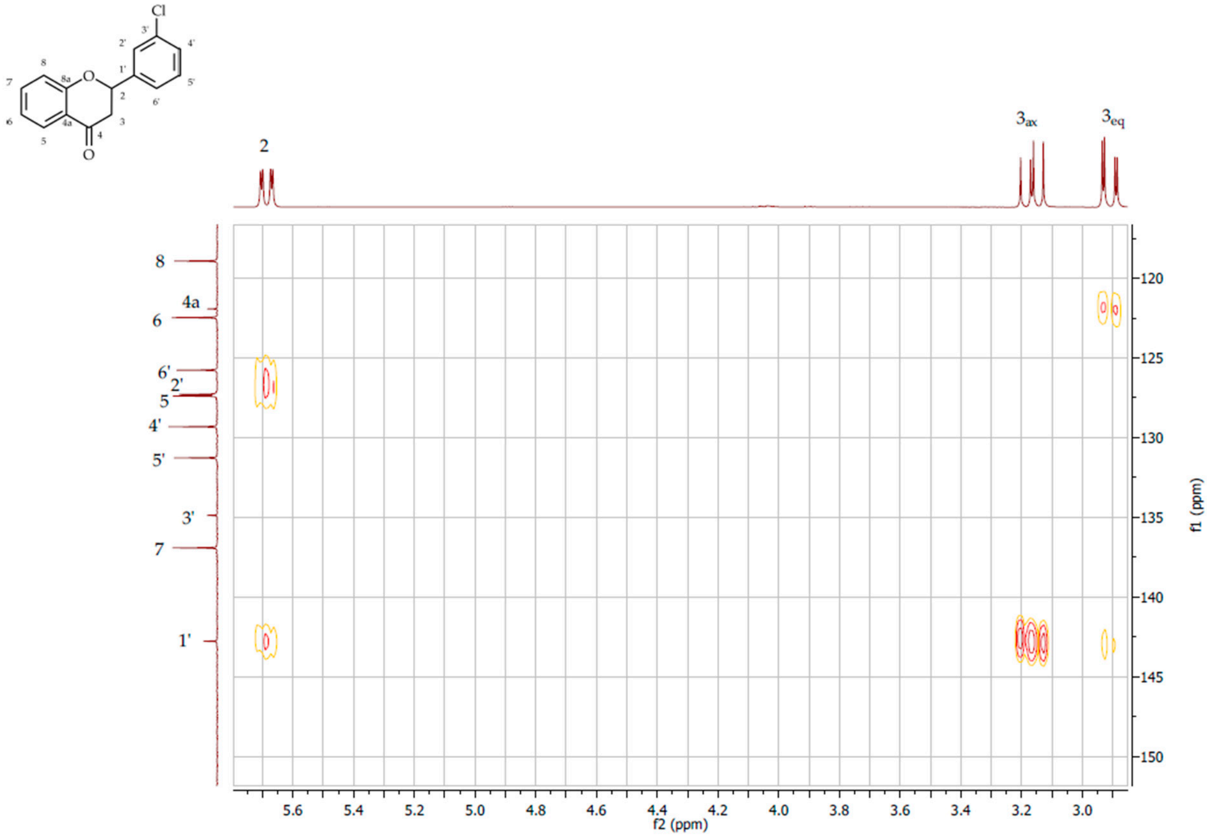

Figure S50. HMBC contour map – ^{1}H x ^{13}C expansion of 3′-chloroflavanone (**2**).

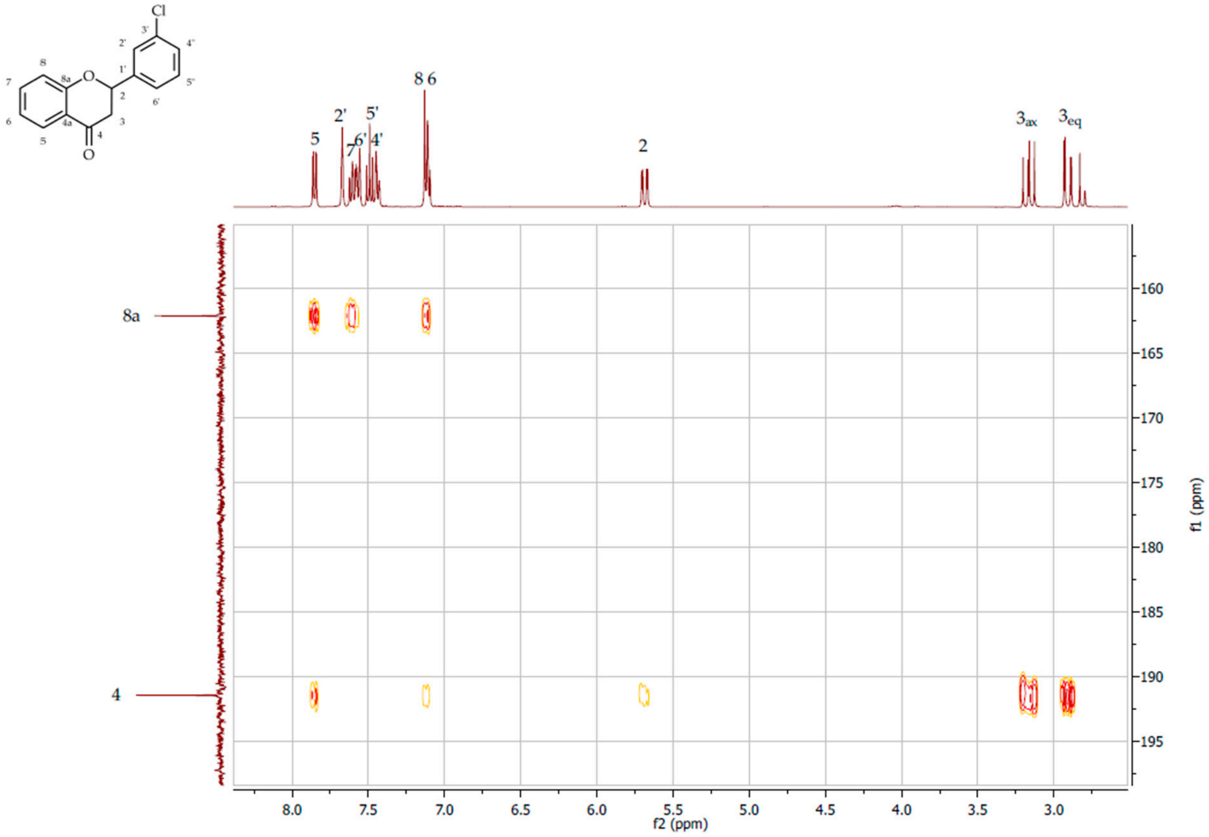

Figure S51. HMBC contour map – ^{1}H x ^{13}C expansion of 3′-chloroflavanone (**2**).

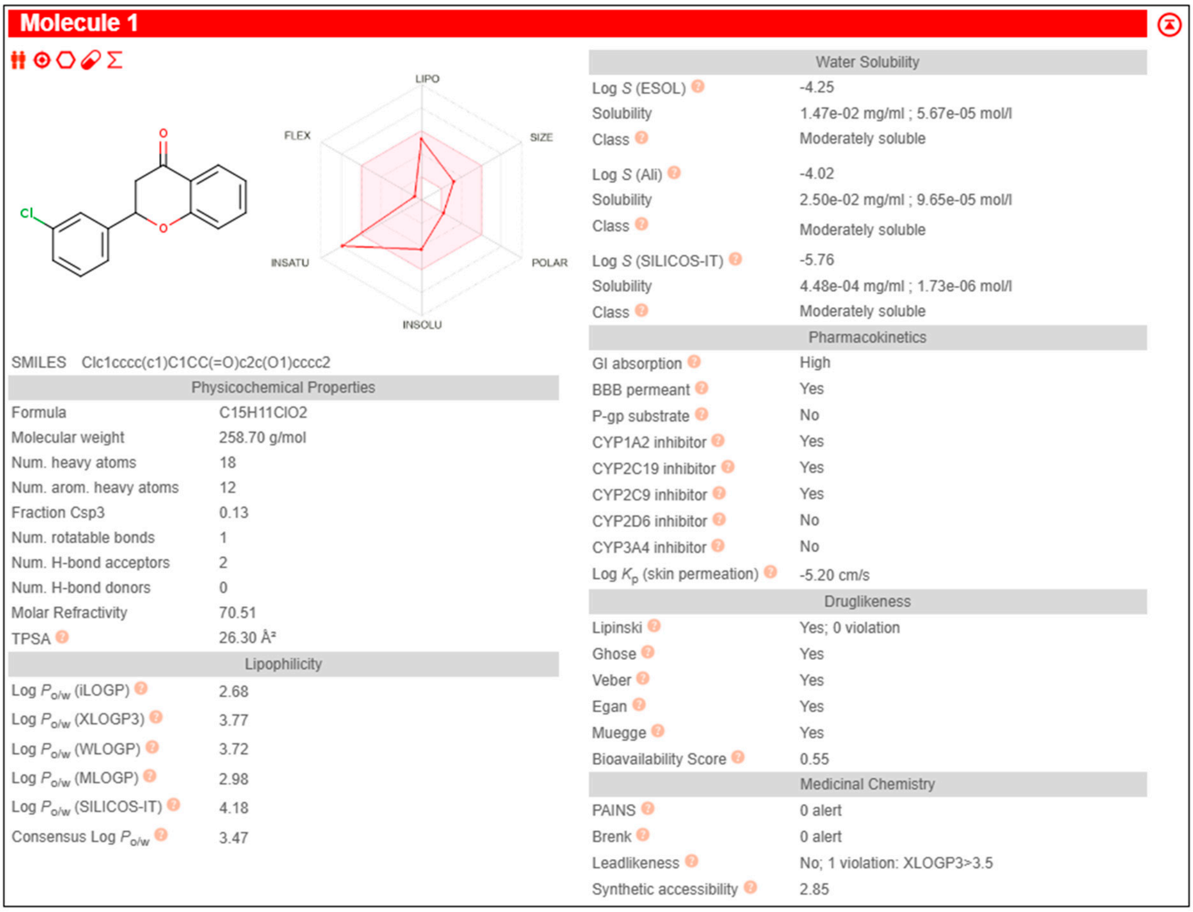

Figure S52. 3′-Chloroflavanone (**2**) physicochemical and ADME parameters prediction using the SwissADME modelling.

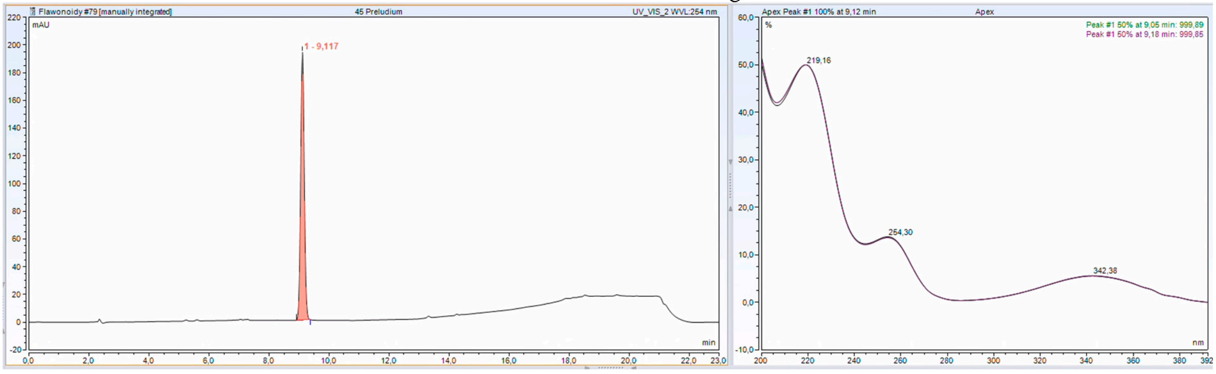

Figure S53. HPLC analysis of 3′-chloroflavanone 6-*O*-*β*-D-(4′′-*O*-methyl)-glucopyranoside (**2a**).

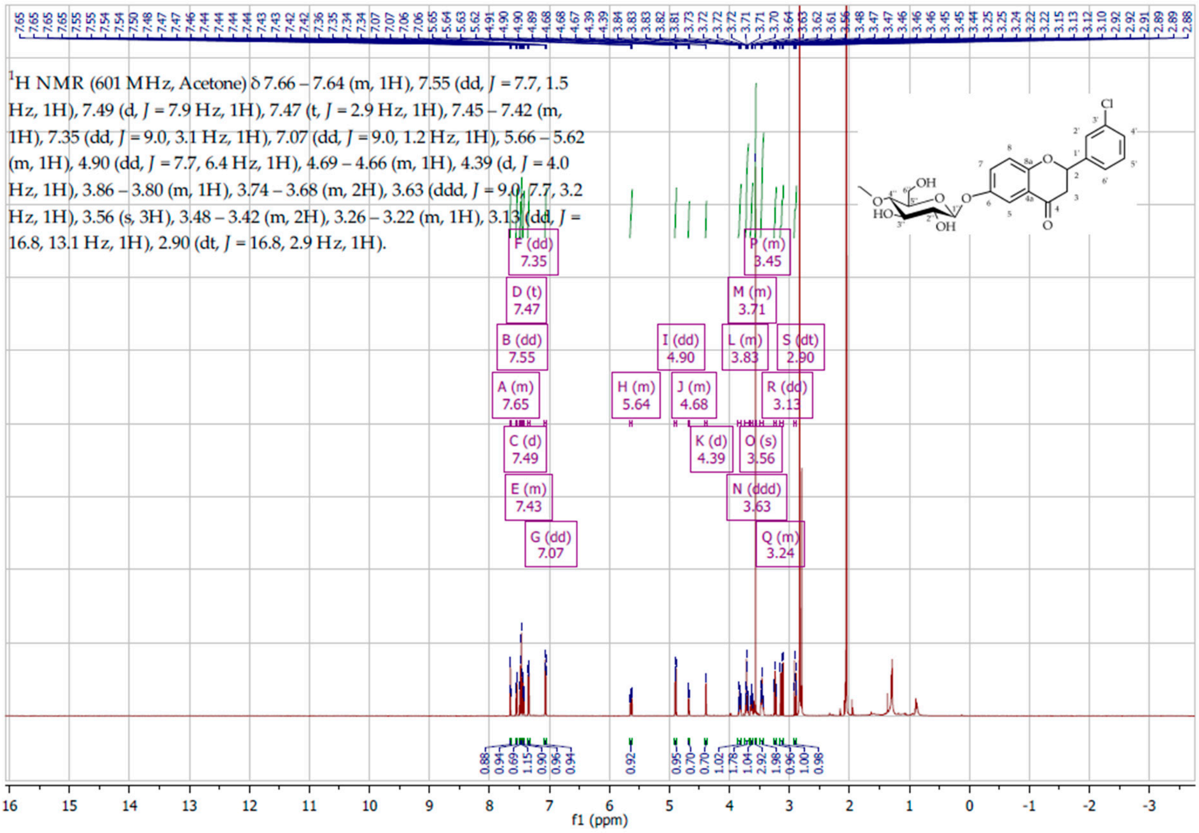

Figure S54. ^{1}H NMR spectrum (*δ*, acetone-d6, 600 MHz) of 3′-chloroflavanone 6-*O*-*β*-D-(4′′-*O*-methyl)-glucopyranoside (**2a**).

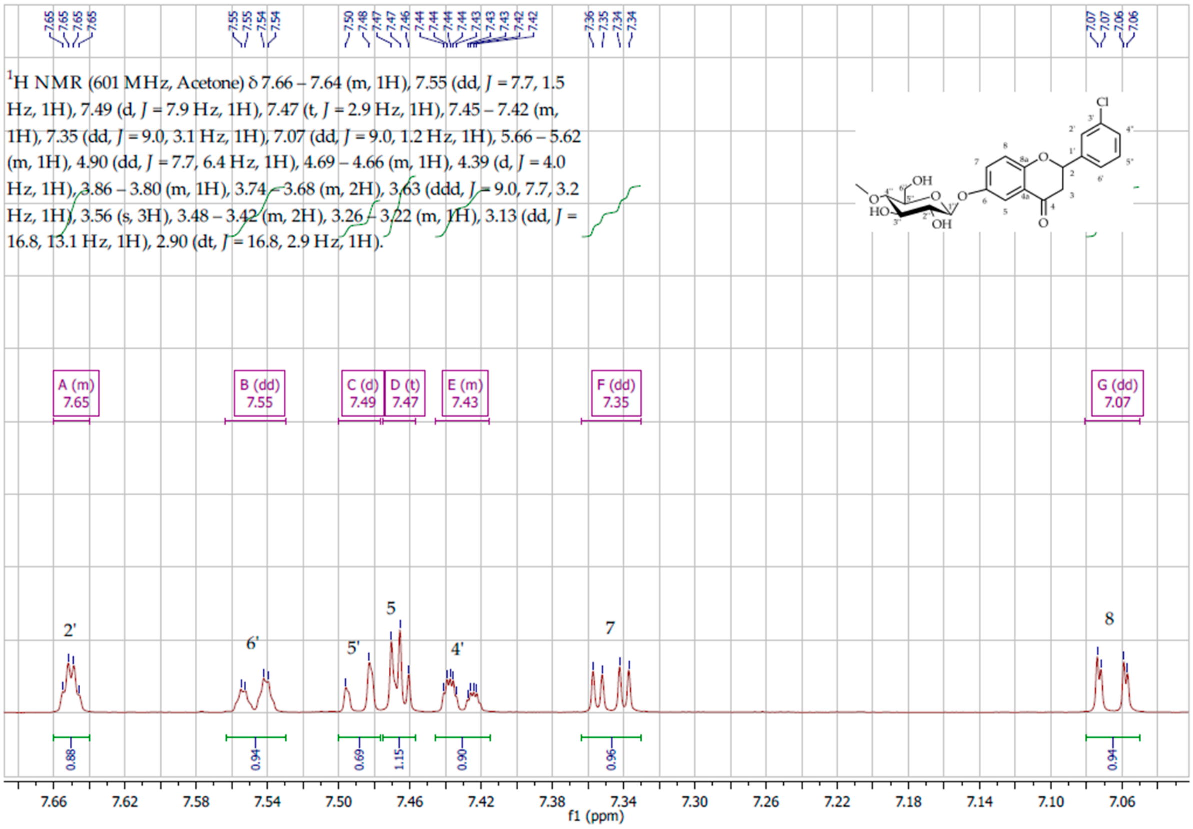

Figure S55. ^{1}H NMR spectrum expansion (*δ*, acetone-d6, 600 MHz) of 3′-chloroflavanone 6-*O*-*β*-D-(4′′-*O*-methyl)-glucopyranoside (**2a**).

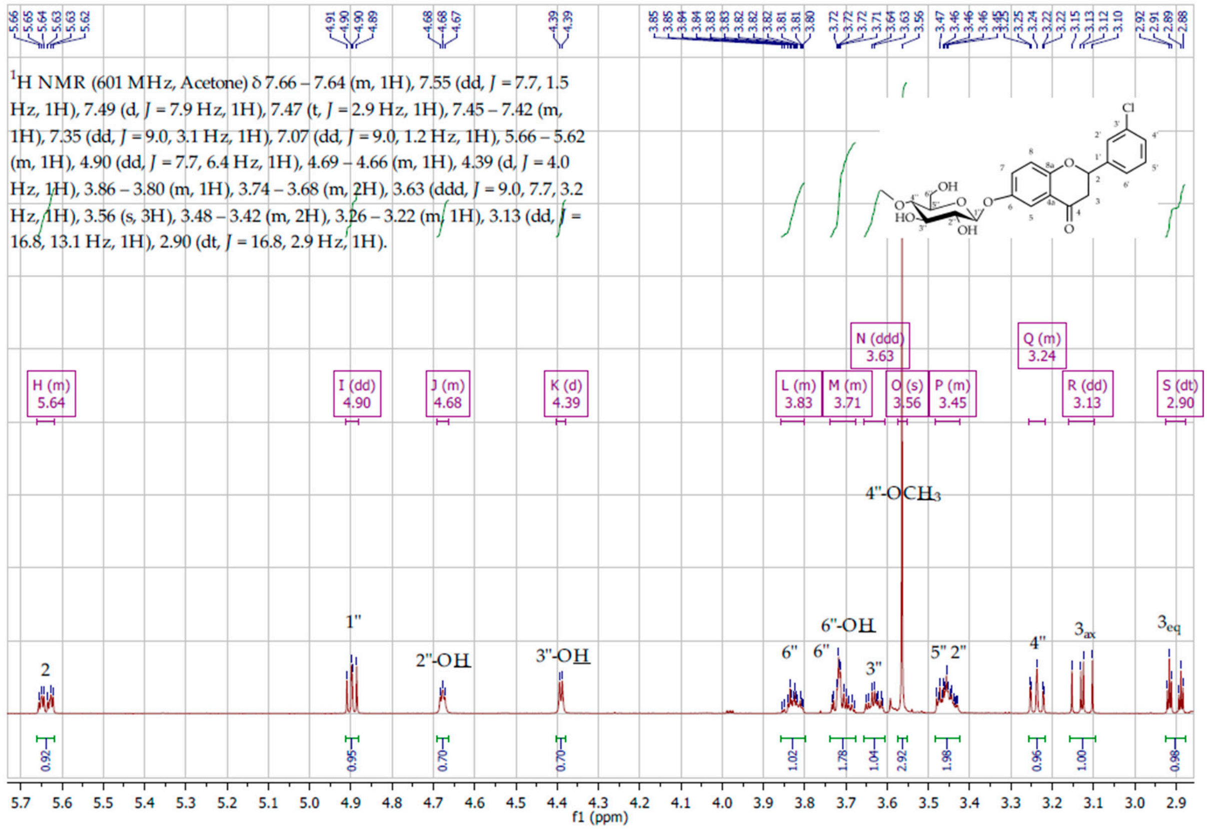

Figure S56. ^{1}H NMR spectrum expansion (*δ*, acetone-d6, 600 MHz) of 3′-chloroflavanone 6-*O*-*β*-D-(4′′-*O*-methyl)-glucopyranoside (**2a**).

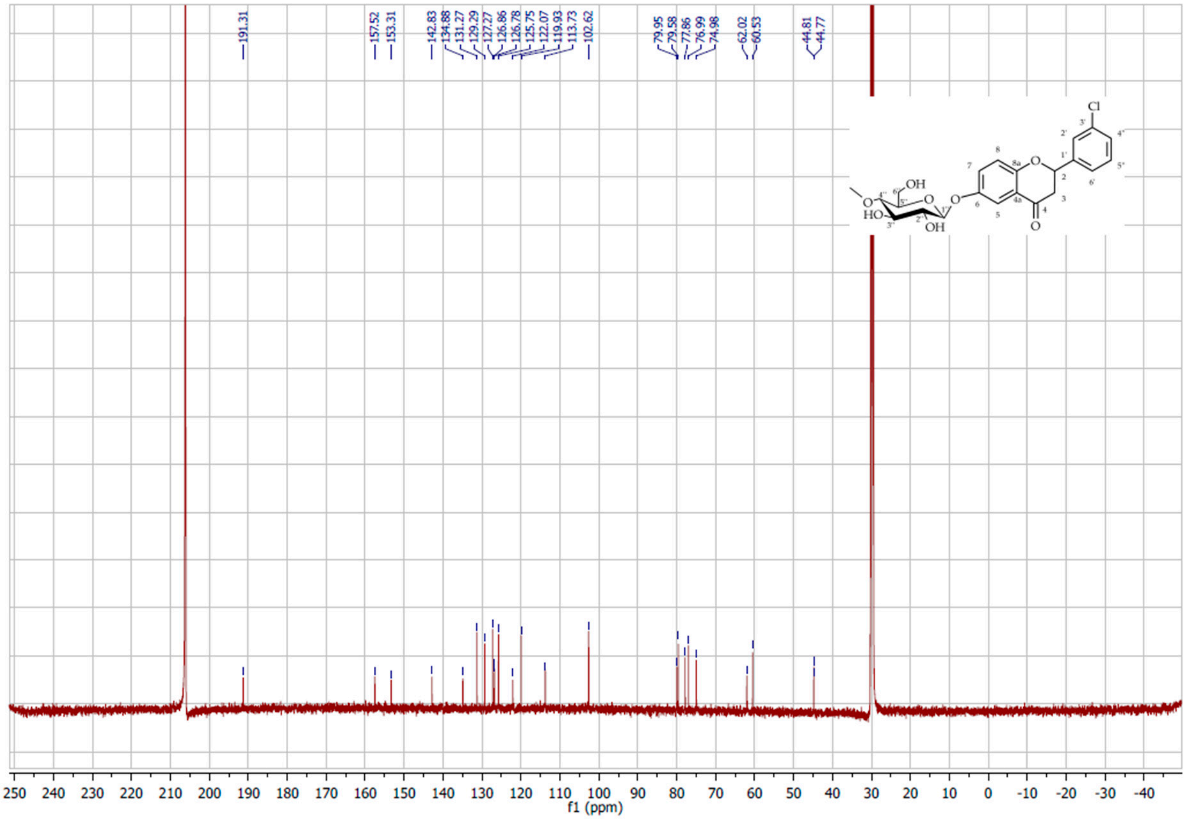

Figure S57. ^{13}C NMR spectrum (*δ*, acetone-d6, 151 MHz) of 3′-chloroflavanone 6-*O*-*β*-D-(4′′-*O*-methyl)-glucopyranoside (**2a**).

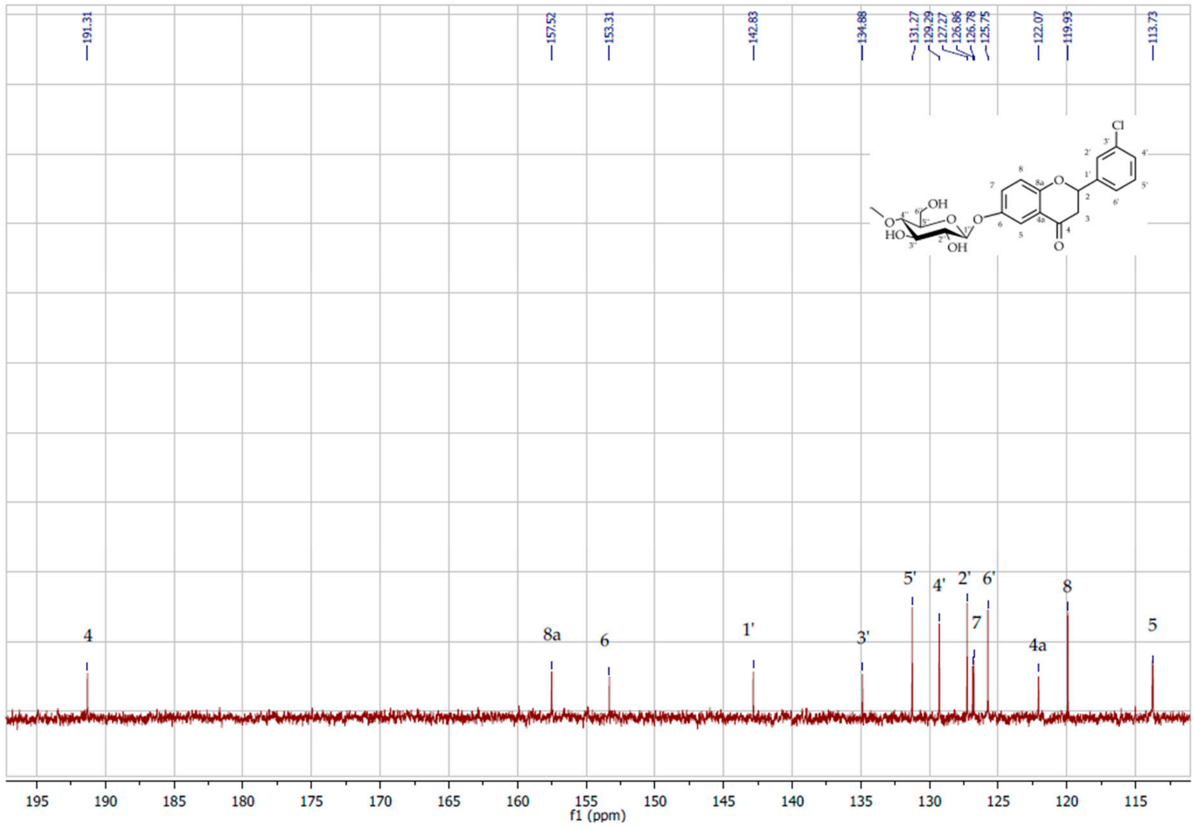

Figure S58. ^{13}C NMR spectrum expansion (*δ*, acetone-d6, 151 MHz) of 3′-chloroflavanone 6-*O*-*β*-D-(4′′-*O*-methyl)-glucopyranoside (**2a**).

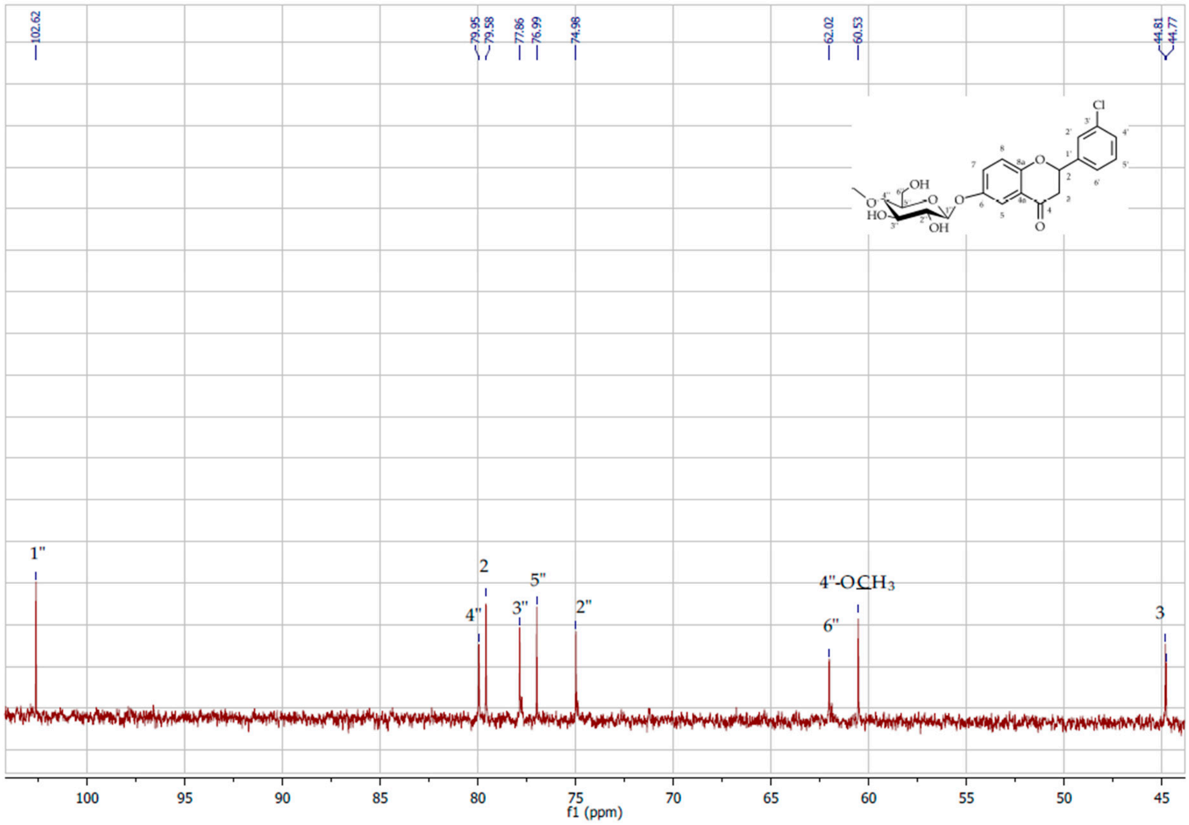

Figure S59. ^{13}C NMR spectrum expansion (*δ*, acetone-d6, 151 MHz) of 3′-chloroflavanone 6-*O*-*β*-D-(4′′-*O*-methyl)-glucopyranoside (**2a**).

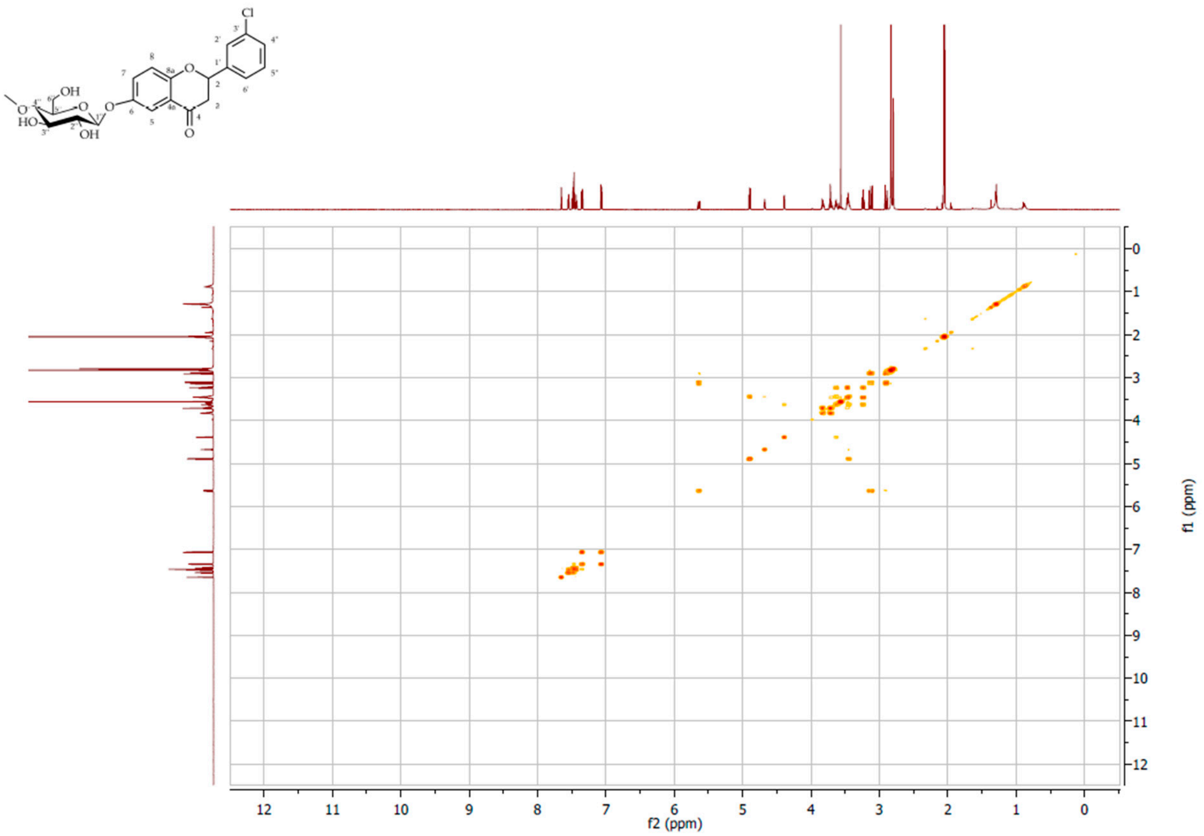

Figure S60. COSY contour map – ^{1}H x ^{1}H of 3′-chloroflavanone 6-*O*-*β*-D-(4′′-*O*-methyl)-glucopyranoside (**2a**).

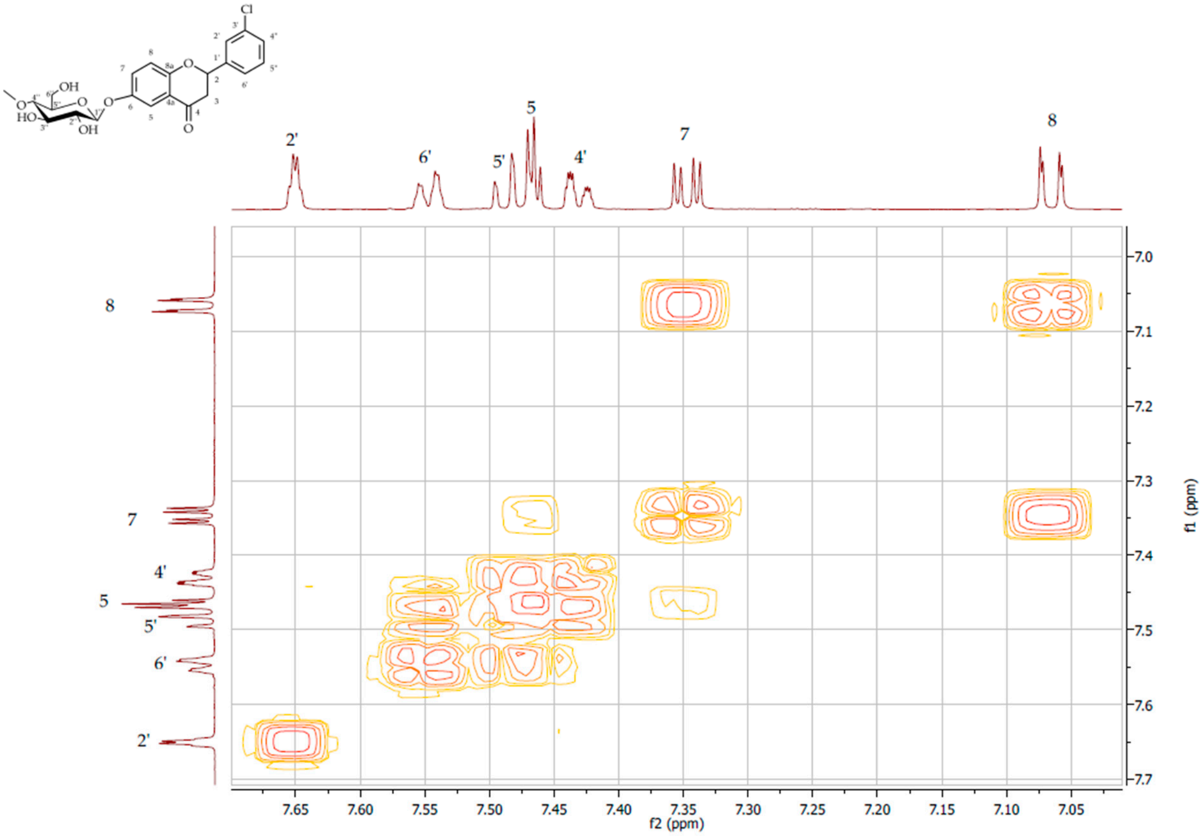

Figure S61. COSY contour map – ^{1}H x ^{1}H expansion of 3′-chloroflavanone 6-*O*-*β*-D-(4′′-*O*-methyl)-glucopyranoside (**2a**).

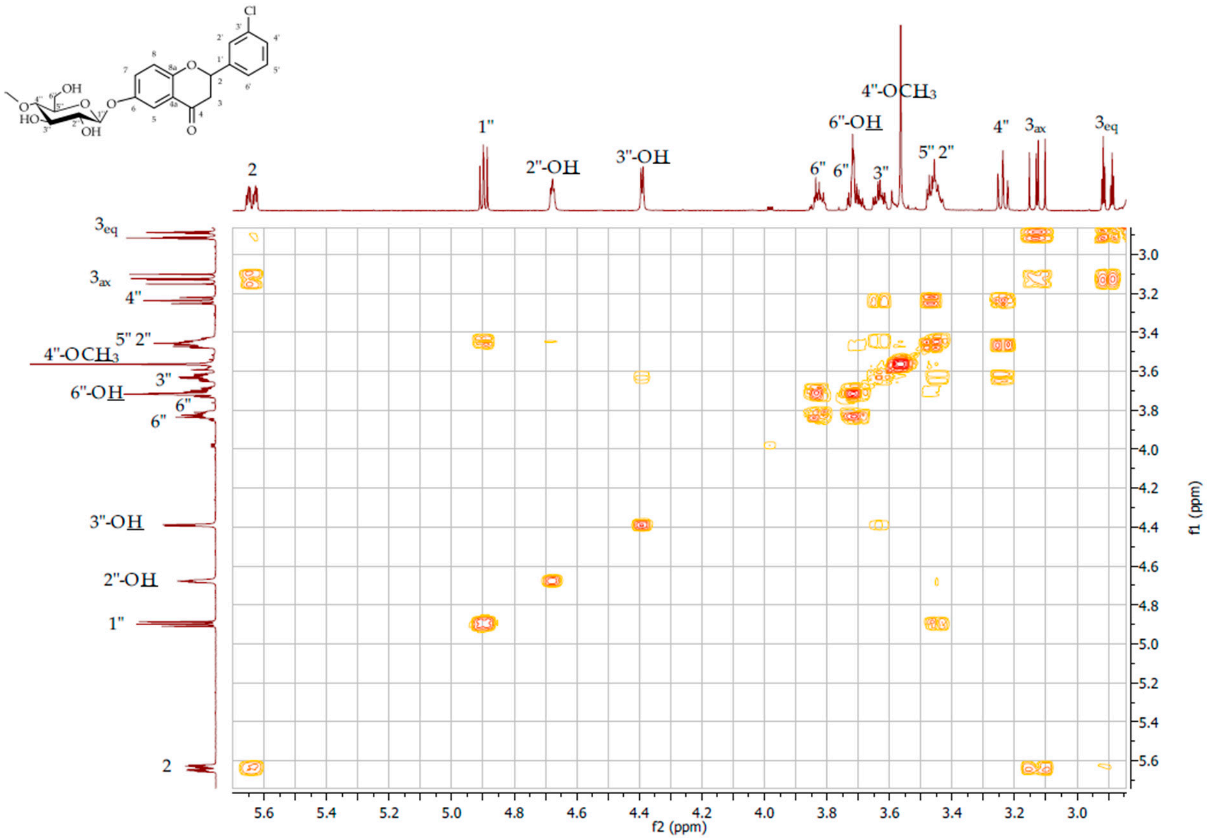

Figure S62. COSY contour map – ^{1}H x ^{1}H expansion of 3′-chloroflavanone 4′-*O*-*β*-D-(4′′-*O*-methyl)-glucopyranoside (**2a**).

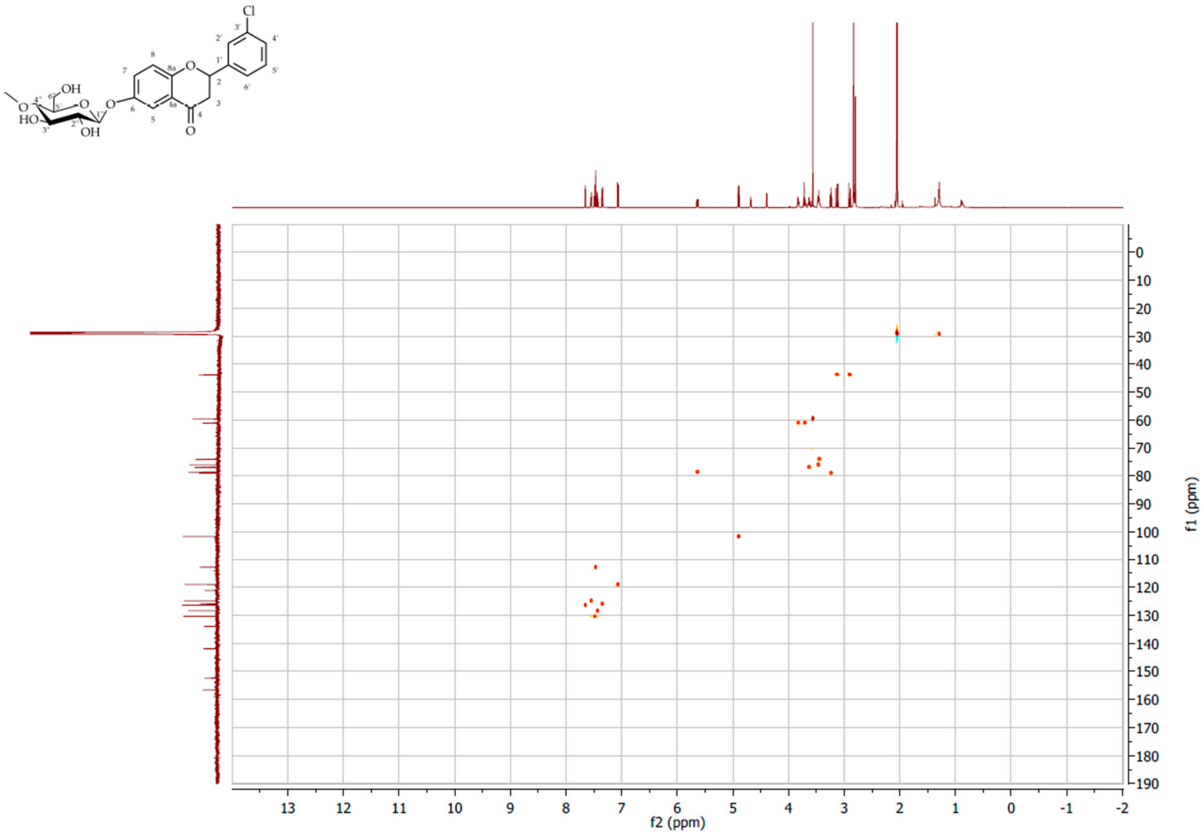

Figure S63. HMQC contour map – ^{1}H x ^{13}C of 3′-chloroflavanone 6-*O*-*β*-D-(4′′-*O*-methyl)-glucopyranoside (**2a**).

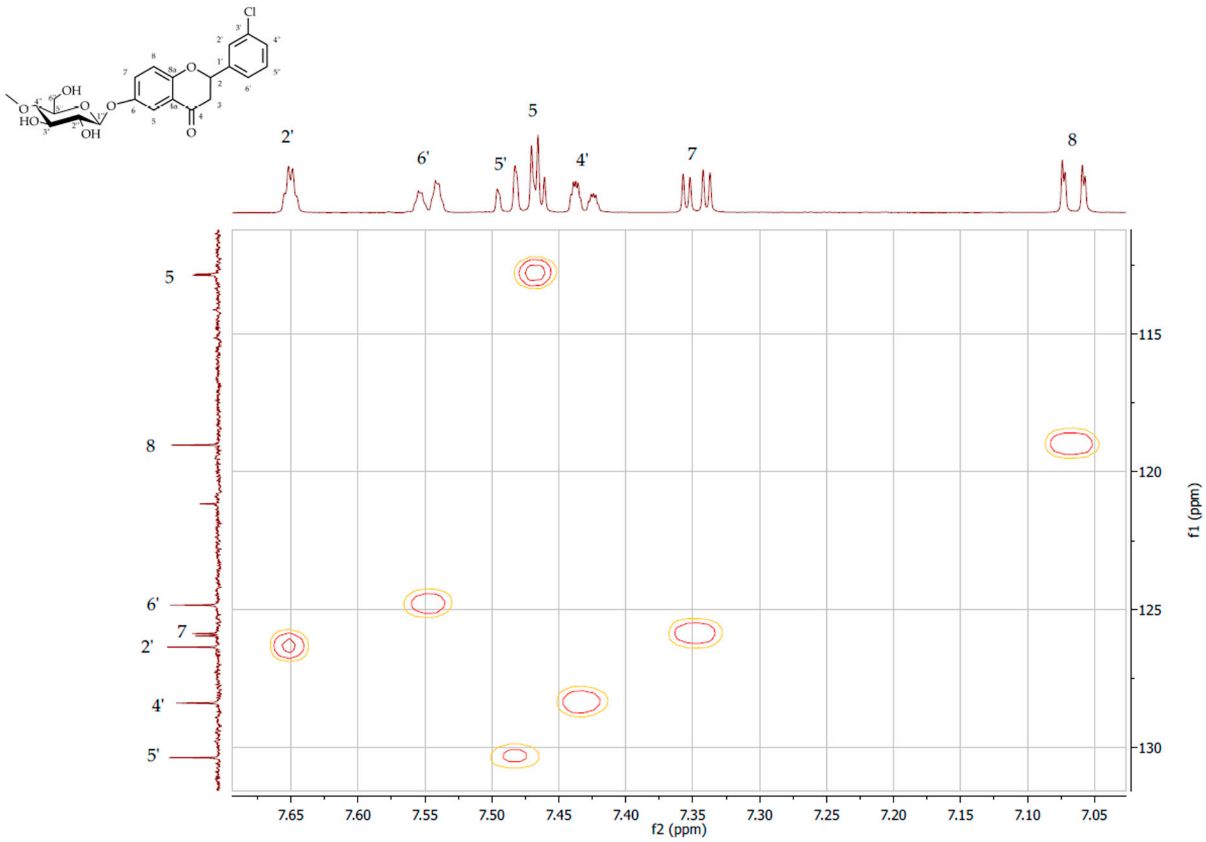

Figure S64. HMQC contour map – ^{1}H x ^{13}C expansion of 3′-chloroflavanone 6-*O*-*β*-D-(4′′-*O*-methyl)-glucopyranoside (**2a**).

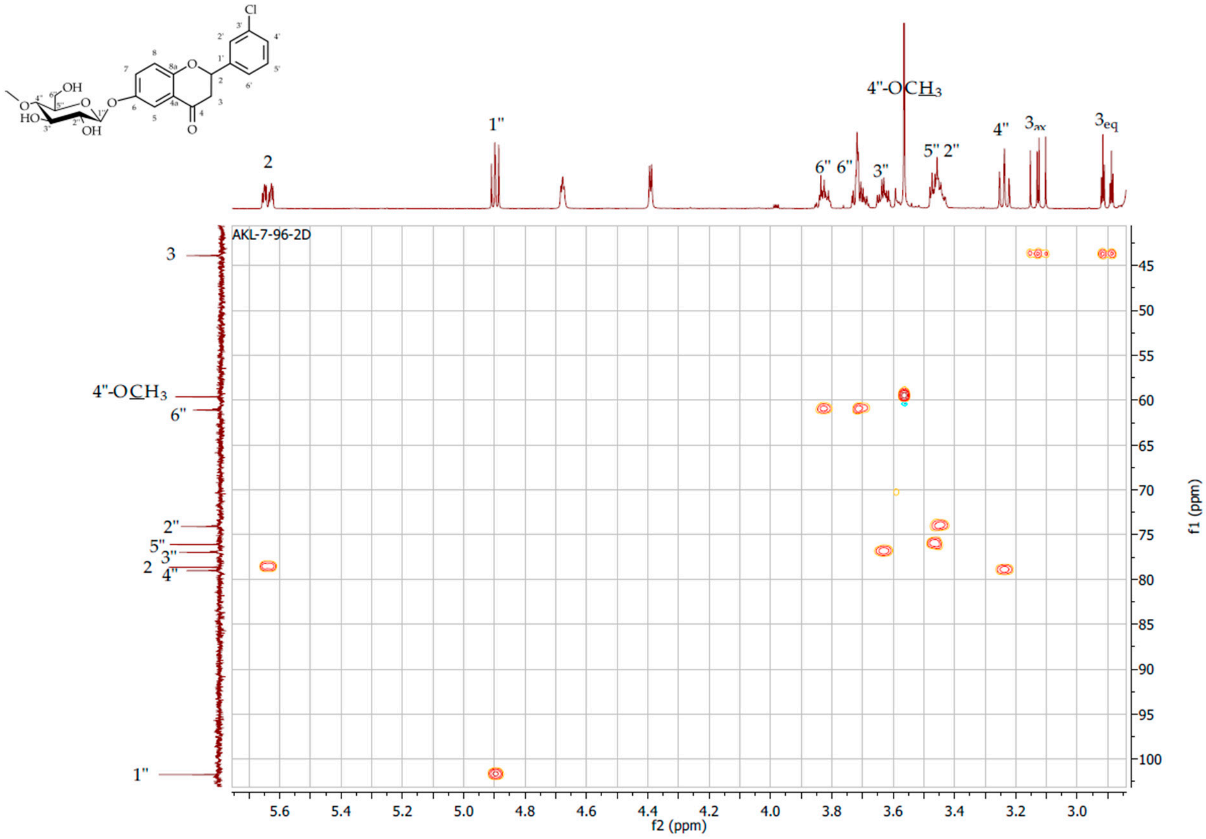

Figure S65. HMQC contour map – ^{1}H x ^{13}C expansion of 3′-chloroflavanone 6-*O*-*β*-D-(4′′-*O*-methyl)-glucopyranoside (**2a**).

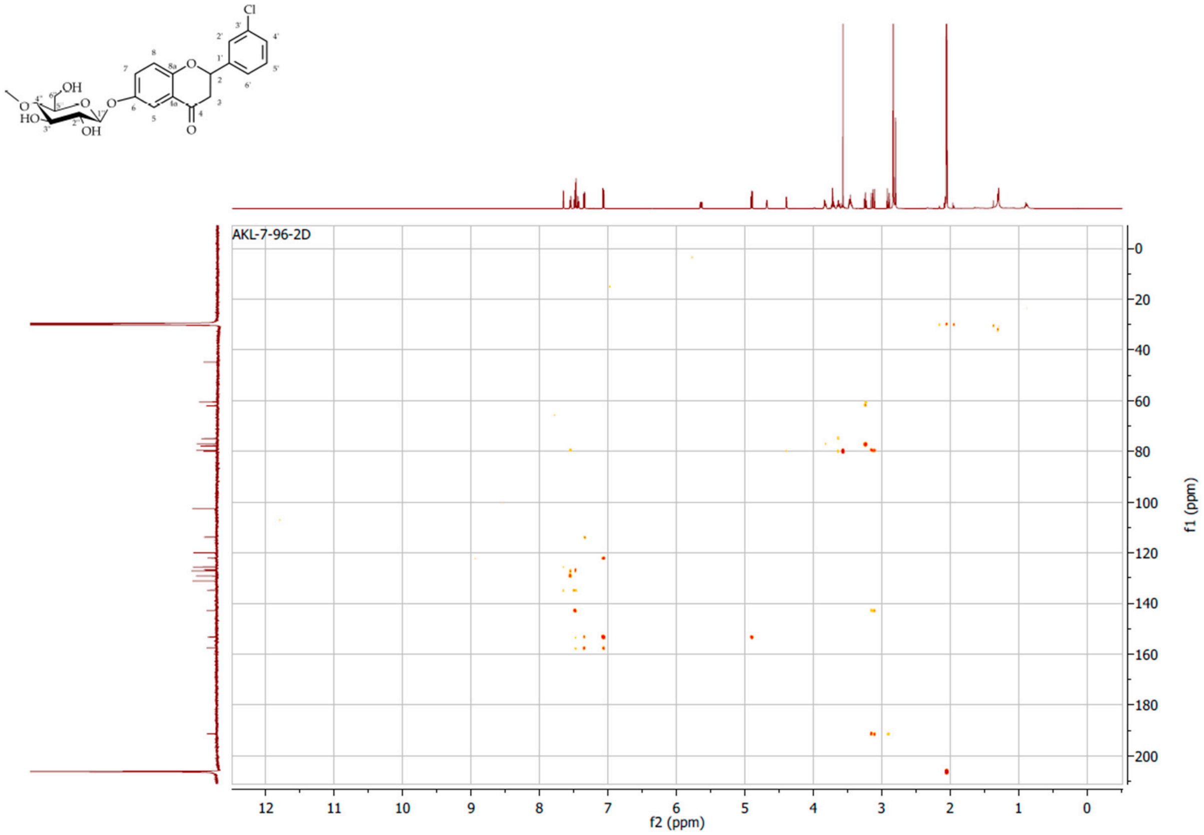

Figure S66. HMBC contour map – ^{1}H x ^{13}C of 3′-chloroflavanone 6-*O*-*β*-D-(4′′-*O*-methyl)-glucopyranoside (**2a**).

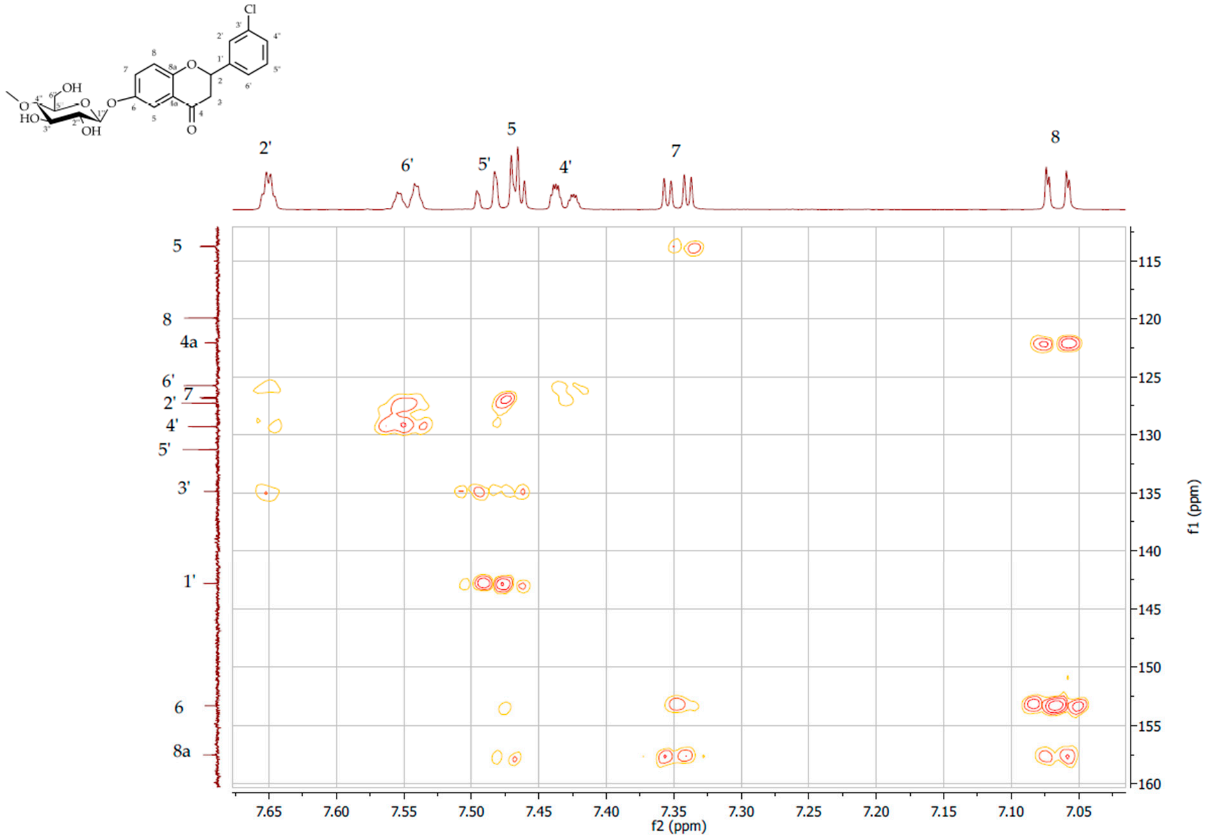

Figure S67. HMBC contour map – ^{1}H x ^{13}C expansion of 3′-chloroflavanone 6-*O*-*β*-D-(4′′-*O*-methyl)-glucopyranoside (**2a**).

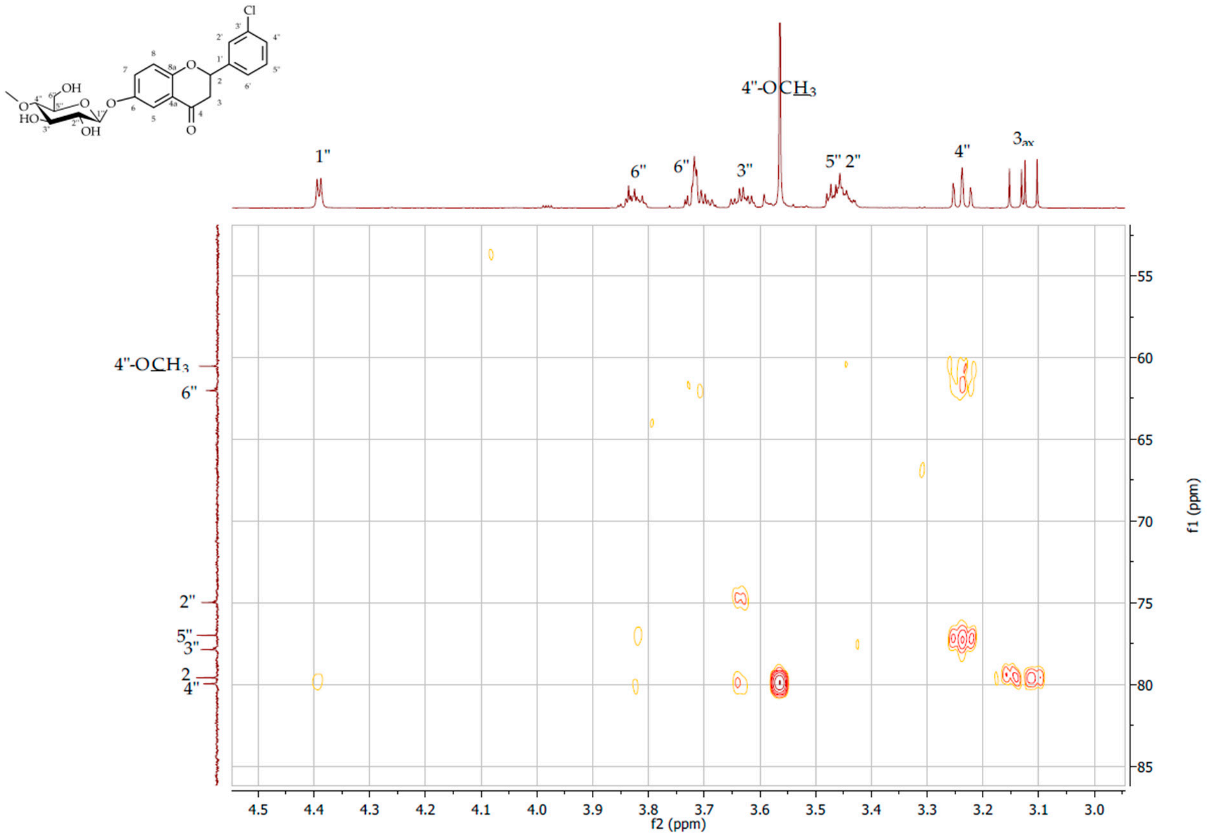

Figure S68. HMBC contour map – ^{1}H x ^{13}C expansion of 3′-chloroflavanone 6-*O*-*β*-D-(4′′-*O*-methyl)-glucopyranoside (**2a**).

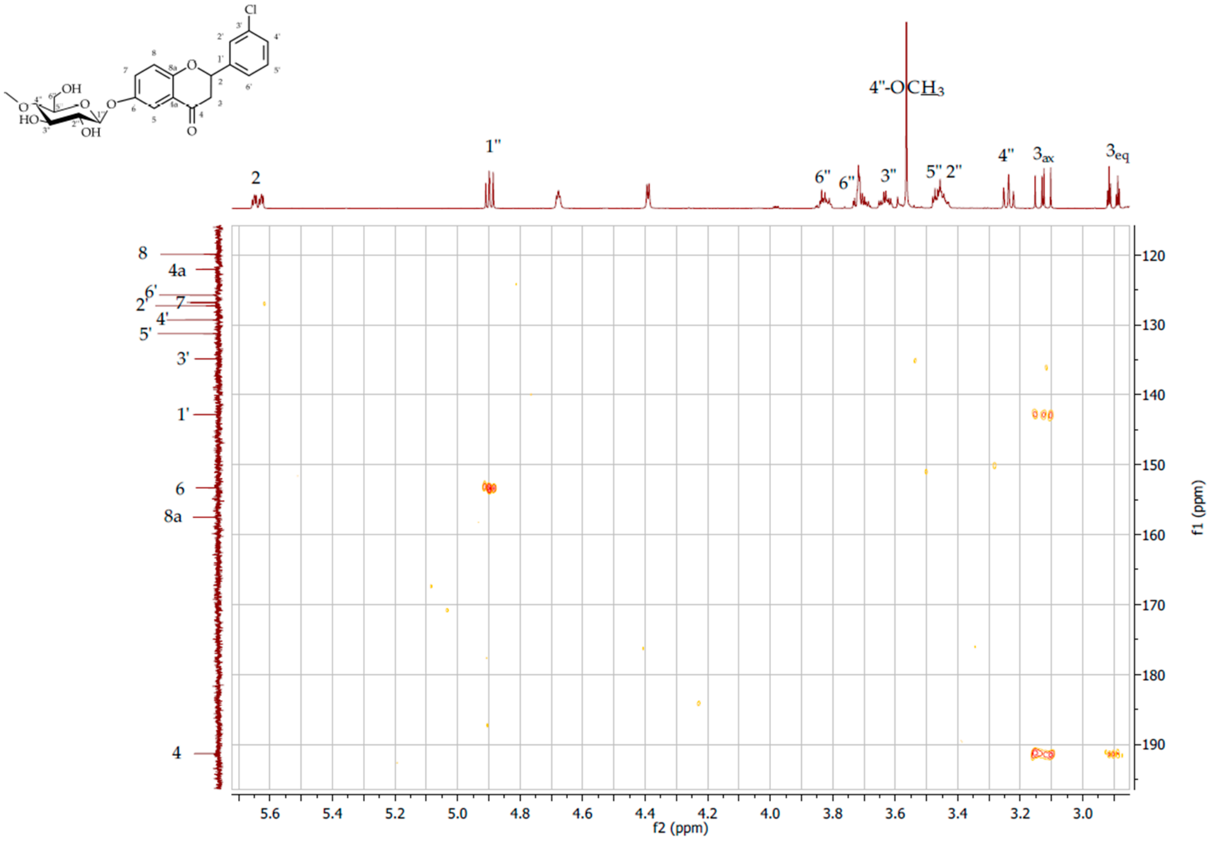

Figure S69. HMBC contour map – ^{1}H x ^{13}C expansion of 3′-chloroflavanone 4′-*O*-*β*-D-(4′′-*O*-methyl)-glucopyranoside (**2a**).

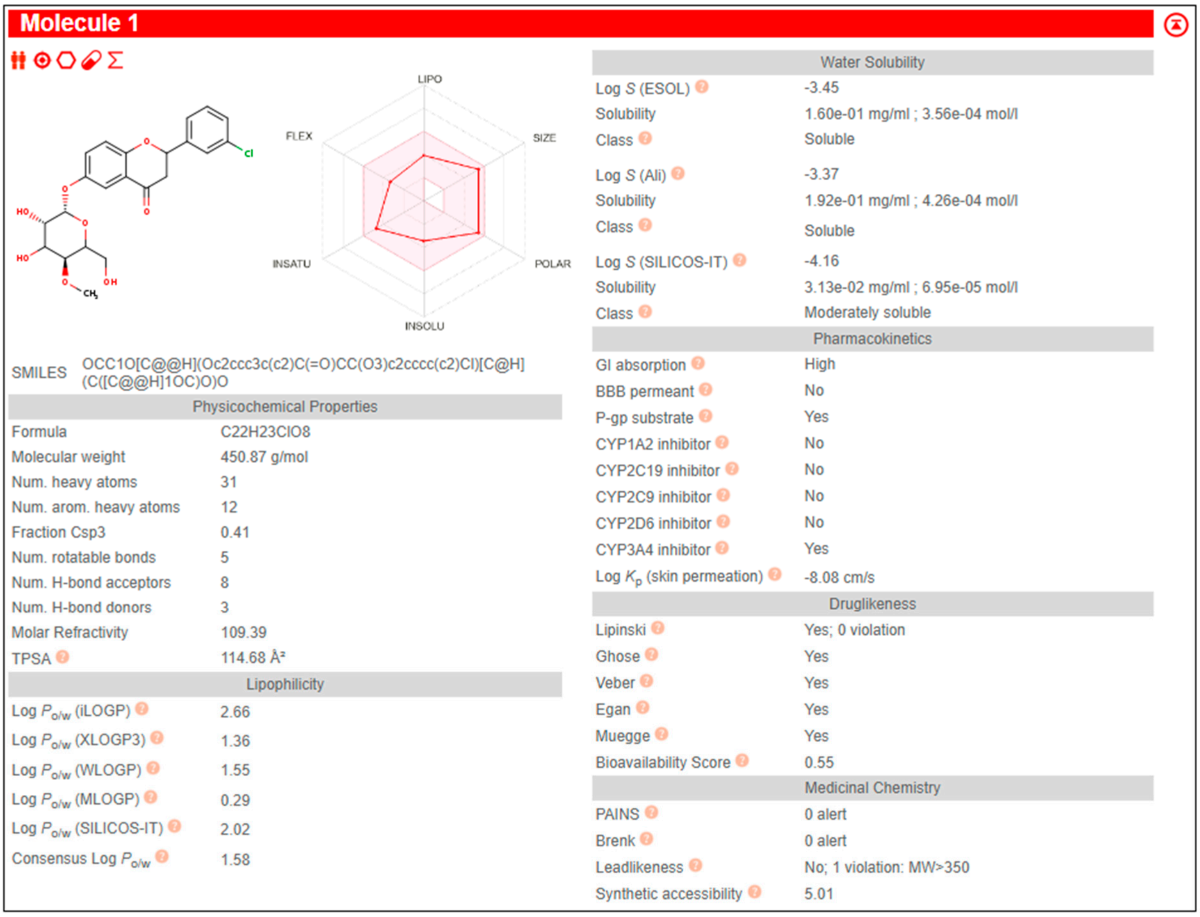

SMILES	OCC1O[C@@H](Oc2ccc3c(c2)C(=O)CC(O3)c2cccc(c2)Cl)[C@H](C([C@@H]1OC)O)O

Physicochemical Properties	
Formula	C22H23ClO8
Molecular weight	450.87 g/mol
Num. heavy atoms	31
Num. arom. heavy atoms	12
Fraction Csp3	0.41
Num. rotatable bonds	5
Num. H-bond acceptors	8
Num. H-bond donors	3
Molar Refractivity	109.39
TPSA	114.68 Å²

Lipophilicity	
Log $P_{o/w}$ (iLOGP)	2.66
Log $P_{o/w}$ (XLOGP3)	1.36
Log $P_{o/w}$ (WLOGP)	1.55
Log $P_{o/w}$ (MLOGP)	0.29
Log $P_{o/w}$ (SILICOS-IT)	2.02
Consensus Log $P_{o/w}$	1.58

Water Solubility	
Log *S* (ESOL)	-3.45
Solubility	1.60e-01 mg/ml ; 3.56e-04 mol/l
Class	Soluble
Log *S* (Ali)	-3.37
Solubility	1.92e-01 mg/ml ; 4.26e-04 mol/l
Class	Soluble
Log *S* (SILICOS-IT)	-4.16
Solubility	3.13e-02 mg/ml ; 6.95e-05 mol/l
Class	Moderately soluble

Pharmacokinetics	
GI absorption	High
BBB permeant	No
P-gp substrate	Yes
CYP1A2 inhibitor	No
CYP2C19 inhibitor	No
CYP2C9 inhibitor	No
CYP2D6 inhibitor	No
CYP3A4 inhibitor	Yes
Log K_p (skin permeation)	-8.08 cm/s

Druglikeness	
Lipinski	Yes; 0 violation
Ghose	Yes
Veber	Yes
Egan	Yes
Muegge	Yes
Bioavailability Score	0.55

Medicinal Chemistry	
PAINS	0 alert
Brenk	0 alert
Leadlikeness	No; 1 violation: MW>350
Synthetic accessibility	5.01

Figure S70. 3′-Chloroflavanone 6-*O*-*β*-D-(4′′-*O*-methyl)-glucopyranoside (**2a**) physicochemical and ADME parameters prediction using the SwissADME modelling.

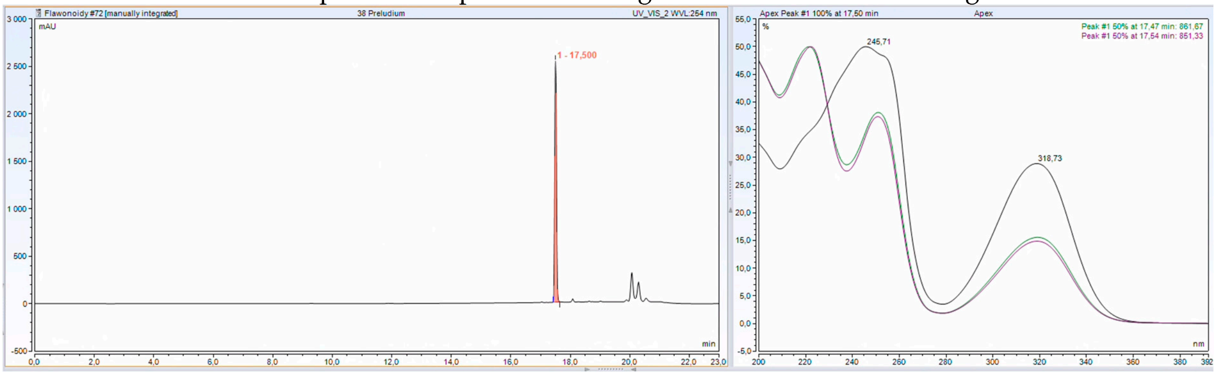

Figure S71. HPLC analysis of 4′-chloroflavanone (**3**).

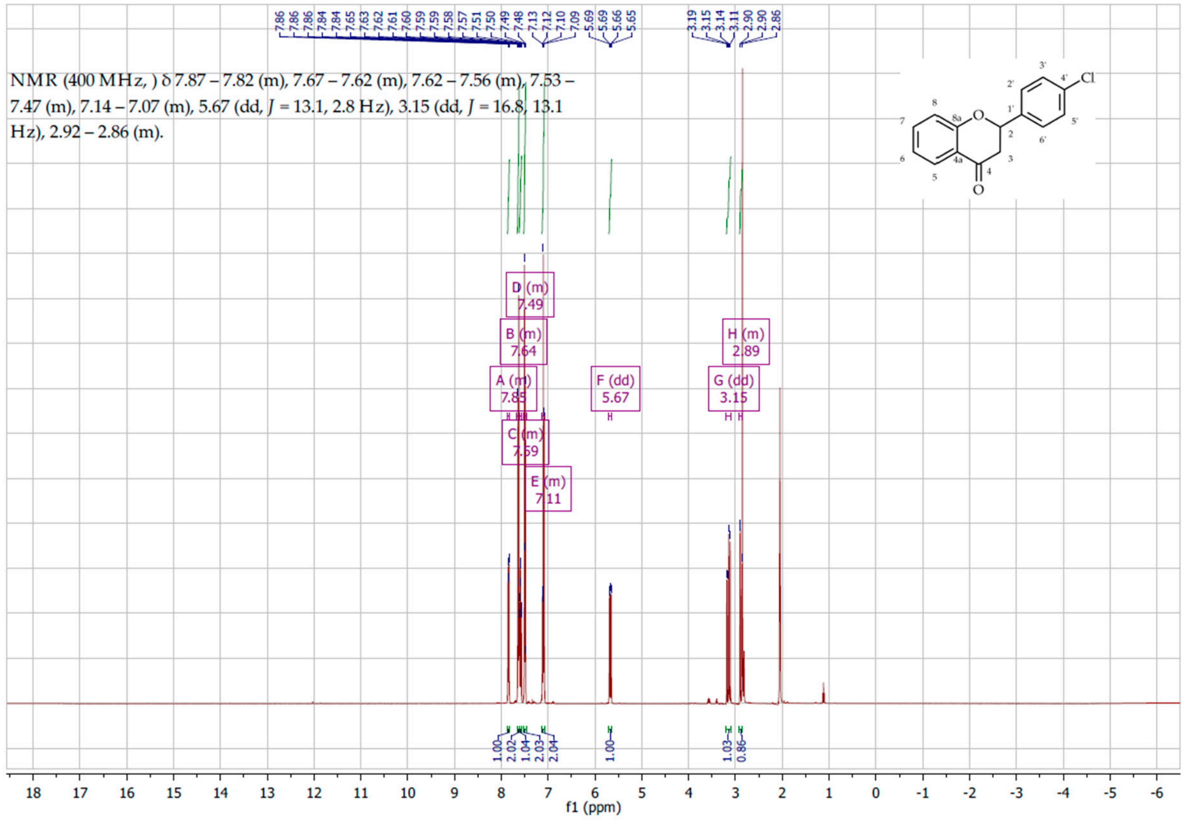

Figure S72. ^{1}H NMR spectrum (*δ*, acetone-d6, 600 MHz) of 4′-chloroflavanone (**3**).

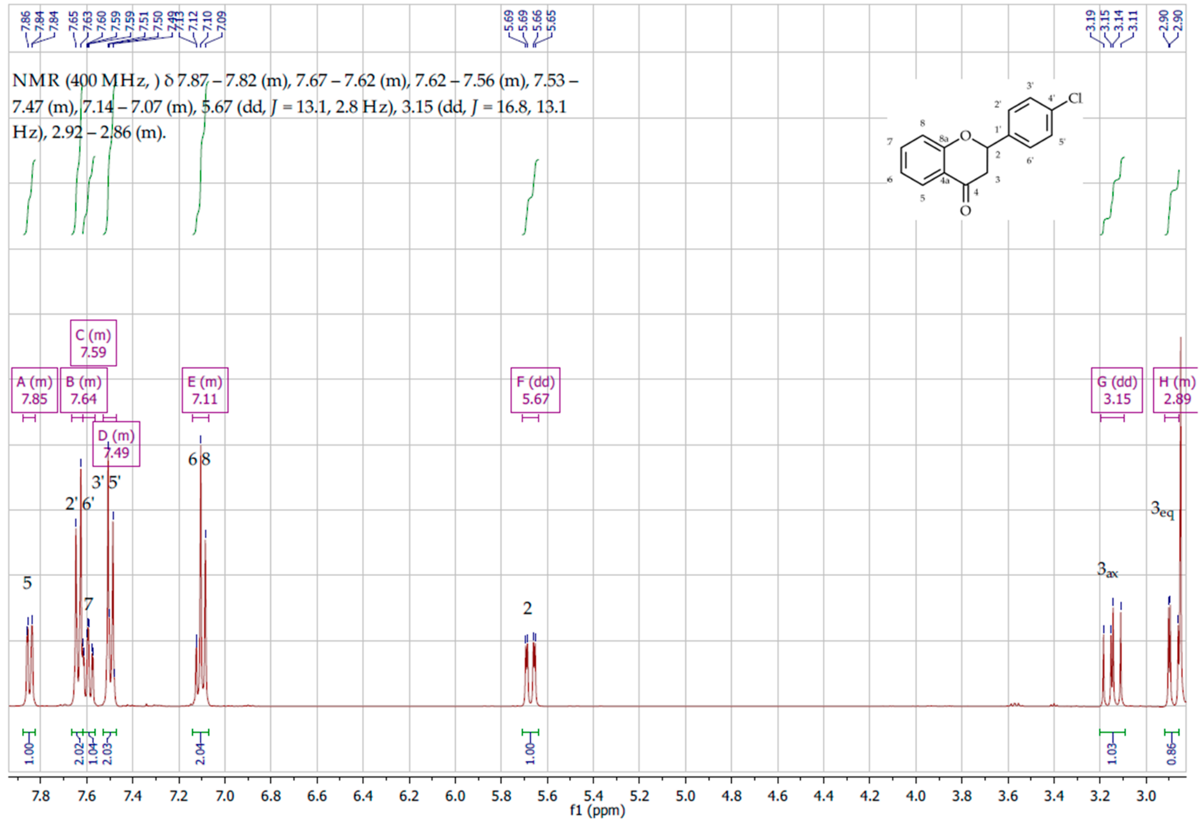

Figure S73. ^{1}H NMR spectrum expansion (*δ*, acetone-d6, 600 MHz) of 4′-chloroflavanone (**3**).

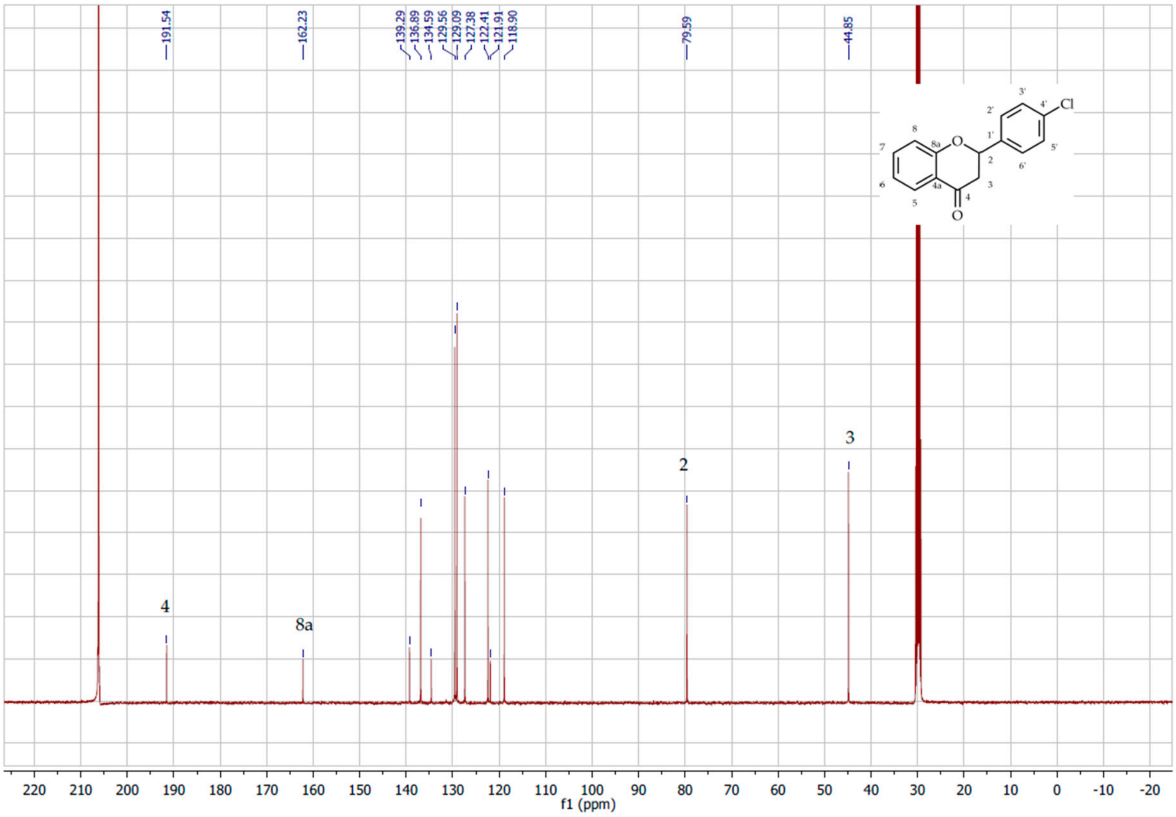

Figure S74. ^{13}C NMR spectrum (δ, acetone-d6, 151 MHz) of 4′-chloroflavanone (**3**).

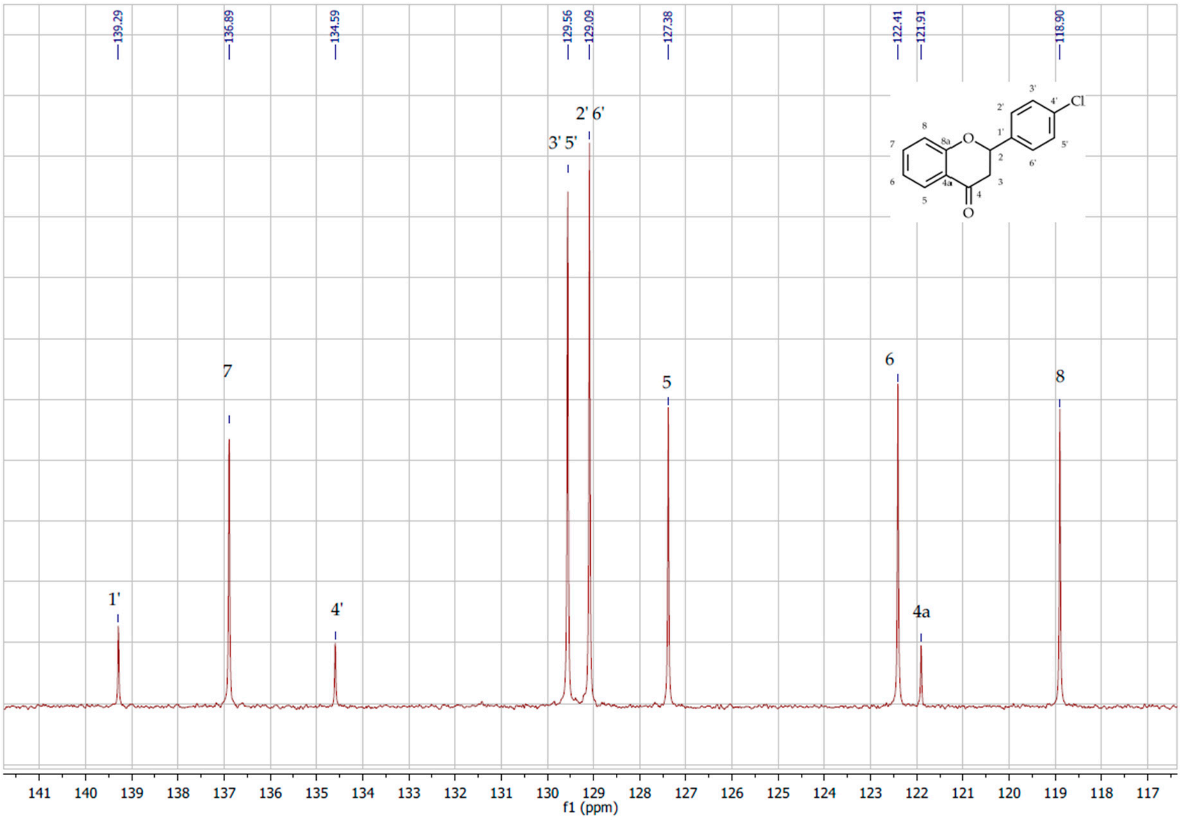

Figure S75. ^{13}C NMR spectrum expansion (δ, acetone-d6, 151 MHz) of 4′-chloroflavanone (**3**).

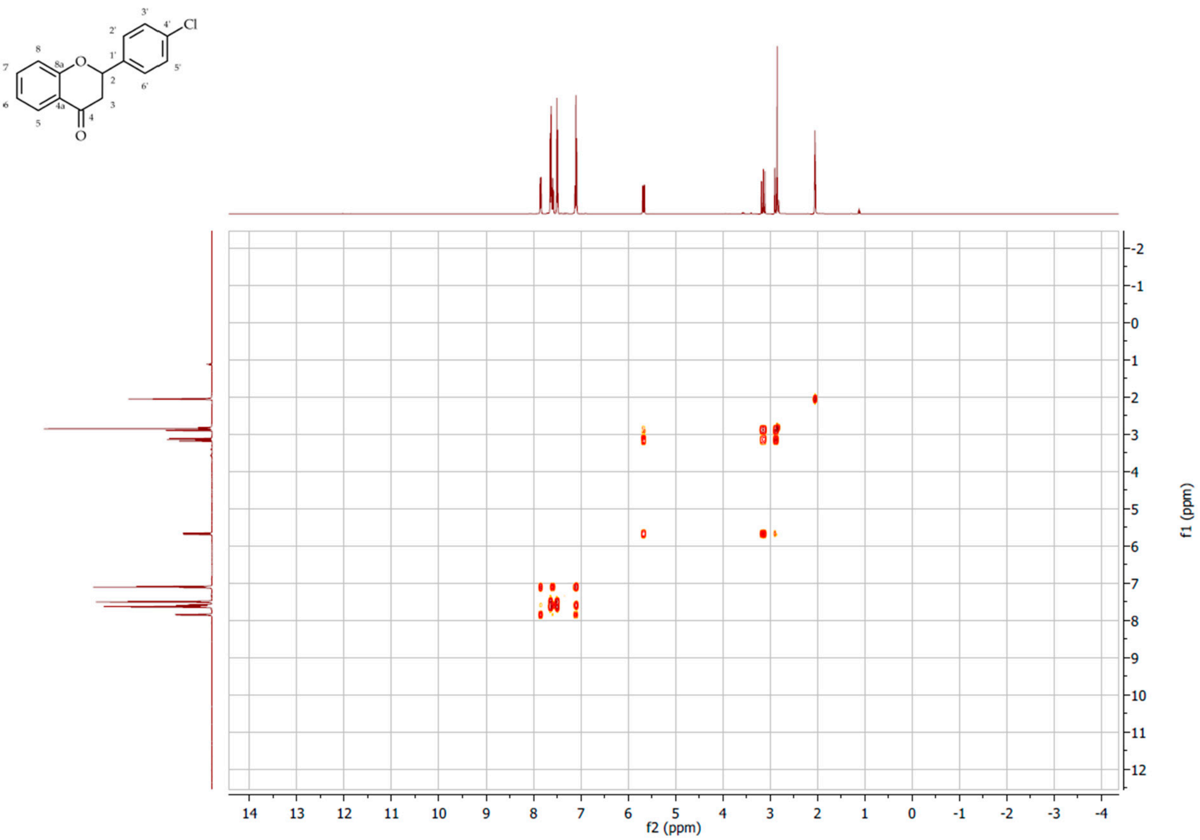

Figure S76. COSY contour map – $^{1}H \times {}^{1}H$ of 4′-chloroflavanone (**3**).

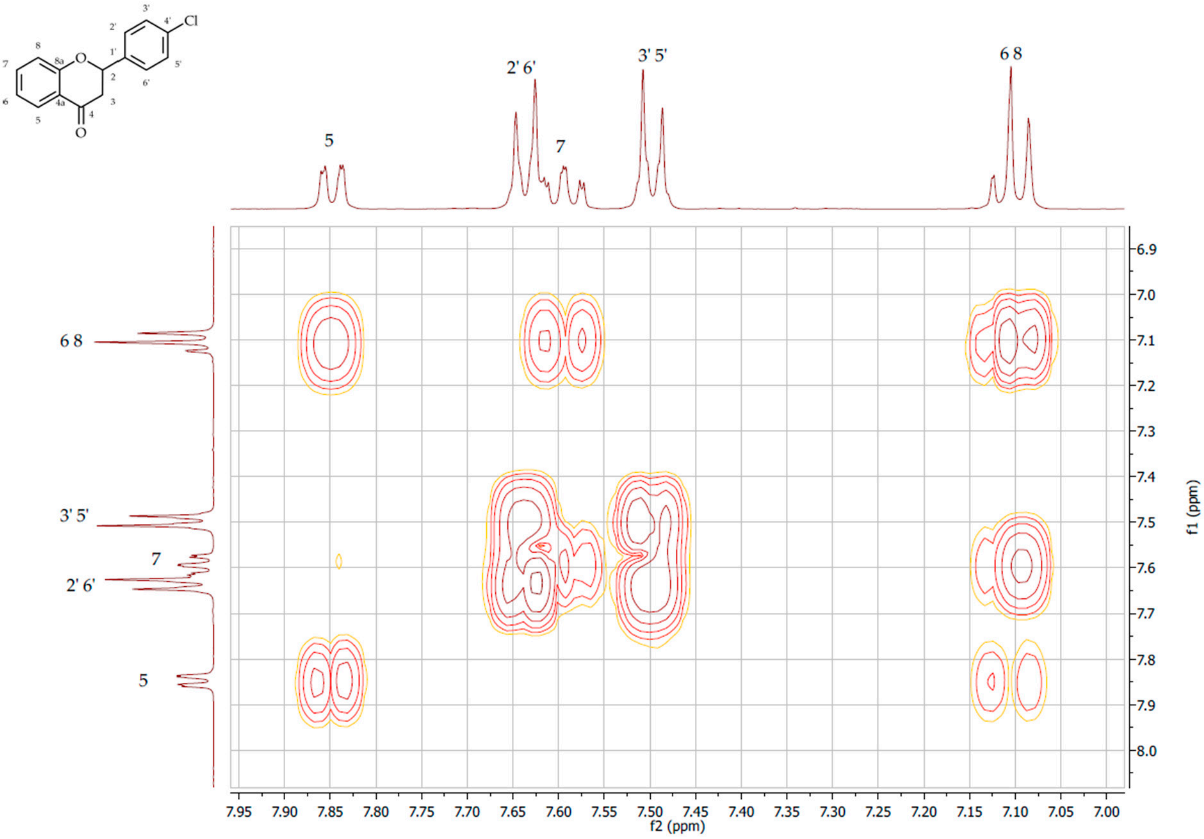

Figure S77. COSY contour map – $^{1}H \times {}^{1}H$ expansion of 4′-chloroflavanone (**3**).

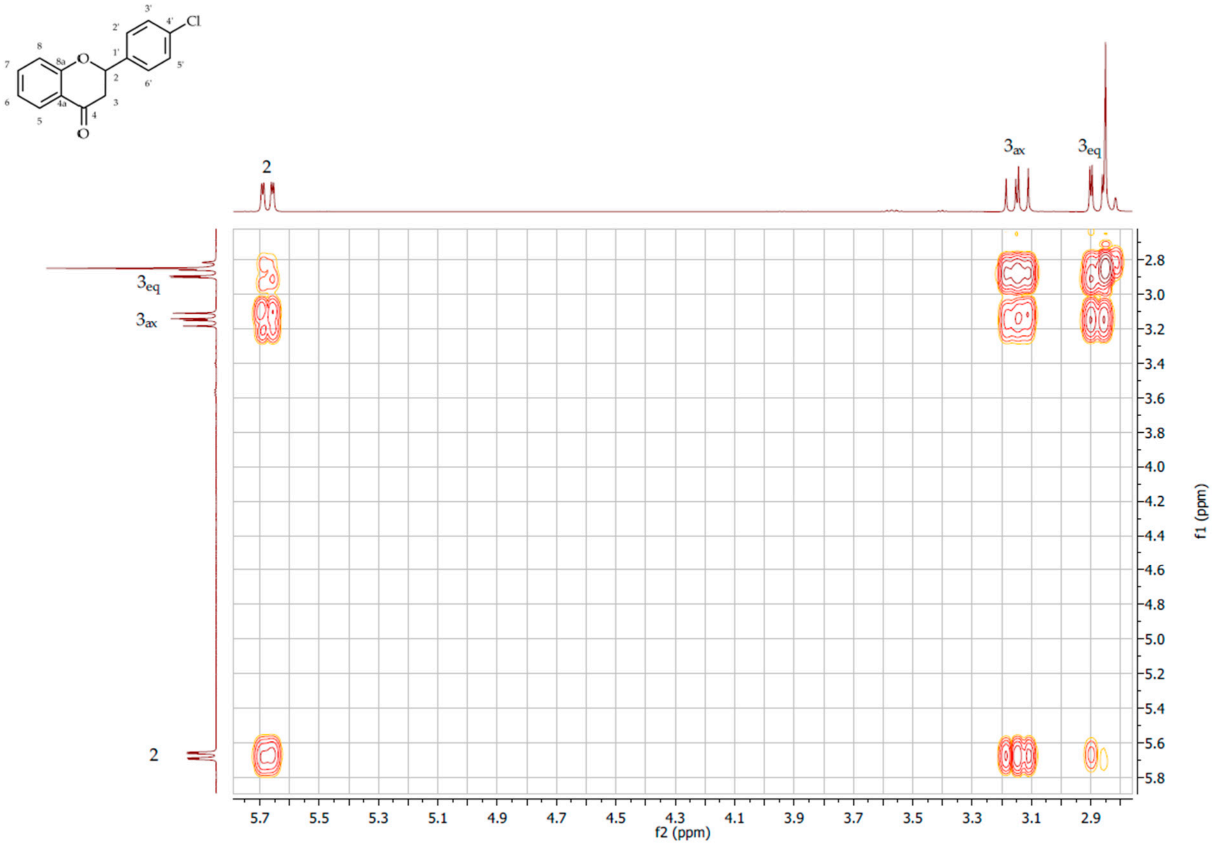

Figure S78. COSY contour map – ^{1}H x ^{1}H expansion of 4′-chloroflavanone (**3**).

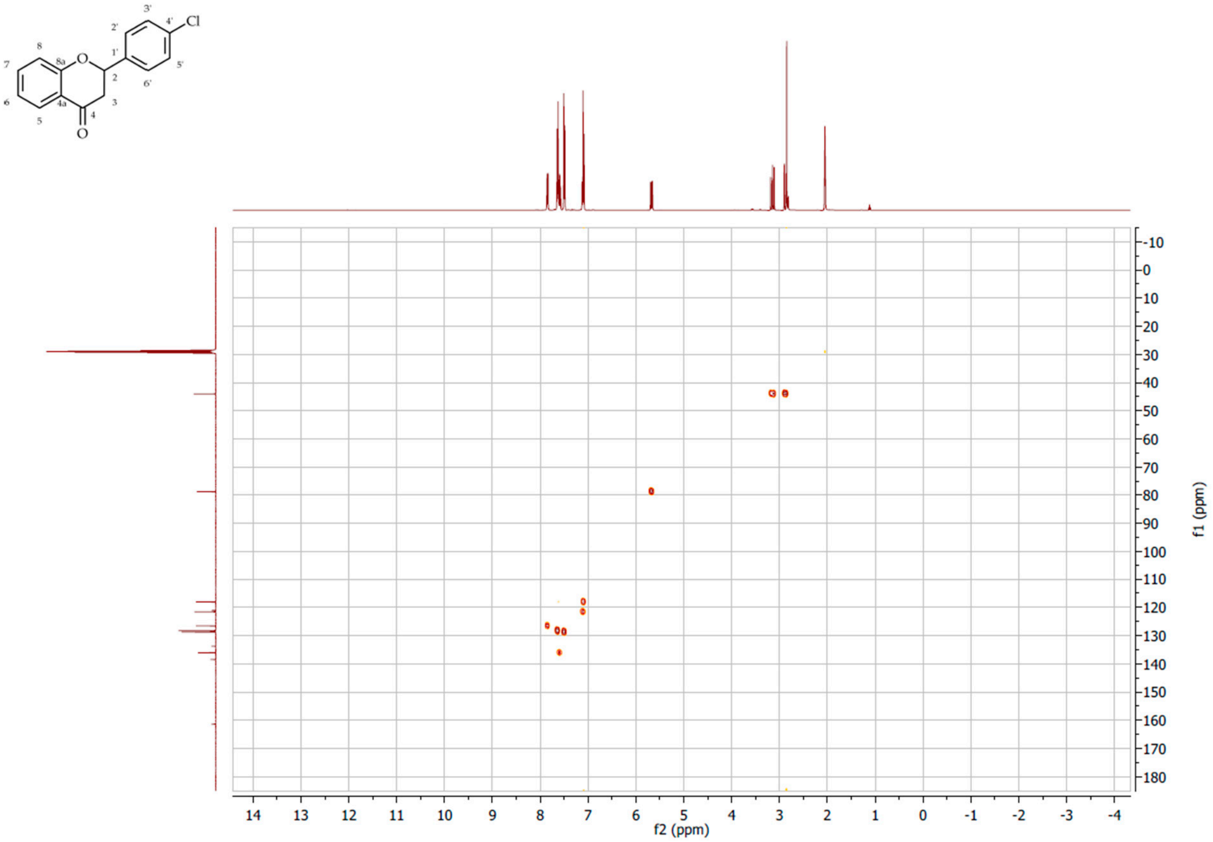

Figure S79. HMQC contour map – ^{1}H x ^{13}C of 4′-chloroflavanone (**3**).

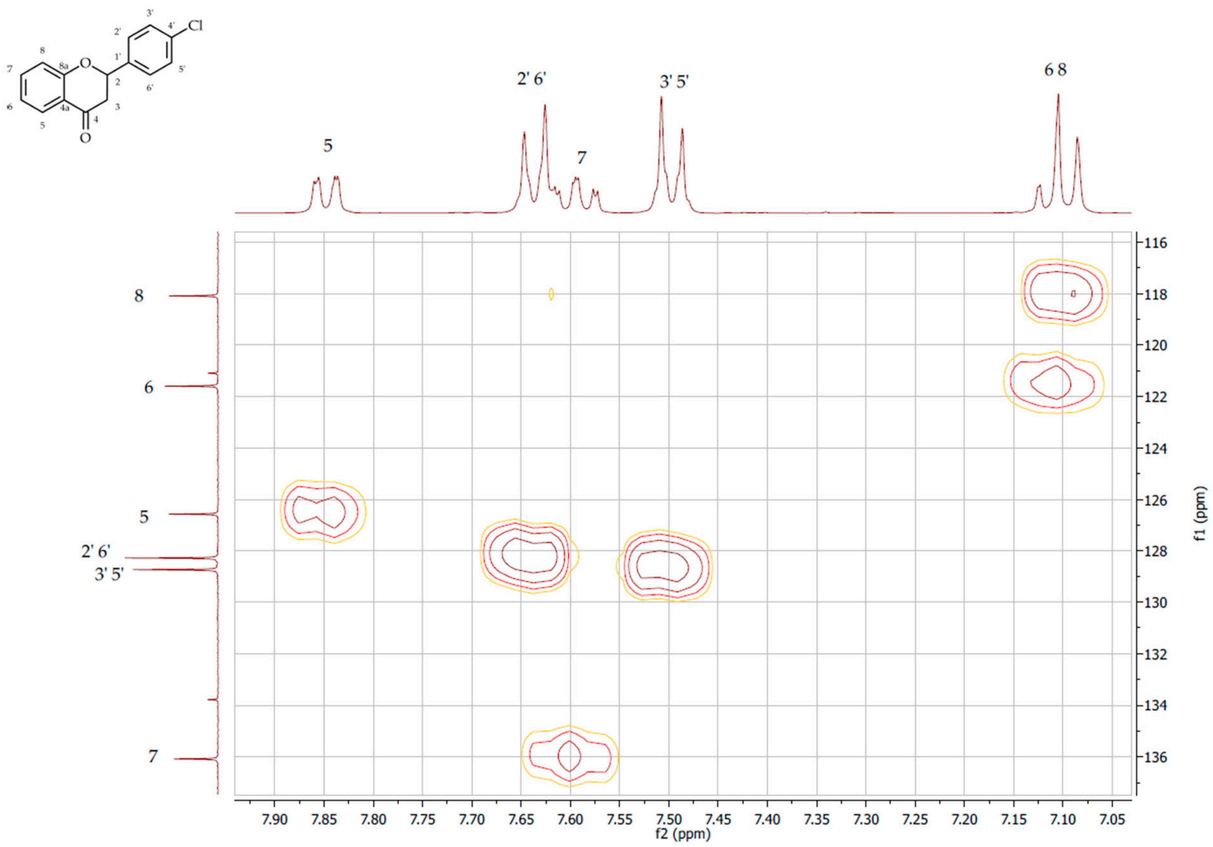

Figure S80. HMQC contour map – ^{1}H x ^{13}C expansion of 4′-chloroflavanone (**3**).

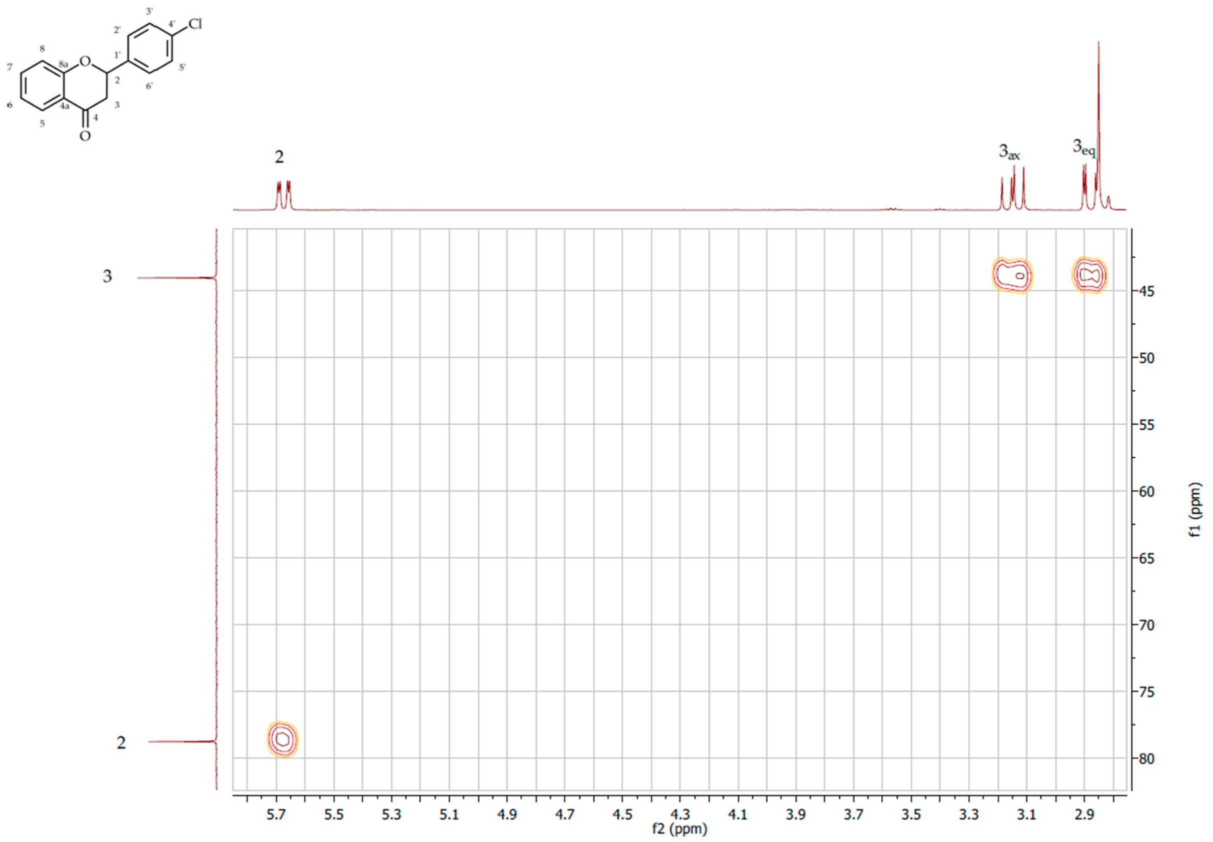

Figure S81. HMQC contour map – ^{1}H x ^{13}C expansion of 4′-chloroflavanone (**3**).

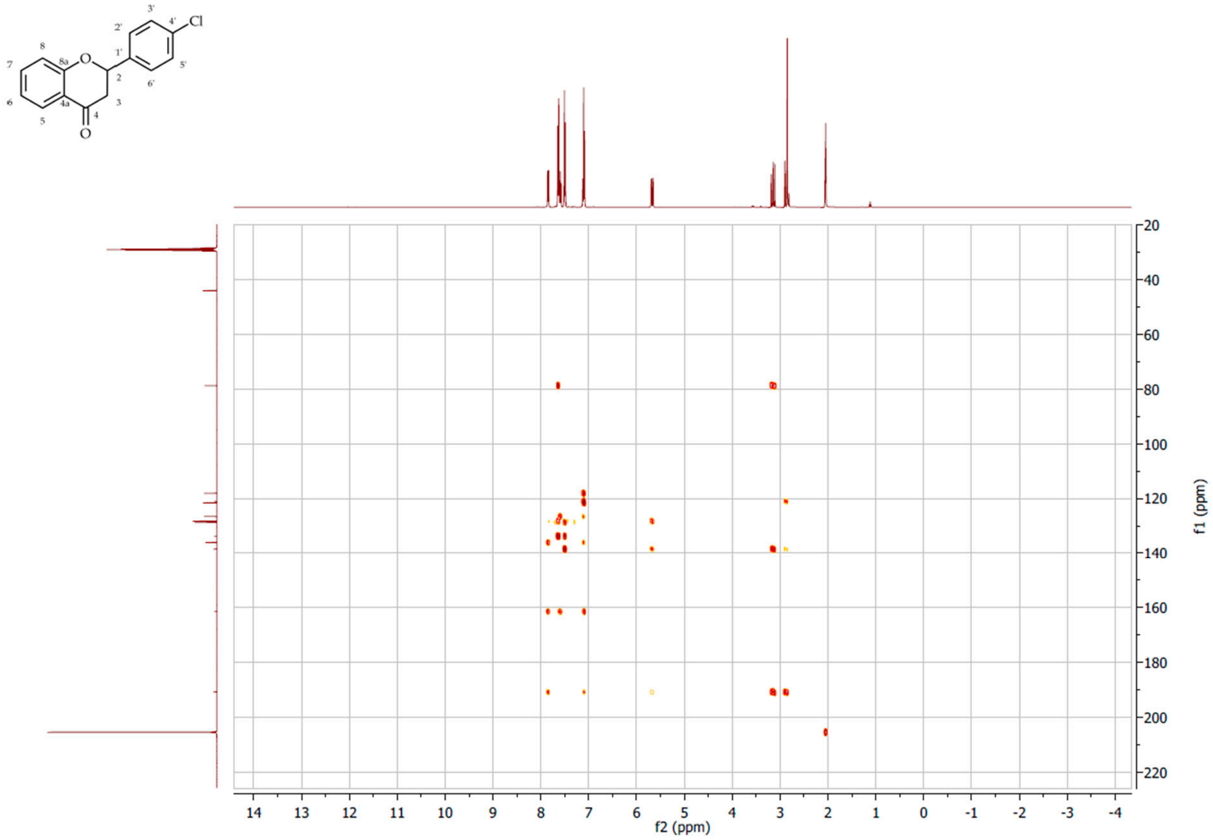

Figure S82. HMBC contour map – ^{1}H x ^{13}C of 4′-chloroflavanone (**3**).

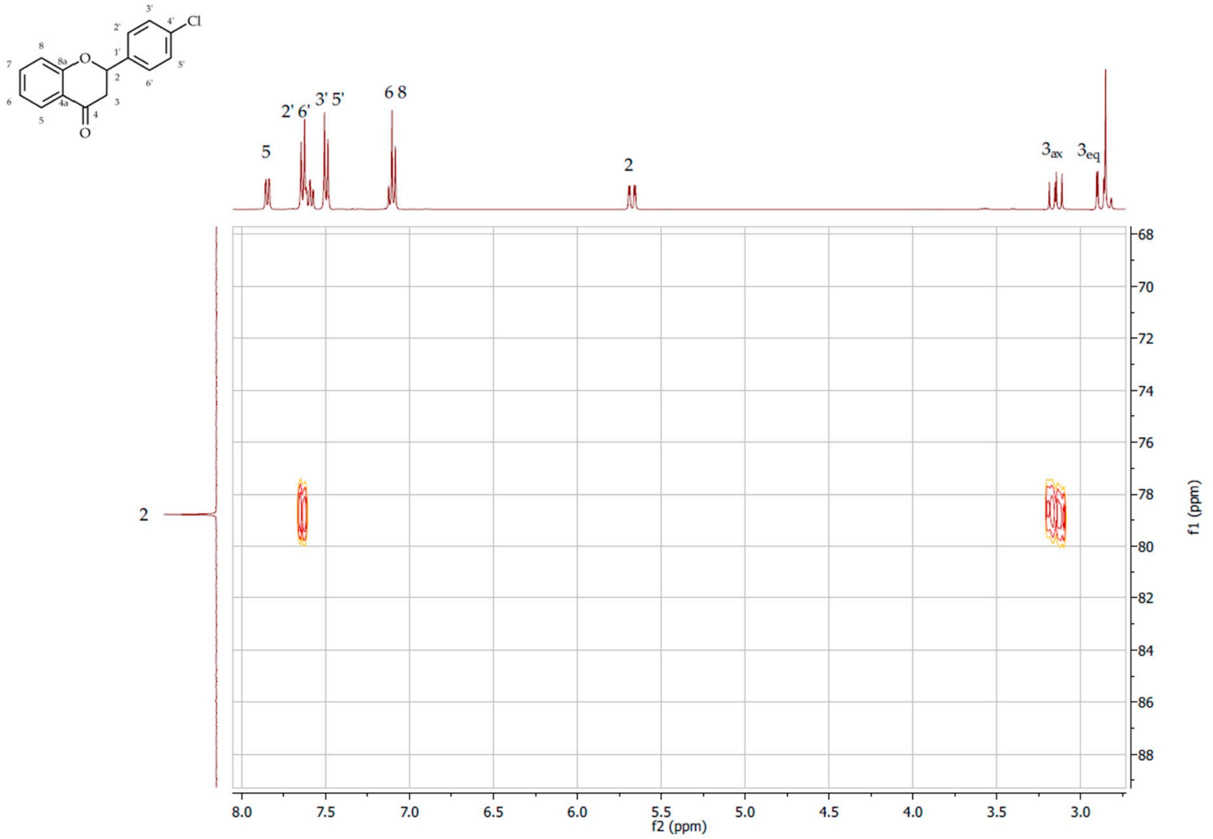

Figure S83. HMBC contour map – ^{1}H x ^{13}C expansion of 4′-chloroflavanone (**3**).

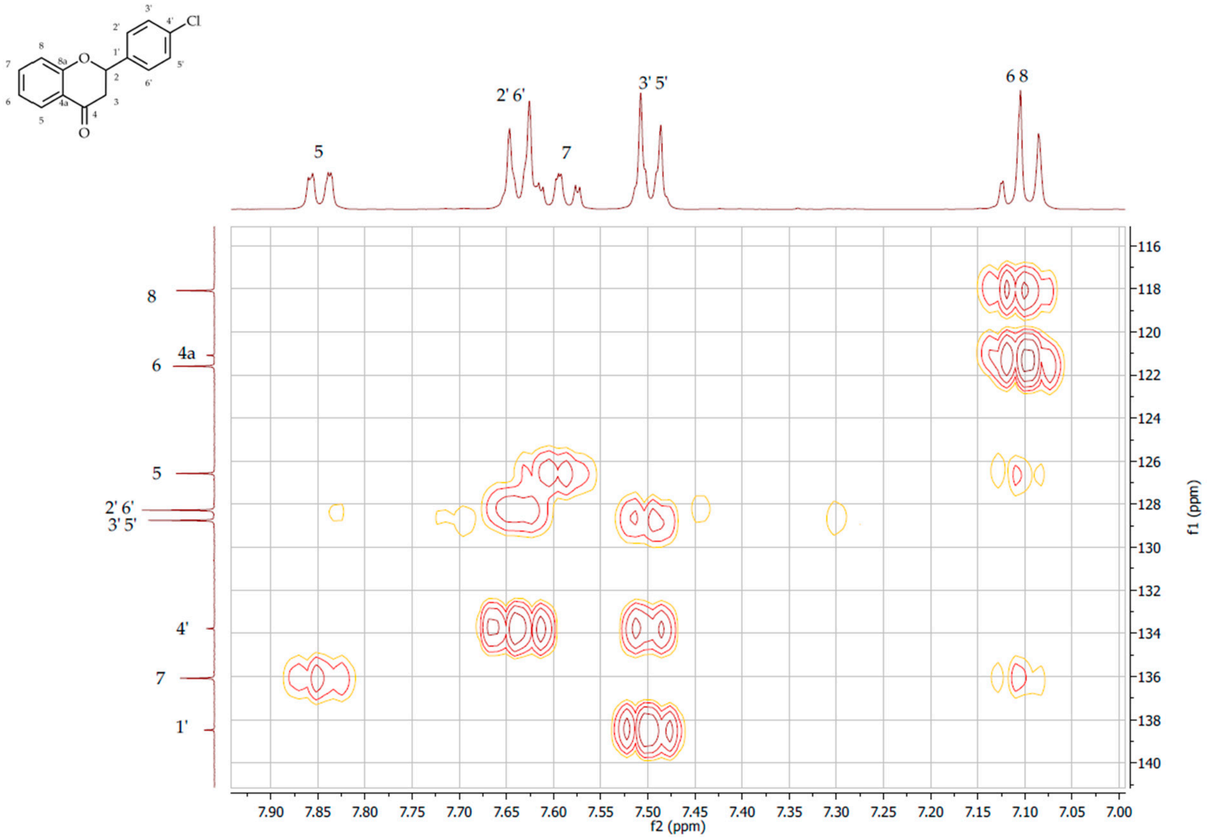

Figure S84. HMBC contour map – ^{1}H x ^{13}C expansion of 4′-chloroflavanone (**3**).

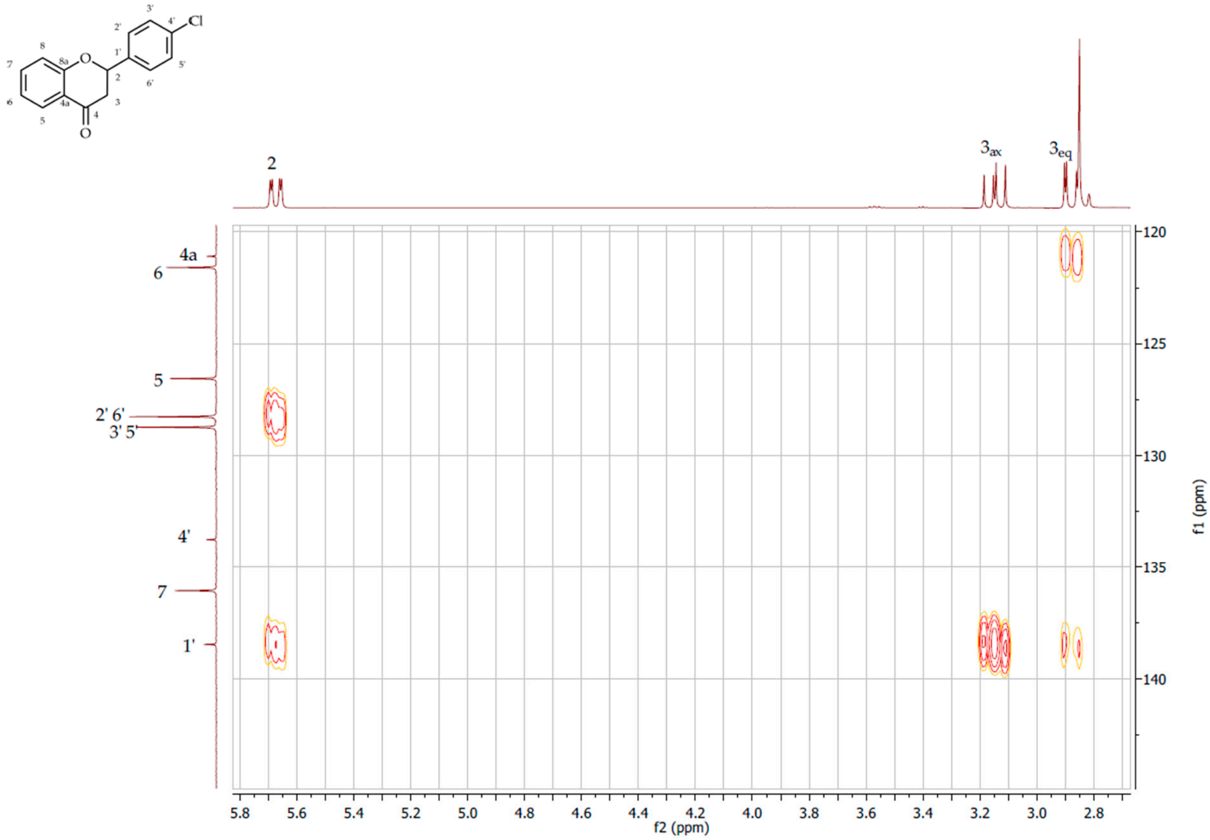

Figure S85. HMBC contour map – ^{1}H x ^{13}C expansion of 4′-chloroflavanone (**3**).

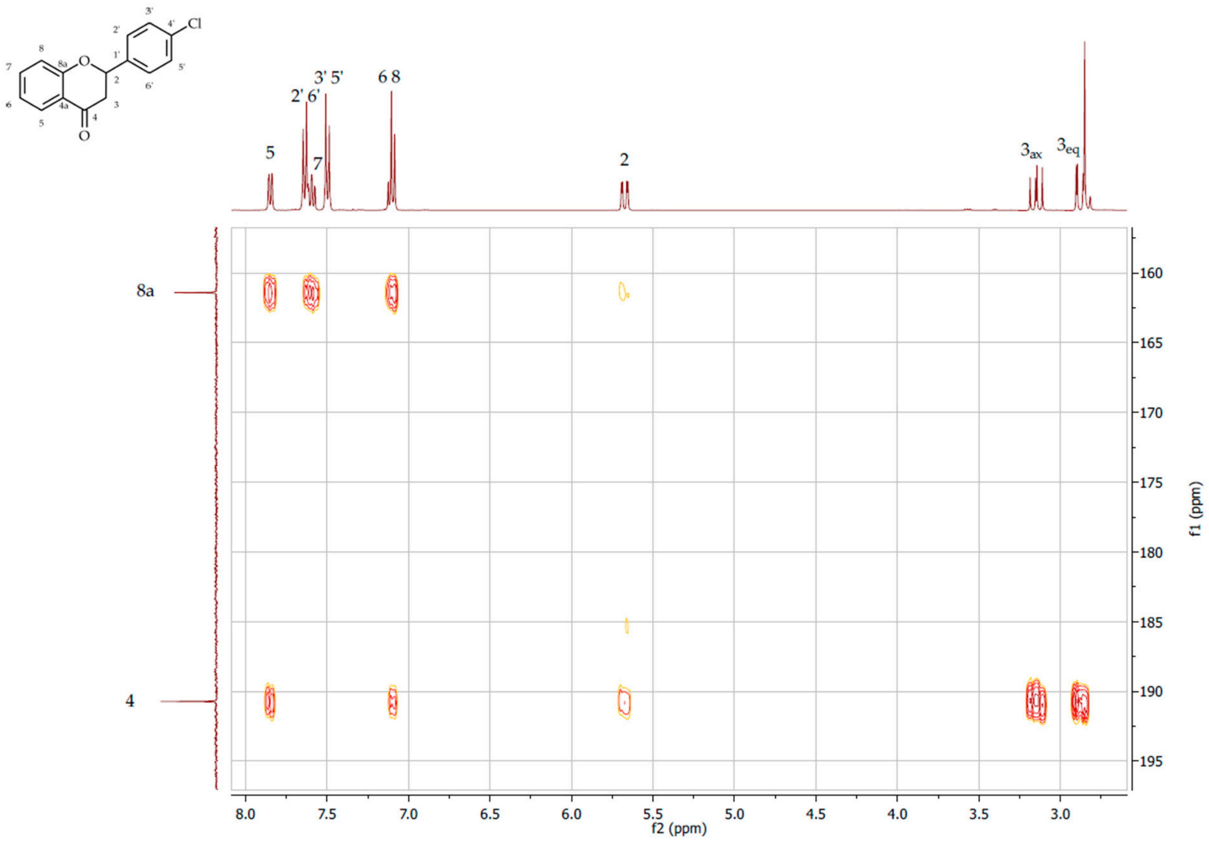

Figure S86. HMBC contour map – ^{1}H x ^{13}C expansion of 4′-chloroflavanone (**3**).

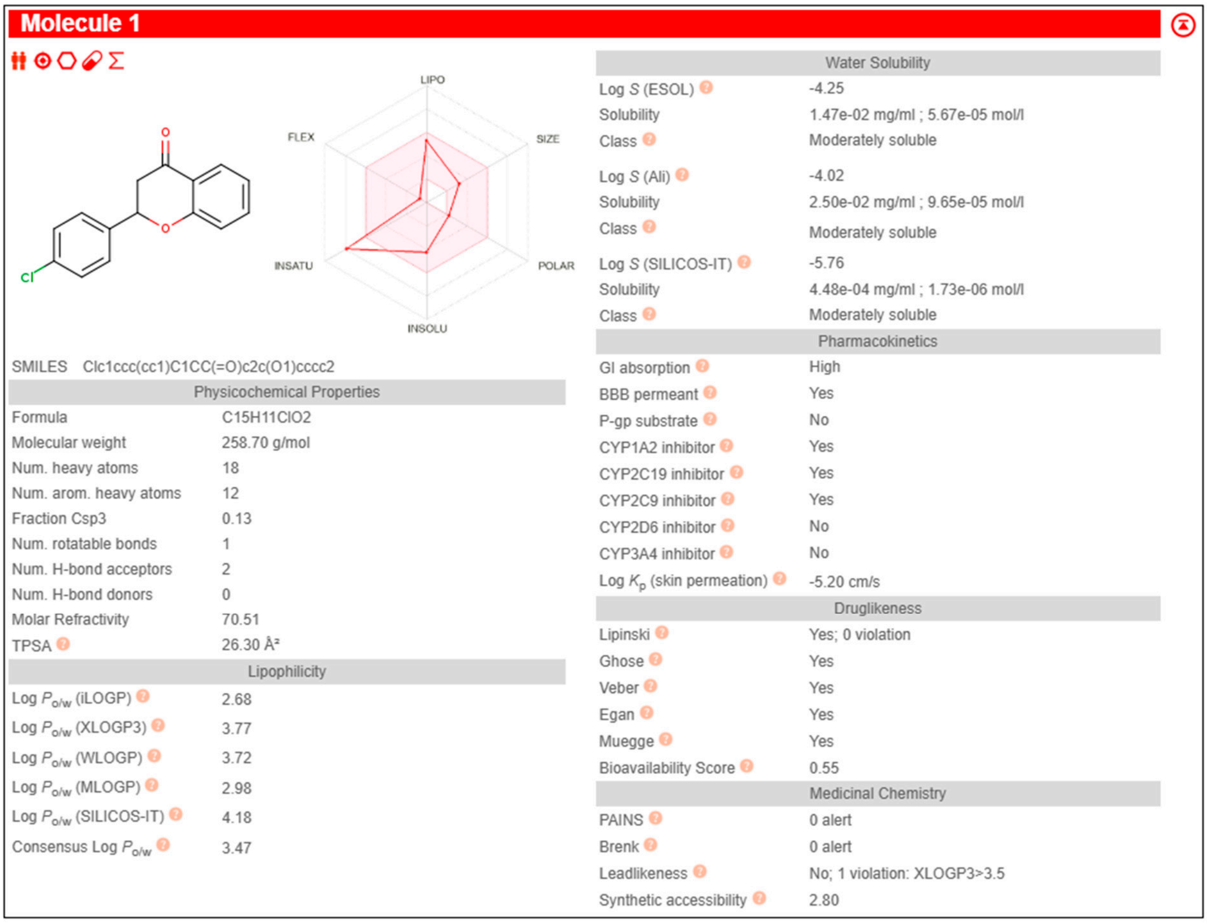

SMILES Clc1ccc(cc1)C1CC(=O)c2c(O1)cccc2

Physicochemical Properties	
Formula	C15H11ClO2
Molecular weight	258.70 g/mol
Num. heavy atoms	18
Num. arom. heavy atoms	12
Fraction Csp3	0.13
Num. rotatable bonds	1
Num. H-bond acceptors	2
Num. H-bond donors	0
Molar Refractivity	70.51
TPSA	26.30 Å²

Lipophilicity	
Log $P_{o/w}$ (iLOGP)	2.68
Log $P_{o/w}$ (XLOGP3)	3.77
Log $P_{o/w}$ (WLOGP)	3.72
Log $P_{o/w}$ (MLOGP)	2.98
Log $P_{o/w}$ (SILICOS-IT)	4.18
Consensus Log $P_{o/w}$	3.47

Water Solubility	
Log *S* (ESOL)	-4.25
Solubility	1.47e-02 mg/ml ; 5.67e-05 mol/l
Class	Moderately soluble
Log *S* (Ali)	-4.02
Solubility	2.50e-02 mg/ml ; 9.65e-05 mol/l
Class	Moderately soluble
Log *S* (SILICOS-IT)	-5.76
Solubility	4.48e-04 mg/ml ; 1.73e-06 mol/l
Class	Moderately soluble

Pharmacokinetics	
GI absorption	High
BBB permeant	Yes
P-gp substrate	No
CYP1A2 inhibitor	Yes
CYP2C19 inhibitor	Yes
CYP2C9 inhibitor	Yes
CYP2D6 inhibitor	No
CYP3A4 inhibitor	No
Log K_p (skin permeation)	-5.20 cm/s

Druglikeness	
Lipinski	Yes; 0 violation
Ghose	Yes
Veber	Yes
Egan	Yes
Muegge	Yes
Bioavailability Score	0.55

Medicinal Chemistry	
PAINS	0 alert
Brenk	0 alert
Leadlikeness	No; 1 violation: XLOGP3>3.5
Synthetic accessibility	2.80

Figure S87. 4′-Chloroflavanone (**3**) physicochemical and ADME parameters prediction using the SwissADME modelling.

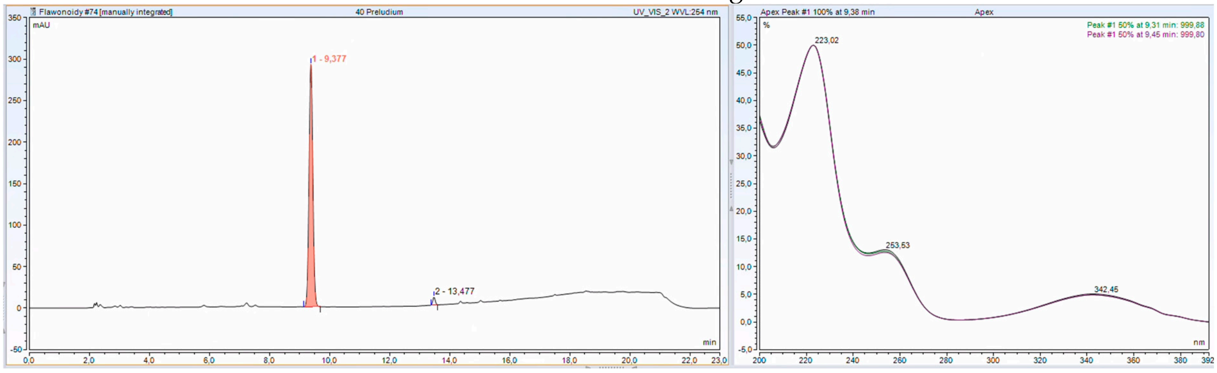

Figure S88. HPLC analysis of 4′-chloroflavanone 6-*O*-*β*-D-(4′′-*O*-methyl)-glucopyranoside (**3a**).

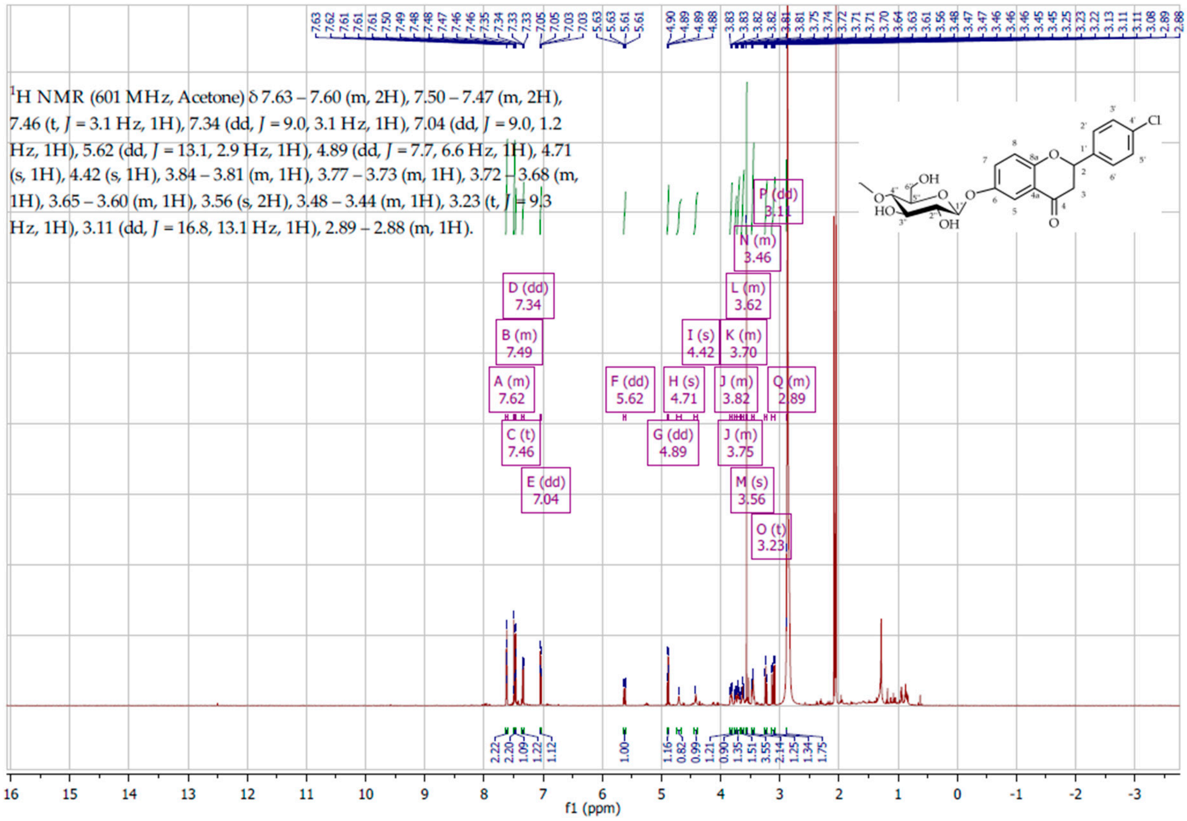

Figure S89. ¹H NMR spectrum (δ, acetone-d6, 600 MHz) of 4′-chloroflavanone 6-*O*-*β*-D-(4′′-*O*-methyl)-glucopyranoside (**3a**).

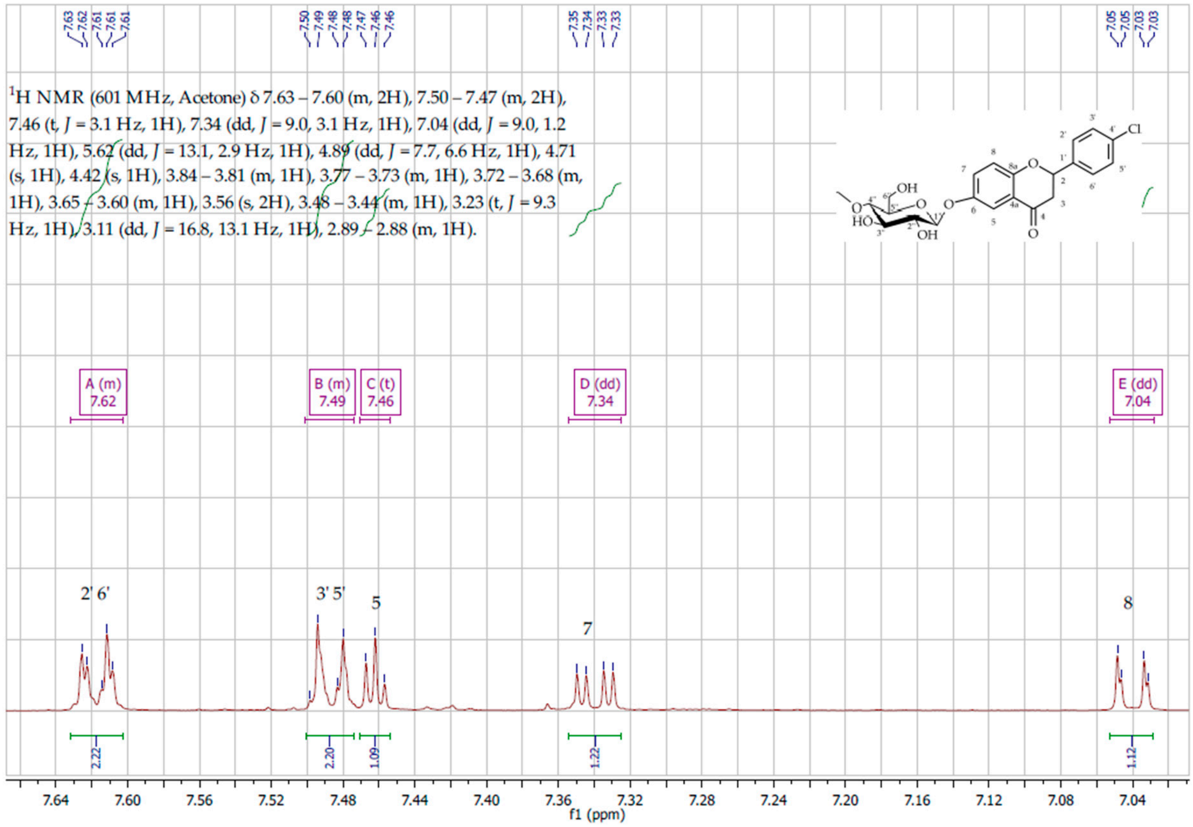

Figure S90. ^{1}H NMR spectrum expansion (*δ*, acetone-d6, 600 MHz) of 4′-chloroflavanone 6-*O*-*β*-D-(4′′-*O*-methyl)-glucopyranoside (**3a**).

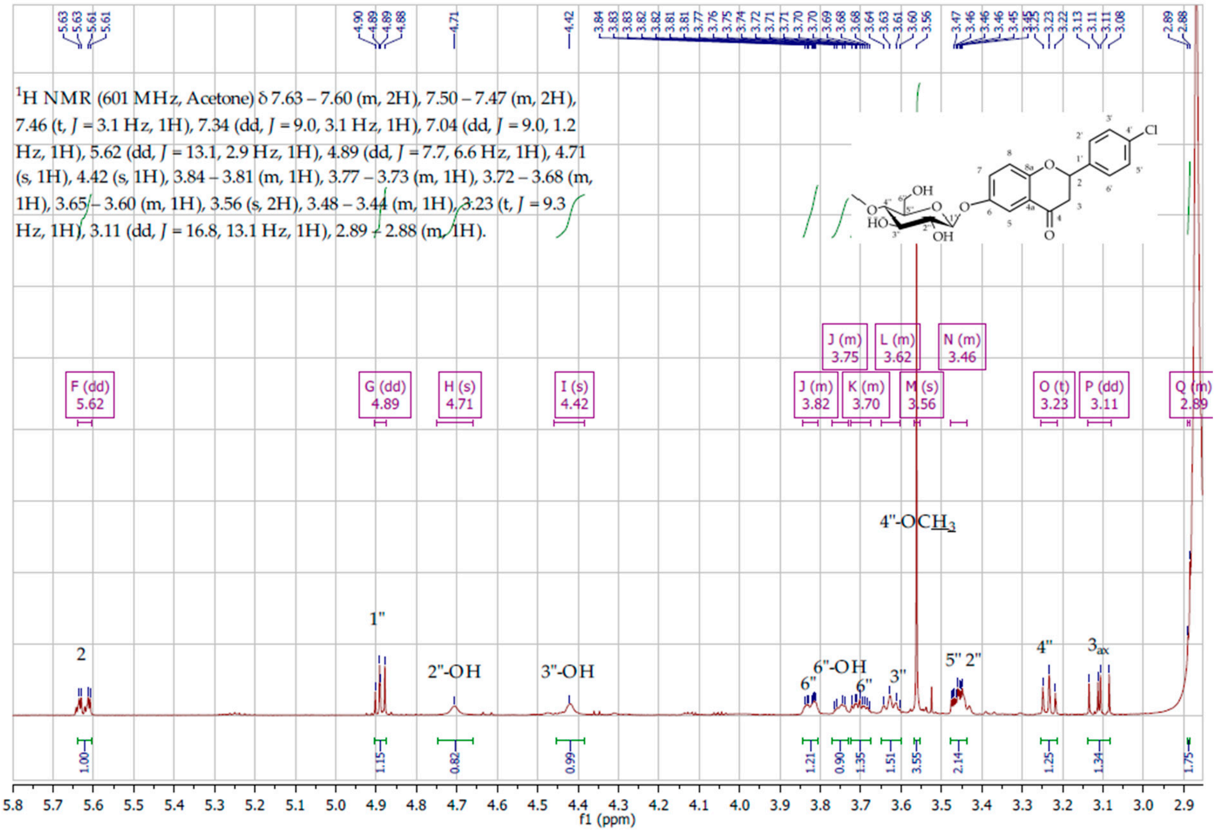

Figure S91. ^{1}H NMR spectrum expansion (*δ*, acetone-d6, 600 MHz) of 4′-chloroflavanone 6-*O*-*β*-D-(4′′-*O*-methyl)-glucopyranoside (**3a**).

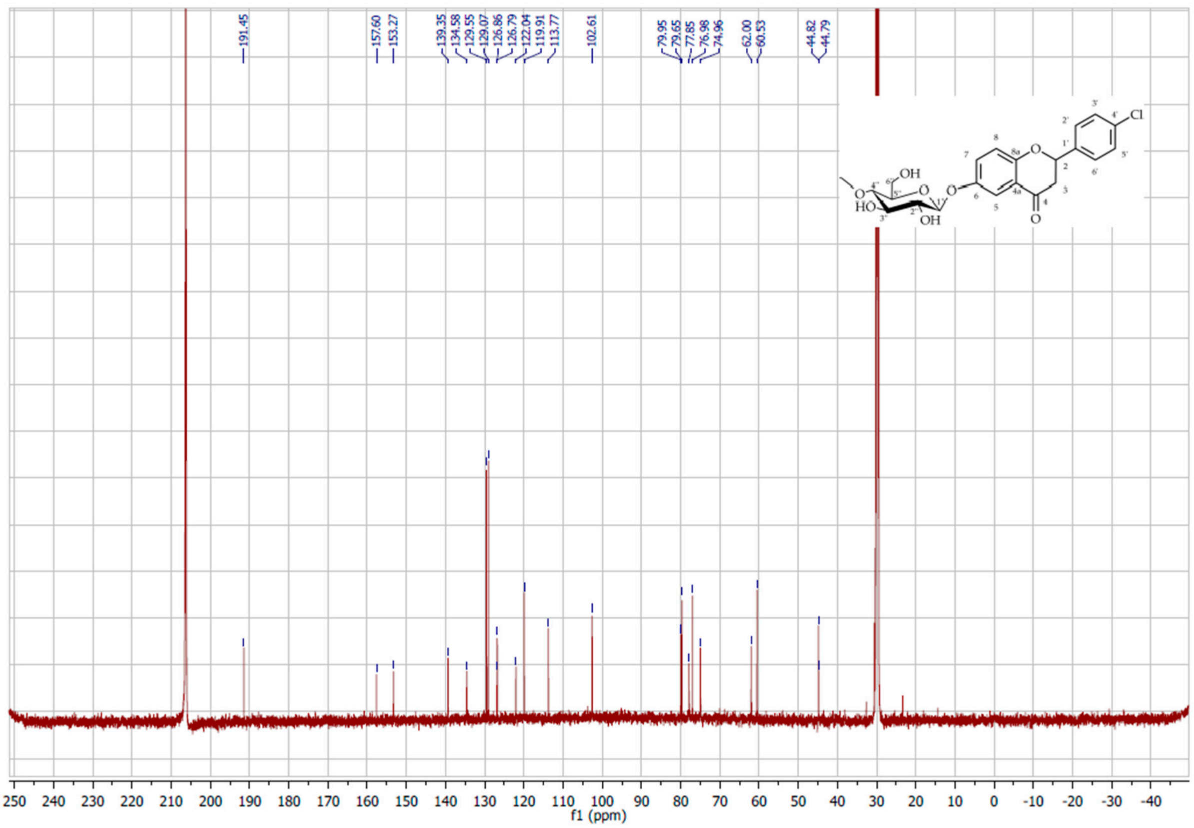

Figure S92. ^{13}C NMR spectrum (*δ,* acetone-d6, 151 MHz) of 4′-chloroflavanone 6-*O*-*β*-D-(4′′-*O*-methyl)-glucopyranoside (**3a**).

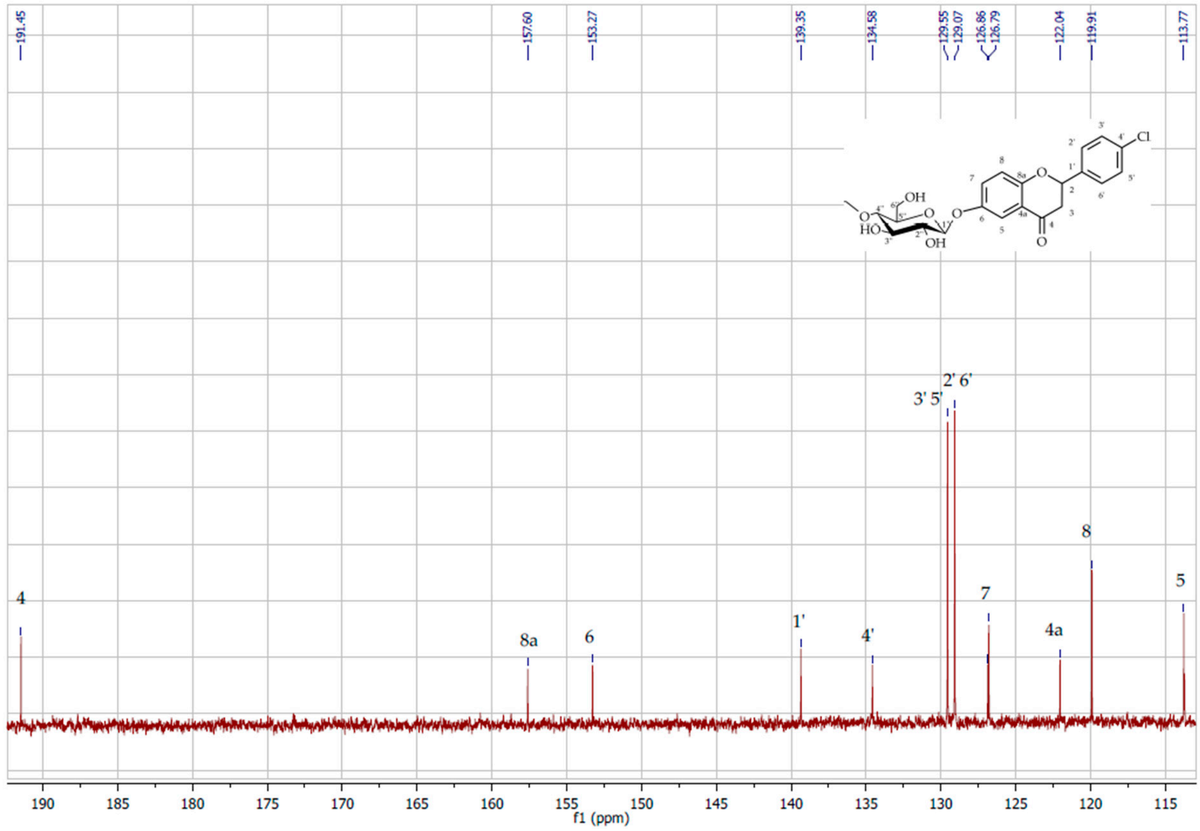

Figure S93. ^{13}C NMR spectrum expansion (*δ,* acetone-d6, 151 MHz) of 4′-chloroflavanone 6-*O*-*β*-D-(4′′-*O*-methyl)-glucopyranoside (**3a**).

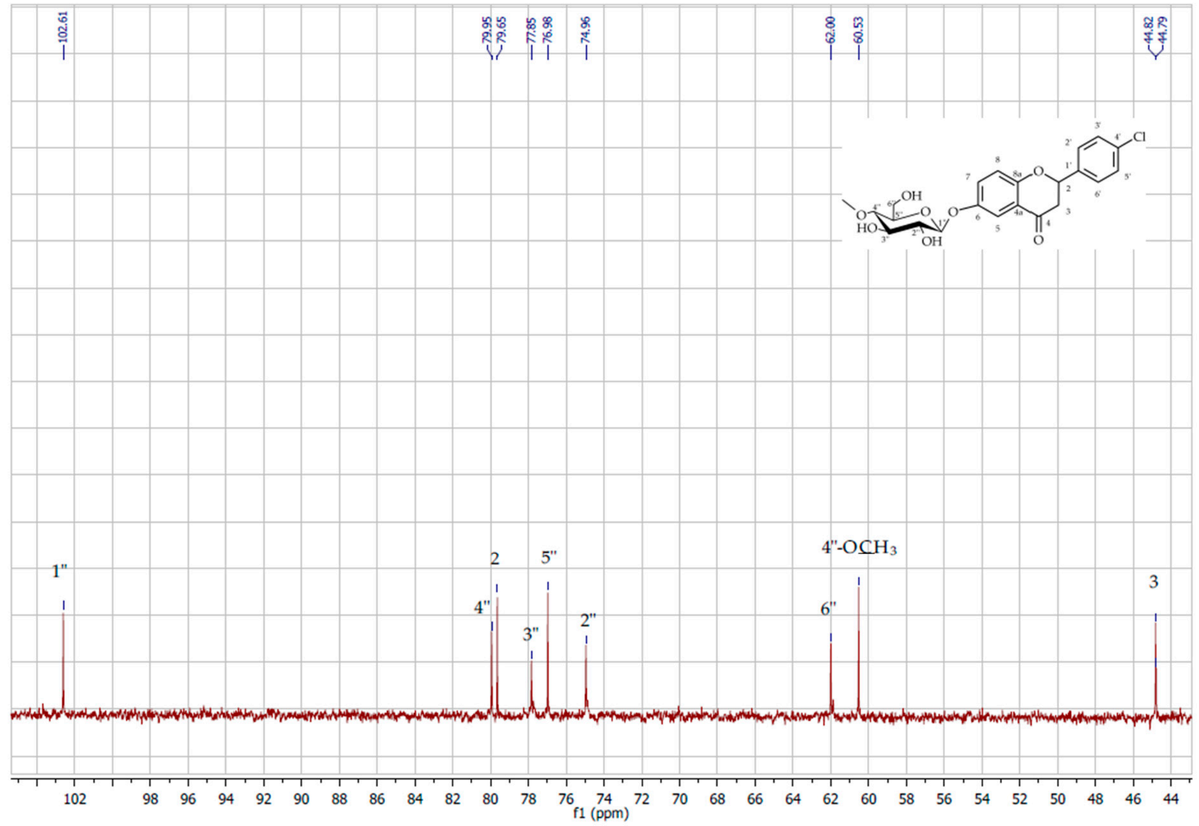

Figure S94. ^{13}C NMR spectrum expansion (*δ*, acetone-d6, 151 MHz) of 4′-chloroflavanone 6-*O*-*β*-D-(4′′-*O*-methyl)-glucopyranoside (**3a**).

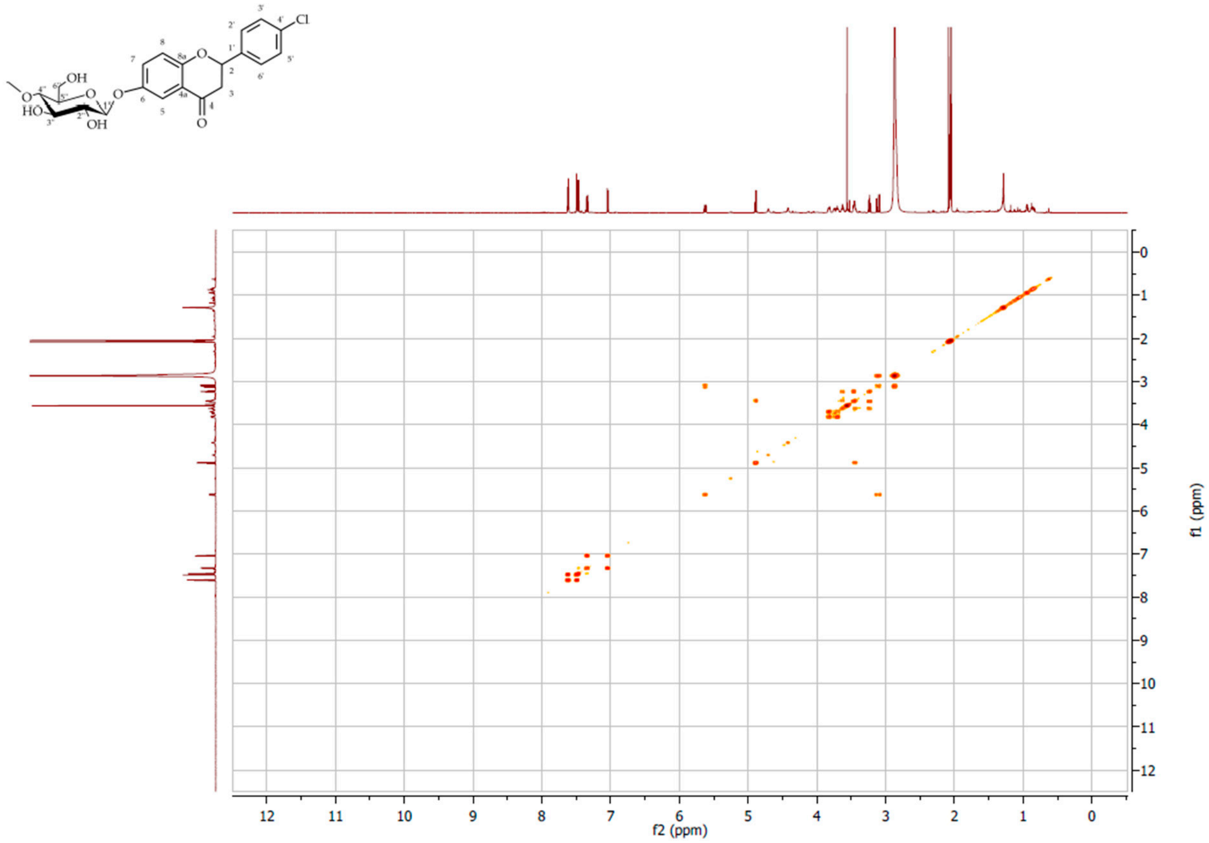

Figure S95. COSY contour map – ^{1}H x ^{1}H of 4′-chloroflavanone 6-*O*-*β*-D-(4′′-*O*-methyl)-glucopyranoside (**3a**).

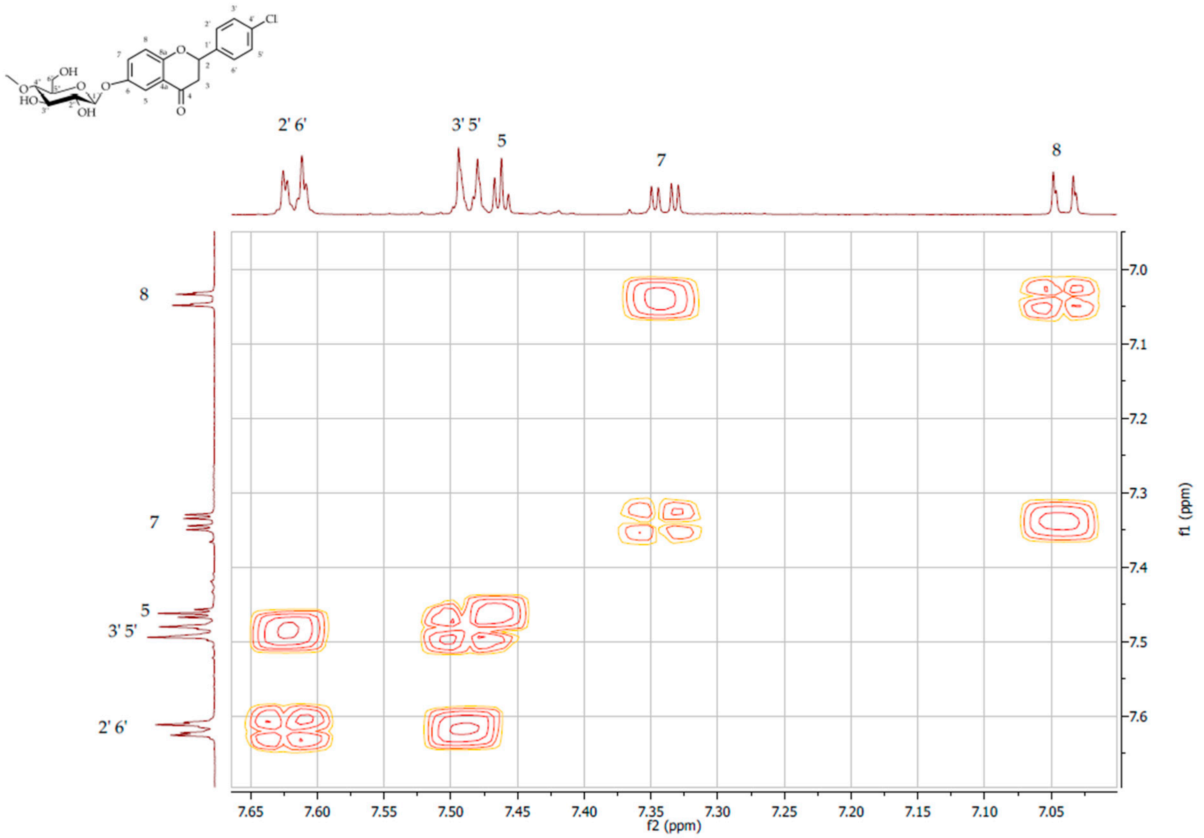

Figure S96. COSY contour map – ¹H x ¹H expansion of 4′-chloroflavanone 6-*O*-*β*-D-(4′′-*O*-methyl)-glucopyranoside (**3a**).

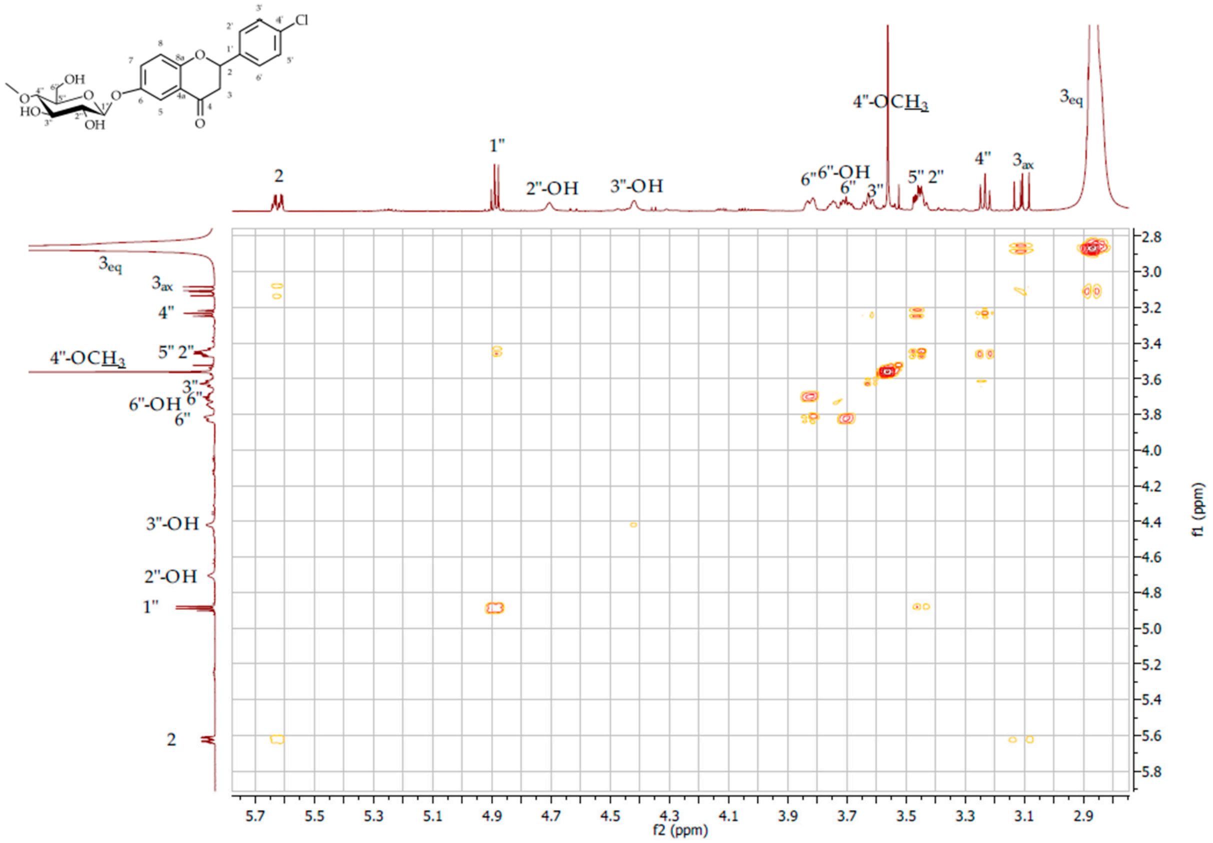

Figure S97. COSY contour map – ¹H x ¹H expansion of 4′-chloroflavanone 6-*O*-*β*-D-(4′′-*O*-methyl)-glucopyranoside (**3a**).

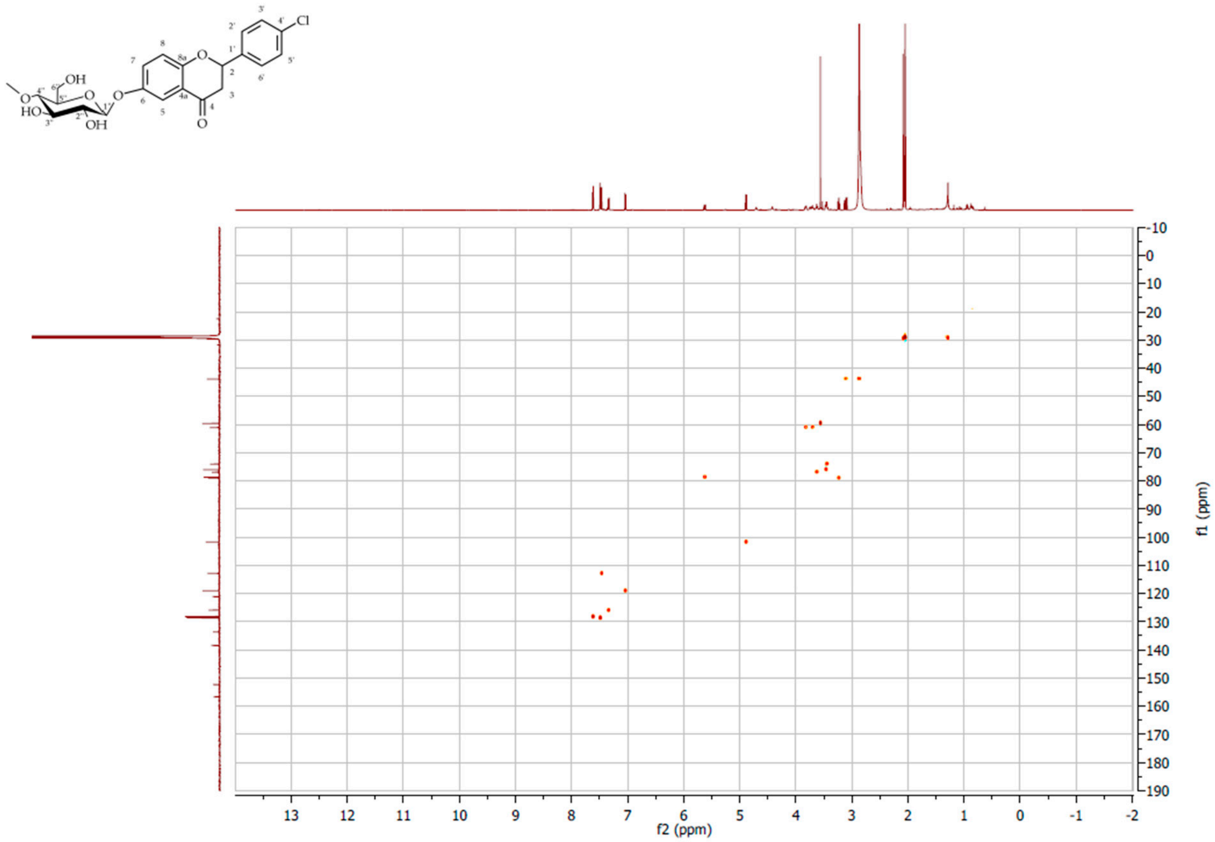

Figure S98. HMQC contour map – ^{1}H x ^{13}C of 4′-chloroflavanone 6-*O*-*β*-D-(4′′-*O*-methyl)-glucopyranoside (**3a**).

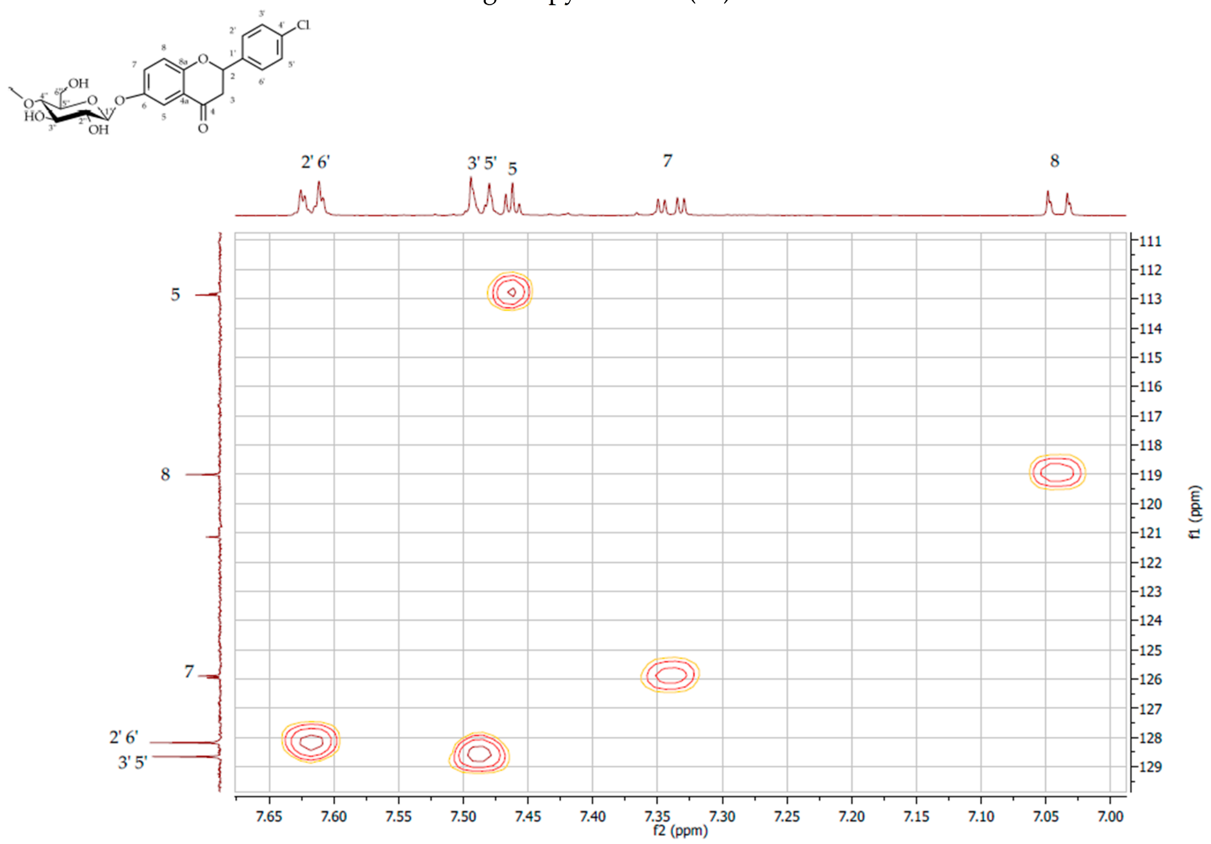

Figure S99. HMQC contour map – ^{1}H x ^{13}C expansion of 4′-chloroflavanone 6-*O*-*β*-D-(4′′-*O*-methyl)-glucopyranoside (**3a**).

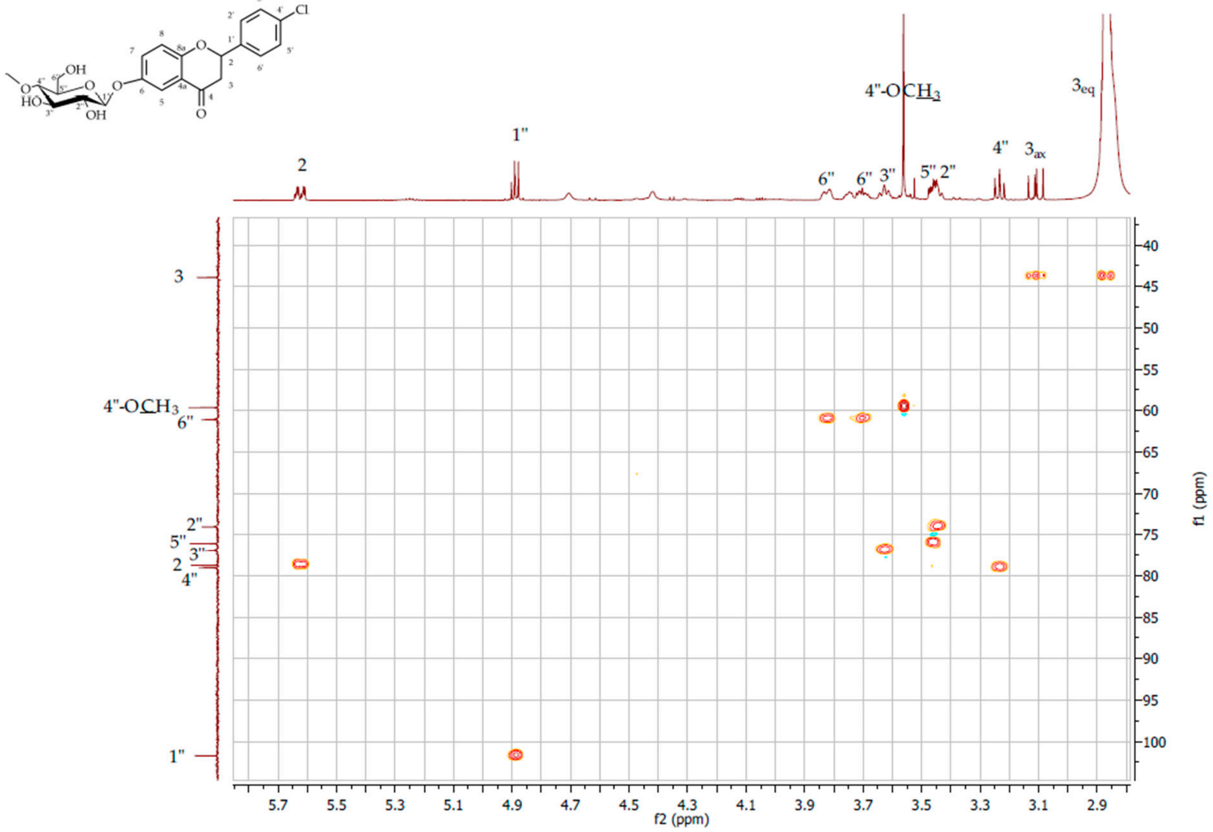

Figure S100. HMQC contour map – $^{1}H \times ^{13}C$ expansion of 4′-chloroflavanone 6-*O*-*β*-D-(4′′-*O*-methyl)-glucopyranoside (**3a**).

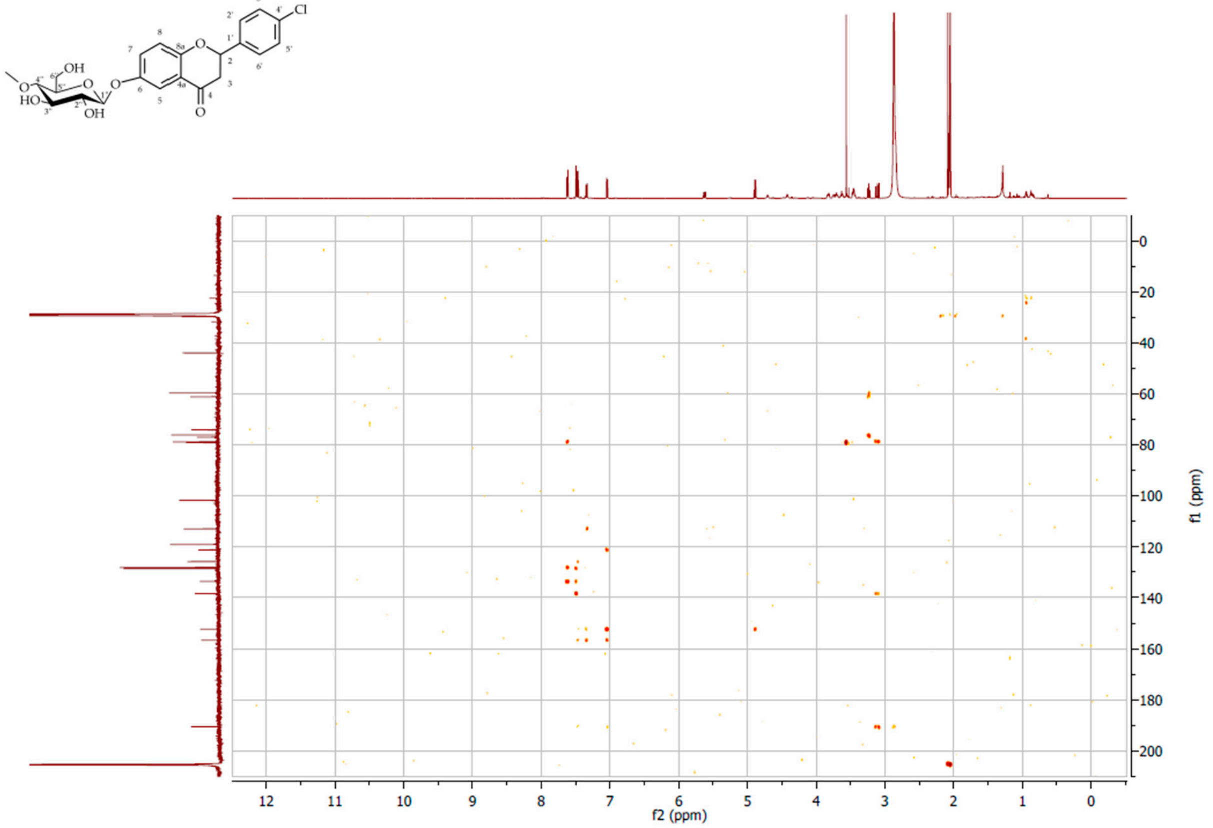

Figure S101. HMBC contour map – $^{1}H \times ^{13}C$ of 4′-chloroflavanone 6-*O*-*β*-D-(4′′-*O*-methyl)-glucopyranoside (**3a**).

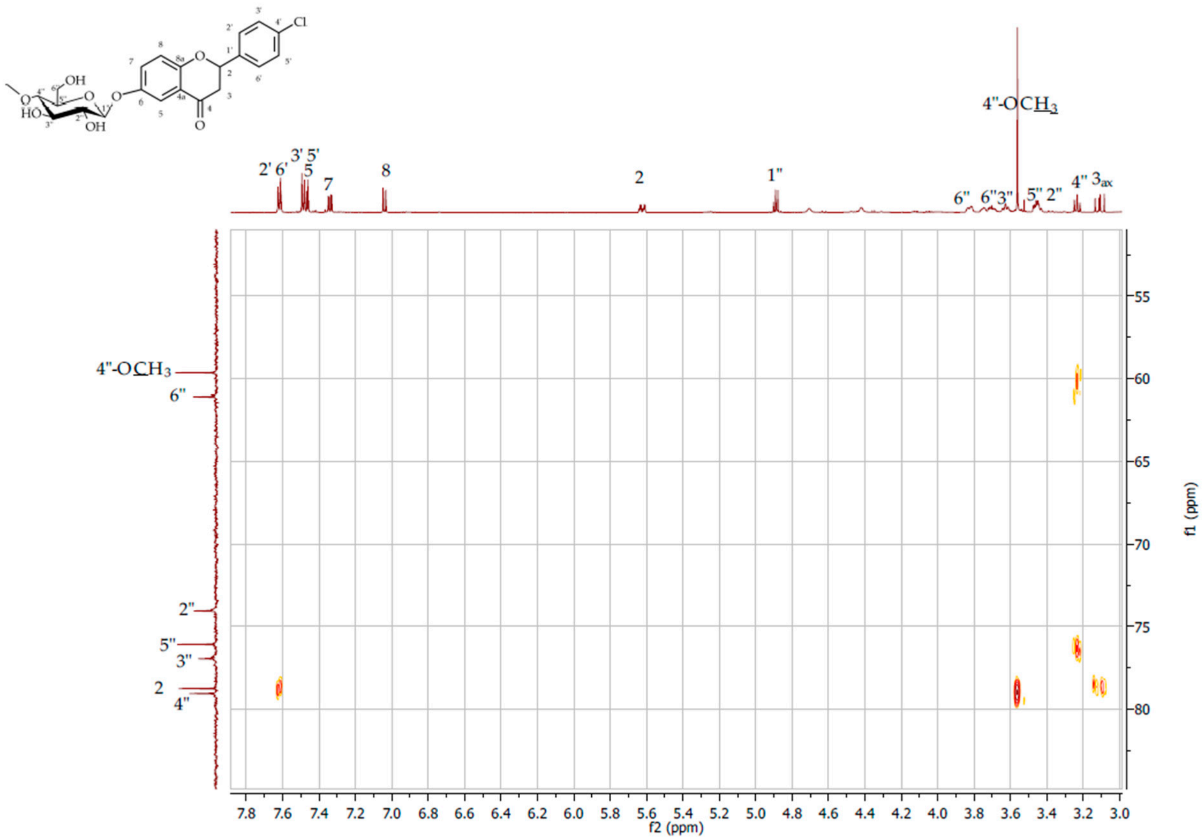

Figure S102. HMBC contour map – ^{1}H x ^{13}C expansion of 4′-chloroflavanone 6-*O*-*β*-D-(4′′-*O*-methyl)-glucopyranoside (**3a**).

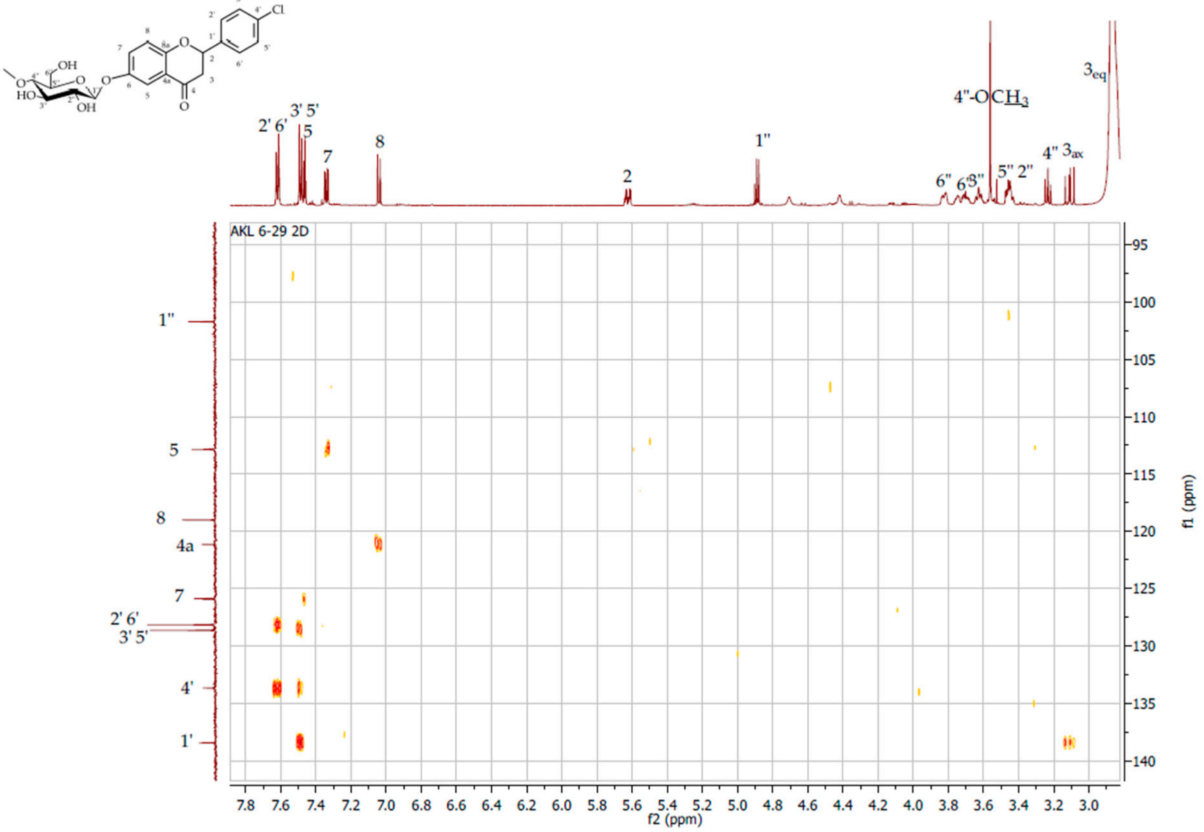

Figure S103. HMBC contour map – ^{1}H x ^{13}C expansion of 4′-chloroflavanone 6-*O*-*β*-D-(4′′-*O*-methyl)-glucopyranoside (**3a**).

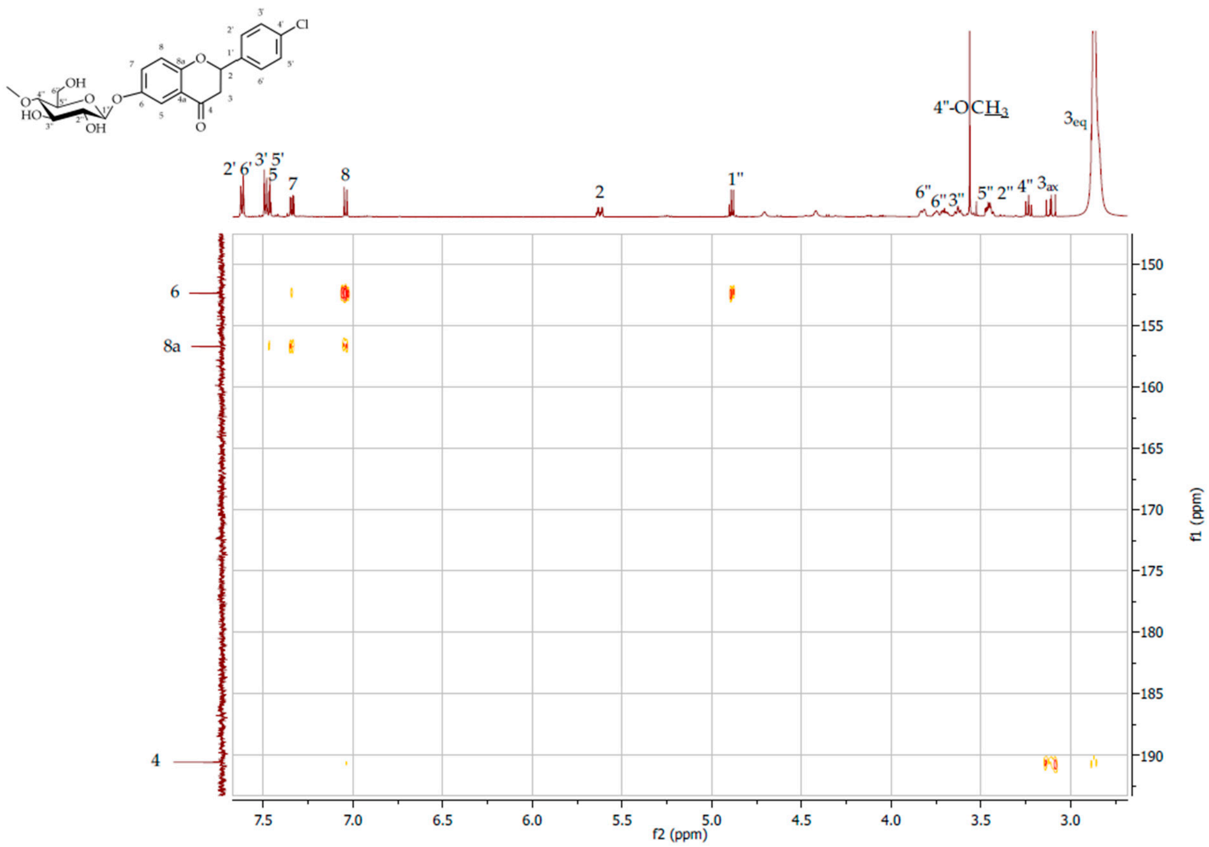

Figure S104. HMBC contour map – ^{1}H x ^{13}C expansion of 4′-chloroflavanone 6-*O*-*β*-D-(4′′-*O*-methyl)-glucopyranoside (**3a**).

Molecule 1

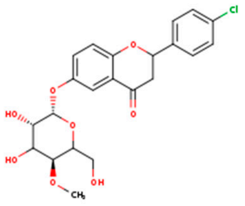

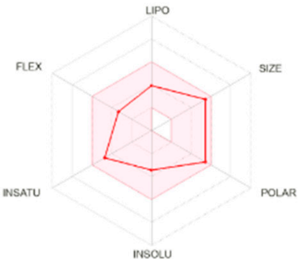

SMILES CO[C@@H]1C(CO)O[C@H]([C@H](C1O)O)Oc1ccc2c(c1)C(=O)CC(O2)c1ccc(cc1)Cl

Physicochemical Properties	
Formula	C22H23ClO8
Molecular weight	450.87 g/mol
Num. heavy atoms	31
Num. arom. heavy atoms	12
Fraction Csp3	0.41
Num. rotatable bonds	5
Num. H-bond acceptors	8
Num. H-bond donors	3
Molar Refractivity	109.39
TPSA	114.68 Å²

Lipophilicity	
Log $P_{o/w}$ (iLOGP)	2.89
Log $P_{o/w}$ (XLOGP3)	1.36
Log $P_{o/w}$ (WLOGP)	1.55
Log $P_{o/w}$ (MLOGP)	0.29
Log $P_{o/w}$ (SILICOS-IT)	2.02
Consensus Log $P_{o/w}$	1.62

Water Solubility	
Log *S* (ESOL)	-3.45
Solubility	1.60e-01 mg/ml ; 3.56e-04 mol/l
Class	Soluble
Log *S* (Ali)	-3.37
Solubility	1.92e-01 mg/ml ; 4.26e-04 mol/l
Class	Soluble
Log *S* (SILICOS-IT)	-4.16
Solubility	3.13e-02 mg/ml ; 6.95e-05 mol/l
Class	Moderately soluble

Pharmacokinetics	
GI absorption	High
BBB permeant	No
P-gp substrate	Yes
CYP1A2 inhibitor	No
CYP2C19 inhibitor	No
CYP2C9 inhibitor	No
CYP2D6 inhibitor	No
CYP3A4 inhibitor	Yes
Log K_p (skin permeation)	-8.08 cm/s

Druglikeness	
Lipinski	Yes; 0 violation
Ghose	Yes
Veber	Yes
Egan	Yes
Muegge	Yes
Bioavailability Score	0.55

Medicinal Chemistry	
PAINS	0 alert
Brenk	0 alert
Leadlikeness	No; 1 violation: MW>350
Synthetic accessibility	4.99

Figure S105. 4′-Chloroflavanone 6-*O*-*β*-D-(4′′-*O*-methyl)-glucopyranoside (**3a**) physicochemical and ADME parameters prediction using the SwissADME modelling.

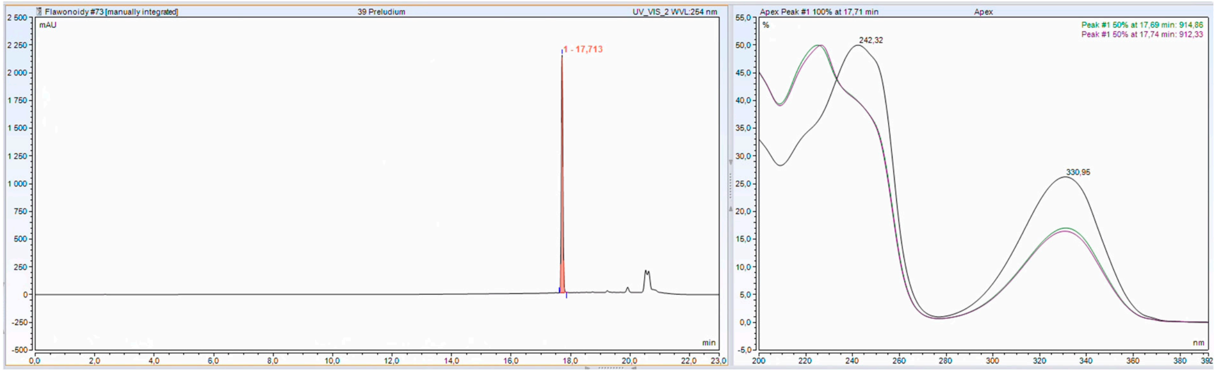

Figure S106. HPLC analysis of 6-chloroflavanone (**4**).

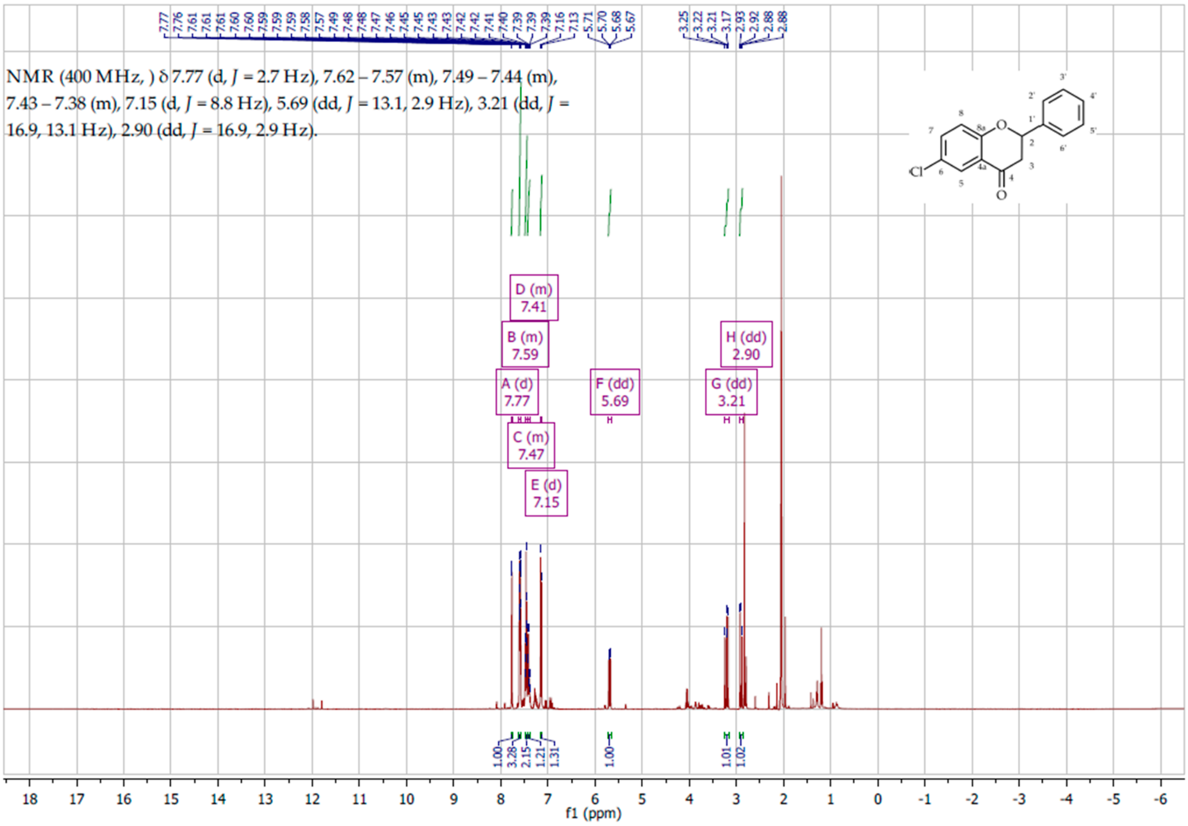

Figure S107. ^{1}H NMR spectrum (*δ*, acetone-d6, 600 MHz) of 6-chloroflavanone (**4**).

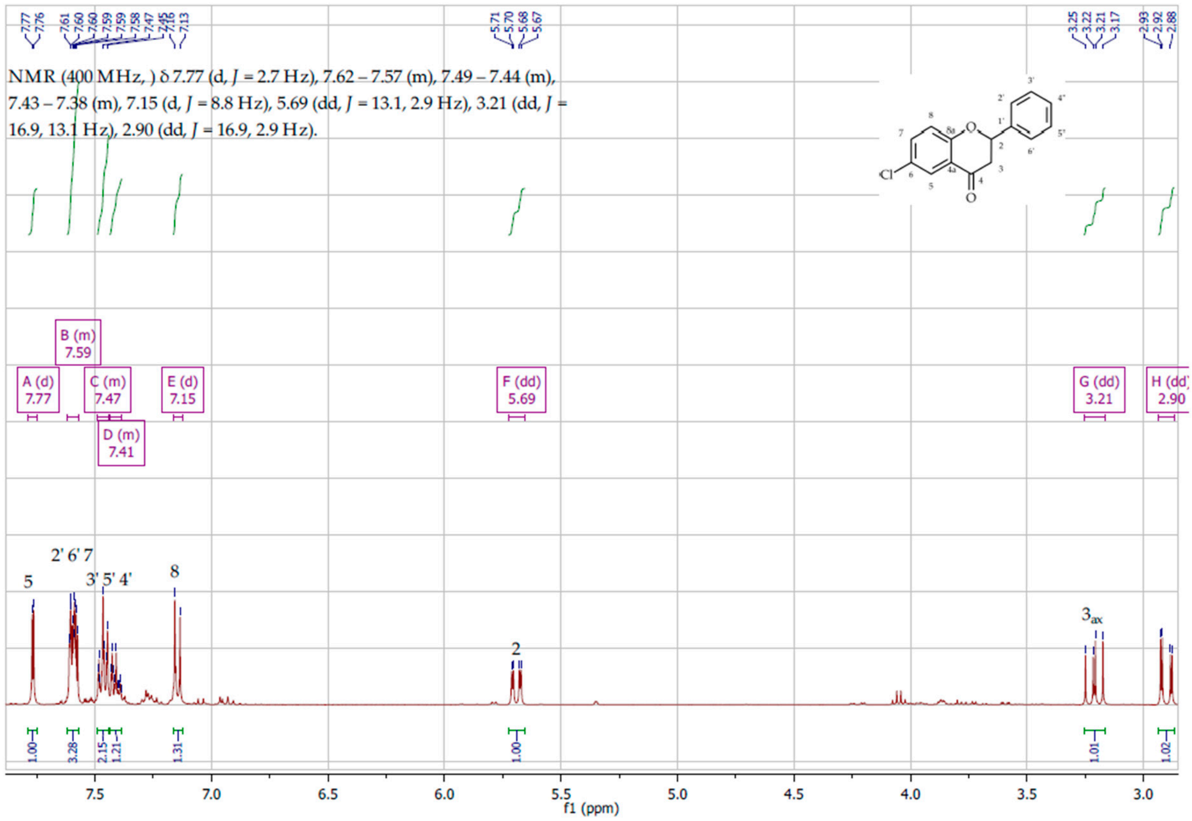

Figure S108. ^{1}H NMR spectrum expansion (δ, acetone-d6, 600 MHz) of 6-chloroflavanone (**4**).

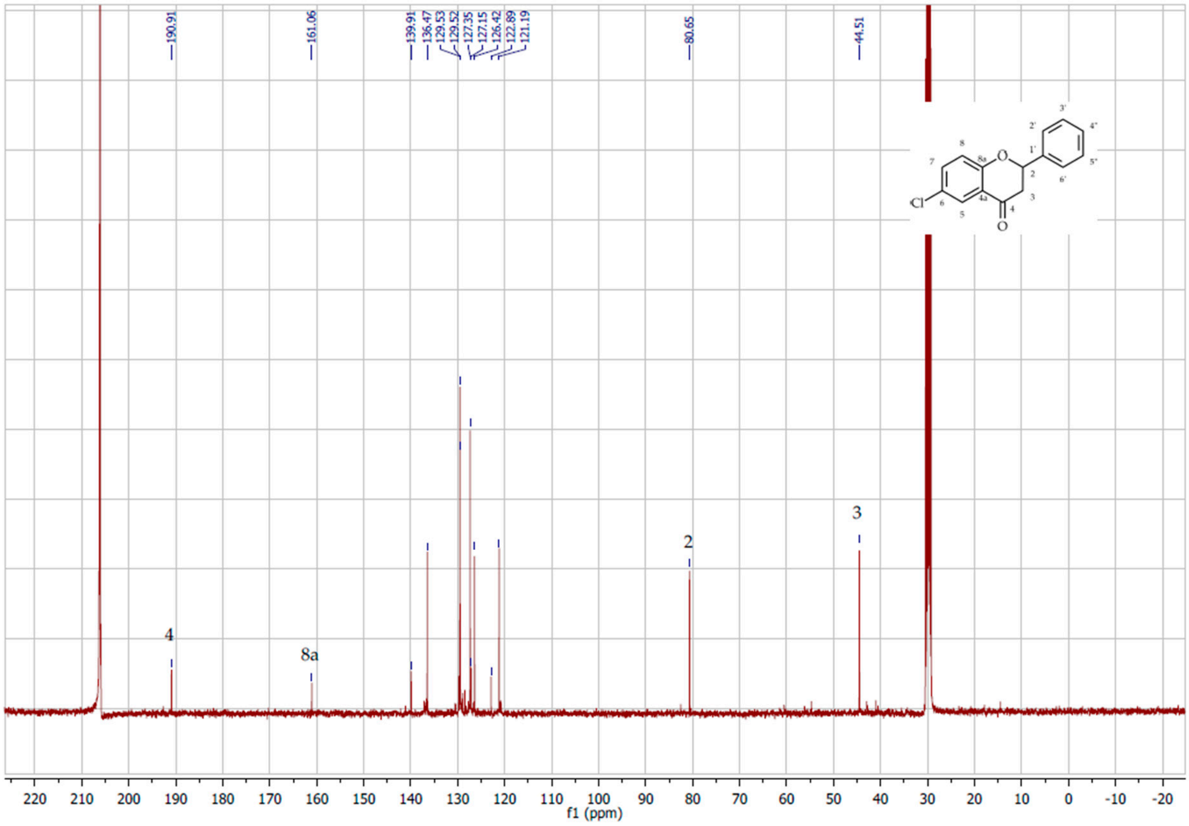

Figure S109. ^{13}C NMR spectrum (δ, acetone-d6, 151 MHz) of 6-chloroflavanone (**4**).

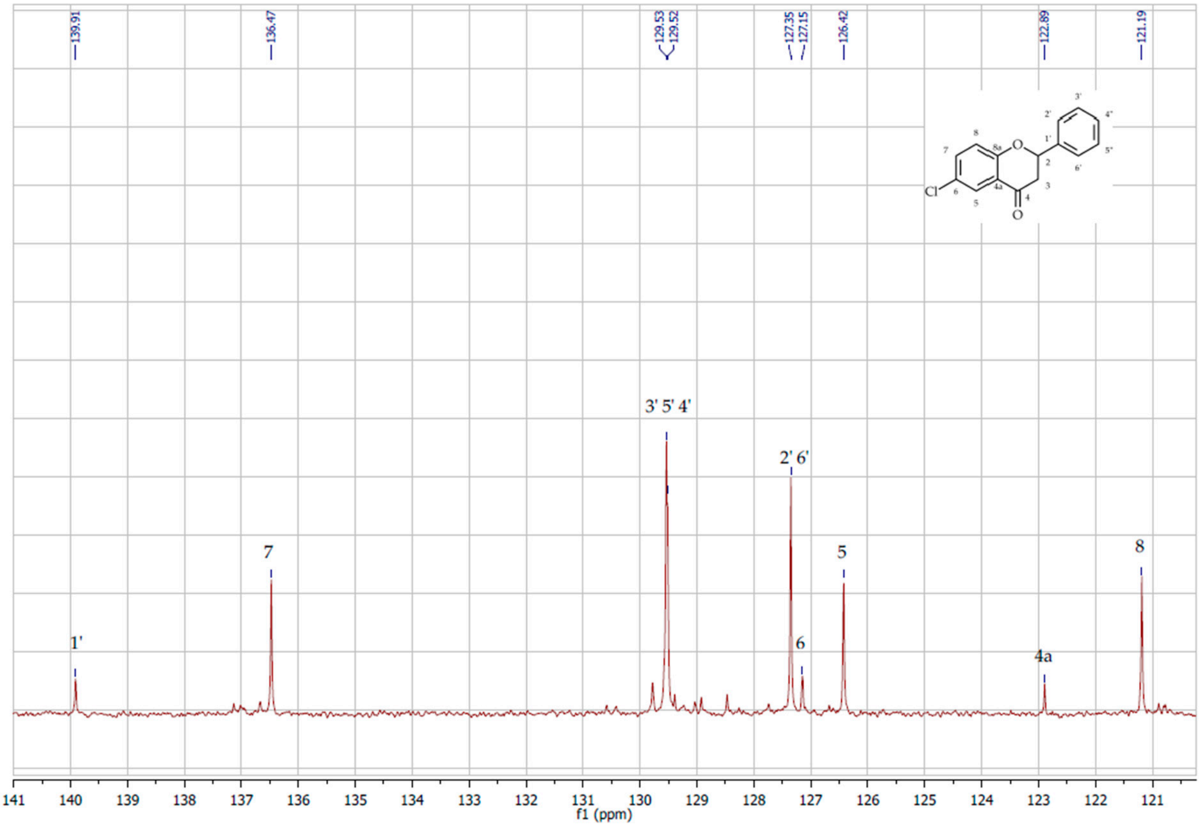

Figure S110. ^{13}C NMR spectrum expansion (δ, acetone-d6, 151 MHz) of 6-chloroflavanone (**4**).

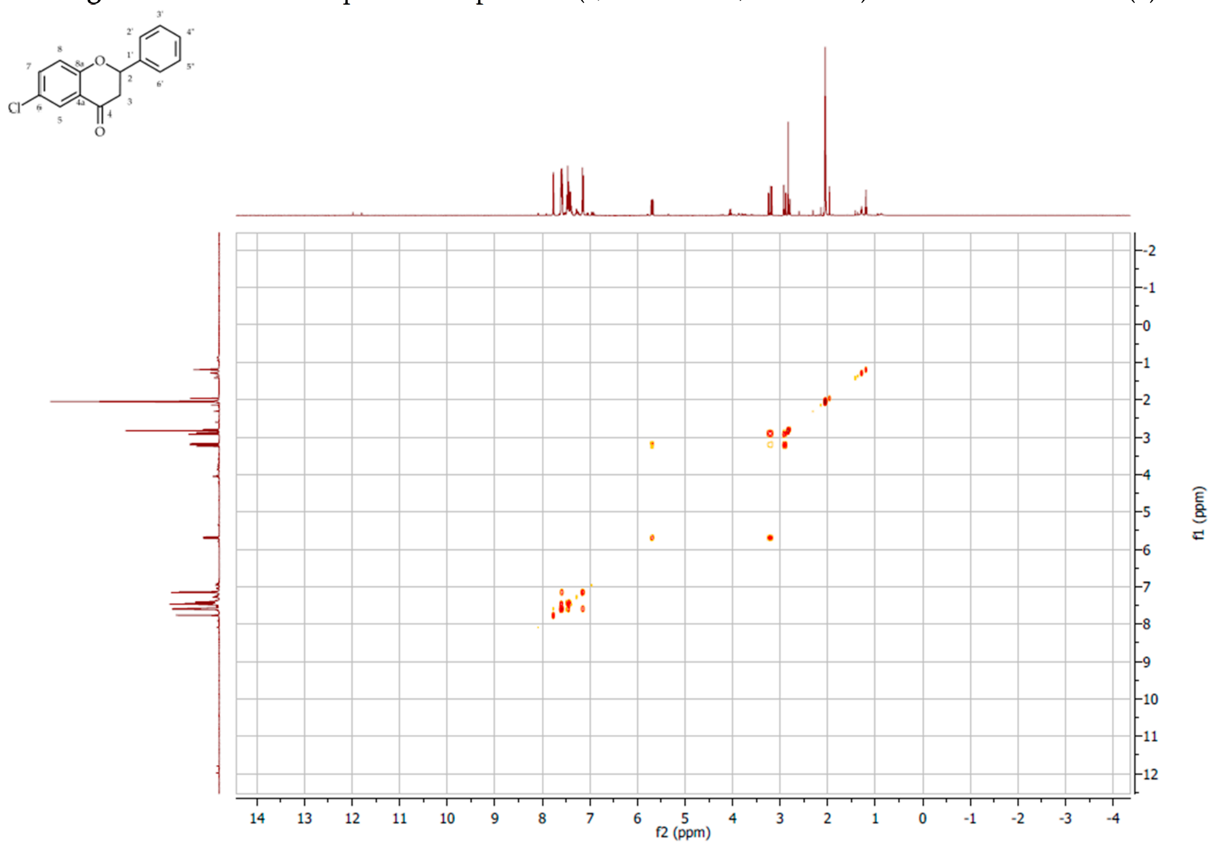

Figure S111. COSY contour map – ^{1}H x ^{1}H of 6-chloroflavanone (**4**).

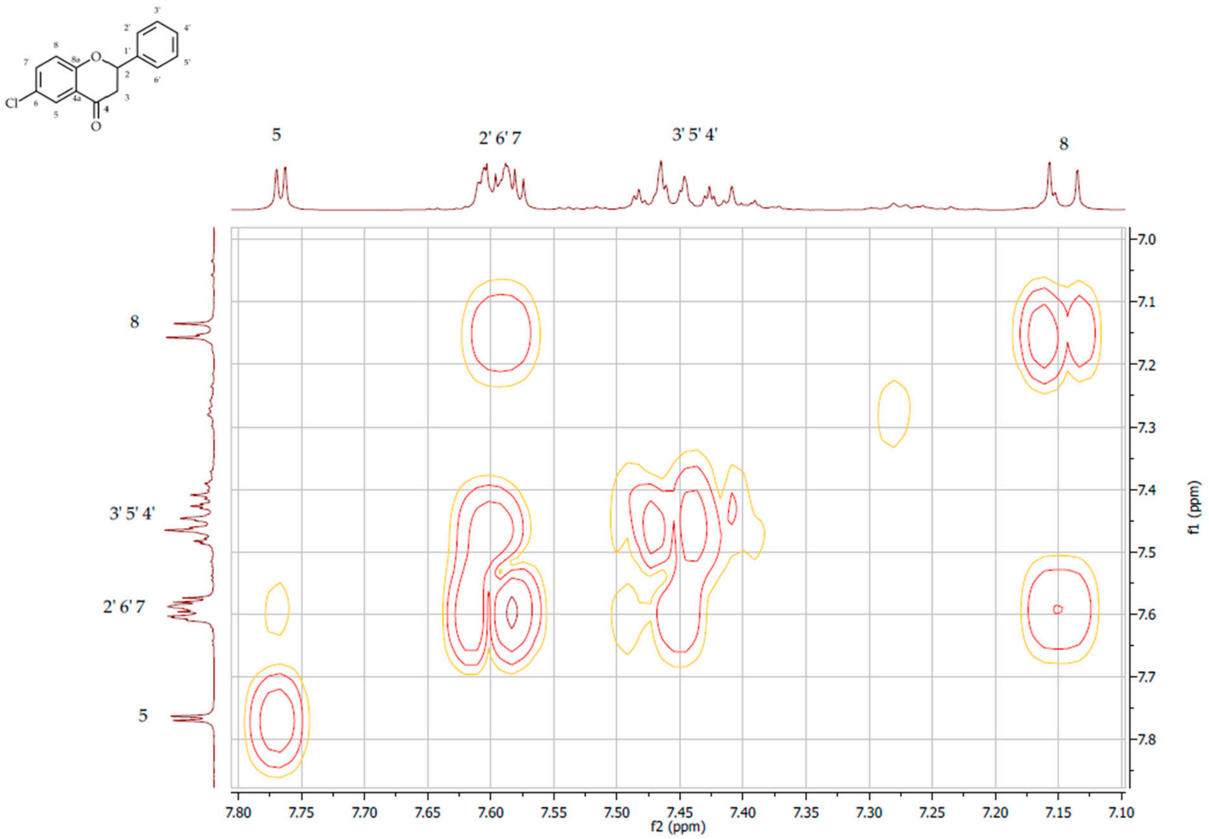

Figure S112. COSY contour map – ^1H x ^1H expansion of 6-chloroflavanone (**4**).

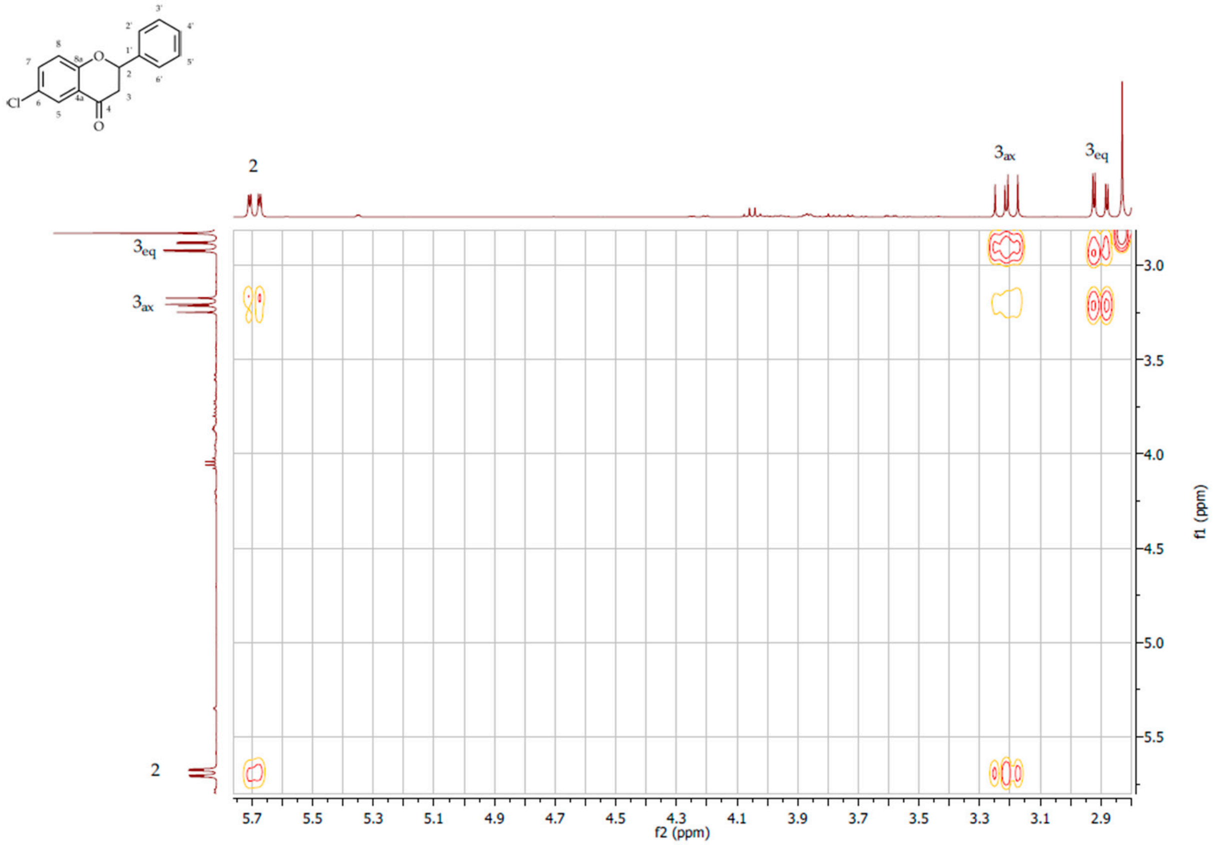

Figure S113. COSY contour map – ^1H x ^1H expansion of 6-chloroflavanone (**4**).

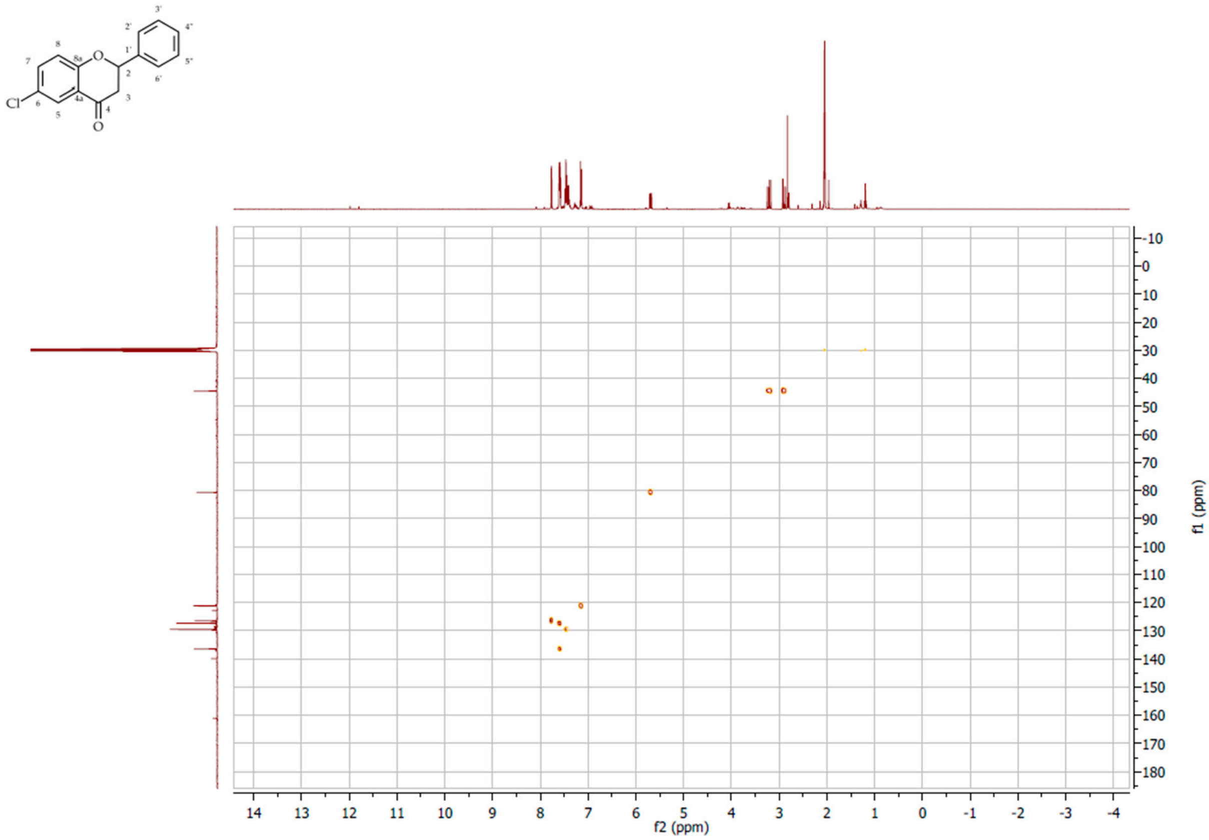

Figure S114. HMQC contour map – ^{1}H x ^{13}C of 6-chloroflavanone (**4**).

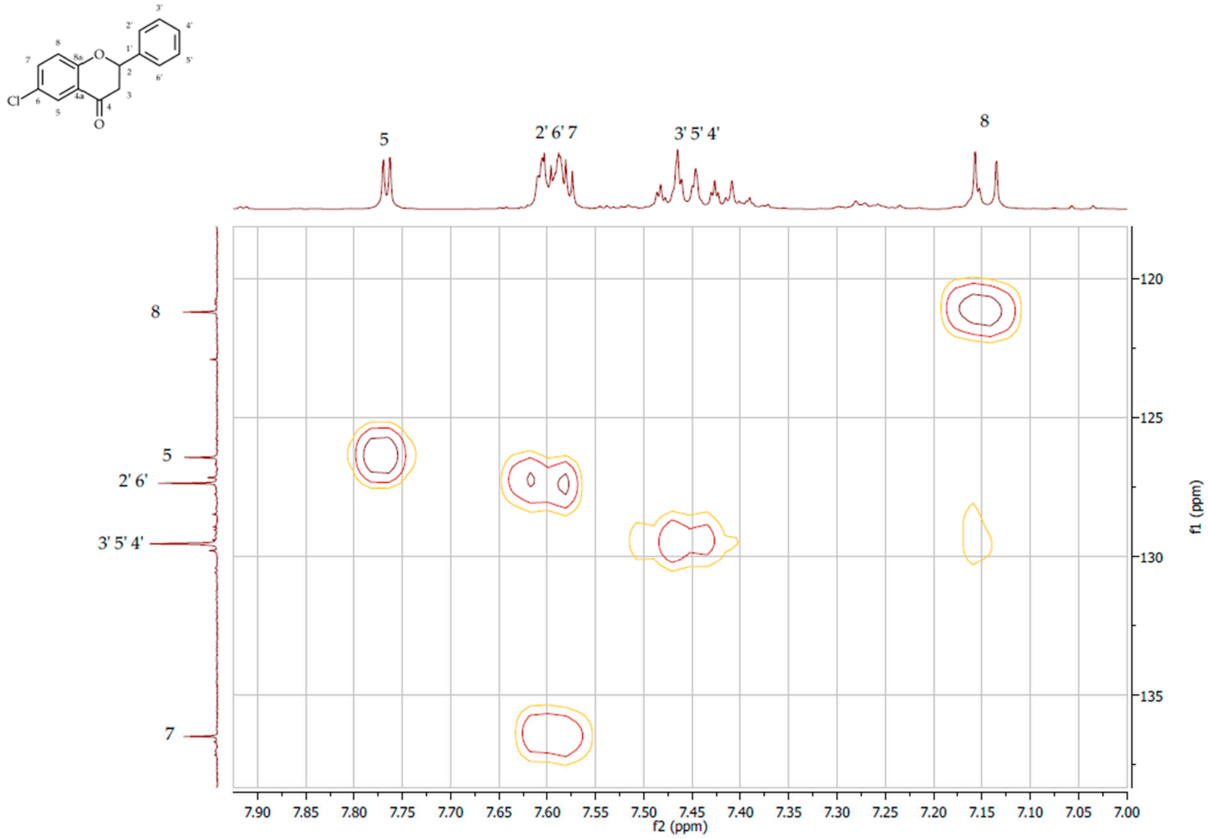

Figure S115. HMQC contour map – ^{1}H x ^{13}C expansion of 6-chloroflavanone (**4**).

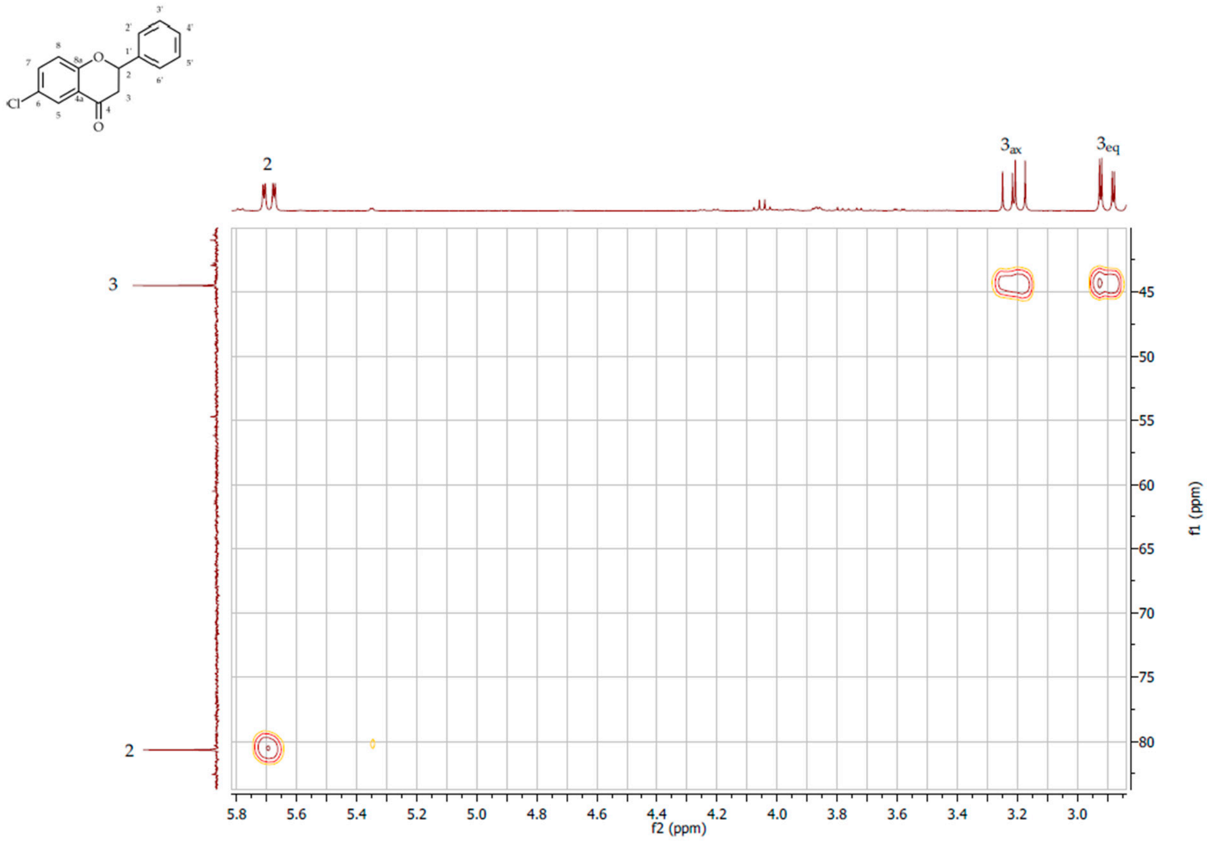

Figure S116. HMQC contour map – ^{1}H x ^{13}C expansion of 6-chloroflavanone (**4**).

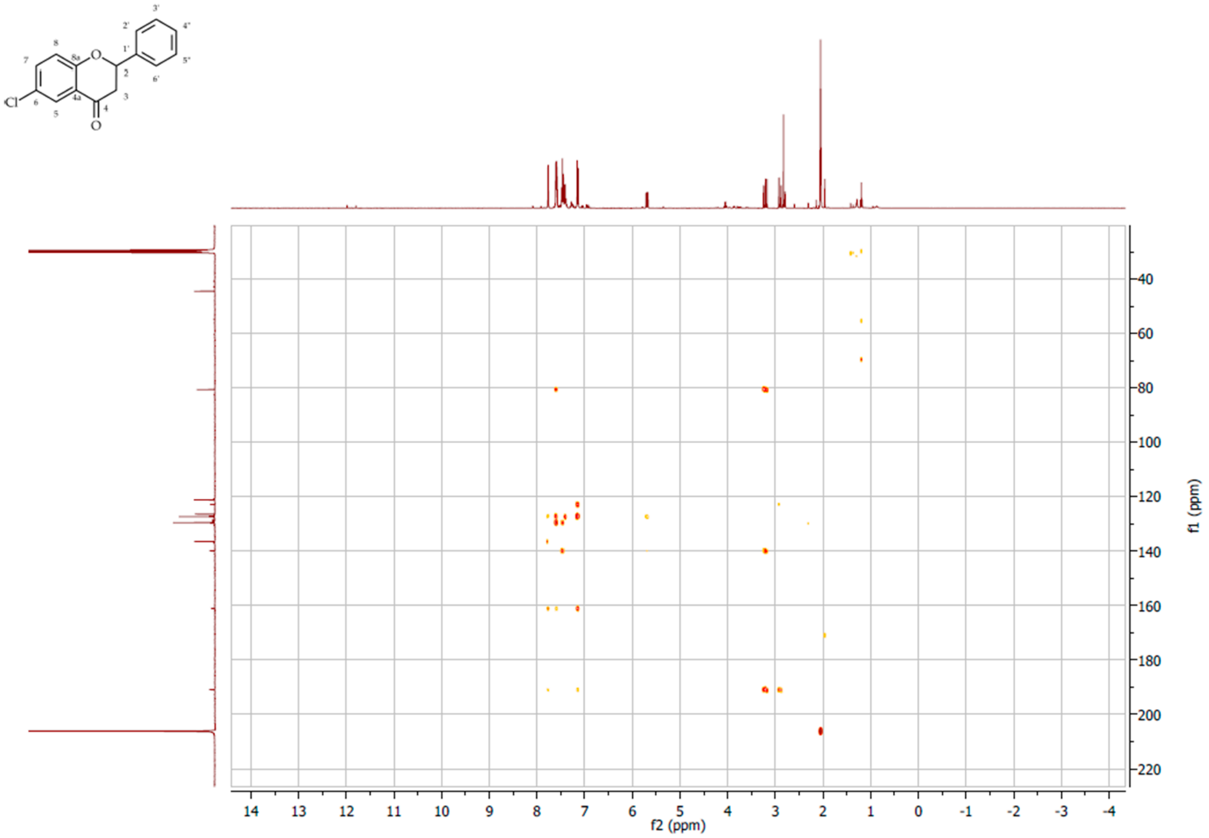

Figure S117. HMBC contour map – ^{1}H x ^{13}C of 6-chloroflavanone (**4**).

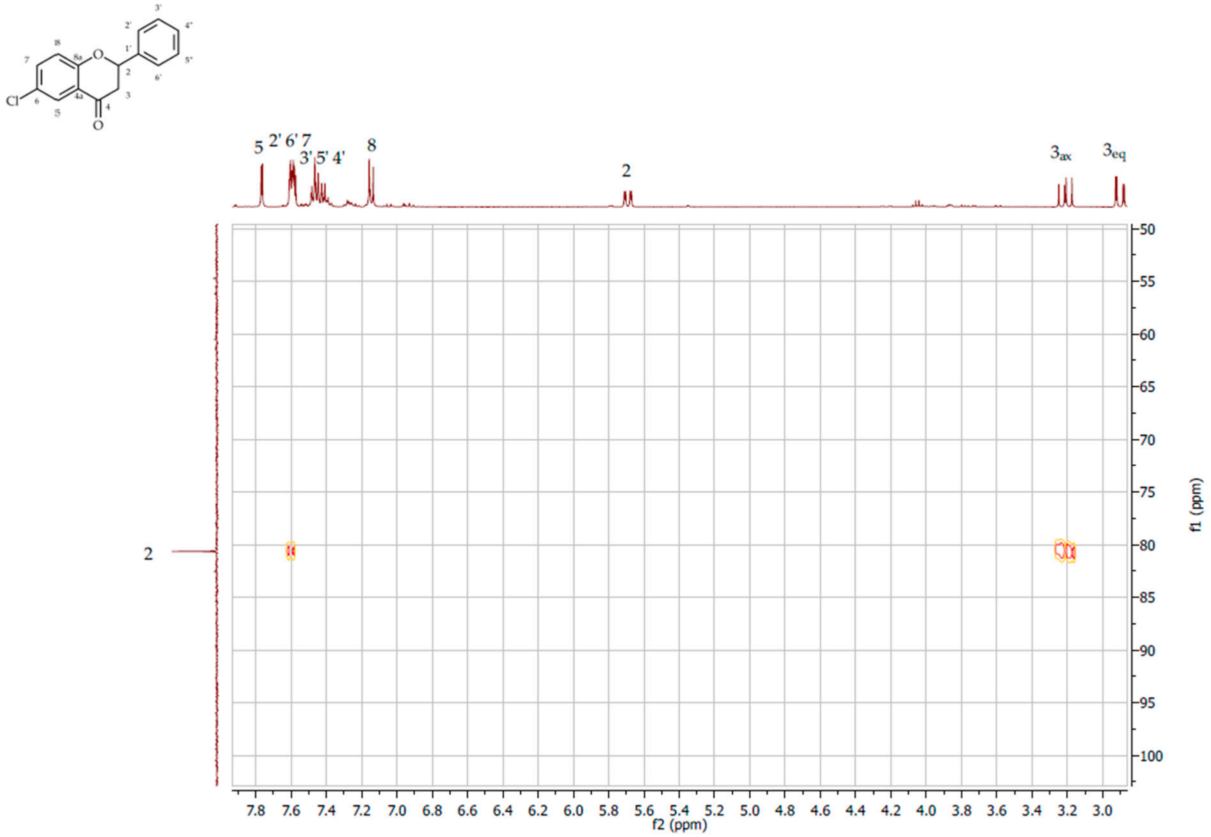

Figure S118. HMBC contour map – ^{1}H x ^{13}C expansion of 6-chloroflavanone (**4**).

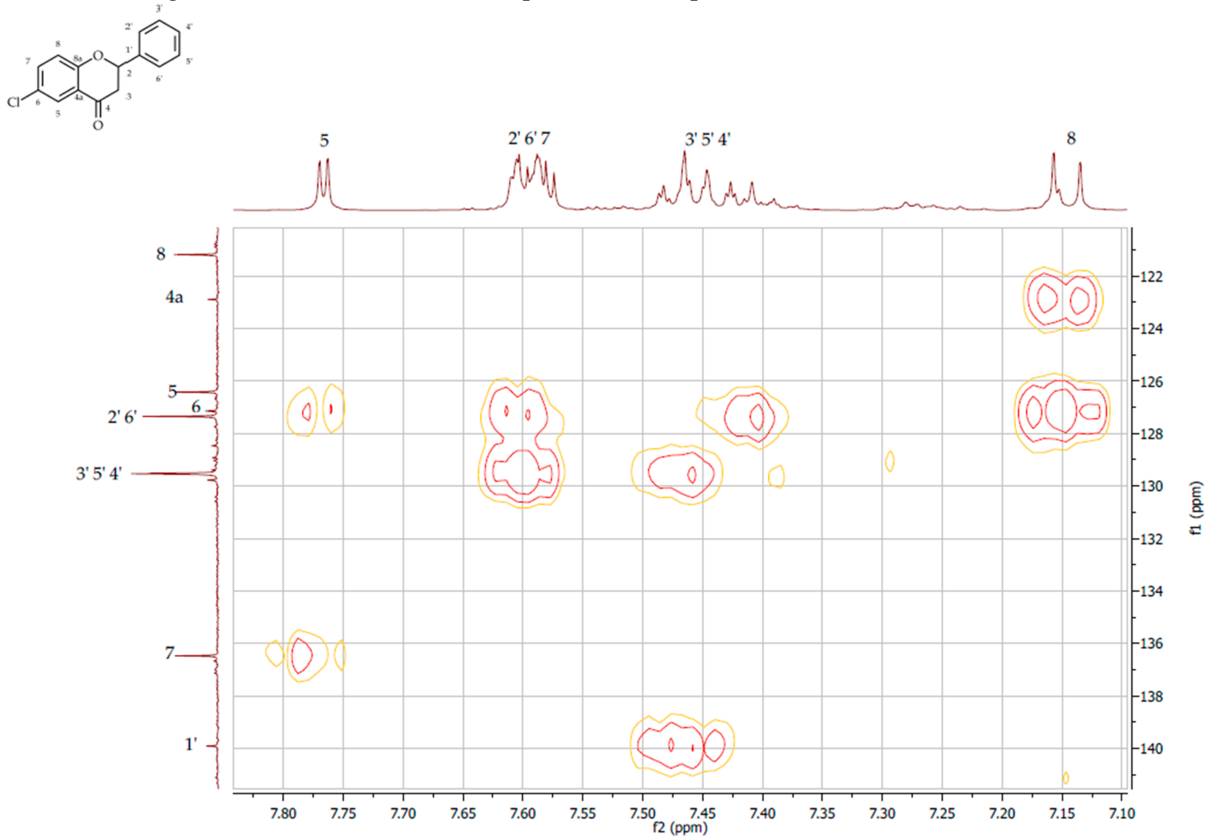

Figure S119. HMBC contour map – ^{1}H x ^{13}C expansion of 6-chloroflavanone (**4**).

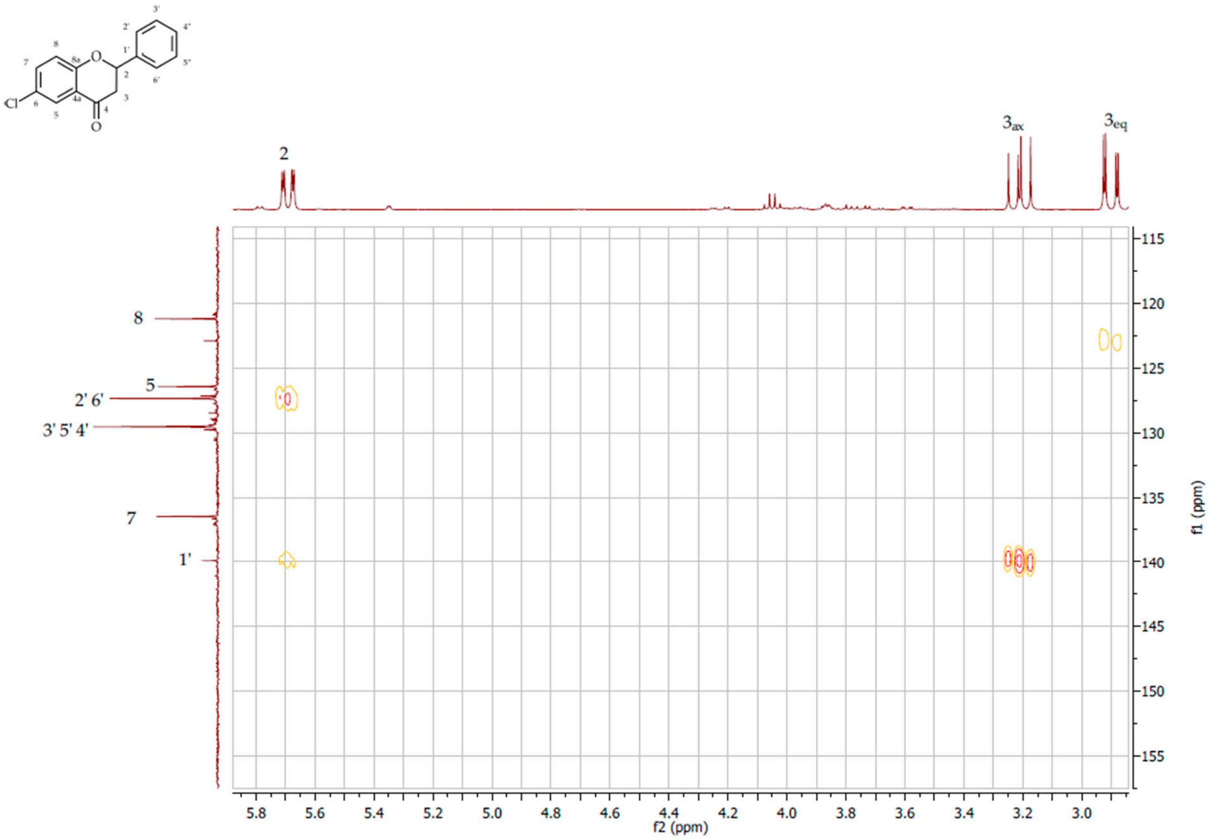

Figure S120. HMBC contour map – ^{1}H x ^{13}C expansion of 6-chloroflavanone (**4**).

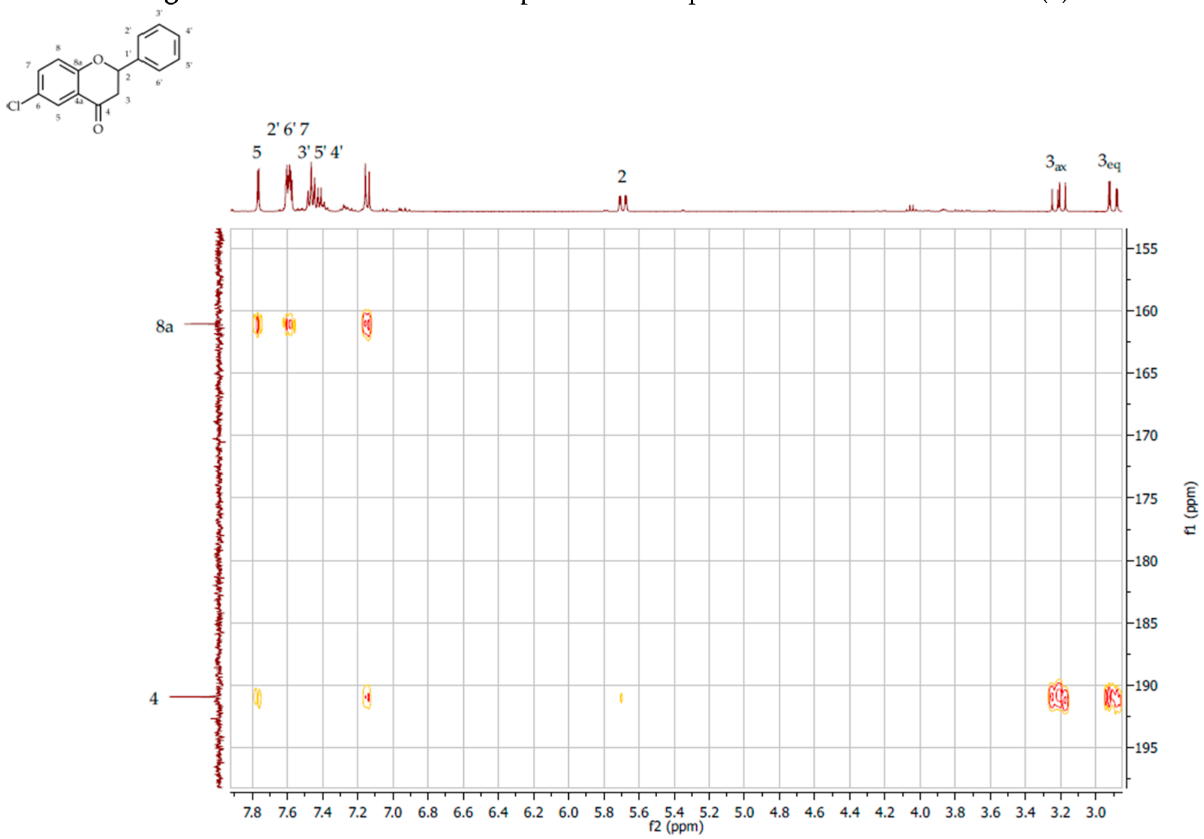

Figure S121. HMBC contour map – ^{1}H x ^{13}C expansion of 6-chloroflavanone (**4**).

Molecule 1

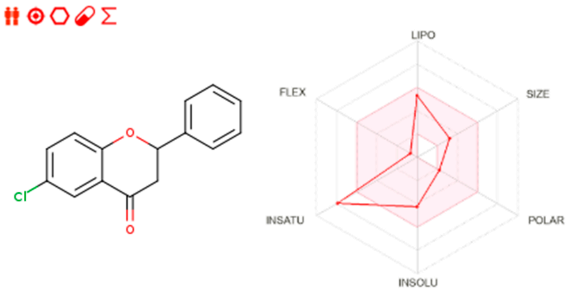

SMILES Clc1ccc2c(c1)C(=O)CC(O2)c1ccccc1

Physicochemical Properties	
Formula	C15H11ClO2
Molecular weight	258.70 g/mol
Num. heavy atoms	18
Num. arom. heavy atoms	12
Fraction Csp3	0.13
Num. rotatable bonds	1
Num. H-bond acceptors	2
Num. H-bond donors	0
Molar Refractivity	70.51
TPSA	26.30 Å²

Lipophilicity	
Log $P_{o/w}$ (iLOGP)	2.63
Log $P_{o/w}$ (XLOGP3)	3.77
Log $P_{o/w}$ (WLOGP)	3.72
Log $P_{o/w}$ (MLOGP)	2.98
Log $P_{o/w}$ (SILICOS-IT)	4.18
Consensus Log $P_{o/w}$	3.46

Water Solubility	
Log *S* (ESOL)	-4.25
Solubility	1.47e-02 mg/ml ; 5.67e-05 mol/l
Class	Moderately soluble
Log *S* (Ali)	-4.02
Solubility	2.50e-02 mg/ml ; 9.65e-05 mol/l
Class	Moderately soluble
Log *S* (SILICOS-IT)	-5.76
Solubility	4.48e-04 mg/ml ; 1.73e-06 mol/l
Class	Moderately soluble

Pharmacokinetics	
GI absorption	High
BBB permeant	Yes
P-gp substrate	No
CYP1A2 inhibitor	Yes
CYP2C19 inhibitor	Yes
CYP2C9 inhibitor	No
CYP2D6 inhibitor	No
CYP3A4 inhibitor	No
Log K_p (skin permeation)	-5.20 cm/s

Druglikeness	
Lipinski	Yes; 0 violation
Ghose	Yes
Veber	Yes
Egan	Yes
Muegge	Yes
Bioavailability Score	0.55

Medicinal Chemistry	
PAINS	0 alert
Brenk	0 alert
Leadlikeness	No; 1 violation: XLOGP3>3.5
Synthetic accessibility	2.83

Figure S122. 6-Chloroflavanone (**4**) physicochemical and ADME parameters prediction using the SwissADME modelling.

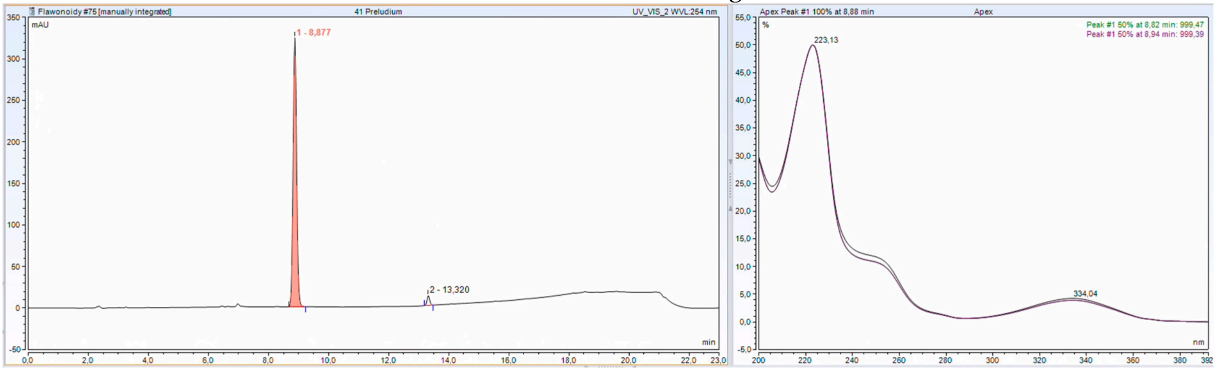

Figure S123. HPLC analysis of 6-chloroflavanone 4′-*O*-*β*-D-(4′′-*O*-methyl)-glucopyranoside (**4a**).

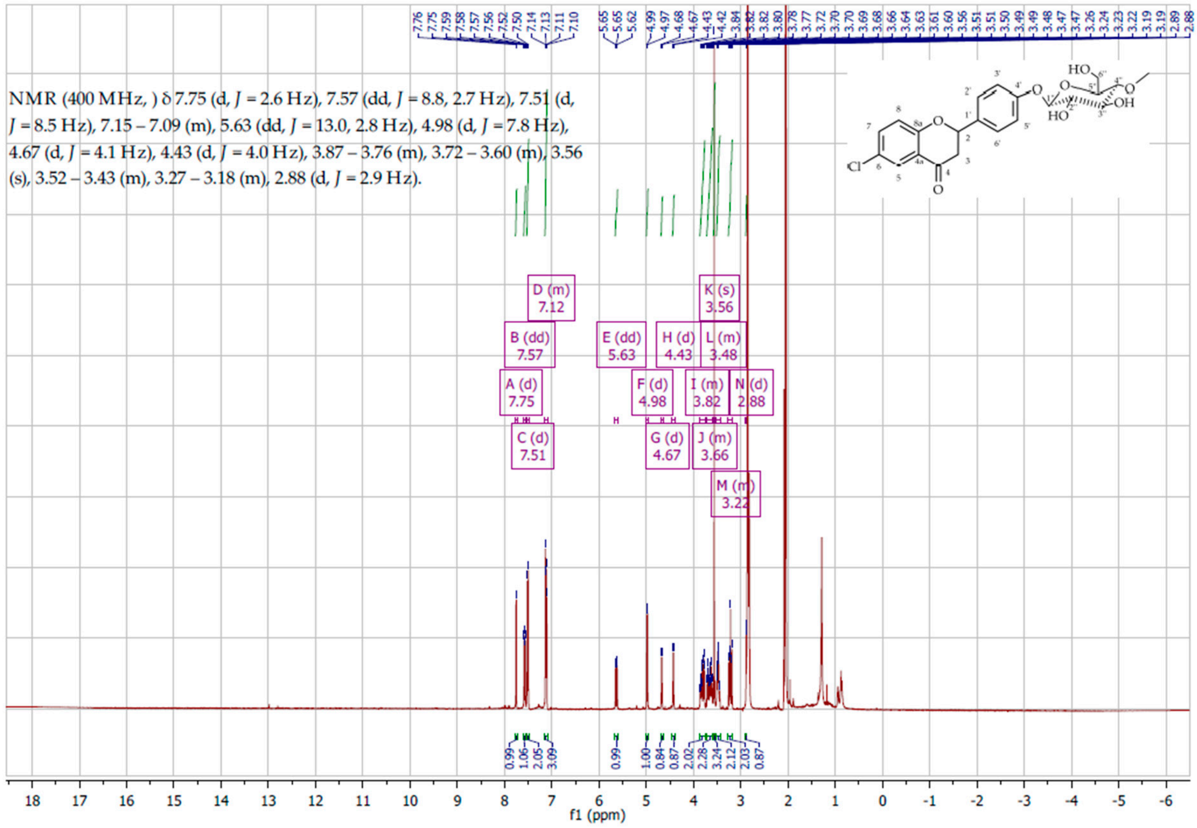

Figure S124. ^{1}H NMR spectrum (*δ*, acetone-d6, 600 MHz) of 6-chloroflavanone 4′-*O*-*β*-D-(4′′-*O*-methyl)-glucopyranoside (**4a**).

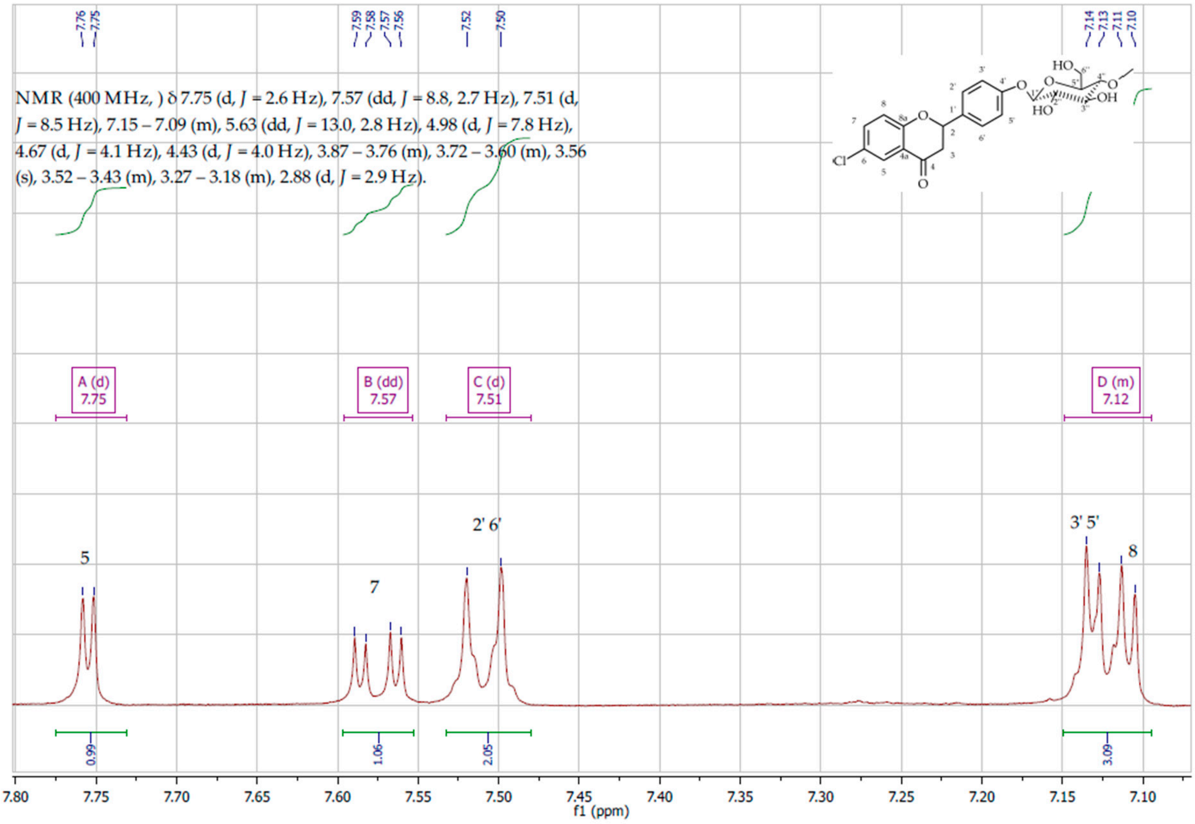

Figure S125. ^{1}H NMR spectrum expansion (*δ*, acetone-d6, 600 MHz) of 6-chloroflavanone 4′-*O*-*β*-D-(4′′-*O*-methyl)-glucopyranoside (**4a**).

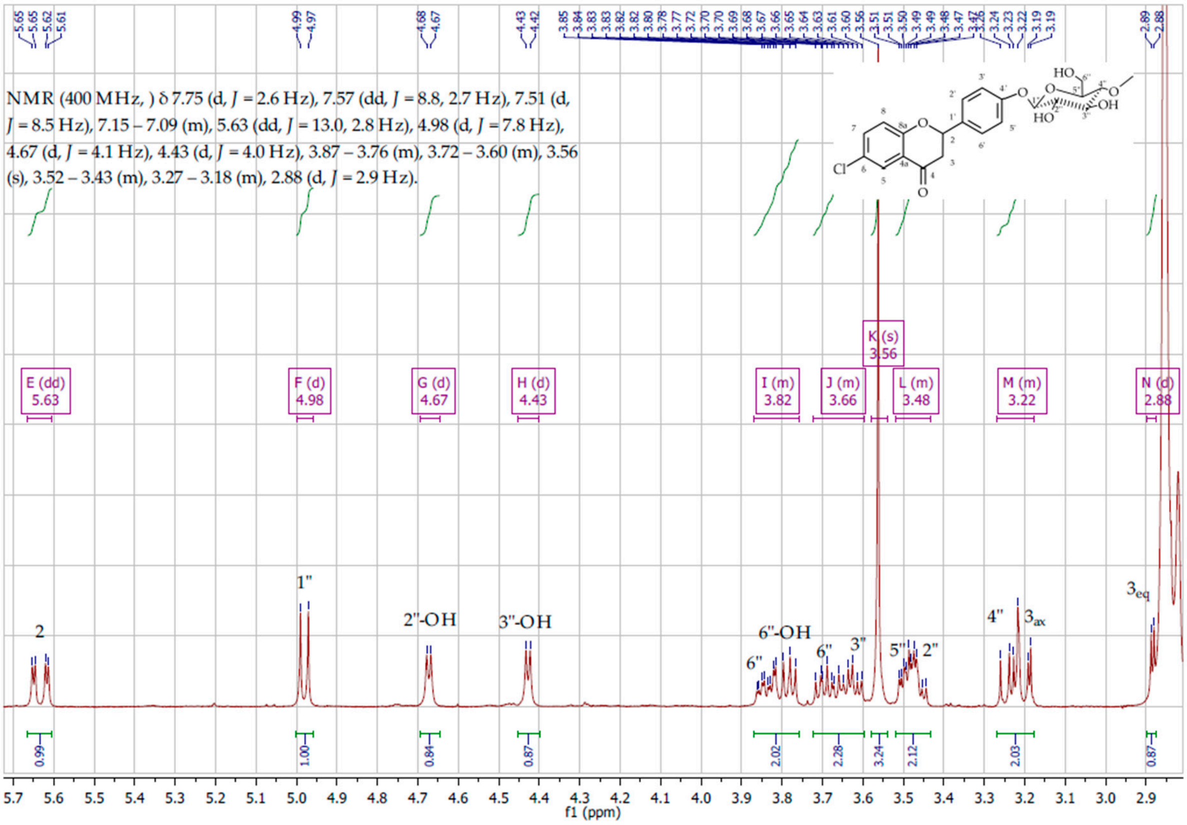

Figure S126. ^{1}H NMR spectrum expansion (*δ,* acetone-d6, 600 MHz) of 6-chloroflavanone 4′-*O*-*β*-D-(4′′-*O*-methyl)-glucopyranoside (**4a**).

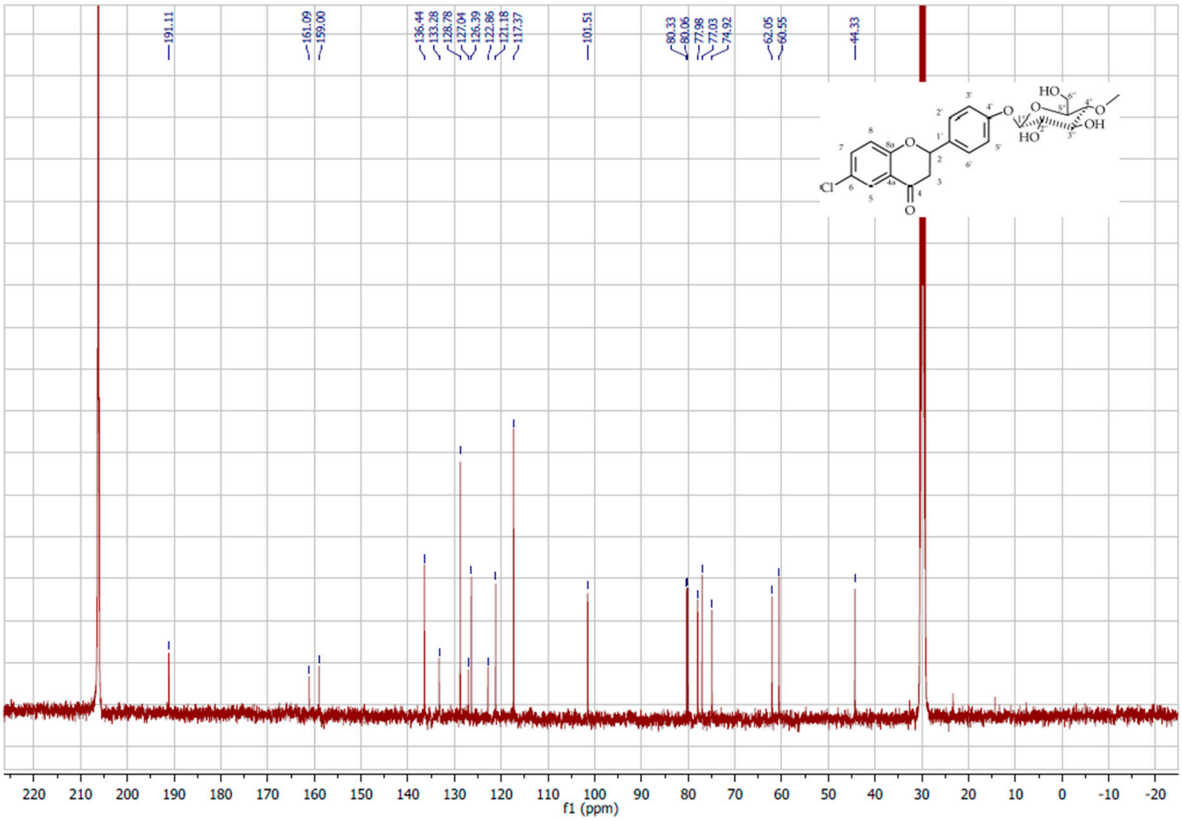

Figure S127. ^{13}C NMR spectrum (*δ,* acetone-d6, 151 MHz) of 6-chloroflavanone 4′-*O*-*β*-D-(4′′-*O*-methyl)-glucopyranoside (**4a**).

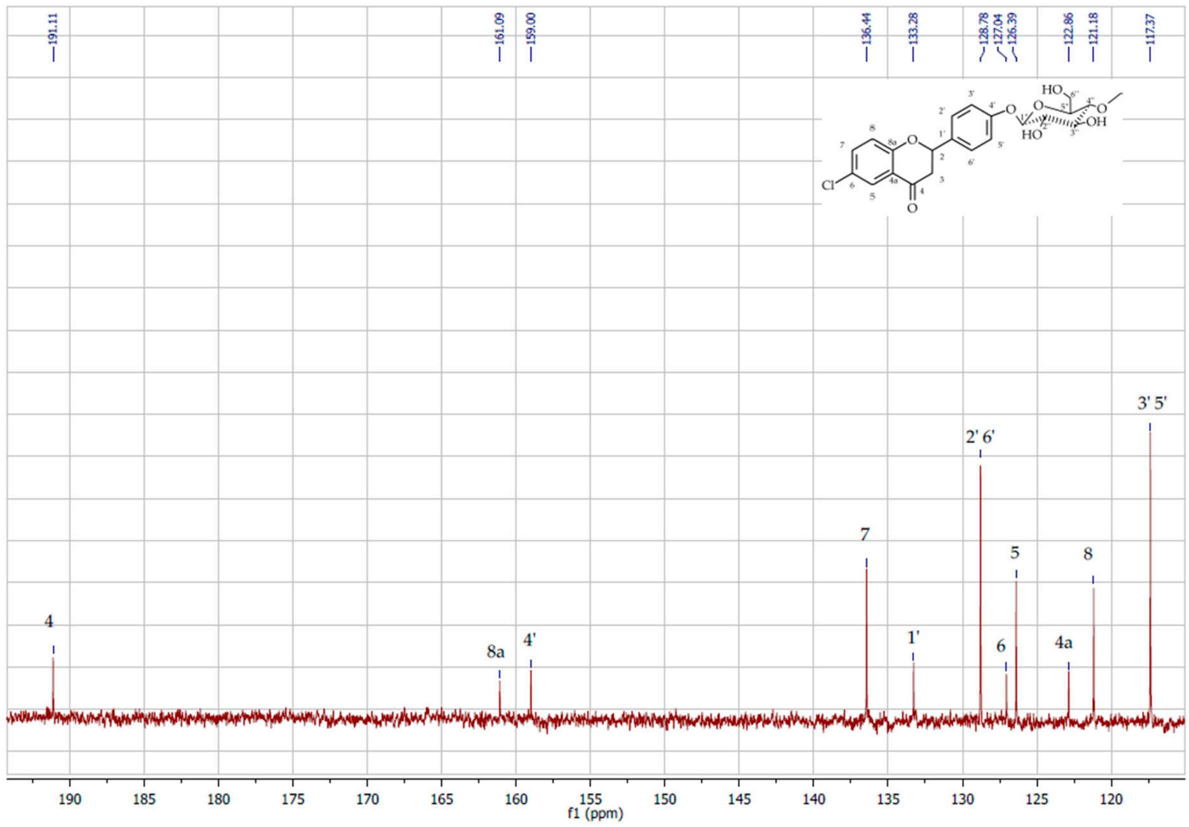

Figure S128. ^{13}C NMR spectrum expansion (*δ*, acetone-d6, 151 MHz) of 6-chloroflavanone 4′-*O*-*β*-D-(4′′-*O*-methyl)-glucopyranoside (**4a**).

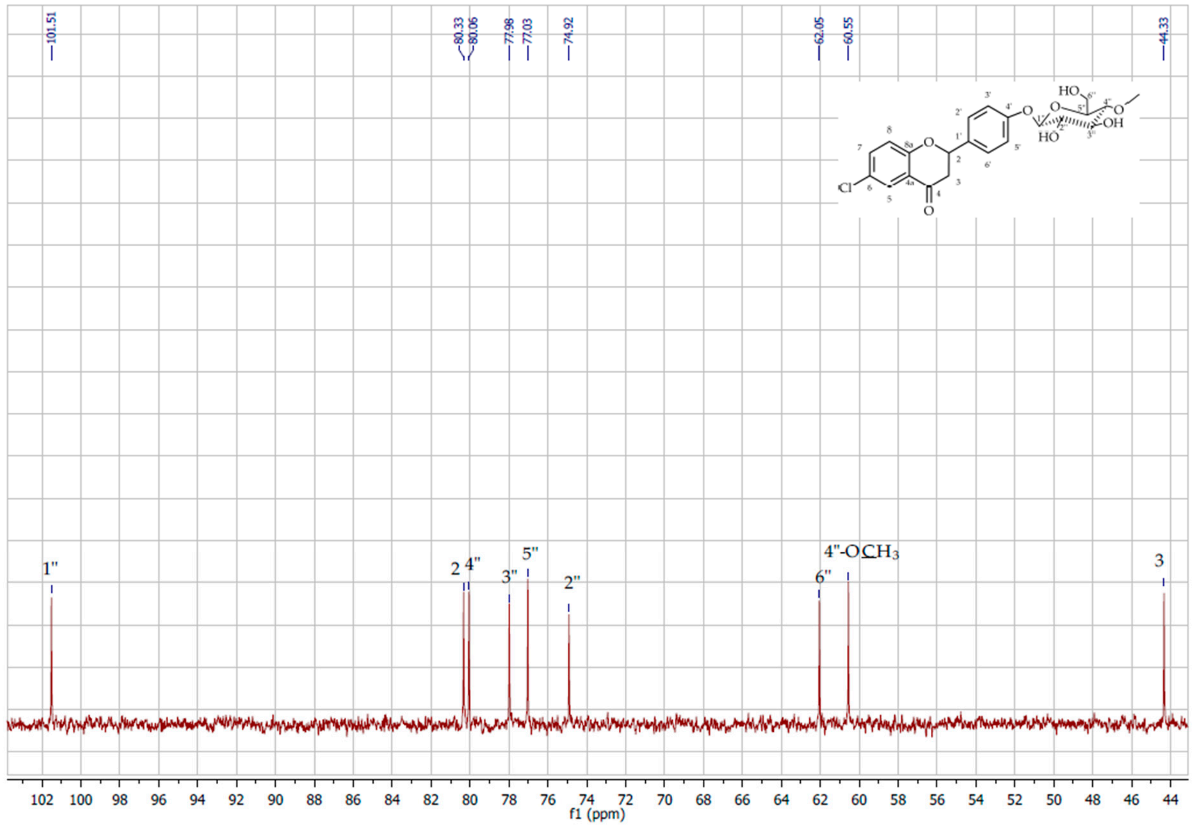

Figure S129. ^{13}C NMR spectrum expansion (*δ*, acetone-d6, 151 MHz) of 6-chloroflavanone 4′-*O*-*β*-D-(4′′-*O*-methyl)-glucopyranoside (**4a**).

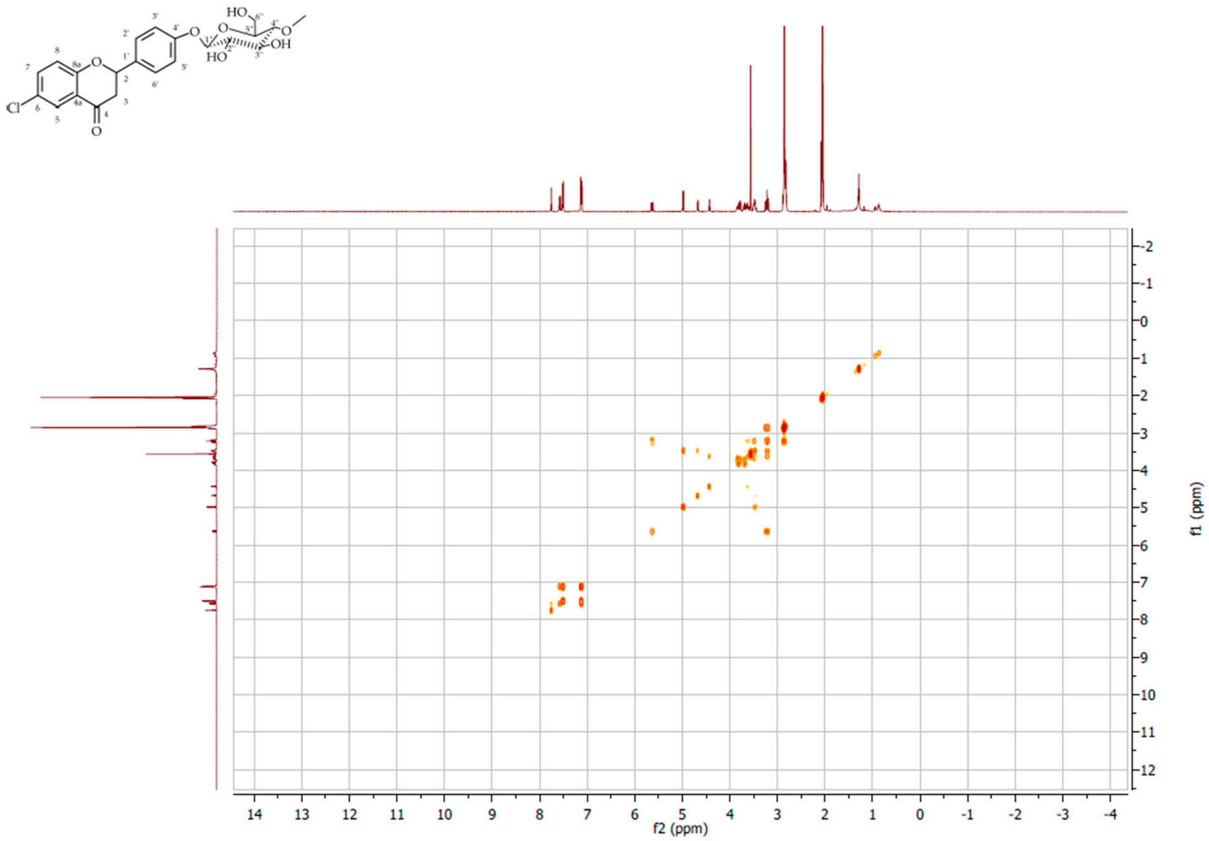

Figure S130. COSY contour map – ^1H x ^1H of 6-chloroflavanone 4′-*O*-*β*-D-(4′′-*O*-methyl)-glucopyranoside (**4a**).

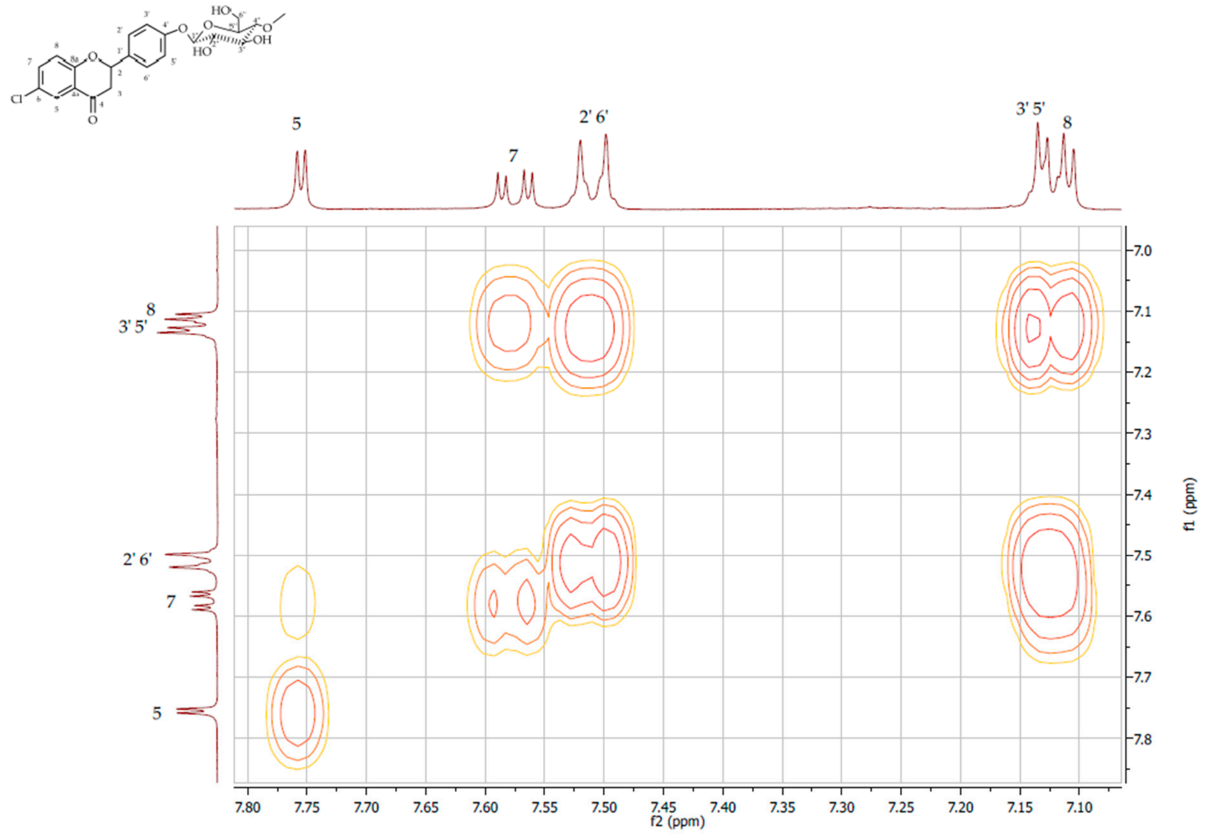

Figure S131. COSY contour map – ^1H x ^1H expansion of 6-chloroflavanone 4′-*O*-*β*-D-(4′′-*O*-methyl)-glucopyranoside (**4a**).

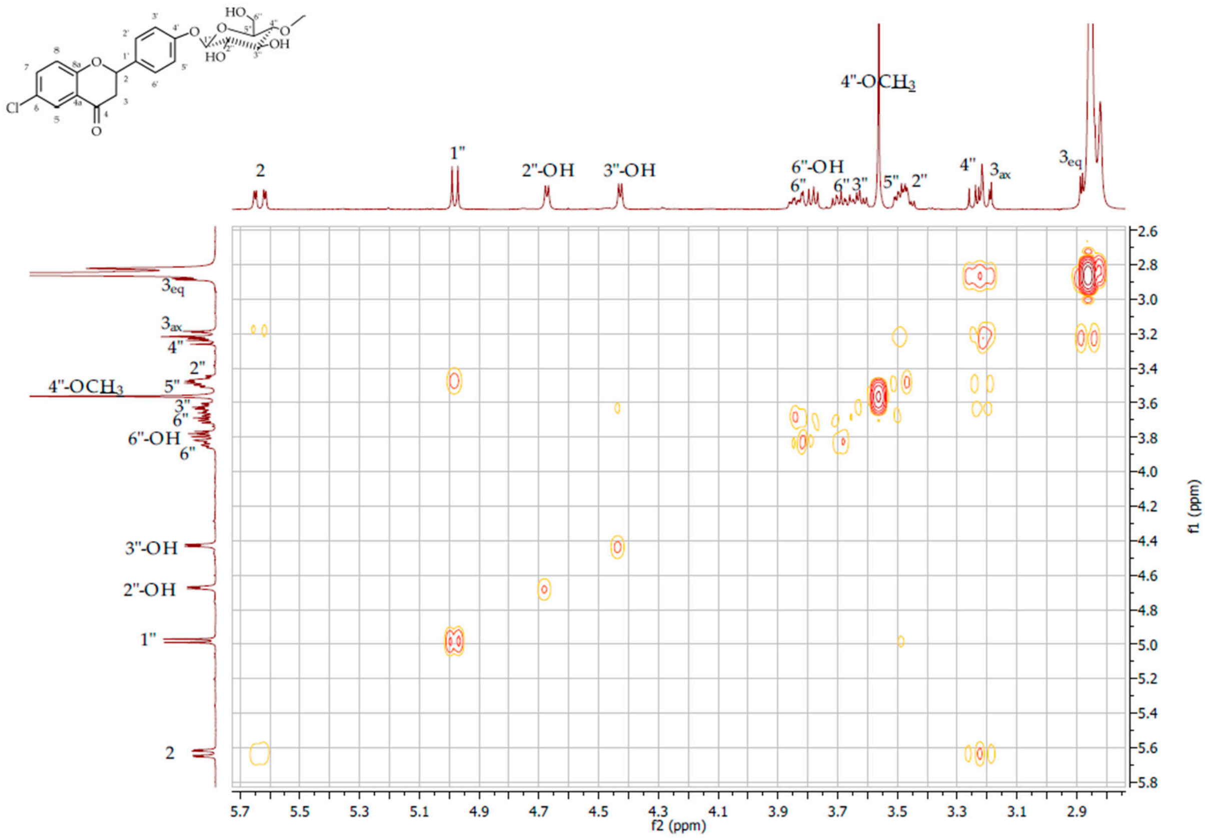

Figure S132. COSY contour map – ^{1}H x ^{1}H expansion of 6-chloroflavanone 4′-*O*-*β*-D-(4′′-*O*-methyl)-glucopyranoside (**4a**).

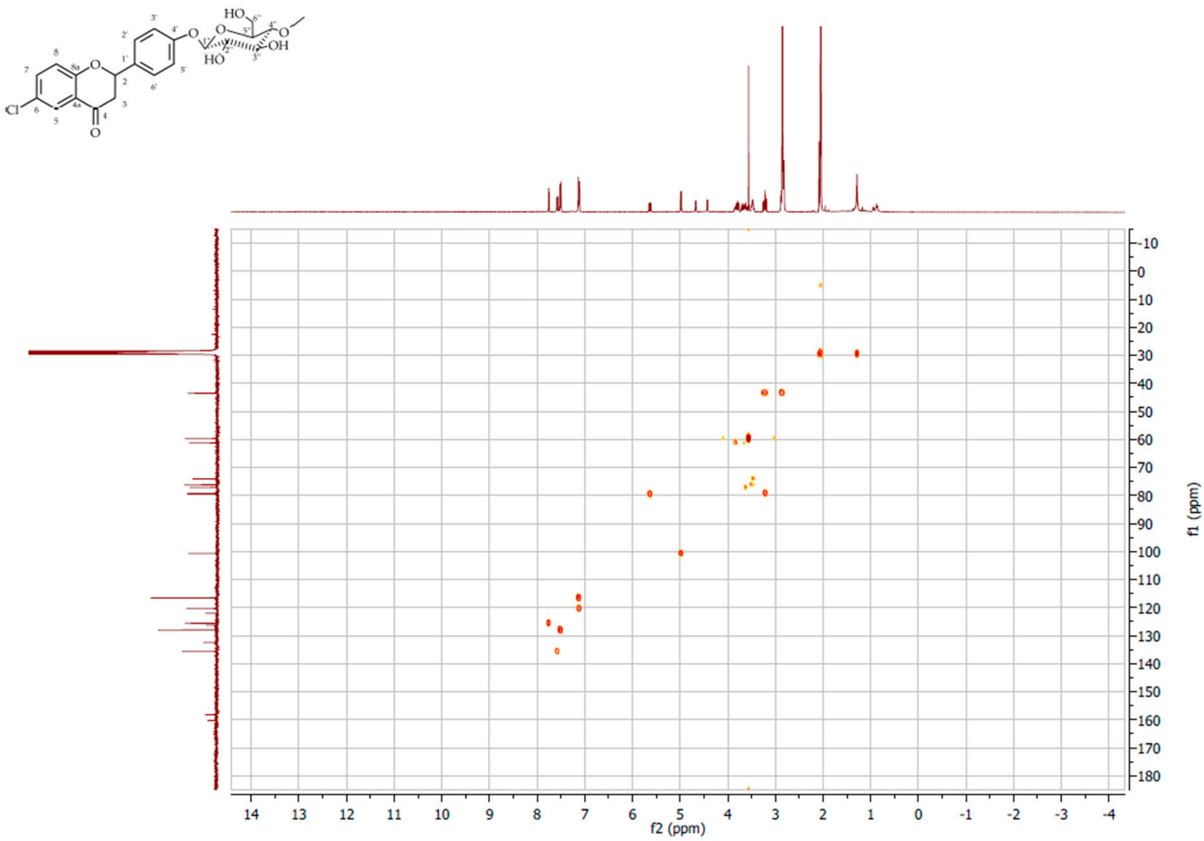

Figure S133. HMQC contour map – [1]H x ^{13}C of 6-chloroflavanone 4′-*O*-*β*-D-(4′′-*O*-methyl)-glucopyranoside (**4a**).

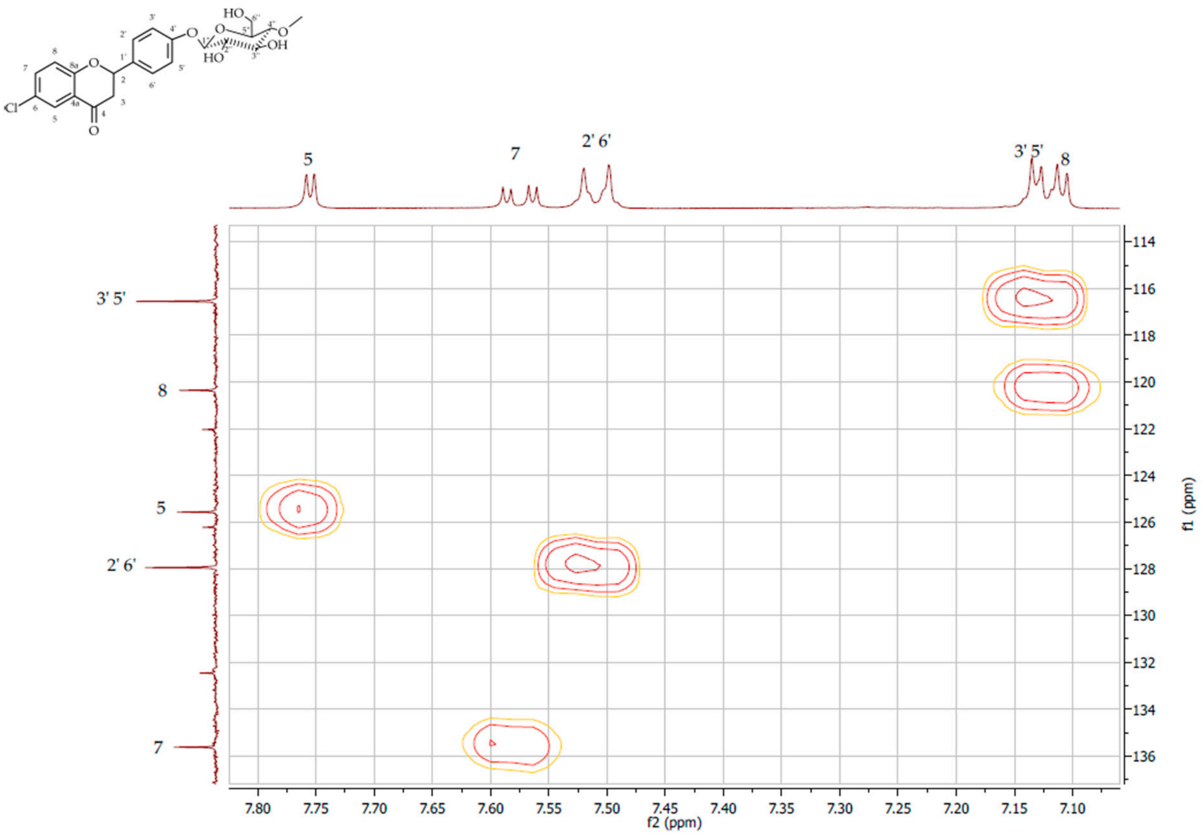

Figure S134. HMQC contour map – [1]H x ^{13}C expansion of 6-chloroflavanone 4′-*O*-*β*-D-(4′′-*O*-methyl)-glucopyranoside (**4a**).

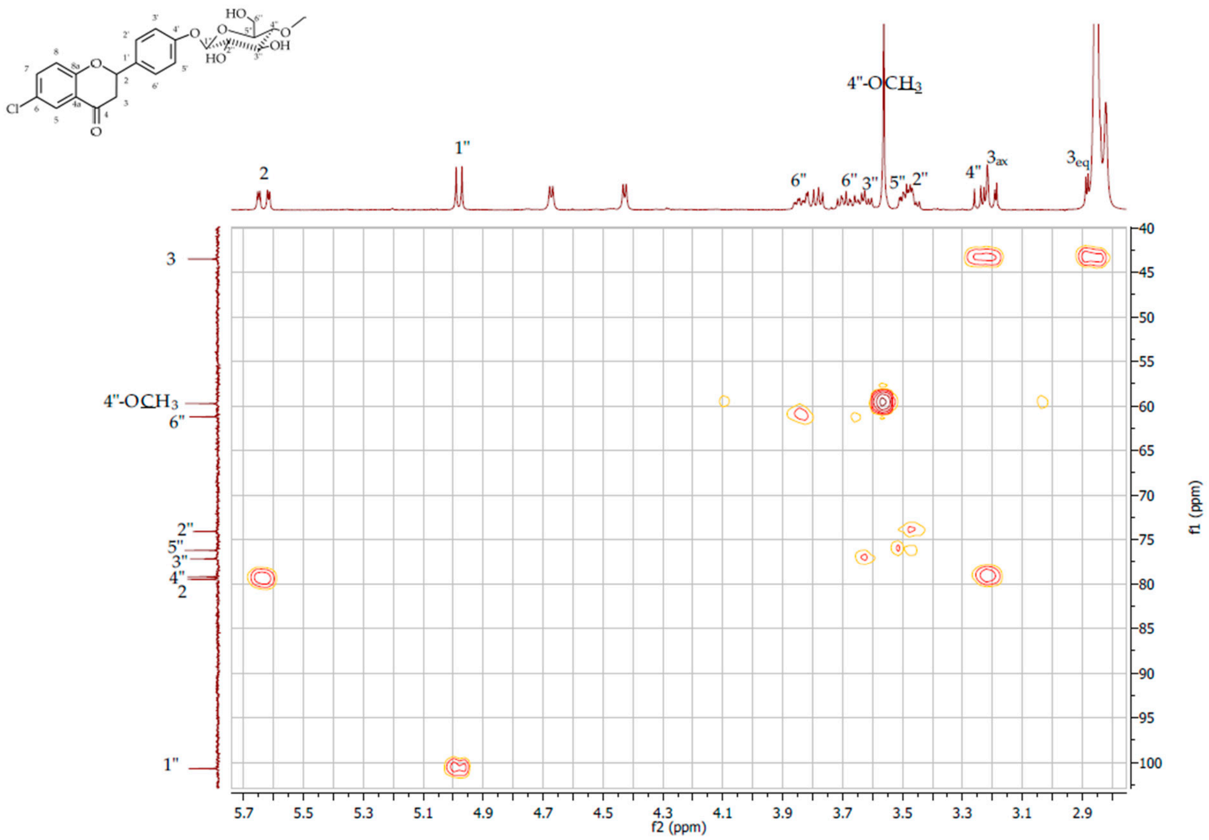

Figure S135. HMQC contour map – [1]H x ^{13}C expansion of 6-chloroflavanone 4′-*O*-*β*-D-(4′′-*O*-methyl)-glucopyranoside (**4a**).

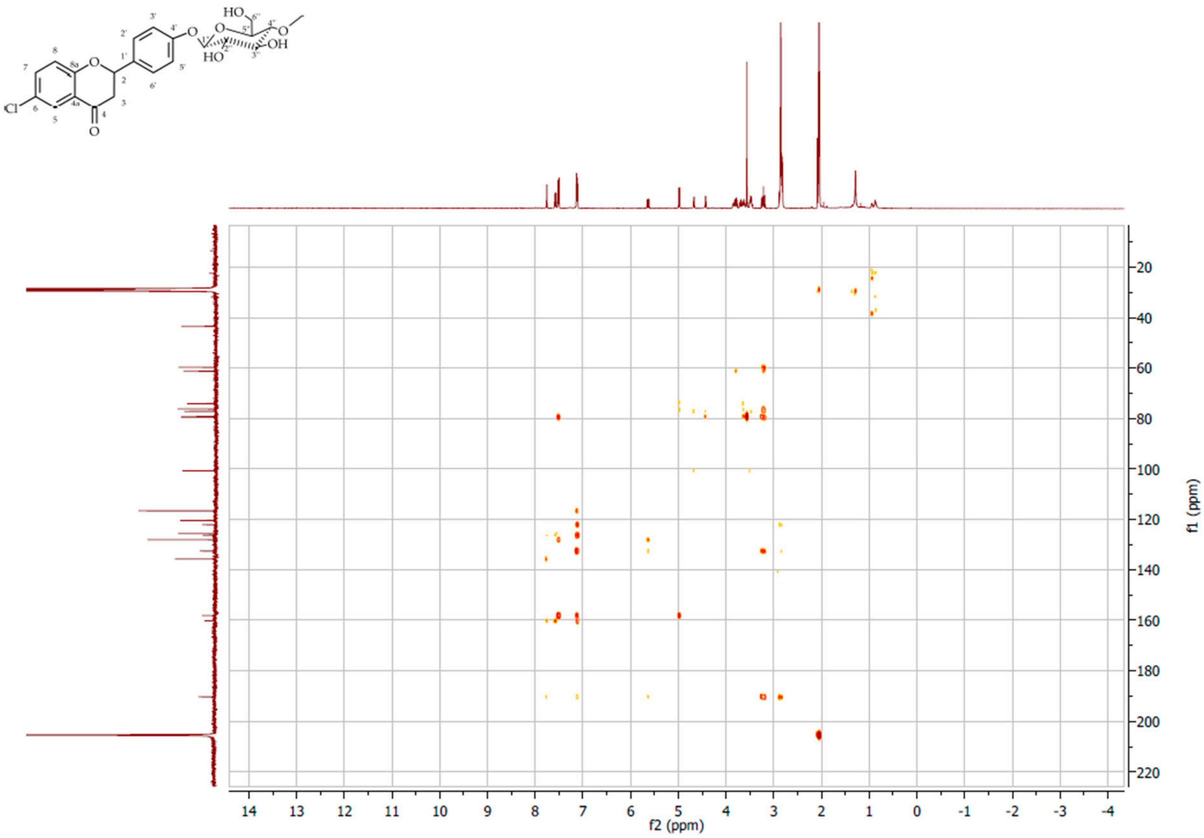

Figure S136. HMBC contour map – ^{1}H x ^{13}C of 6-chloroflavanone 4′-*O*-*β*-D-(4′′-*O*-methyl)-glucopyranoside (**4a**).

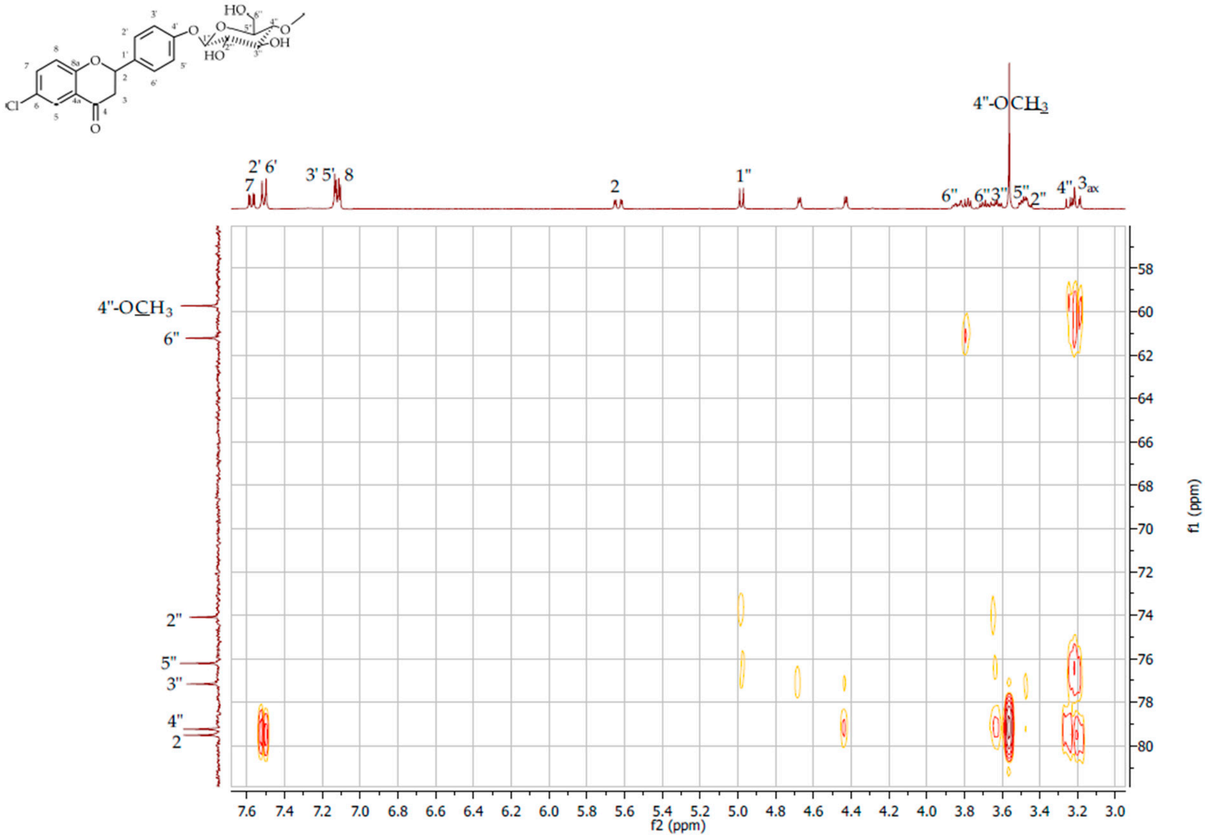

Figure S137. HMBC contour map – ^{1}H x ^{13}C expansion of 6-chloroflavanone 4′-*O*-*β*-D-(4′′-*O*-methyl)-glucopyranoside (**4a**).

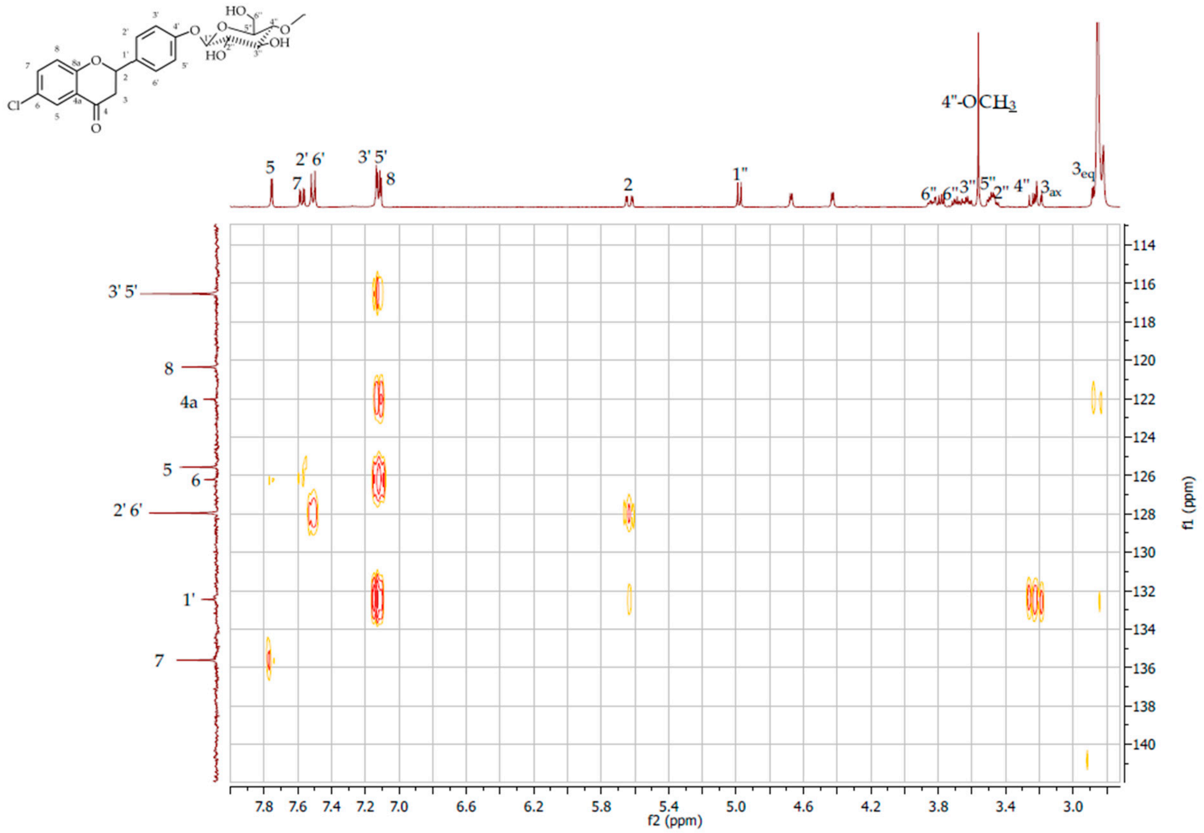

Figure S138. HMBC contour map – ^{1}H x ^{13}C expansion of 6-chloroflavanone 4′-*O*-*β*-D-(4′′-*O*-methyl)-glucopyranoside (**4a**).

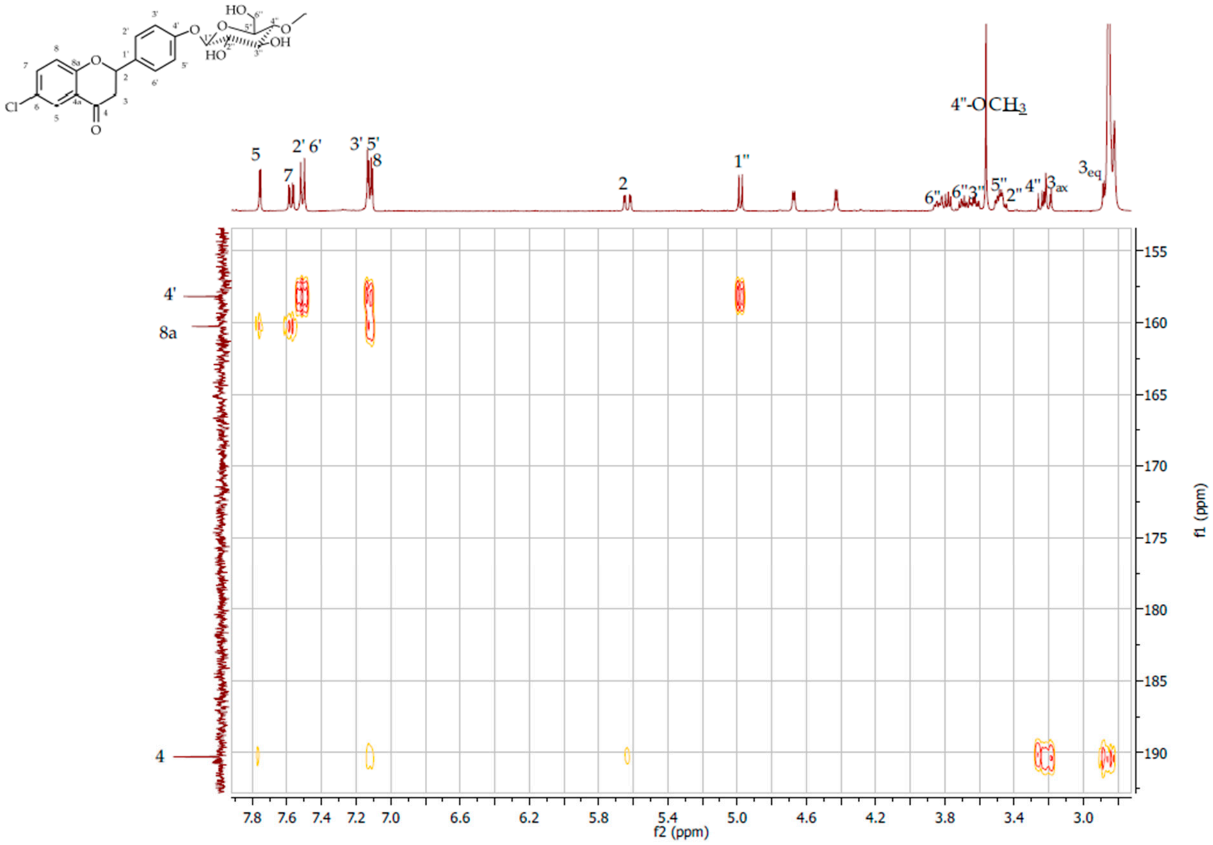

Figure S139. HMBC contour map – ^{1}H x ^{13}C expansion of 6-chloroflavanone 4′-*O*-*β*-D-(4′′-*O*-methyl)-glucopyranoside (**4a**).

Molecule 1

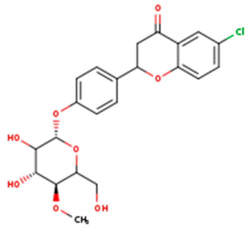

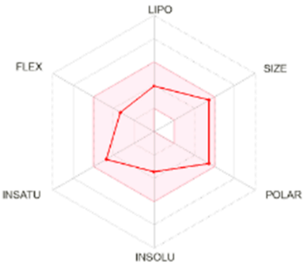

SMILES CO[C@@H]1C(CO)O[C@H](C([C@H]1O)O)Oc1ccc(cc1)C1CC(=O)c2c(O1)ccc(c2)Cl

Physicochemical Properties	
Formula	C22H23ClO8
Molecular weight	450.87 g/mol
Num. heavy atoms	31
Num. arom. heavy atoms	12
Fraction Csp3	0.41
Num. rotatable bonds	5
Num. H-bond acceptors	8
Num. H-bond donors	3
Molar Refractivity	109.39
TPSA	114.68 Å²

Lipophilicity	
Log $P_{o/w}$ (iLOGP)	2.76
Log $P_{o/w}$ (XLOGP3)	1.36
Log $P_{o/w}$ (WLOGP)	1.55
Log $P_{o/w}$ (MLOGP)	0.29
Log $P_{o/w}$ (SILICOS-IT)	2.02
Consensus Log $P_{o/w}$	1.60

Water Solubility	
Log *S* (ESOL)	-3.45
Solubility	1.60e-01 mg/ml ; 3.56e-04 mol/l
Class	Soluble
Log *S* (Ali)	-3.37
Solubility	1.92e-01 mg/ml ; 4.26e-04 mol/l
Class	Soluble
Log *S* (SILICOS-IT)	-4.16
Solubility	3.13e-02 mg/ml ; 6.95e-05 mol/l
Class	Moderately soluble

Pharmacokinetics	
GI absorption	High
BBB permeant	No
P-gp substrate	Yes
CYP1A2 inhibitor	No
CYP2C19 inhibitor	No
CYP2C9 inhibitor	No
CYP2D6 inhibitor	No
CYP3A4 inhibitor	Yes
Log K_p (skin permeation)	-8.08 cm/s

Druglikeness	
Lipinski	Yes; 0 violation
Ghose	Yes
Veber	Yes
Egan	Yes
Muegge	Yes
Bioavailability Score	0.55

Medicinal Chemistry	
PAINS	0 alert
Brenk	0 alert
Leadlikeness	No; 1 violation: MW>350
Synthetic accessibility	4.94

Figure S140. 6-Chloroflavanone 4′-*O*-*β*-D-(4′′-*O*-methyl)-glucopyranoside (**4a**) physicochemical and ADME parameters prediction using the SwissADME modelling.

Molecule 1

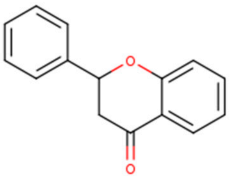

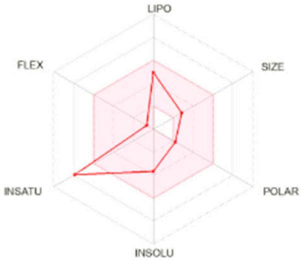

SMILES O=C1CC(Oc2c1cccc2)c1ccccc1

Physicochemical Properties	
Formula	C15H12O2
Molecular weight	224.25 g/mol
Num. heavy atoms	17
Num. arom. heavy atoms	12
Fraction Csp3	0.13
Num. rotatable bonds	1
Num. H-bond acceptors	2
Num. H-bond donors	0
Molar Refractivity	65.50
TPSA	26.30 Å²

Lipophilicity	
Log $P_{o/w}$ (iLOGP)	2.41
Log $P_{o/w}$ (XLOGP3)	3.14
Log $P_{o/w}$ (WLOGP)	3.07
Log $P_{o/w}$ (MLOGP)	2.47
Log $P_{o/w}$ (SILICOS-IT)	3.57
Consensus Log $P_{o/w}$	2.93

Water Solubility	
Log *S* (ESOL)	-3.66
Solubility	4.85e-02 mg/ml ; 2.16e-04 mol/l
Class	Soluble
Log *S* (Ali)	-3.36
Solubility	9.75e-02 mg/ml ; 4.35e-04 mol/l
Class	Soluble
Log *S* (SILICOS-IT)	-5.15
Solubility	1.59e-03 mg/ml ; 7.08e-06 mol/l
Class	Moderately soluble

Pharmacokinetics	
GI absorption	High
BBB permeant	Yes
P-gp substrate	No
CYP1A2 inhibitor	Yes
CYP2C19 inhibitor	No
CYP2C9 inhibitor	No
CYP2D6 inhibitor	No
CYP3A4 inhibitor	No
Log K_p (skin permeation)	-5.44 cm/s

Druglikeness	
Lipinski	Yes; 0 violation
Ghose	Yes
Veber	Yes
Egan	Yes
Muegge	Yes
Bioavailability Score	0.55

Medicinal Chemistry	
PAINS	0 alert
Brenk	0 alert
Leadlikeness	No; 1 violation: MW<250
Synthetic accessibility	2.77

Figure S141. Flavanone (**5**) physicochemical and ADME parameters prediction using the SwissADME modelling.
